# *In Silico* Screening of Semi-Synthesized Compounds as Potential Inhibitors for SARS-CoV-2 Papain-like Protease: Pharmacophoric Features, Molecular Docking, ADMET, Toxicity and DFT Studies

**DOI:** 10.3390/molecules26216593

**Published:** 2021-10-30

**Authors:** Mohamed S. Alesawy, Eslam B. Elkaeed, Aisha A. Alsfouk, Ahmed M. Metwaly, Ibrahim H. Eissa

**Affiliations:** 1 Pharmaceutical Medicinal Chemistry and Drug Design Department, Faculty of Pharmacy (Boys), Al-Azhar University, Cairo 11884, Egypt; mohammedalesawy@azhar.edu.eg; 2 Department of Pharmaceutical Sciences, College of Pharmacy, Almaarefa University, Ad Diriyah, Riyadh 13713, Saudi Arabia; ikaeed@mcst.edu.sa; 3 Department of Pharmaceutical Sciences, College of Pharmacy, Princess Nourah Bint Abdulrahman University, Riyadh 11671, Saudi Arabia; aaalsfouk@pnu.edu.sa; 4 Pharmacognosy and Medicinal Plants Department, Faculty of Pharmacy (Boys), Al-Azhar University, Cairo 11884, Egypt; 5 Biopharmaceutical Products Research Department, Genetic Engineering and Biotechnology Research Institute, City of Scientific Research and Technological Applications (SRTA-City), Alexandria 21934, Egypt

**Keywords:** COVID-19, papain-like protease, pharmacophore, molecular docking, ADMET, toxicity, DFT, semi-synthesized

## Abstract

Papain-like protease is an essential enzyme in the proteolytic processing required for the replication of SARS-CoV-2. Accordingly, such an enzyme is an important target for the development of anti-SARS-CoV-2 agents which may reduce the mortality associated with outbreaks of SARS-CoV-2. A set of 69 semi-synthesized molecules that exhibited the structural features of SARS-CoV-2 papain-like protease inhibitors (PLPI) were docked against the coronavirus papain-like protease (PLpro) enzyme (PDB ID: (4OW0). Docking studies showed that derivatives **34** and **58** were better than the co-crystallized ligand while derivatives **17**, **28**, **31**, **40**, **41**, **43**, **47**, **54**, and **65** exhibited good binding modes and binding free energies. The pharmacokinetic profiling study was conducted according to the four principles of the Lipinski rules and excluded derivative 31. Furthermore, ADMET and toxicity studies showed that derivatives **28**, **34**, and **47** have the potential to be drugs and have been demonstrated as safe when assessed via seven toxicity models. Finally, comparing the molecular orbital energies and the molecular electrostatic potential maps of **28**, **34**, and **47** against the co-crystallized ligand in a DFT study indicated that **28** is the most promising candidate to interact with the target receptor (PLpro).

## 1. Introduction

As of 10 September 2021, the uncontrolled SARS-CoV-2 had infected 222,406,582 and killed 4,592,934 people all over the world, according to the WHO [1]. The alarming spread necessitates contentious work until a cure is discovered. Computational (*in silico*, computer-aided, or cheminformatics) approaches have been used in various fields related to drug discovery [2], such as molecular docking [3,4,5], pharmacophore studies [6], drug molecular design [7,8], QSAR [9], toxicity prediction [10,11,12], ADMET assessment [13,14,15], and DFT calculations [16].

These approaches have been practiced fruitfully and frequently in several scientific studies to find a treatment against COVID-19 with the advantage of taking less effort, time, and cost [17,18,19,20,21].

Since the first historical records, people counted on natural sources around them to get food, cures, and even beauty tools [22,23]. Scientists authenticated the healing effects of plants [24,25] and, recently, microorganisms [26,27]. Plants and microorganisms naturally synthesize diverse chemical compounds as nitrogenous alkaloids [28], flavonoids [29,30], saponins [31,32], steroids [33], chromenes [34], α-pyrones [35], diterpenoids [36] and sesquiterpenoids [37,38]. 

More than 30% of FDA-approved drugs were based on natural products from 1981 to 2014 [39]. The main aim of the semi-synthesis approach in developing natural products is to obtain several analogs allowing the discovery of stronger drugs and even repurposing [40]. Additionally, it’s much easier to conduct structure-activity relationship investigations, which gives the advantage of obtaining novel bioactive molecules and modifying drug-likeness, pharmacodynamic and pharmacokinetic characteristics [41].

Papain-like protease (PLpro) is a pivotal enzyme in the coronavirus that has two major functions. The first one is included in the generation of an efficient replicase complex via the processing mechanism of viral polyproteins [42]. Additionally, the PLpro plays a vital role against the immunity of the host (human) via performing different cleaving modifications on the proteins of human immune responses [43]. Consequently, the inhibition of such a vital protein could be a great step toward finding a cure against COVID-19. Accordingly, we utilized PLpro as a potential target in our virtual screening

**Figure 1:**
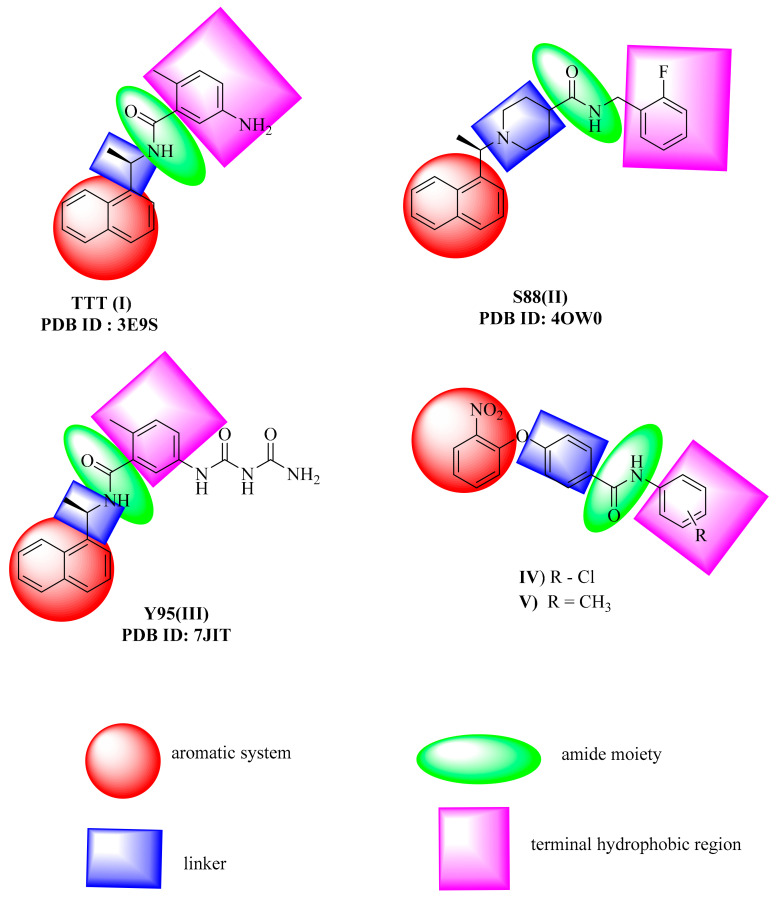
Essential pharmacophoric features of SARS-CoV-2 PLPIs.

At Protein Data Bank, there are three crystal structures of coronavirus papain-like proteases (PLpro) with their co-crystallized ligands. The first crystal structure has the PDB ID of 3E9S, and the co-crystallized ligand is 5-amino-2-methyl-*N*-[(1R)-1-naphthalen-1-ylethyl]benzamide (TTT) I [44]. The second one has the PDB ID of 4OW0, and the co-crystallized ligand is N-[(3-fluorophenyl)methyl]-1-[(1R)-1-naphthalen-1-ylethyl] piperidine-4-carboxamide (S88) II [45]. The third one has the PDB ID of 7JIT, and the co-crystallized ligand is 5-[(carbamoylcarbamoyl)amino]-2-methyl-*N*-[(1R)-1-(naphthalen-1-yl) ethyl]benzamide (Y95) III [46]. The reported SARS-CoV-2 papain-like protease inhibitors (PLPIs) have the following four main pharmacophoric features: (1) aromatic system, (2) linker, (3) amide moiety, and (4) terminal hydrophobic region [47]. These features were satisfied in several PLPIs as shown in Figure 1. In the literature, compounds IV and V showed promising activity against Adenovirus, HSV-1, coxsackievirus, and SAR-CoV-2. In addition, these compounds showed a good binding mode against PLP. Furthermore, such compounds have the same features of PLP inhibitors [48].

**Figure 2:**
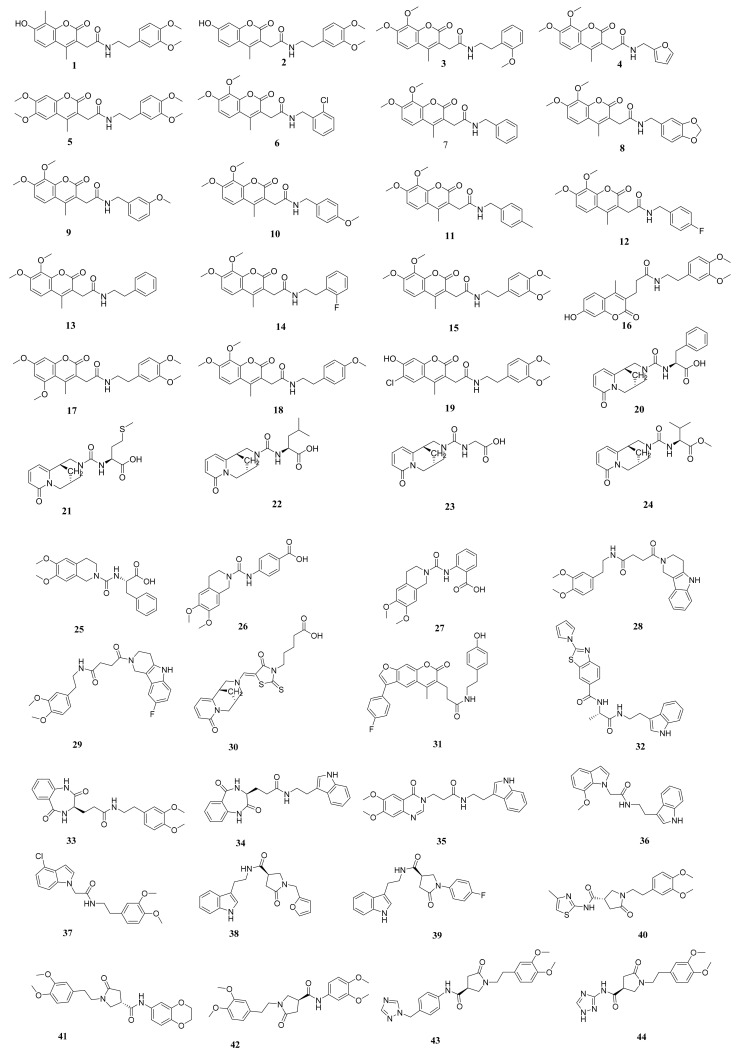

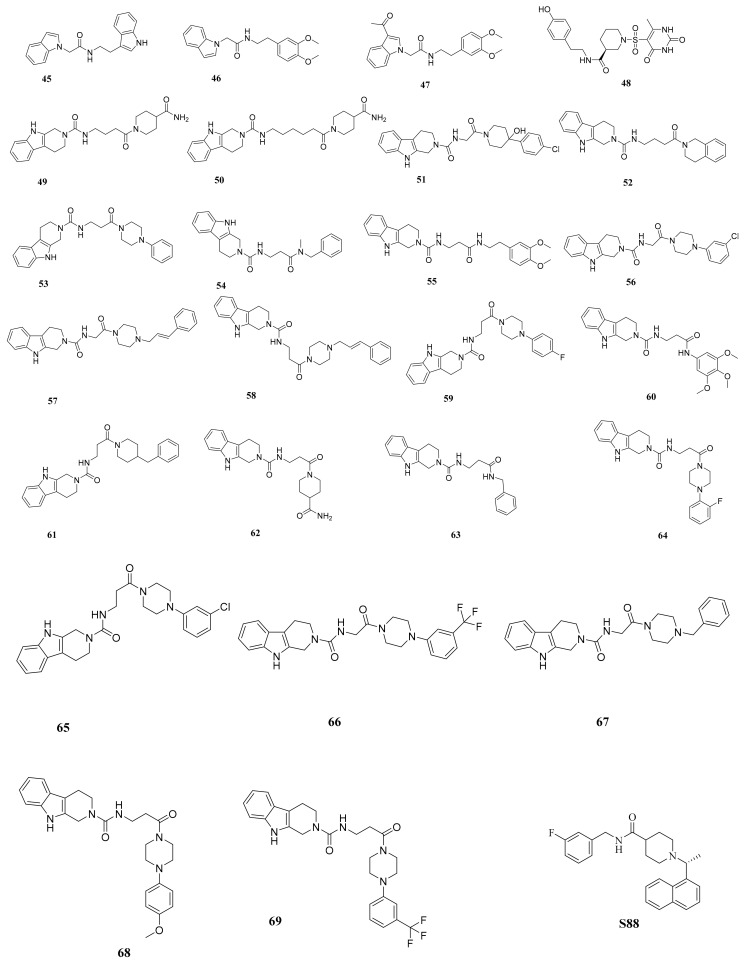
The chemical structures of the examined molecules.

**Figure 3:**
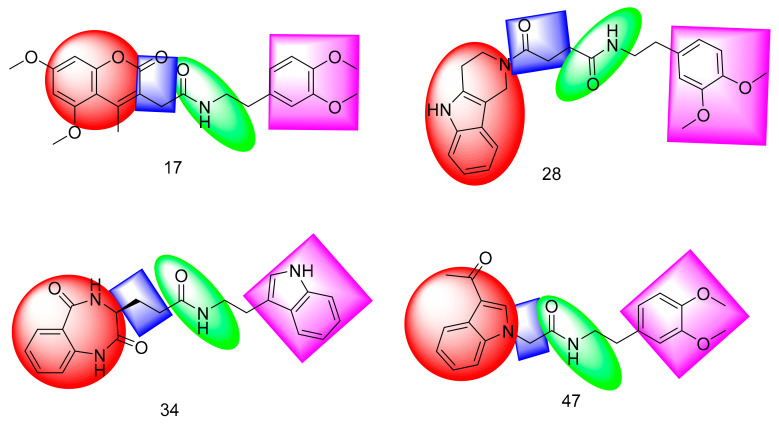
Representative sample of the examined semi-synthesized molecules having the main features of PLPIs.

A set of 69 semi-synthesized molecules (Figure 2) that have the essential features of SARS-CoV-2 PLPIs was downloaded from the Eximed laboratory website [49] and used in this research. The selected semi-synthesized molecules were screened against PLpro through docking studies. Figure 3 demonstrates the presence of those features in a representative sample of the examined semi-synthesized molecules. The examined molecules that showed a good binding modes and high-affinity values against PLpro were further *in silico* examined for their drug-likeness characters using the Lipinski rule of five, ADMET, and toxicity profiling. The most promising derivatives were subjected to DFT studies to get additional insight into their electron distribution.

## 2. Results

### 2.1. Docking Studies 

**Figure 4:**
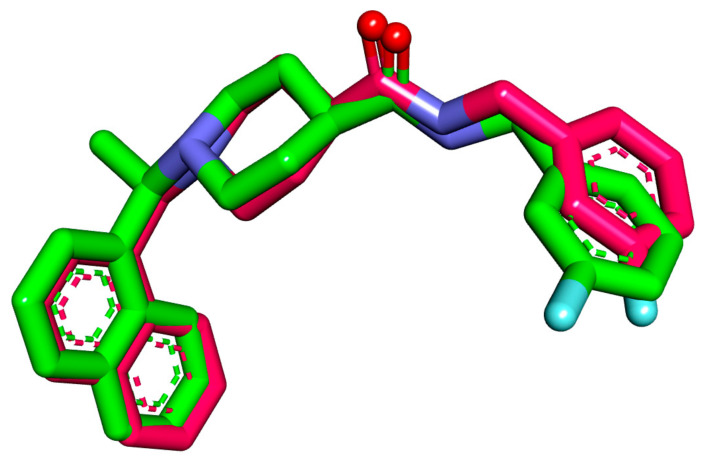
Superimposition of the redocked pose colored in pink against the co-crystallized one colored in green (**S88**) in PLpro active site.

MOE software was used to conduct docking studies (Supplementary Materials) on the investigated derivatives, with co-crystallized ligand **S88** as a reference. The study aimed at getting a deeper insight into the binding modes of the examined semi-synthesized molecules in the active site of the coronavirus papain-like protease (PLpro) enzyme (PDB ID: (4OW0)). The docking method was validated through redocking of the co-crystallized ligand in the enzyme active site. The protocol’s applicability was confirmed through the demonstration of small RMSD (0.54 Å) between the co-crystallized pose and the re-docked one (Figure 4).

In this study, we relied on the corrected mode of binding of the examined semi-synthesized molecules and S88 as well as the values of the binding free energy (ΔG) between them. Table 1 illustrates the calculated ΔG of the tested semi-synthesized molecules and (**S88**) against the coronavirus papain-like protease enzyme. The semi-synthesized molecules **34** and **58** showed affinity values of −8.97 and −8.65, respectively, that were higher than that of the redocked ligand **S88** (−8.59 kcal/mol). Moreover, the semi-synthesized molecules **17**, **28**, **31**, **40**, **41**, **43**, **47**, **54** and **65** revealed binding energy scores ranging from −8.33 to −8.57 kcal/mol, which were highly close to the redocked ligand **S88**. On the other hand, the other semi-synthesized molecules demonstrated affinity values lower than **S88**.

**Figure 5:**
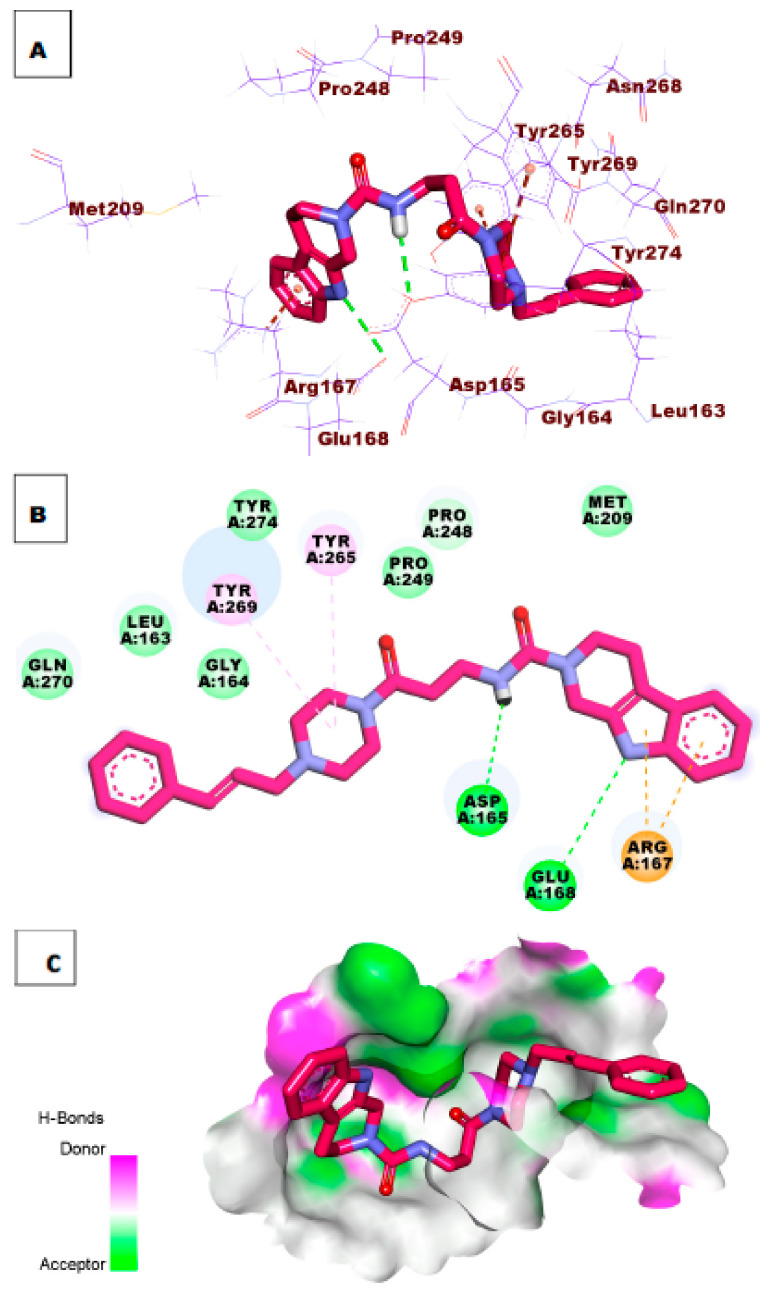
(**A**) 3D of **S88** docked into the active site of PLpro. (**B**) 2D of **S88** docked into the active site of PLpro. (**C**) Surface mapping of **S88** docked into the active site of PLpro.

The proposed binding mode of the redocked ligand **S88** revealed an affinity value of −8.59 kcal/mol. This high binding affinity is probably attributed to the formation of two hydrogen-bonding interactions. One was formed between the N-H group of the amide moiety and Tyr269 while the other was formed between the nitrogen atom of the pyridine ring and Asp165. Additionally, the naphthyl moiety formed four hydrophobic interactions with Tyr269 and Pro249. These results were found to be consistent with the reported data [45] (Figure 5).

**Figure 6:**
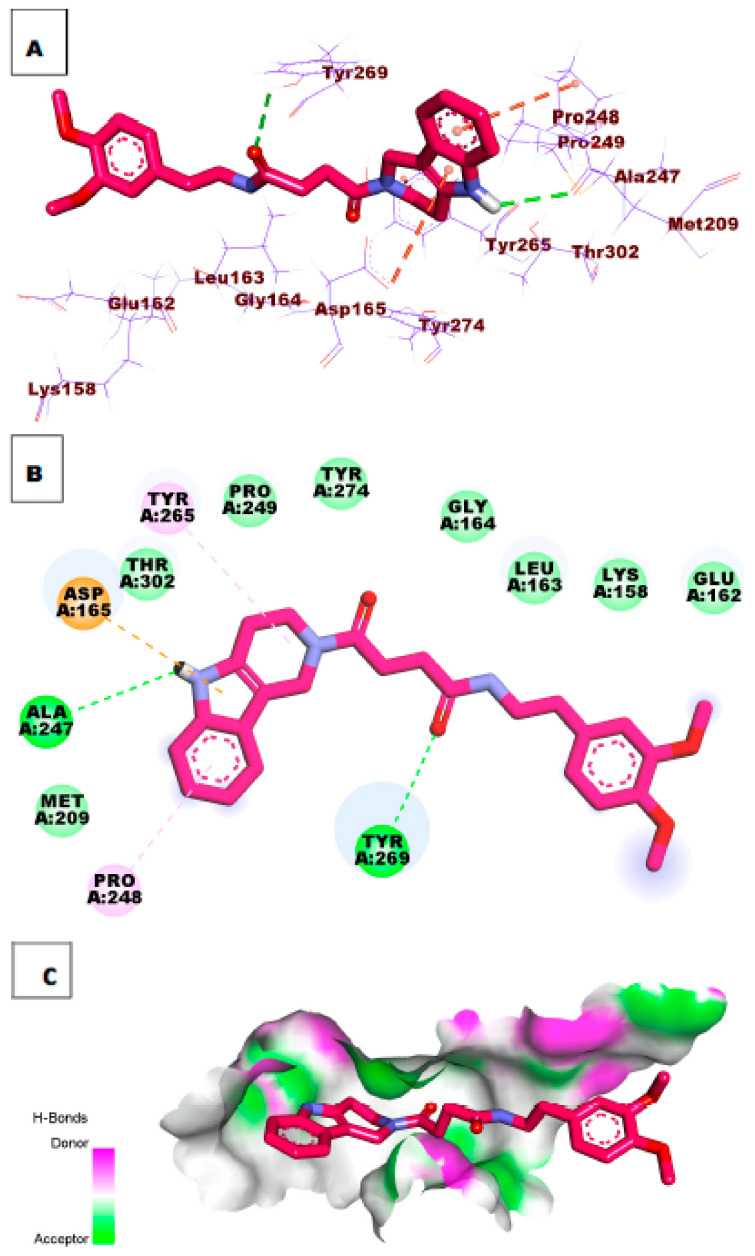
(**A**) 3D of compound **28** docked into the active site of PLpro. (**B**) 2D of compound **28** docked into the active site of PLpro. (**C**) Surface mapping of compound **28** docked into the active site of PLpro.

The docking simulation of compound **28** revealed that it has a good fitting into the enzyme active site with a docking score of −8.48 kcal/mol. The oxygen of the carbonyl group formed one hydrogen bond with the essential amino acid Tyr269. Additionally, the NH group of the pyrrole ring formed one hydrogen bond with Ala247. The 2,3,4,5-tetrahydro-1*H*-pyrido[4,3-*b*]indole moiety formed Tyr265, Asp165, and Pro248 (Figure 6).

**Figure 7:**
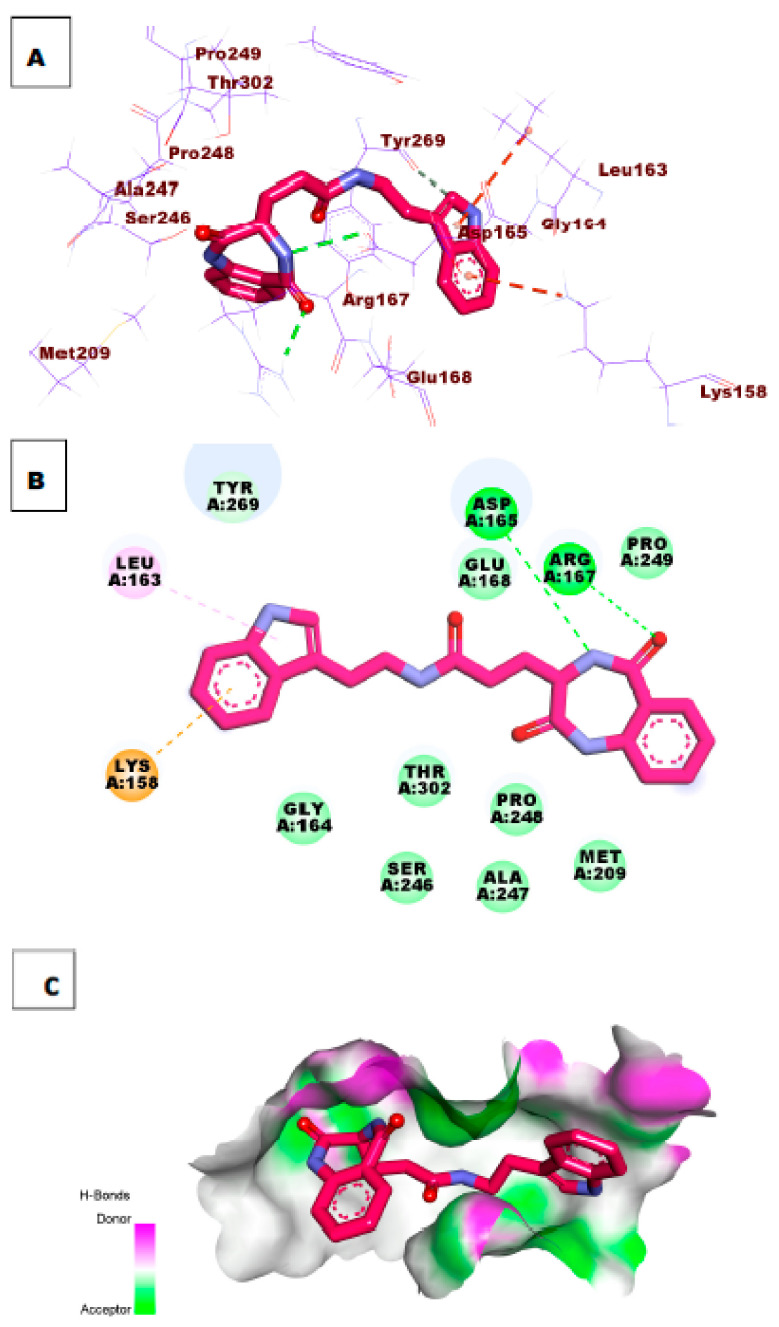
(**A**) 3D of compound **34** docked into the active site of PLpro. (**B**) 2D of compound **34** docked into the active site of PLpro. (**C**) Surface mapping of compound **34** docked into the active site of PLpro.

As illustrated in Figure 7, compound **34** possessed a significant potential binding affinity (ΔG = −8.97 kcal/mol) into the papain-like protease active site. This high binding affinity, which is higher than ligand **S88**, presumably attributed to the formation of one hydrogen bond interaction with Arg167. In addition, the 1*H*-indole formed two hydrophobic interactions with Lys158 and Leu163.

**Figure 8:**
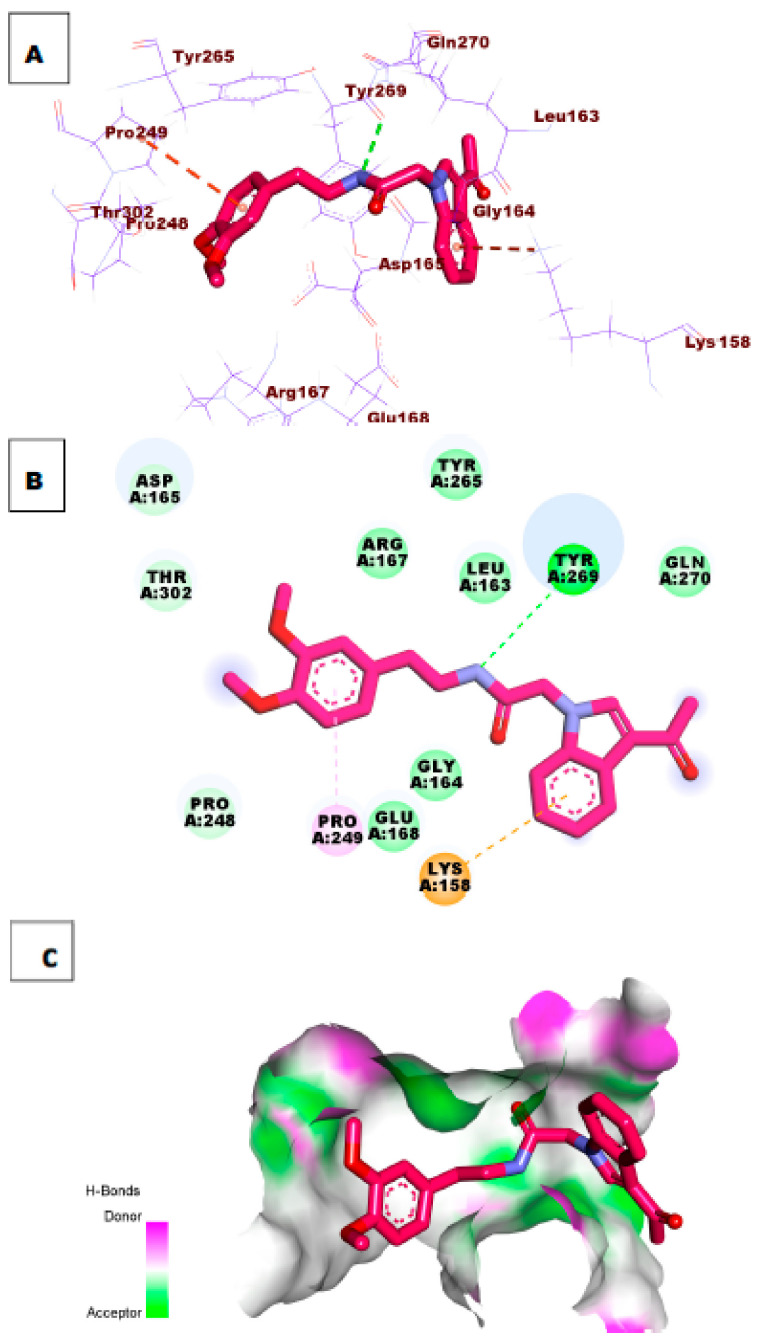
(**A**) 3D of compound **47** docked into the active site of PLpro. (**B**) 2D of compound **47** docked into the active site of PLpro. (**C**) Surface mapping of compound **47** docked into the active site of PLpro.

**Figure 9:**
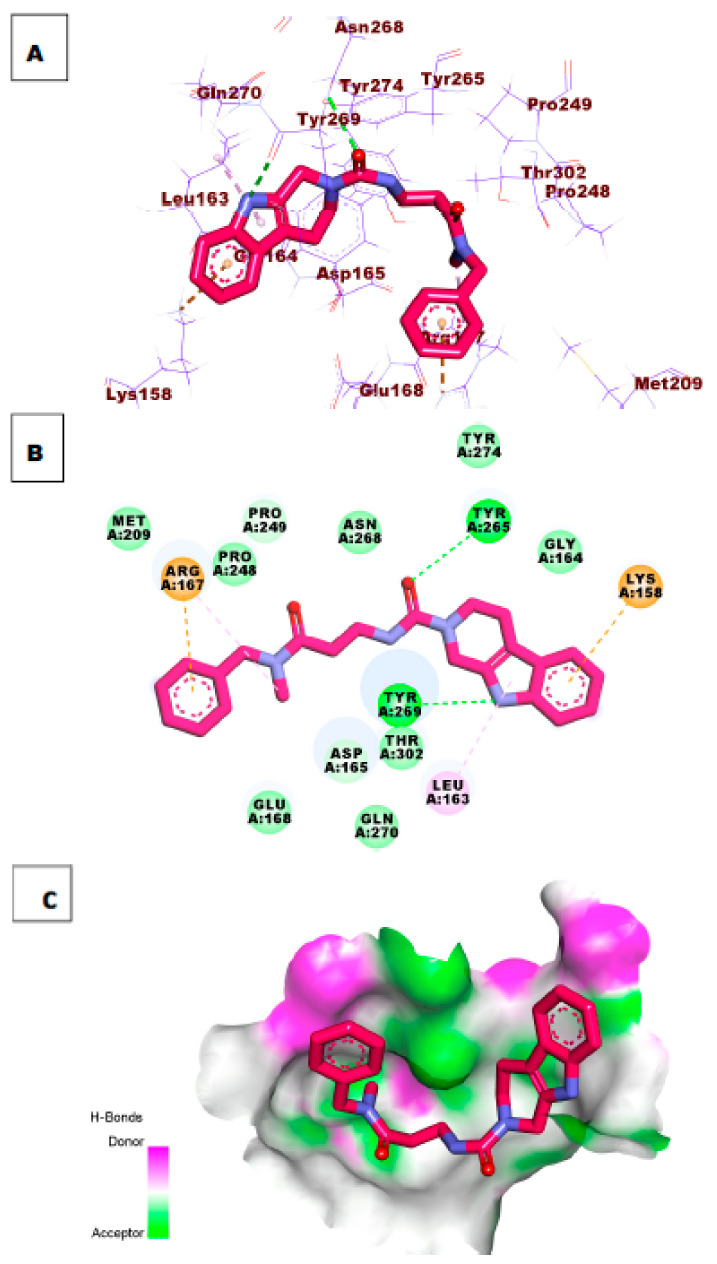
(**A**) 3D of compound **54** docked into the active site of PLpro. (**B**) 2D of compound **54** docked into the active site of PLpro. (**C**) Surface mapping of compound **54** docked into the active site of PLpro.

Investigation of the top docking poses of compounds **47** and **54** (affinity values of −8.57 and 8.33 kcal/mol) respectively, demonstrated that compound **47** formed one hydrogen bond interaction with Tyr269. In addition, it formed two hydrophobic interactions with Pro249 and Lys158. Compound **54** formed two hydrogen bonds with the essential amino acid Tyr269 and Tyr265. In addition, it formed two hydrophobic interactions with Lys158 and Arg167 in the active site of PLpro. Figure 8 and Figure 9.

**Figure 10:**
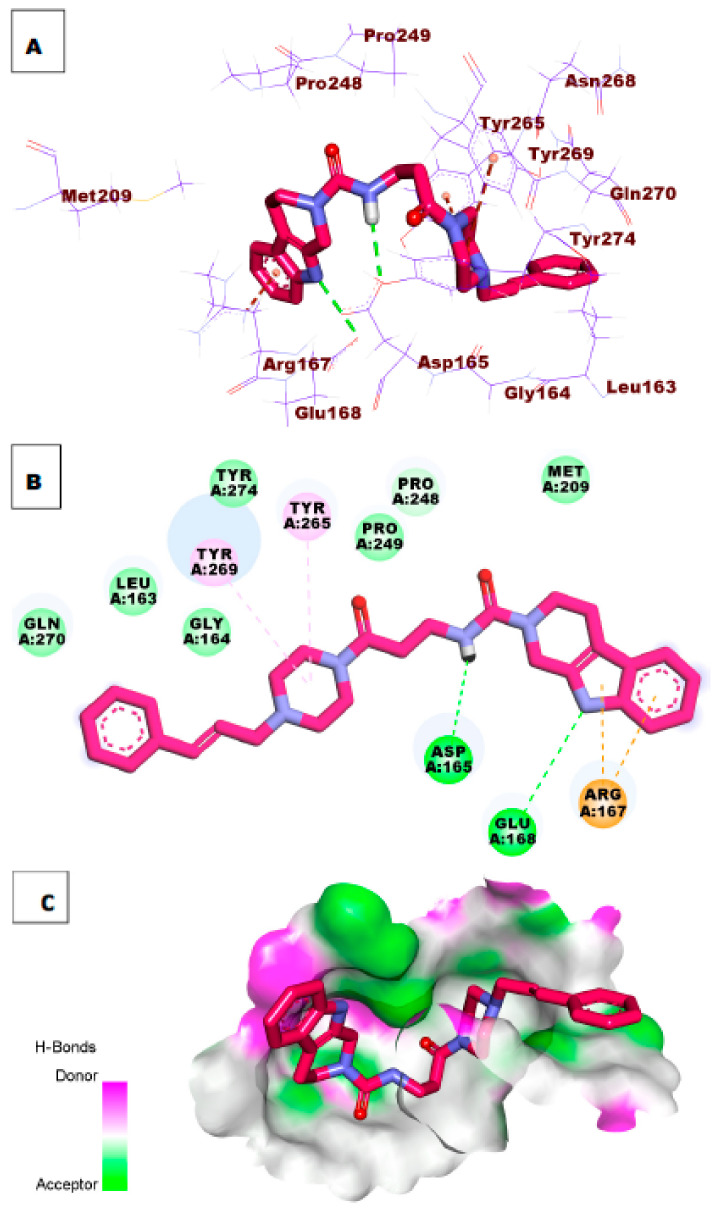
(**A**) 3D of compound **58** docked into the active site of PLpro. (**B**) 2D of compound **58** docked into the active site of PLpro. (**C**) Surface mapping of compound **58** docked into the active site of PLpro.

The binding mode of compound **58** (affinity value of −8.65 kcal/mol) was better than ligand **S88.** In detail, the amide moiety formed one hydrogen bond with fundamental amino acid Asp165, and NH of the pyrrole ring formed another hydrogen bond with Glu168. Furthermore, it formed aromatic stacking interactions (4 pi-cation bonds) with Tyr269, Tyr265, and Arg167 (Figure 10).

### 2.2. Pharmacokinetic Profiling Study

An *in silico* computational evaluation of the physicochemical properties and profiling pharmacokinetics for the most active eleven semi-synthesized molecules, with ligand **S88** as a reference compound, were conducted. The oral absorption of a drug is more likely to be better if the molecule fulfills at least three of the four principles of the Lipinski rules, listed below: (1) H bond donors (OH, NH, and SH) ≤ 5; (2) H bond acceptors (N, O, and S atoms) ≤ 10; (3) molecular weight < 500; (4) log P < 5. The bioavailability of compounds that violate more than one of these requirements is unlikely to be high. Moreover, reduced molecular flexibility, as measured by the number of rotatable bonds, and low polar surface area are found to be important predictors of good oral bioavailability [50]. Compounds with 10 or fewer rotatable bonds and a polar surface area of 140 Å or less have a high probability of good oral bioavailability [50,51].

The results, given in Table 2, revealed that all tested semi-synthesized molecules and reference ligand **S88** showed no violation of Lipinski’s rule except compound **31** (the Log P of compounds **31** was anticipated to be more than 5). 

### 2.3. ADMET Studies

**Figure 11:**
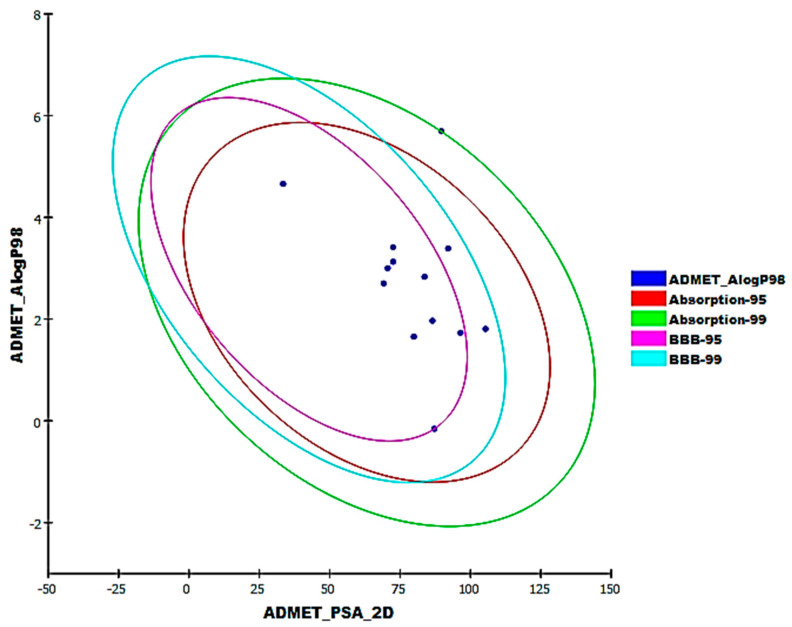
The expected ADMET study of the most potent semi-synthesized molecules.

**S88** and favipiravir were used as reference drugs in ADMET studies for the most active eleven semi-synthesized molecules using Discovery studio 4.0 software. ADMET studies include many descriptors. The predicted descriptors are listed in Table 3. All tested semi-synthesized molecules and favipiravir showed BBB penetration levels ranging from medium to low except compound **31**, which displayed a very low BBB penetration level, and ligand **S88** showed a high BBB penetration level. All semi-synthesized molecules, favipiravir, and ligand **S88** have good absorption behavior except compound **31**, which is expected to have a moderate absorption level. Moreover, the solubility level of the semi-synthesized molecules is projected to be better than or even comparable to that of the **S88**, which showed a low solubility level, except compound **31** that showed a very low solubility level. On the other hand, favipiravir demonstrated an optimal solubility level. All examined semi-synthesized molecules and favipiravir were predicted to be non-inhibitors of CYP2D6 except compounds **31**, **34**, **47**, and **S88**. Hepatotoxicity predictions found that all of the tested compounds and ligand **S88** are predicted to be non-toxic except compounds **17**, **31**, **41**, **43** and favipiravir, which have unfavorable hepatotoxic effects. All tested semi-synthesized molecules and **S88** were expected to bind to plasma proteins more than 90% except compounds **28**, **40**, **43**, **54**, and favipiravir (Figure 11).

### 2.4. Toxicity Studies

Discovery Studio 4.0 software was used to generate toxicity predictions for the most active eleven semi-synthesized molecules, which were based on validated and assembled models as follows: FDA rat carcinogenicity [52,53], carcinogenic potency TD_50_ [54], rat maximum tolerated dose (MTD) [55,56], rat oral LD_50_ [57], rat chronic LOAEL [58,59], ocular irritancy [60] and skin [19,60,61]. 

As shown in Table 4, most of the examined semi-synthesized molecules have low toxicity. All the tested semi-synthesized molecules are non-carcinogens except **54**, **58**, and **S88**, which were predicted to be carcinogens. All tested semi-synthesized molecules showed TD_50_ values ranging from 0.31 to 1.86 mg/kg body weight/day, which were lower than **S88** (2.54 mg/kg body weight/day), except compounds **17, 31**, **40**, and **47** that showed TD_50_ values of **5.57**, **5.32**, **10.31** and **14.54** mg/kg body weight/day, respectively, which were higher than **S88**. All the investigated semi-synthesized molecules revealed a maximum tolerated dose with a range of 0.045 to 0.122 g/kg body weight that was lower than **S88** (0.124 g/kg body weight) except compounds **16**, **28**, **31**, and **34**, which demonstrated maximum tolerated doses of 0.142, 0.144, 0.369 and 0.328 g/kg body weight, respectively, which were higher than **S88**. All tested semi-synthesized molecules showed oral LD_50_ values ranging from 2.97 to 32.39 mg/kg body weight/day that were higher than the LD_50_ value of **S88** (1.229 mg/kg body weight/day), except compound **31**, which revealed an oral LD_50_ value of 0.251 mg/kg body weight/day, which was lower than **S88**. Semi-synthesized molecules **54**, **58**, and **65** showed LOAEL values of 0.015, 0.001, and 0.018 g/kg body weight, respectively. These values were lower than **S88** (0.035 g/kg body weight), while other semi-synthesized molecules revealed rat chronic LOAEL values ranging from 0.039 to 0.539 g/kg body weight, which were higher than **S88**. Moreover, all the tested semi-synthesized molecules and **S88** were predicted to be mild irritants against the ocular irritancy model. On the other hand, the examined semi-synthesized molecules and **S88** were expected to be non-irritant against the skin irritancy model except compound **58**, which was anticipated to be a mild skin irritant.

### 2.5. DFT Studies 

DFT parameters, including total energy [62], HOMO [63], LUMO [63], gap energy [64], and dipole moment [65,66], were studied for the most semi-synthesized molecules **28, 34**, and **47** using Discovery studio 4.0 software. The co-crystallized S88 was used as a reference molecule.

#### 2.5.1. Molecular Orbital Analysis

The total energies of **28, 34, 47**, and **S88** were −1422.912, −1285.184, −1252.334, and 1242.947kcal/mol, respectively. These results indicated that compound **28** has higher total energy than **34** and **47** and is expected to have a more efficient interaction with PLpro. Accordingly, compound **28** can bind more efficiently with PLpro than **34** and **47**. Furthermore, the dipole moment values of **28, 34, 47**, and **S88** were 2.790, 1.558, 2.249, and 3.542, respectively (Table 5). These values indicate that **28** has a higher dipole moment than compounds **34** and **47**. Based on these findings, it was expected that compound **28** can easily form hydrogen bonds and non-bonded interactions with PLpro, which, consequently, leads to an increased binding affinity with the target receptor during SARS-CoV-2 inhibition. Therefore, compound **28** is considered the most promising candidate to interact with the target receptor.

**Figure 12:**
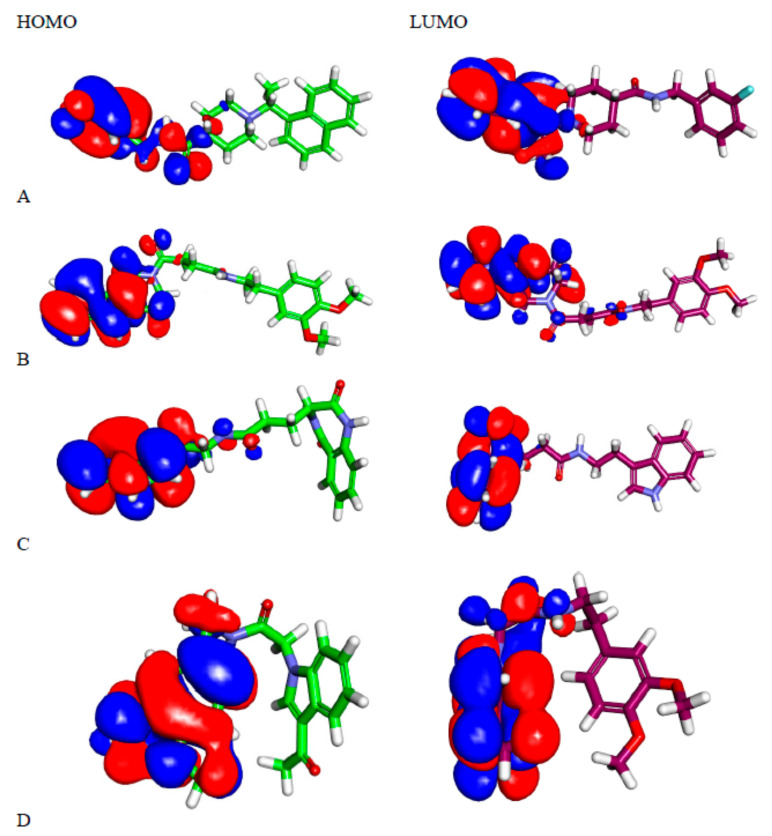
Spatial distribution of molecular orbitals for (**A**) **S88**, (**B**) **28**, and (**C**) **34**, and (**D**) **47**.

As reported, HOMO and LUMO have a key role in chemical stability and reactivity [67]. Compound **28** had a gap energy value of 0.134 kcal/mol, which is higher than that of compounds **34** (0.099 kcal/mol) and **47** (0.097kcal/mol). The increased gap energy of compound **28** indicates the higher stability of this compound. Figure 12 showed the spatial distribution of molecular orbitals for the tested compounds.

#### 2.5.2. Molecular Electrostatic Potential Maps (MEP)

MEP demonstrates the total electrostatic potential of a molecule in three dimensions depending on its partial charges, electronegativity, and chemical reactivity [68]. Identifying the electrostatic potential will help in the understanding of the drug’s binding mode against a PLpro [69].

MEP displays the electronegative atoms (negative values) in red. Electronegative atoms act as hydrogen bonding acceptors. On the other hand, it displays electron-poor atoms (positive value) in blue. Electron-poor atoms act as hydrogen bonding donors. It displays the neutral atoms (zero values) in a green to yellow color. Neutral atoms can form π- and other types of hydrophobic interactions. Such information facilitates the prediction of the chemical reaction and the binding mode with the biological target [70].

**Figure 13:**
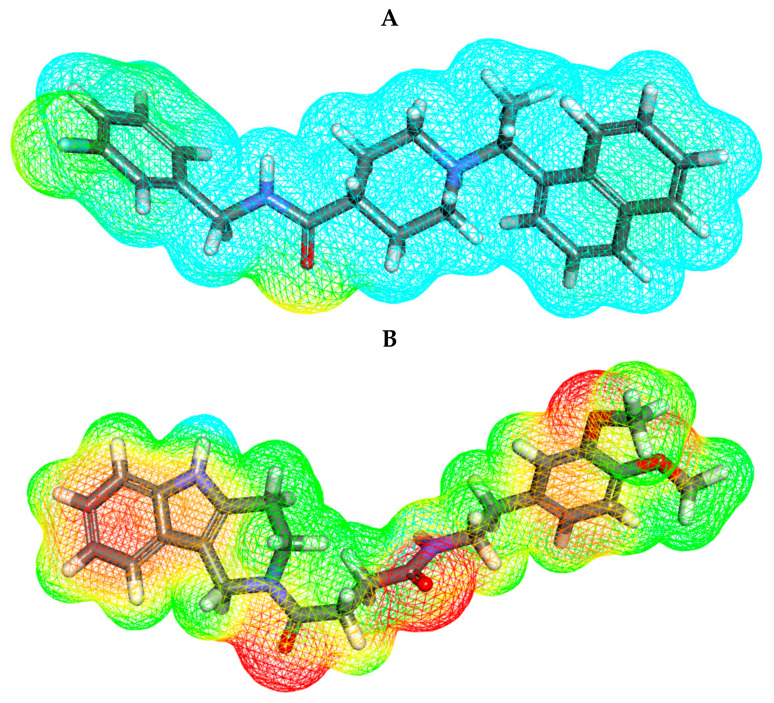

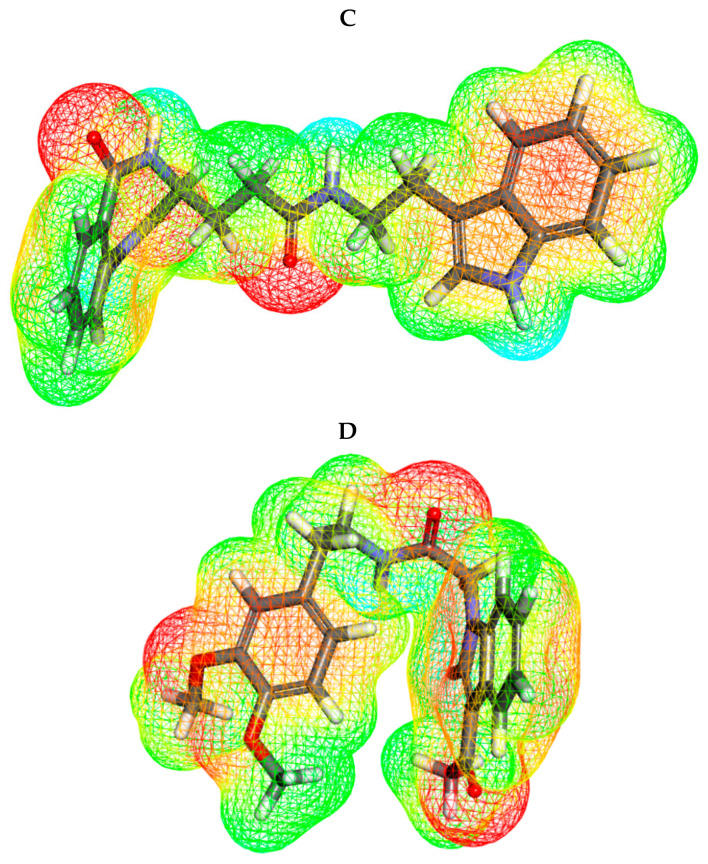
Molecular electrostatic potential map of (**A**) **S88**, (**B**) **28**, and (**C**) **34**, and (**D**) **47**.

Compound **28** showed five red patches and two blue patches, which can form hydrogen bond acceptors and hydrogen bond donors, respectively. The aromatic moieties showed yellow patches, which can form hydrophobic interactions with hydrophobic amino acid residues (Figure 12 and Figure 13).

Compounds **34** and **47** showed four red patches, which can form hydrogen bond acceptors. Compound **34** showed three red patches and two blue patches. The aromatic moieties of these compounds showed yellow patches which can form hydrophobic interactions with hydrophobic amino acid residues (Figure 12 and Figure 13).

As compound **28** showed five red patches, this explains its high binding energy (−8.48 kcal/mol) and ability to form two hydrogen bonds in the docking procedure. The yellow patches on aromatic moieties facilitated the hydrophobic interaction with the target receptor. Compounds **34** and **47** showed four red patches in MEP, which clarified the formation of two and three hydrogen bonds, respectively. In addition, these compounds showed high binding energies of −8.97 and −8.57 kcal/mol, respectively.

## 3. Conclusions

A set of 69 semi-synthesized molecules that exhibited the structural features of PLpro inhibitors (PLPI) were screened *in silico* to select the most potent inhibitor of PLpro enzyme (PDB ID: (4OW0). Docking studies showed that 11 molecules exhibited good binding modes and binding free energies. The pharmacokinetic profiling study excluded an unsuitable compound. Furthermore, ADMET and toxicity studies favored three molecules. Finally, a DFT study has been carried out and indicated that N-(3,4-dimethoxyphenethyl)-4-oxo-4-(1,3,4,5-tetrahydro-2H-pyrido[4,3-b]indol-2-yl)butanamide (**28**) is the most promising PLpro inhibitor. Further work has to be conducted to build on the presented results in the hopes of finding a cure.

## 4. Method

### 4.1. Docking Studies 

Crystal structure of human coronavirus papain-like protease inhibitor [PDB ID: 4OW0, resolution: 2.10 Å] was obtained from Protein Data Bank. The docking investigation was accomplished using MOE2014 software. At first, the crystal structure of SARS-CoV-2 helicase was prepared by removing water molecules. Only one chain was retained beside the co-crystallized ligand (S88). Then, the selected chain was protonated and subjected to minimization of energy process. Next, the active site of the target protein was defined.

Structures of the tested compounds and the co-crystallized ligand were drawn using ChemBioDraw Ultra 14.0 and saved as MDL-SD format. Such file was opened using MOE to display the 3D structures which were protonated and subjected to energy minimization. Formerly, validation of the docking process was performed by docking the co-crystallized ligand against the isolated pocket of active site. The produced RMSD value indicated the validity of process. Finally, docking of the tested compounds was done through the dock option inserted in computer window. For each docked molecule, 30 docked poses were produced using ASE for scoring function and force field for refinement. The retain was kept at 30. The crystal parameters were adjusted at default values (Coordinates: Normal, Lattice Style: the same, Lattice: (1)). The results of the docking process were then visualized using Discovery Studio 4.0 software [71]. 

### 4.2. Pharmacokinetic Profiling

Pharmacokinetic profile of the compounds was determined using Discovery studio 4.0. [72].

### 4.3. ADMET Analysis

ADMET descriptors (absorption, distribution, metabolism, excretion and toxicity) of the compounds were determined using Discovery studio 4.0. At first, the CHARMM force field was applied, then the tested compounds were prepared and minimized according to the preparation of small molecule protocol. Then ADMET descriptors protocol was applied to carry out these studies [73].

### 4.4. Toxicity Studies 

The toxicity parameters of the tested compounds were calculated using Discovery studio 4.0. Indinavir was used as a reference drug. At first, the CHARMM force field was applied then the compounds were prepared and minimized according to the preparation of small molecule protocol. Then different parameters were calculated from the toxicity prediction (extensible) protocol [73,74,75]. 

### 4.5. DFT Studies

The DFT parameters (total energy, binding energy, HOMO, LUMO, gap energy, dipole moment, and electrostatic potential) were calculated using Discovery studio software. The tested compounds were prepared using prepare ligand protocol. Then, the prepared compounds were subjected to DFT calculation protocol using the default option [76]. 

## Figures and Tables

**Table 1:** The calculated ΔG in Kcal/mol of the semi-synthesized molecules **1**–**69** and **S88** against PLpro.

Comp.	ΔG	Comp.	ΔG
**1**	−7.55	**36**	−7.49
**2**	−7.66	**37**	−7.47
**3**	−8.07	**38**	−8.04
**4**	−7.27	**39**	−7.92
**5**	−7.54	**40**	−8.54
**6**	−7.78	**41**	−8.39
**7**	−6.72	**42**	−8.22
**8**	−7.44	**43**	−8.53
**9**	−8.06	**44**	−7.79
**10**	−6.79	**45**	−7.47
**11**	−7.42	**46**	−7.67
**12**	−6.73	**47**	−8.57
**13**	−7.57	**48**	−7.22
**14**	−7.35	**49**	−7.28
**15**	−7.54	**50**	−8.13
**16**	−8.08	**51**	−8.27
**17**	−8.50	**52**	−7.59
**18**	−8.05	**53**	−7.63
**19**	−7.34	**54**	−8.33
**20**	−6.55	**55**	−7.60
**21**	−7.08	**56**	−7.10
**22**	−7.19	**57**	−8.34
**23**	−5.80	**58**	−8.65
**24**	−6.15	**59**	−8.01
**25**	−7.98	**60**	−8.22
**26**	−6.86	**61**	−7.58
**27**	−6.05	**62**	−7.98
**28**	−8.48	**63**	−7.50
**29**	−8.12	**64**	−8.07
**30**	−7.48	**65**	−8.33
**31**	−8.33	**66**	−8.15
**32**	−8.11	**67**	−7.86
**33**	−7.50	**68**	−8.20
**34**	−8.97	**69**	−8.18
**35**	−7.72	**ligand** **S88**	−8.59

**Table 2:** Physicochemical properties of the semi-synthesized molecules under study.

Compound	Lipinski’s Rule of 5					Veber’s Rule	
	Log P	Mole. Wt.	HBD	HBA	Violation	Number of Rotatable Bonds	TPSA
					of Lipinski’s Rule		
**17**	**3.38**	**441.47**	1	7	0	9	92.32
**28**	2.83	435.51	2	4	0	8	83.66
**31**	5.69	485.5	2	4	1	7	88.77
**34**	1.8	390.43	4	3	0	6	103.09
**40**	1.46	389.46	1	5	0	7	109
**41**	1.96	426.46	1	6	0	7	86.33
**43**	1.73	449.5	1	6	0	9	98.58
**47**	3	380.43	1	4	0	8	69.56
**54**	2.7	390.47	2	2	0	5	68.44
**58**	1.56	472.6	3	2	0	6	72.88
**65**	3.41	465.97	2	3	0	4	71.68
**S88**	3.09	391.5	2	1	0	5	33.54

**Table 3:** Predicted ADMET descriptors for the examined compounds, **S88**, and favipiravir.

Comp. No.	BBB Level ^1^	Absorption Level ^2^	Solubility	CYP2D6 ^4^	Hepatotoxicity Probability ^5^	PPB ^6^
			Level ^3^			
**17**	++	+++	++	−ve	0.549	2
**28**	++	+++	+++	−ve	0.37	0
**31**	+	++	+	+ve	0.629	2
**34**	++	+++	+++	+ve	0.47	1
**40**	++	+++	+++	−ve	0.437	0
**41**	++	+++	+++	−ve	0.821	2
**43**	++	+++	+++	−ve	0.622	0
**47**	+++	+++	++	+ve	0.456	2
**54**	+++	+++	+++	−ve	0.092	0
**58**	++	+++	+++	−ve	0.324	2
**65**	+++	+++	++	−ve	0.271	2
**S88**	++++	+++	++	+ve	0.092	1
Favipiravir	++	+++	++++	−ve	0.728	0

^1^ BBB level: ++++ = high, +++ = medium, ++ = low, + = very low. ^2^ Absorption level: +++ = good, ++ = moderate, + = poor. ^3^ solubility level: + = very low, ++ = low, +++ = good, ++++ = optimal. ^4^ CYP2D6(cytochrome P2D6); −ve = non inhibitor, +ve = inhibitor. ^5^ Hepatotoxicity probability: value > 0.5 means toxic, value < 0.5 means non-toxic. ^6^ PPB (plasma protein binding): 0 means less than 90%, 1 means more than 90%, 2 means more than 95%.

**Table 4:** Toxicity properties of tested compounds and **S88**.

Comp.	FDA Rat Carcinogenicity ^1^	Carcinogenic Potency TD_50_ (Rat) ^2^	Rat Maximum Tolerated Dose (Feed) ^3^	Rat Oral LD_50_^3^	Rat Chronic LOAEL ^3^	Ocular Irritancy	Skin Irritancy
**17**	−ve	5.577	0.142	4.097	0.039	Mild	None
**28**	−ve	1.199	0.144	27.190	0.539	Mild	None
**31**	−ve	5.323	0.369	0.251	0.043	Mild	None
**34**	−ve	0.826	0.328	32.393	0.232	Mild	None
**40**	−ve	10.310	0.033	14.820	0.339	Mild	None
**41**	−ve	1.162	0.122	26.756	0.446	Mild	None
**43**	−ve	1.860	0.100	26.741	0.151	Mild	None
**47**	−ve	14.544	0.045	8.150	0.139	Mild	None
**54**	+ve	0.324	0.068	5.277	0.015	Mild	None
**58**	+ve	1.725	0.071	5.145	0.001	Mild	Mild
**65**	−ve	0.315	0.117	2.971	0.018	Mild	None
**S88**	+ve	2.548	0.124	1.229	0.035	Mild	None

^1^ −ve = noncarcinogenic, +ve = carcinogenic. ^2^ Unit: mg/kg body weight/day. ^3^ Unit: g/kg body weight.

**Table 5:** Spatial distribution of molecular orbitals for candidates **28**, **34**, **47** and **S88**.

Name	Total Energy (kcal/mol)	Binding Energy (kcal/mol)	HOMO Energy (kcal/mol)	LUMO Energy (kcal/mol)	Dipole Mag	Band Gap Energy (kcal/mol)
**28**	−1422.912	−12.075	−0.170	−0.036	2.790	0.134
**34**	−1285.184	−10.458	−0.175	−0.076	1.558	0.099
**47**	−1252.334	−10.395	−0.172	−0.075	2.249	0.097
**S88**	−1242.947	−11.176	−0.292	−0.187	3.542	0.105

## Data Availability

Data is contained within the article.

## Supplementary Materials

The following are available online, Table S1: Detailed toxicity report, in addition to the method (Docking studies, ADMET studies, Toxicity studies and DFT studies).

## Sample Availability

Samples of the compounds are not available from the authors.
