# Supplementary material for: In Silico Screening of Semi-Synthesized Compounds as Potential Inhibitors for SARS-CoV-2 Papain-like Protease: Pharmacophoric Features, Molecular Docking, ADMET, Toxicity and DFT Studies
Source: Molecules. 2021 Oct 30;26(21):6593. doi: 10.3390/molecules26216593 (PMC8588135; doi:10.3390/molecules26216593)
Supplement: Supplementary file 1 [file molecules-26-06593-s001.zip › molecules-1399002-supplementary.pdf]

In silico screening of semi synthesized compounds as potential inhibitors for SARS-CoV-2 papain-like protease: Pharmacophoric features, molecular docking, ADMET, toxicity, and DFT studies

Mohamed S. Alesawy [a], Eslam B. Elkaeed[b], Aisha A. Alsfouk[c], Ahmed M. Metwaly[d]*, Ibrahim. H. Eissa[a]*

[a] Pharmaceutical Medicinal Chemistry & Drug Design Department, Faculty of Pharmacy (Boys), Al-Azhar University, Cairo11884, Egypt.

[b] Department of Pharmaceutical Sciences, College of Pharmacy, Almaarefa University, Ad Diriyah 13713, Riyadh, Saudi Arabia.

[c] Department of Pharmaceutical Sciences, College of Pharmacy, Princess Nourah bint Abdulrahman University, Riyadh, Saudi Arabia.

[d] Pharmacognosy and Medicinal Plants Department, Faculty of Pharmacy (Boys), Al-Azhar University, Cairo 11884, Egypt.

Content

Method	Docking studies
	ADMET studies
	Toxicity studies
	DFT studies
Toxicity report	

Method

1- Docking studies

The title molecules were investigated with the aid of docking studies using MOE software for their binding capabilities against Papain-Like Protease (PLpro). The crystal structures of the target protein was acquired from the RCSB Protein Data Bank (PDB ID: 4OW0), resolution: 2.10 Å [4]. the co-crystallized ligands **S88** was used as reference molecules. Molecular Operating Environment (MOE) was used for the docking analysis. In these studies, the free energies, and binding modes of the tested molecules against target proteins were determined. At first, the water molecules were removed from the crystal structures of target proteins, retaining only one chain which is essential for binding. The Co-crystallized ligands were used as reference ligands. Then, the protein structures wer protonated and the hydrogen atoms were hidden. Next, the energy was energy minimized by applying MMFF94x force field. After that, the binding pockets of each protein were defined. The structures of the examined compound and the co-crystallized ligands were drawn using ChemBioDraw Ultra 14.0 and saved as SDF formats. Then, the saved files were opened using MOE software and 3D structures were protonated. Next, the energy of the molecule was minimized applying MMFF94x force field. Validation processes were performed for each target receptor by running the docking process for only the co-crystallized ligand. low RMSD values between docked and crystal conformations indicate valid performance . The docking procedures were carried out utilizing a default protocol. In each case, 30 docked structures were generated using genetic algorithm searches. The output from of MOE software was further analyzed and visualized using Discovery Studio 4.0 software

2- ADMET studies

ADMET descriptors (absorption, distribution, metabolism, excretion and toxicity) of the compounds were determined using Discovery studio 4.0. At first, the CHARMM force field was applied then the tested compounds were prepared and minimized according to the preparation of small molecule protocol. Then ADMET descriptors protocol was applied to carry out these studies.

3- Toxicity studies

The toxicity parameters of the tested compounds were calculated using Discovery studio 4.0. Indinavir was used as a reference drug. At first, the CHARMM force field was applied then the compounds were prepared and minimized according to the preparation of small molecule protocol. Then different parameters were calculated from the toxicity prediction (extensible) protocol.

4- DFT studies:

The DFT parameters (total energy, binding energy, HOMO, LUMO, gap energy, dipole moment, and electrostatic potential) were calculated using Discovery studio software. the tested compounds were prepared using prepare ligand protocol. Then, the prepared compounds were subjected to DFT calculation protocol using the default option.

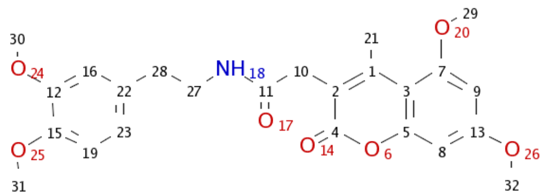

$C_{24}H_{27}NO_7$
Molecular Weight: 441.47368
ALogP: 3.388
Rotatable Bonds: 9
Acceptors: 7
Donors: 1

Model Prediction

Prediction: Non-Carcinogen
Probability: 0.230
Enrichment: 0.719
Bayesian Score: -8.302

Prediction: Positive if the Bayesian score is above the estimated best cutoff value.
Probability: The esimated probability that the sample is in the category.
Enrichment: An estimate of enrichment, that is, the increased likelihood (versus random) of this sample being in the category.
Bayesian Score: The standard Laplacian-modified Bayesian score.

Structural Similar Compounds

Name	Nabumetone	Atenolol	Glyburide
Structure	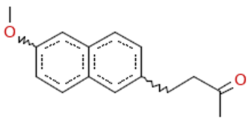	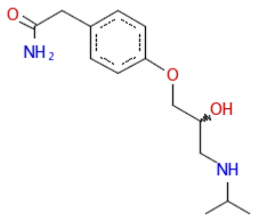	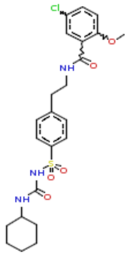
Actual Endpoint	Non-Carcinogen	Non-Carcinogen	Non-Carcinogen
Predicted Endpoint	Non-Carcinogen	Non-Carcinogen	Non-Carcinogen
Similarity	0.395	0.383	0.375
Reference	US FDA (Centre for Drug Eval.& Res./Off. Testing & Res.) Sept. 1997	US FDA (Centre for Drug Eval.& Res./Off. Testing & Res.) Sept. 1997	US FDA (Centre for Drug Eval.& Res./Off. Testing & Res.) Sept. 1997

Model Applicability

Missing features are fingerprint features in the query molecule not found in the training set.

1. Missing Feature: ECFP_6 1792159373 [*]C(=C(C)[c](:[*]):[*])[*]

Feature Contribution

Top features for Carcinogen

Fingerprint	Bit	Structure/Smiles	Score	Carcinogen in training set
ECFP_6	-770854792	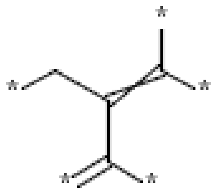	0.617	2 out of 2

ECFP_6	-464490300	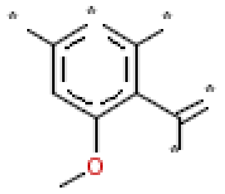	0.424	1 out of 1
ECFP_6	-1791034651	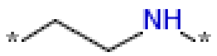	0.296	7 out of 16

Top Features for Non-Carcinogen

Fingerprint	Bit	Structure/Smiles	Score	Carcinogen in training set
ECFP_6	2077607946	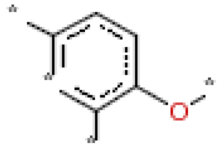	-1.152	0 out of 7
ECFP_6	-468366781	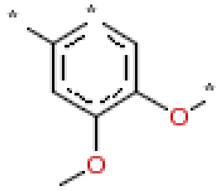	-0.805	0 out of 4
ECFP_6	1731843802	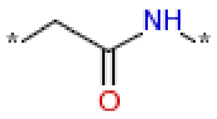	-0.657	0 out of 3

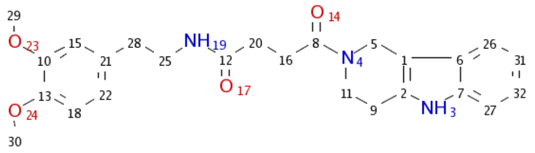

$C_{25}H_{29}N_3O_4$
Molecular Weight: 435.51546
ALogP: 2.831
Rotatable Bonds: 8
Acceptors: 4
Donors: 2

Model Prediction

Prediction: Non-Carcinogen
Probability: 0.243
Enrichment: 0.759
Bayesian Score: -9.274

Prediction: Positive if the Bayesian score is above the estimated best cutoff value.
Probability: The esimated probability that the sample is in the category.
Enrichment: An estimate of enrichment, that is, the increased likelihood (versus random) of this sample being in the category.
Bayesian Score: The standard Laplacian-modified Bayesian score.

Structural Similar Compounds

Name	Reserpine	Pergolide	Etodolac
Structure	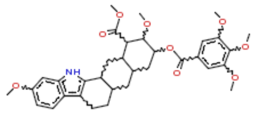	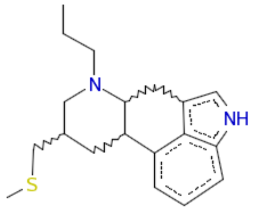	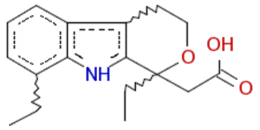
Actual Endpoint	Carcinogen	Carcinogen	Non-Carcinogen
Predicted Endpoint	Carcinogen	Carcinogen	Non-Carcinogen
Similarity	0.352	0.303	0.297
Reference	US FDA (Centre for Drug Eval.& Res./Off. Testing & Res.) Sept. 1997	US FDA (Centre for Drug Eval.& Res./Off. Testing & Res.) Sept. 1997	US FDA (Centre for Drug Eval.& Res./Off. Testing & Res.) Sept. 1997

Model Applicability

Missing features are fingerprint features in the query molecule not found in the training set.

1. Missing Feature: ECFP_6 979542842 [*]C[c]1:n:[*]:[*]:[c]:1[*]

Feature Contribution

Top features for Carcinogen				
**Fingerprint**	**Bit**	**Structure/Smiles**	**Score**	**Carcinogen in training set**
ECFP_6	51876938	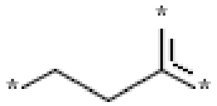	0.473	16 out of 31

ECFP_6	246515844	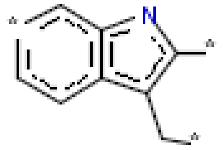	0.424	1 out of 1
ECFP_6	-1791034651	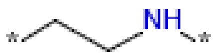	0.296	7 out of 16

Top Features for Non-Carcinogen

Fingerprint	Bit	Structure/Smiles	Score	Carcinogen in training set
ECFP_6	2077607946	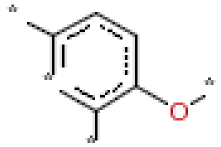	-1.152	0 out of 7
ECFP_6	-1102925512	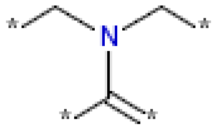	-0.805	0 out of 4
ECFP_6	-468366781	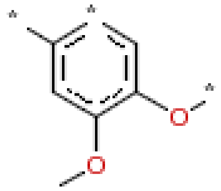	-0.805	0 out of 4

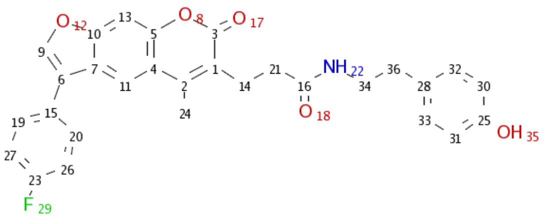

$C_{29}H_{24}FNO_5$
Molecular Weight: 485.50296
ALogP: 5.695
Rotatable Bonds: 7
Acceptors: 4
Donors: 2

Model Prediction

Prediction: Carcinogen
Probability: 0.252
Enrichment: 0.787
Bayesian Score: 0.506

Prediction: Positive if the Bayesian score is above the estimated best cutoff value.
Probability: The esimated probability that the sample is in the category.
Enrichment: An estimate of enrichment, that is, the increased likelihood (versus random) of this sample being in the category.
Bayesian Score: The standard Laplacian-modified Bayesian score.

Structural Similar Compounds

Name	Glyburide	Fluvastatin	Glimepride
Structure	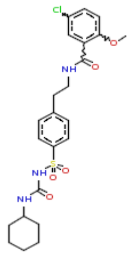	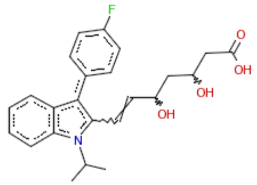	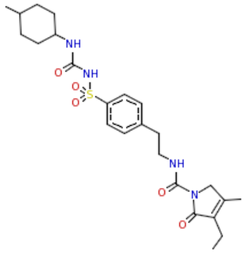
Actual Endpoint	Non-Carcinogen	Non-Carcinogen	Carcinogen
Predicted Endpoint	Non-Carcinogen	Non-Carcinogen	Carcinogen
Similarity	0.357	0.324	0.319
Reference	US FDA (Centre for Drug Eval.& Res./Off. Testing & Res.) Sept. 1997	US FDA (Centre for Drug Eval.& Res./Off. Testing & Res.) Sept. 1997	US FDA (Centre for Drug Eval.& Res./Off. Testing & Res.) Sept. 1997

Model Applicability

Missing features are fingerprint features in the query molecule not found in the training set.

1. Missing Feature: ECFP_6 1792159373 [*]C(=C(C)[c](:[*]):[*])[*]
2. Missing Feature: ECFP_6 -785659985 [*][c]1:[*]:[*]:o:c:1

Feature Contribution

Top features for Carcinogen				
**Fingerprint**	**Bit**	**Structure/Smiles**	**Score**	**Carcinogen in training set**
ECFP_6	1419645508	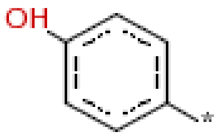	0.675	4 out of 5

ECFP_6	-770854792	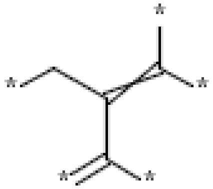	0.617	2 out of 2
ECFP_6	-790637051	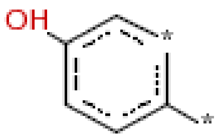	0.615	6 out of 9

Top Features for Non-Carcinogen

Fingerprint	Bit	Structure/Smiles	Score	Carcinogen in training set
ECFP_6	-219423964	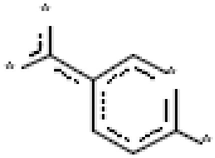	-0.935	0 out of 5
ECFP_6	1731843802	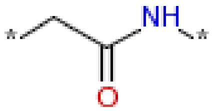	-0.657	0 out of 3
ECFP_6	-427397688	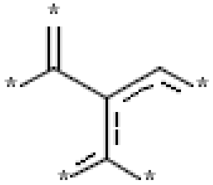	-0.476	5 out of 28

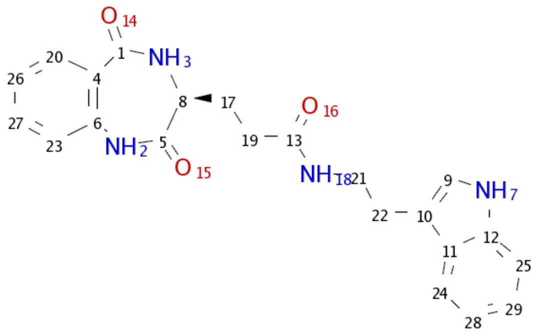

$C_{22}H_{22}N_4O_3$
Molecular Weight: 390.43508
ALogP: 1.807
Rotatable Bonds: 6
Acceptors: 3
Donors: 4

Model Prediction

Prediction: Non-Carcinogen
Probability: 0.213
Enrichment: 0.664
Bayesian Score: -6.012

Prediction: Positive if the Bayesian score is above the estimated best cutoff value.
Probability: The esimated probability that the sample is in the category.
Enrichment: An estimate of enrichment, that is, the increased likelihood (versus random) of this sample being in the category.
Bayesian Score: The standard Laplacian-modified Bayesian score.

Structural Similar Compounds

Name	Tryptophan	Prilocaine	Sumatriptan
Structure	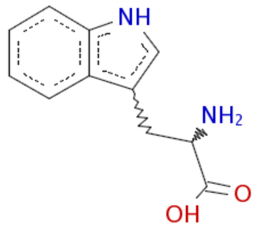	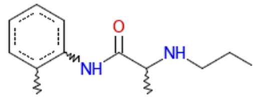	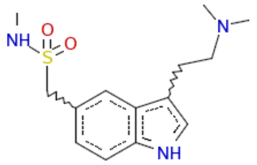
Actual Endpoint	Non-Carcinogen	Carcinogen	Non-Carcinogen
Predicted Endpoint	Non-Carcinogen	Carcinogen	Non-Carcinogen
Similarity	0.440	0.400	0.375
Reference	US FDA (Centre for Drug Eval.& Res./Off. Testing & Res.) Sept. 1997	US FDA (Centre for Drug Eval.& Res./Off. Testing & Res.) Sept. 1997	US FDA (Centre for Drug Eval.& Res./Off. Testing & Res.) Sept. 1997

Model Applicability

Missing features are fingerprint features in the query molecule not found in the training set.

1. All descriptors are in range.

Feature Contribution

Top features for Carcinogen

Fingerprint	Bit	Structure/Smiles	Score	Carcinogen in training set
ECFP_6	-953984246	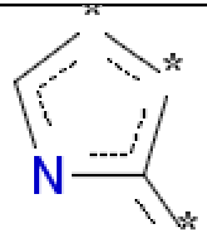	0.364	4 out of 8

ECFP_6	-1699286547	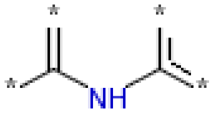	0.297	12 out of 28
ECFP_6	-1791034651	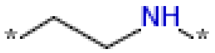	0.296	7 out of 16
**Top Features for Non-Carcinogen**				
**Fingerprint**	**Bit**	**Structure/Smiles**	**Score**	**Carcinogen in training set**
ECFP_6	-1659633832	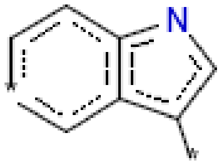	-0.657	0 out of 3
ECFP_6	1731843802	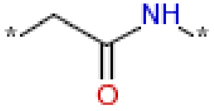	-0.657	0 out of 3
ECFP_6	-1613480181	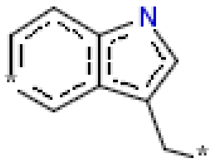	-0.482	0 out of 2

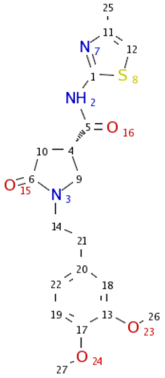

$C_{19}H_{23}N_3O_4S$
Molecular Weight: 389.46862
ALogP: 1.461
Rotatable Bonds: 7
Acceptors: 5
Donors: 1

Model Prediction

Prediction: Non-Carcinogen
Probability: 0.221
Enrichment: 0.691
Bayesian Score: -7.417

Prediction: Positive if the Bayesian score is above the estimated best cutoff value.
Probability: The esimated probability that the sample is in the category.
Enrichment: An estimate of enrichment, that is, the increased likelihood (versus random) of this sample being in the category.
Bayesian Score: The standard Laplacian-modified Bayesian score.

Structural Similar Compounds

Name	Sulfamethazine	Glipizide	Sulfadiazine
Structure	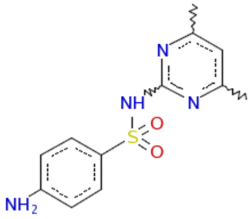	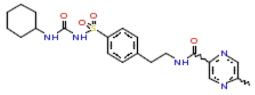	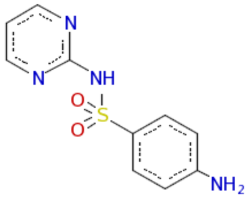
Actual Endpoint	Carcinogen	Non-Carcinogen	Non-Carcinogen
Predicted Endpoint	Carcinogen	Non-Carcinogen	Non-Carcinogen
Similarity	0.314	0.295	0.294
Reference	US FDA (Centre for Drug Eval.& Res./Off. Testing & Res.) Sept. 1997	US FDA (Centre for Drug Eval.& Res./Off. Testing & Res.) Sept. 1997	US FDA (Centre for Drug Eval.& Res./Off. Testing & Res.) Sept. 1997

Model Applicability

Missing features are fingerprint features in the query molecule not found in the training set.

1. Missing Feature: ECFP_6 -1596132236 [*]N[c]1:n:[*]:[*]:s:1
2. Missing Feature: ECFP_6 -656741159 [*]CN1C[*][*]C1=[*]
3. Missing Feature: ECFP_6 -857146788 [*]C(=[*])C1C[*][*]C1

Feature Contribution

Top features for Carcinogen

Fingerprint	Bit	Structure/Smiles	Score	Carcinogen in training set
ECFP_6	2106656448	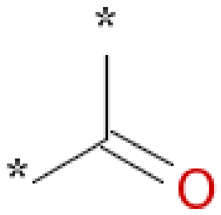	0.254	31 out of 77

ECFP_6	-1426923364	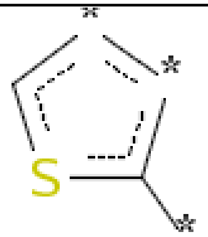	0.164	2 out of 5
ECFP_6	914325265	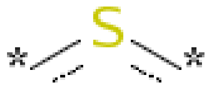	0.127	4 out of 11
**Top Features for Non-Carcinogen**				
**Fingerprint**	**Bit**	**Structure/Smiles**	**Score**	**Carcinogen in training set**
ECFP_6	2077607946	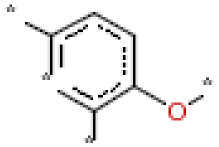	-1.152	0 out of 7
ECFP_6	-468366781	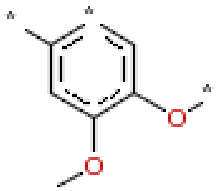	-0.805	0 out of 4
ECFP_6	1307307440	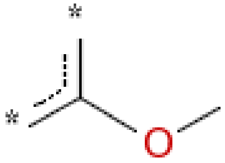	-0.558	4 out of 25

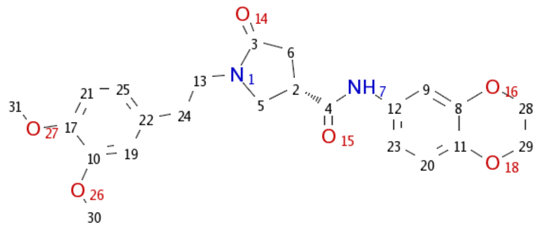

$C_{23}H_{26}N_2O_6$
Molecular Weight: 426.46234
ALogP: 1.967
Rotatable Bonds: 7
Acceptors: 6
Donors: 1

Model Prediction

Prediction: Non-Carcinogen
Probability: 0.238
Enrichment: 0.741
Bayesian Score: -8.876

Prediction: Positive if the Bayesian score is above the estimated best cutoff value.
Probability: The esimated probability that the sample is in the category.
Enrichment: An estimate of enrichment, that is, the increased likelihood (versus random) of this sample being in the category.
Bayesian Score: The standard Laplacian-modified Bayesian score.

Structural Similar Compounds

Name	Phenacetin	Nitroacetophenetide	Carteolol
Structure	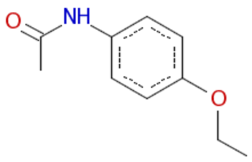	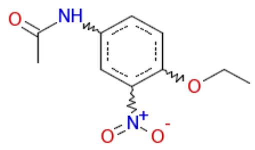	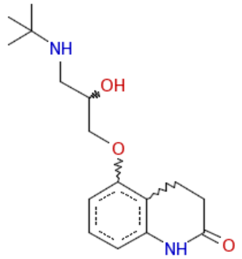
Actual Endpoint	Carcinogen	Non-Carcinogen	Non-Carcinogen
Predicted Endpoint	Carcinogen	Non-Carcinogen	Non-Carcinogen
Similarity	0.425	0.409	0.358
Reference	US FDA (Centre for Drug Eval.& Res./Off. Testing & Res.) Sept. 1997	US FDA (Centre for Drug Eval.& Res./Off. Testing & Res.) Sept. 1997	US FDA (Centre for Drug Eval.& Res./Off. Testing & Res.) Sept. 1997

Model Applicability

Missing features are fingerprint features in the query molecule not found in the training set.

1. Missing Feature: ECFP_6 -656741159 [*]CN1C[*][*]C1=[*]
2. Missing Feature: ECFP_6 -857146788 [*]C(=[*])C1C[*][*]C1

Feature Contribution

Top features for Carcinogen				
**Fingerprint**	**Bit**	**Structure/Smiles**	**Score**	**Carcinogen in training set**
ECFP_6	-91640731	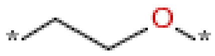	0.337	3 out of 6

ECFP_6	-177077903	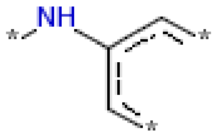	0.279	4 out of 9
ECFP_6	2106656448	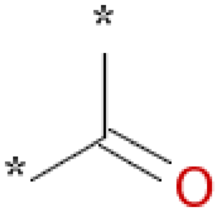	0.254	31 out of 77
**Top Features for Non-Carcinogen**				
**Fingerprint**	**Bit**	**Structure/Smiles**	**Score**	**Carcinogen in training set**
ECFP_6	2077607946	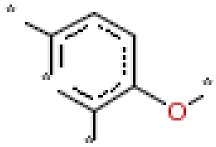	-1.152	0 out of 7
ECFP_6	-468366781	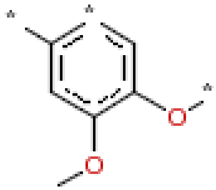	-0.805	0 out of 4
ECFP_6	1307307440	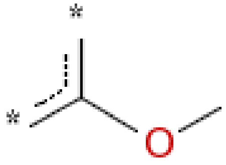	-0.558	4 out of 25

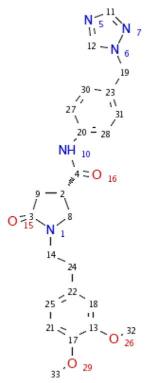

$C_{24}H_{27}N_5O_4$

Molecular Weight: 449.50228

ALogP: 1.731

Rotatable Bonds: 9

Acceptors: 6

Donors: 1

Model Prediction

Prediction: Non-Carcinogen

Probability: 0.253

Enrichment: 0.791

Bayesian Score: -12.418

Prediction: Positive if the Bayesian score is above the estimated best cutoff value.
Probability: The esimated probability that the sample is in the category.
Enrichment: An estimate of enrichment, that is, the increased likelihood (versus random) of this sample being in the category.
Bayesian Score: The standard Laplacian-modified Bayesian score.

Structural Similar Compounds

Name	Phenacetin	Nitroacetophenetide	Itraconazole
Structure	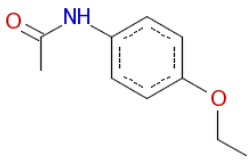	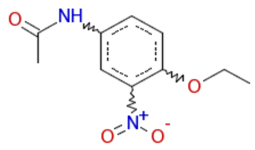	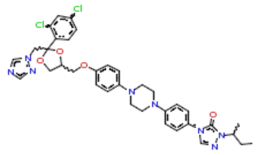
Actual Endpoint	Carcinogen	Non-Carcinogen	Non-Carcinogen
Predicted Endpoint	Carcinogen	Non-Carcinogen	Non-Carcinogen
Similarity	0.314	0.309	0.291
Reference	US FDA (Centre for Drug Eval.& Res./Off. Testing & Res.) Sept. 1997	US FDA (Centre for Drug Eval.& Res./Off. Testing & Res.) Sept. 1997	US FDA (Centre for Drug Eval.& Res./Off. Testing & Res.) Sept. 1997

Model Applicability

Missing features are fingerprint features in the query molecule not found in the training set.

1. Missing Feature: ECFP_6 -656741159 [*]CN1C[*][*]C1=[*]
2. Missing Feature: ECFP_6 -857146788 [*]C(=[*])C1C[*][*]C1

Feature Contribution

Top features for Carcinogen				
**Fingerprint**	**Bit**	**Structure/Smiles**	**Score**	**Carcinogen in training set**
ECFP_6	738938915	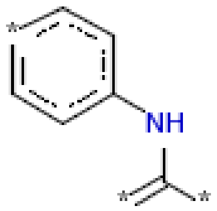	0.617	2 out of 2

ECFP_6	-177077903	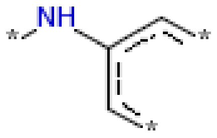	0.279	4 out of 9
ECFP_6	2106656448	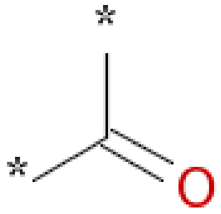	0.254	31 out of 77

Top Features for Non-Carcinogen

Fingerprint	Bit	Structure/Smiles	Score	Carcinogen in training set
ECFP_6	2077607946	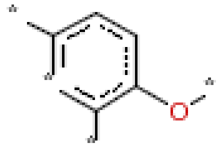	-1.152	0 out of 7
ECFP_6	-468366781	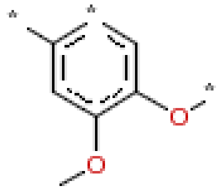	-0.805	0 out of 4
ECFP_6	1307307440	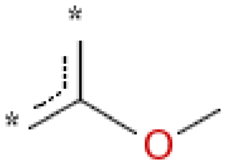	-0.558	4 out of 25

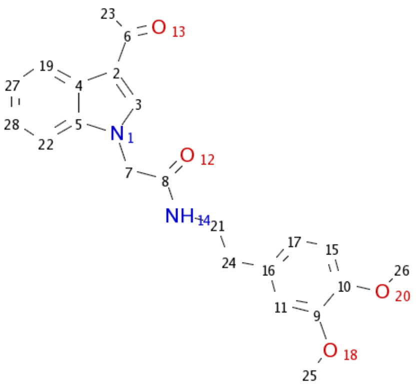

$C_{22}H_{24}N_2O_4$
Molecular Weight: 380.43696
ALogP: 3.001
Rotatable Bonds: 8
Acceptors: 4
Donors: 1

Model Prediction

Prediction: Non-Carcinogen
Probability: 0.248
Enrichment: 0.775
Bayesian Score: -13.316

Prediction: Positive if the Bayesian score is above the estimated best cutoff value.
Probability: The esimated probability that the sample is in the category.
Enrichment: An estimate of enrichment, that is, the increased likelihood (versus random) of this sample being in the category.
Bayesian Score: The standard Laplacian-modified Bayesian score.

Structural Similar Compounds

Name	Propafenone	Glyburide	Ondansetron
Structure	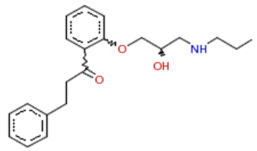	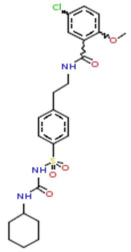	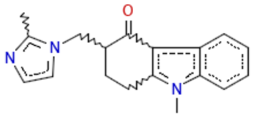
Actual Endpoint	Non-Carcinogen	Non-Carcinogen	Non-Carcinogen
Predicted Endpoint	Non-Carcinogen	Non-Carcinogen	Non-Carcinogen
Similarity	0.345	0.344	0.333
Reference	US FDA (Centre for Drug Eval.& Res./Off. Testing & Res.) Sept. 1997	US FDA (Centre for Drug Eval.& Res./Off. Testing & Res.) Sept. 1997	US FDA (Centre for Drug Eval.& Res./Off. Testing & Res.) Sept. 1997

Model Applicability

Missing features are fingerprint features in the query molecule not found in the training set.

1. Missing Feature: ECFP_6 499043293 [*]C(=[*])Cn(:[*]):[*]

Feature Contribution

Top features for Carcinogen

Fingerprint	Bit	Structure/Smiles	Score	Carcinogen in training set
ECFP_6	-1791034651	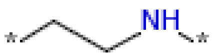	0.296	7 out of 16

ECFP_6	1527623780	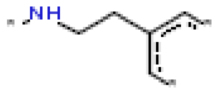	0.164	2 out of 5
ECFP_6	-182236392	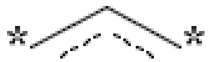	0.048	89 out of 274
**Top Features for Non-Carcinogen**				
**Fingerprint**	**Bit**	**Structure/Smiles**	**Score**	**Carcinogen in training set**
ECFP_6	2077607946	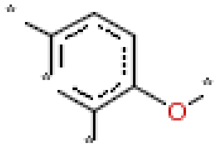	-1.152	0 out of 7
ECFP_6	1334415134	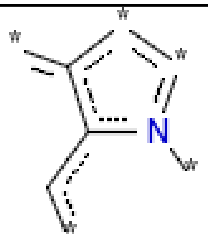	-0.935	0 out of 5
ECFP_6	-468366781	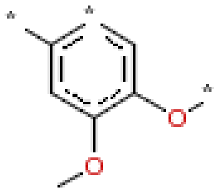	-0.805	0 out of 4

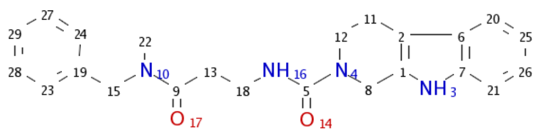

$C_{23}H_{26}N_4O_2$
Molecular Weight: 390.47814
ALogP: 2.702
Rotatable Bonds: 5
Acceptors: 2
Donors: 2

Model Prediction

Prediction: Non-Carcinogen
Probability: 0.214
Enrichment: 0.669
Bayesian Score: -2.945

Prediction: Positive if the Bayesian score is above the estimated best cutoff value.
Probability: The esimated probability that the sample is in the category.
Enrichment: An estimate of enrichment, that is, the increased likelihood (versus random) of this sample being in the category.
Bayesian Score: The standard Laplacian-modified Bayesian score.

Structural Similar Compounds

Name	Etodolac	Tryptophan	Glimepride
Structure	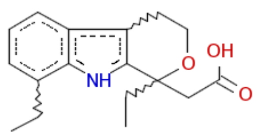	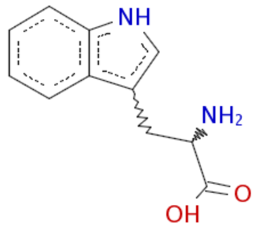	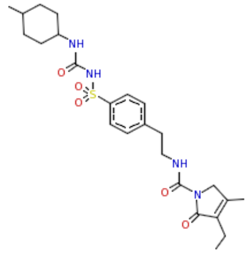
Actual Endpoint	Non-Carcinogen	Non-Carcinogen	Carcinogen
Predicted Endpoint	Non-Carcinogen	Non-Carcinogen	Carcinogen
Similarity	0.368	0.340	0.297
Reference	US FDA (Centre for Drug Eval.& Res./Off. Testing & Res.) Sept. 1997	US FDA (Centre for Drug Eval.& Res./Off. Testing & Res.) Sept. 1997	US FDA (Centre for Drug Eval.& Res./Off. Testing & Res.) Sept. 1997

Model Applicability

Missing features are fingerprint features in the query molecule not found in the training set.

1. Missing Feature: ECFP_6 979542842 [*]C[c]1:n:[*]:[*]:[c]:1[*]
2. Missing Feature: ECFP_6 -1022383729 [*]CN(C)C(=[*])[*]

Feature Contribution

Top features for Carcinogen				
**Fingerprint**	**Bit**	**Structure/Smiles**	**Score**	**Carcinogen in training set**
ECFP_6	51876938	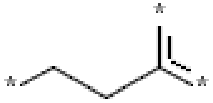	0.473	16 out of 31

ECFP_6	246515844	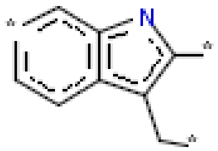	0.424	1 out of 1
ECFP_6	1657836083	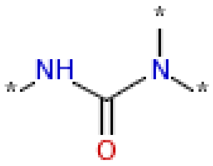	0.424	1 out of 1

Top Features for Non-Carcinogen

Fingerprint	Bit	Structure/Smiles	Score	Carcinogen in training set
ECFP_6	-1102925512	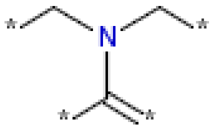	-0.805	0 out of 4
ECFP_6	-43933357	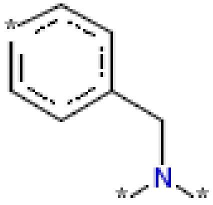	-0.657	0 out of 3
ECFP_6	-426213989	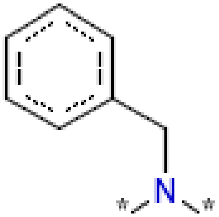	-0.482	0 out of 2

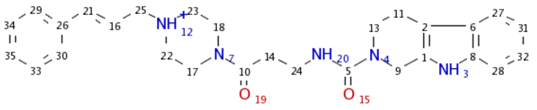

$C_{28}H_{34}N_5O_2$
Molecular Weight: 472.60186
ALogP: 1.567
Rotatable Bonds: 6
Acceptors: 2
Donors: 3

Model Prediction

Prediction: Carcinogen
Probability: 0.244
Enrichment: 0.760
Bayesian Score: -0.029

Prediction: Positive if the Bayesian score is above the estimated best cutoff value.
Probability: The esimated probability that the sample is in the category.
Enrichment: An estimate of enrichment, that is, the increased likelihood (versus random) of this sample being in the category.
Bayesian Score: The standard Laplacian-modified Bayesian score.

Structural Similar Compounds

Name	Etodolac	Tryptophan	Bromocriptine
Structure	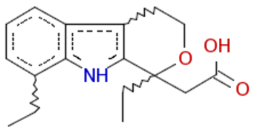	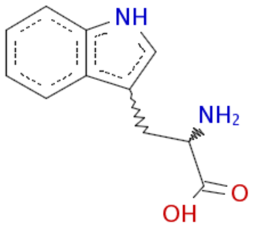	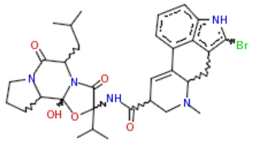
Actual Endpoint	Non-Carcinogen	Non-Carcinogen	Non-Carcinogen
Predicted Endpoint	Non-Carcinogen	Non-Carcinogen	Non-Carcinogen
Similarity	0.333	0.286	0.272
Reference	US FDA (Centre for Drug Eval.& Res./Off. Testing & Res.) Sept. 1997	US FDA (Centre for Drug Eval.& Res./Off. Testing & Res.) Sept. 1997	US FDA (Centre for Drug Eval.& Res./Off. Testing & Res.) Sept. 1997

Model Applicability

Missing features are fingerprint features in the query molecule not found in the training set.

1. Missing Feature: ECFP_6 1976330679 [*][NH+]([*])[*]
2. Missing Feature: ECFP_6 979542842 [*]C[c]1:n:[*]:[*]:[c]:1[*]
3. Missing Feature: ECFP_6 1133499173 [*]C[NH+](C[*])C[*]
4. Missing Feature: ECFP_6 -244159614 [*]CC[NH+]([*])[*]
5. Missing Feature: ECFP_6 474483418 [*][NH+]([*])CC=[*]
6. Missing Feature: ECFP_6 -176483725 [*]=C[c](:c:[*]):c:[*]

Feature Contribution

Top features for Carcinogen

Fingerprint	Bit	Structure/Smiles	Score	Carcinogen in training set
ECFP_6	51876938	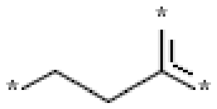	0.473	16 out of 31

ECFP_6	-97048906	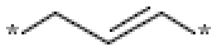	0.442	2 out of 3
ECFP_6	477797263	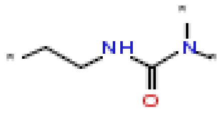	0.424	1 out of 1

Top Features for Non-Carcinogen

Fingerprint	Bit	Structure/Smiles	Score	Carcinogen in training set
ECFP_6	-1831055759	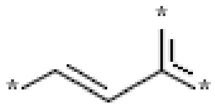	-0.805	0 out of 4
ECFP_6	-1102925512	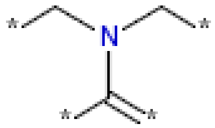	-0.805	0 out of 4
ECFP_6	-1789102870	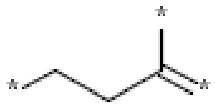	-0.384	2 out of 11

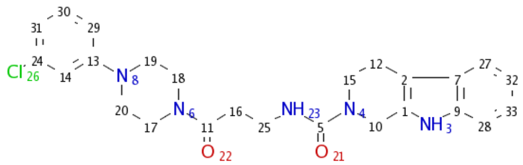

$C_{25}H_{28}ClN_5O_2$
Molecular Weight: 465.97512
ALogP: 3.413
Rotatable Bonds: 4
Acceptors: 3
Donors: 2

Model Prediction

Prediction: Non-Carcinogen
Probability: 0.264
Enrichment: 0.823
Bayesian Score: -10.443

Prediction: Positive if the Bayesian score is above the estimated best cutoff value.
Probability: The esimated probability that the sample is in the category.
Enrichment: An estimate of enrichment, that is, the increased likelihood (versus random) of this sample being in the category.
Bayesian Score: The standard Laplacian-modified Bayesian score.

Structural Similar Compounds

Name	Temazepam	Diazepam	Ketoconazole
Structure	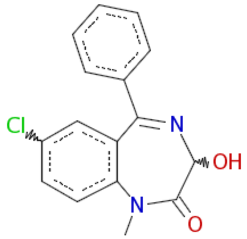	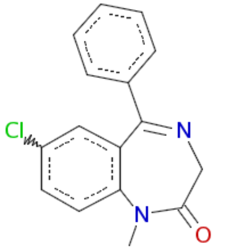	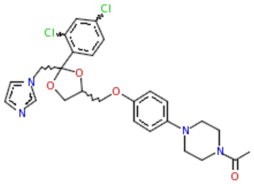
Actual Endpoint	Non-Carcinogen	Carcinogen	Non-Carcinogen
Predicted Endpoint	Non-Carcinogen	Non-Carcinogen	Non-Carcinogen
Similarity	0.339	0.328	0.312
Reference	US FDA (Centre for Drug Eval.& Res./Off. Testing & Res.) Sept. 1997	US FDA (Centre for Drug Eval.& Res./Off. Testing & Res.) Sept. 1997	US FDA (Centre for Drug Eval.& Res./Off. Testing & Res.) Sept. 1997

Model Applicability

Missing features are fingerprint features in the query molecule not found in the training set.

1. Missing Feature: ECFP_6 979542842 [*]C[c]1:n:[*]:[*]:[c]:1[*]

Feature Contribution

Top features for Carcinogen				
Fingerprint	Bit	Structure/Smiles	Score	Carcinogen in training set
ECFP_6	51876938	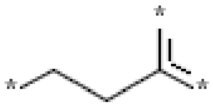	0.473	16 out of 31

ECFP_6	246515844	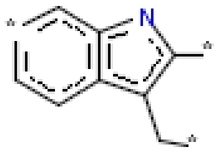	0.424	1 out of 1
ECFP_6	477797263	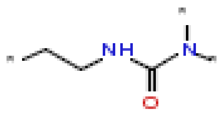	0.424	1 out of 1

Top Features for Non-Carcinogen

**Fingerprint**	**Bit**	**Structure/Smiles**	**Score**	**Carcinogen in training set**
ECFP_6	342267039	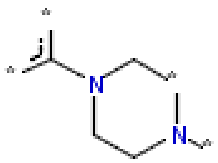	-1.152	0 out of 7
ECFP_6	-175021654	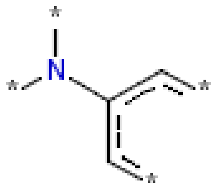	-0.805	0 out of 4
ECFP_6	-1102925512	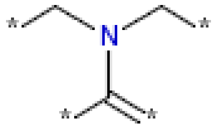	-0.805	0 out of 4

MOST ACTIVE.mol

TOPKAT_Mouse_Female_FDA_None_vs_Carcinogen

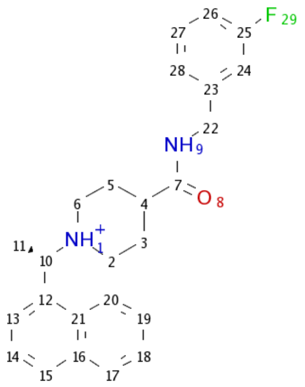

$C_{25}H_{28}FN_2O$
Molecular Weight: 391.50102
ALogP: 3.098
Rotatable Bonds: 5
Acceptors: 1
Donors: 2

Model Prediction

Prediction: Carcinogen
Probability: 0.248
Enrichment: 0.775
Bayesian Score: 0.272

Prediction: Positive if the Bayesian score is above the estimated best cutoff value.
Probability: The esimated probability that the sample is in the category.
Enrichment: An estimate of enrichment, that is, the increased likelihood (versus random) of this sample being in the category.
Bayesian Score: The standard Laplacian-modified Bayesian score.

Structural Similar Compounds

Name	Bupropion	Chlorpropamide	Phenobarbital
Structure	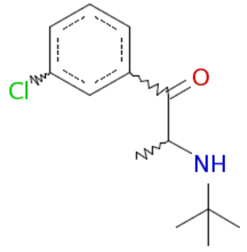	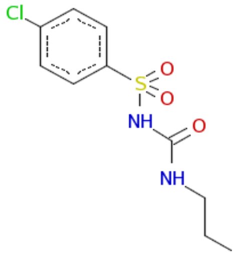	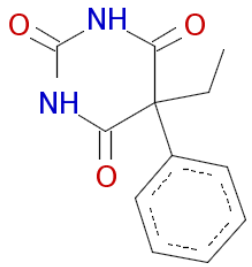
Actual Endpoint	Non-Carcinogen	Non-Carcinogen	Carcinogen
Predicted Endpoint	Non-Carcinogen	Non-Carcinogen	Carcinogen
Similarity	0.362	0.327	0.311
Reference	US FDA (Centre for Drug Eval.& Res./Off. Testing & Res.) Sept. 1997	US FDA (Centre for Drug Eval.& Res./Off. Testing & Res.) Sept. 1997	US FDA (Centre for Drug Eval.& Res./Off. Testing & Res.) Sept. 1997

Model Applicability

Missing features are fingerprint features in the query molecule not found in the training set.

1. Missing Feature: ECFP_6 1976330679 [*][NH+]([*])[*]
2. Missing Feature: ECFP_6 2070272975 [*]C[NH+](C[*])C([*])[*]
3. Missing Feature: ECFP_6 -244159614 [*]CC[NH+]([*])[*]
4. Missing Feature: ECFP_6 -857146788 [*]C(=[*])C1C[*][*]C1
5. Missing Feature: ECFP_6 -1670638661 [*][NH+]([*])[C@H](C)[c](:[*]):[*]

Feature Contribution

Top features for Carcinogen

Fingerprint	Bit	Structure/Smiles	Score	Carcinogen in training set
ECFP_6	769925792	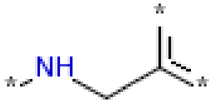	0.617	2 out of 2

ECFP_6	-81428579	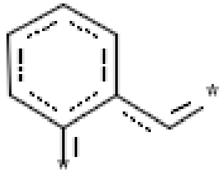	0.581	3 out of 4
ECFP_6	-178525456	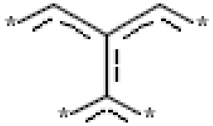	0.457	4 out of 7

Top Features for Non-Carcinogen

Fingerprint	Bit	Structure/Smiles	Score	Carcinogen in training set
ECFP_6	2007300961	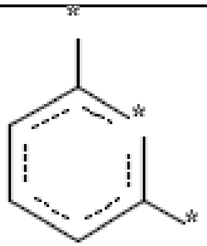	-0.652	5 out of 34
ECFP_6	-81134287	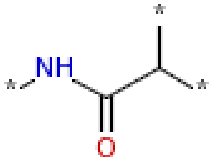	-0.482	0 out of 2
ECFP_6	-1910270391	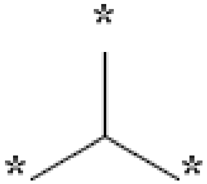	-0.307	20 out of 89

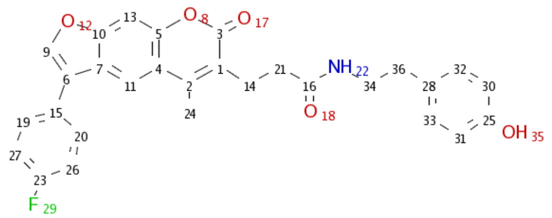

$C_{29}H_{24}FNO_5$

Molecular Weight: 485.50296

ALogP: 5.695

Rotatable Bonds: 7

Acceptors: 4

Donors: 2

Model Prediction

Prediction: Multiple-Carcinogen

Probability: 0.628

Enrichment: 1.531

Bayesian Score: 4.779

Prediction: Positive if the Bayesian score is above the estimated best cutoff value.
Probability: The esimated probability that the sample is in the category.
Enrichment: An estimate of enrichment, that is, the increased likelihood (versus random) of this sample being in the category.
Bayesian Score: The standard Laplacian-modified Bayesian score.

Structural Similar Compounds

Name	Glimepride	Salmeterol	Labetalol
Structure	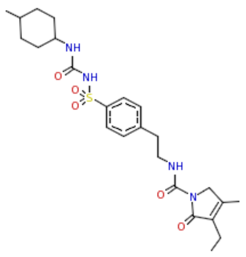	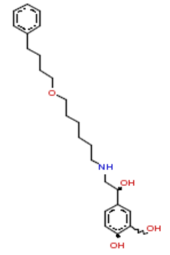	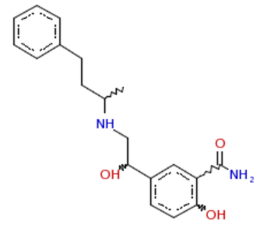
Actual Endpoint	Single-Carcinogen	Single-Carcinogen	Single-Carcinogen
Predicted Endpoint	Single-Carcinogen	Single-Carcinogen	Single-Carcinogen
Similarity	0.319	0.271	0.261
Reference	US FDA (Centre for Drug Eval.& Res./Off. Testing & Res.) Sept. 1997	US FDA (Centre for Drug Eval.& Res./Off. Testing & Res.) Sept. 1997	US FDA (Centre for Drug Eval.& Res./Off. Testing & Res.) Sept. 1997

Model Applicability

Missing features are fingerprint features in the query molecule not found in the training set.

1. Missing Feature: ECFP_4 1792159373 [*]C(=C(C)[c](:[*]):[*])[*]
2. Missing Feature: ECFP_4 -785659985 [*][c]1:[*]:[*]:o:c:1
3. Missing Feature: ECFP_4 1731843802 [*]CC(=O)N[*]

Feature Contribution

Top features for Multiple-Carcinogen

Fingerprint	Bit	Structure/Smiles	Score	Multiple-Carcinogen in training set
ECFP_4	-97599322	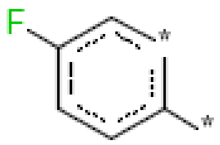	0.641	4 out of 4

ECFP_4	-176686665	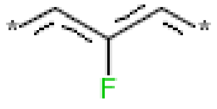	0.641	4 out of 4
ECFP_4	-296909061	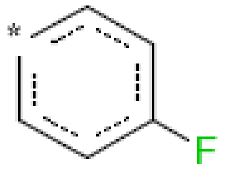	0.586	3 out of 3

Top Features for Single-Carcinogen

**Fingerprint**	**Bit**	**Structure/Smiles**	**Score**	**Multiple-Carcinogen in training set**
ECFP_4	497523368	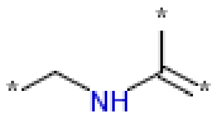	-0.968	0 out of 4
ECFP_4	1205550831	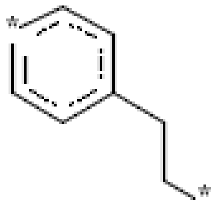	-0.545	1 out of 6
ECFP_4	-1897341097	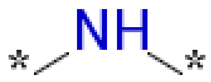	-0.356	8 out of 29

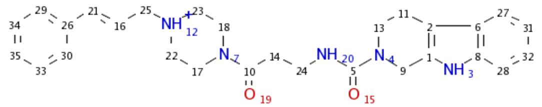

$C_{28}H_{34}N_5O_2$
Molecular Weight: 472.60186
ALogP: 1.567
Rotatable Bonds: 6
Acceptors: 2
Donors: 3

Model Prediction

Prediction: Single-Carcinogen
Probability: 0.279
Enrichment: 0.680
Bayesian Score: -4.530

Prediction: Positive if the Bayesian score is above the estimated best cutoff value.
Probability: The esimated probability that the sample is in the category.
Enrichment: An estimate of enrichment, that is, the increased likelihood (versus random) of this sample being in the category.
Bayesian Score: The standard Laplacian-modified Bayesian score.

Structural Similar Compounds

Name	Methylphenidate	Glimepride	Pergolide
Structure	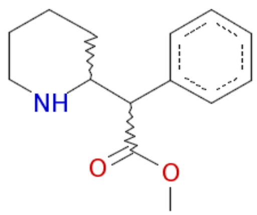	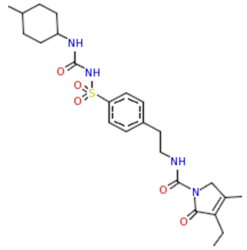	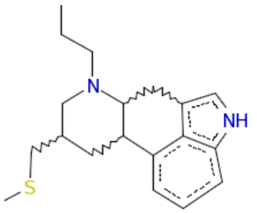
Actual Endpoint	Single-Carcinogen	Single-Carcinogen	Single-Carcinogen
Predicted Endpoint	Single-Carcinogen	Single-Carcinogen	Single-Carcinogen
Similarity	0.258	0.257	0.247
Reference	US FDA (Centre for Drug Eval.& Res./Off. Testing & Res.) Sept. 1997	US FDA (Centre for Drug Eval.& Res./Off. Testing & Res.) Sept. 1997	US FDA (Centre for Drug Eval.& Res./Off. Testing & Res.) Sept. 1997

Model Applicability

Missing features are fingerprint features in the query molecule not found in the training set.

1. Missing Feature: ECFP_4 1976330679 [*][NH+]([*])[*]
2. Missing Feature: ECFP_4 979542842 [*]C[c]1:n:[*]:[*]:[c]:1[*]
3. Missing Feature: ECFP_4 -1102925512 [*]CN(C[*])C(=[*])[*]
4. Missing Feature: ECFP_4 1133499173 [*]C[NH+](C[*])C[*]
5. Missing Feature: ECFP_4 -1831055759 [*]\C=C\[c](:[*]):[*]
6. Missing Feature: ECFP_4 -244159614 [*]CC[NH+]([*])[*]
7. Missing Feature: ECFP_4 474483418 [*][NH+]([*])CC=[*]
8. Missing Feature: ECFP_4 -176483725 [*]=C[c](:c:[*]):c:[*]

Feature Contribution

Top features for Multiple-Carcinogen

Fingerprint	Bit	Structure/Smiles	Score	Multiple-Carcinogen in training set

ECFP_4	246515844	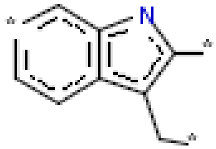	0.351	1 out of 1
ECFP_4	1571214559	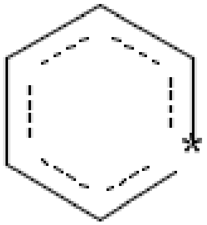	0.283	9 out of 16
ECFP_4	-757679000	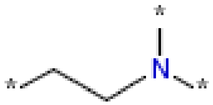	0.242	6 out of 11
**Top Features for Single-Carcinogen**				
**Fingerprint**	**Bit**	**Structure/Smiles**	**Score**	**Multiple-Carcinogen in training set**
ECFP_4	497523368	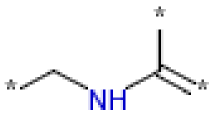	-0.968	0 out of 4
ECFP_4	-1925046727	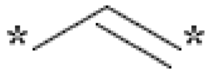	-0.605	2 out of 11

ECFP_4	1639858918	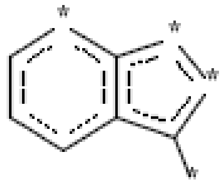	-0.597	0 out of 2

MOST ACTIVE.mol

TOPKAT_Mouse_Female_FDA_Single_vs_Multiple

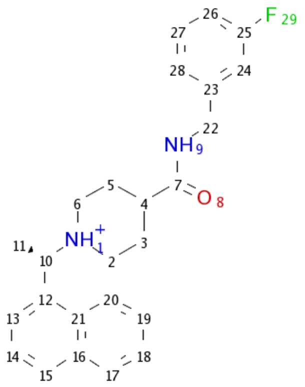

$C_{25}H_{28}FN_2O$

Molecular Weight: 391.50102

ALogP: 3.098

Rotatable Bonds: 5

Acceptors: 1

Donors: 2

Structural Similar Compounds

Name	Phenobarbital	Sertraline	Diazepam
Structure	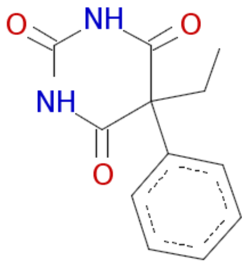	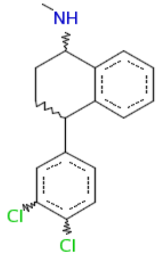	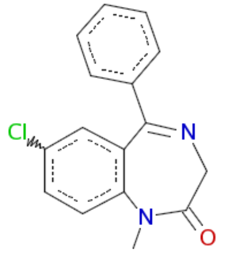
Actual Endpoint	Multiple-Carcinogen	Single-Carcinogen	Multiple-Carcinogen
Predicted Endpoint	Multiple-Carcinogen	Single-Carcinogen	Multiple-Carcinogen
Similarity	0.311	0.306	0.302
Reference	US FDA (Centre for Drug Eval.& Res./Off. Testing & Res.) Sept. 1997	US FDA (Centre for Drug Eval.& Res./Off. Testing & Res.) Sept. 1997	US FDA (Centre for Drug Eval.& Res./Off. Testing & Res.) Sept. 1997

Model Prediction

Prediction: Multiple-Carcinogen

Probability: 0.356

Enrichment: 0.869

Bayesian Score: -1.198

Prediction: Positive if the Bayesian score is above the estimated best cutoff value.
Probability: The esimated probability that the sample is in the category.
Enrichment: An estimate of enrichment, that is, the increased likelihood (versus random) of this sample being in the category.
Bayesian Score: The standard Laplacian-modified Bayesian score.

Model Applicability

Missing features are fingerprint features in the query molecule not found in the training set.

1. Missing Feature: ECFP_4 1976330679 [*][NH+]([*])[*]
2. Missing Feature: ECFP_4 2070272975 [*]C[NH+](C[*])C([*])[*]
3. Missing Feature: ECFP_4 -244159614 [*]CC[NH+]([*])[*]
4. Missing Feature: ECFP_4 -857146788 [*]CC(C[*])C(=[*])[*]
5. Missing Feature: ECFP_4 -81134287 [*]NC(=O)C([*])[*]
6. Missing Feature: ECFP_4 -1670638661 [*][NH+]([*])[C@H](C)[c](:[*]):[*]
7. Missing Feature: ECFP_4 1335340087 [*]C([*])[c](:c:[*]):[c](:[*]):[*]

Feature Contribution

Top features for Multiple-Carcinogen

Fingerprint	Bit	Structure/Smiles	Score	Multiple-Carcinogen in training set

ECFP_4	-176686665	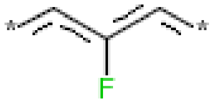	0.641	4 out of 4
ECFP_4	220735655	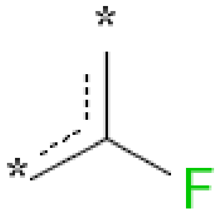	0.497	4 out of 5
ECFP_4	-1560646374	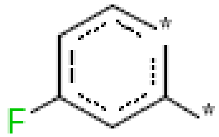	0.351	1 out of 1

Top Features for Single-Carcinogen

Fingerprint	Bit	Structure/Smiles	Score	Multiple-Carcinogen in training set
ECFP_4	-178525456	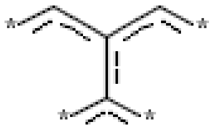	-0.968	0 out of 4
ECFP_4	497523368	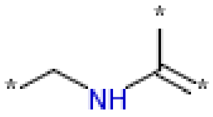	-0.968	0 out of 4

ECFP_4	717474525	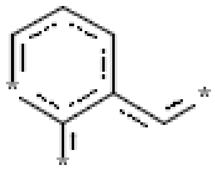	-0.800	0 out of 3

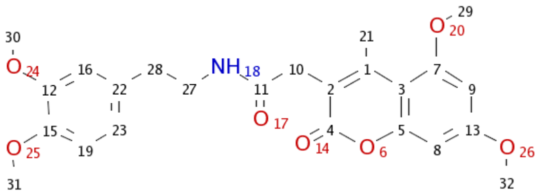

$C_{24}H_{27}NO_7$
Molecular Weight: 441.47368
ALogP: 3.388
Rotatable Bonds: 9
Acceptors: 7
Donors: 1

Model Prediction

Prediction: Non-Carcinogen
Probability: 0.193
Enrichment: 0.655
Bayesian Score: -5.286

Prediction: Positive if the Bayesian score is above the estimated best cutoff value.
Probability: The esimated probability that the sample is in the category.
Enrichment: An estimate of enrichment, that is, the increased likelihood (versus random) of this sample being in the category.
Bayesian Score: The standard Laplacian-modified Bayesian score.

Structural Similar Compounds

Name	Nabumetone	Atenolol	Glyburide
Structure	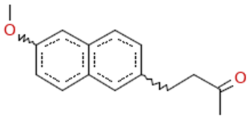	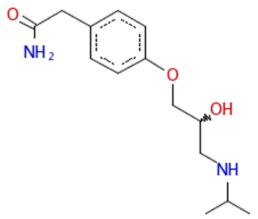	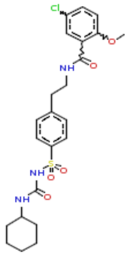
Actual Endpoint	Non-Carcinogen	Non-Carcinogen	Non-Carcinogen
Predicted Endpoint	Non-Carcinogen	Non-Carcinogen	Non-Carcinogen
Similarity	0.395	0.383	0.375
Reference	US FDA (Centre for Drug Eval.& Res./Off. Testing & Res.) Sept. 1997	US FDA (Centre for Drug Eval.& Res./Off. Testing & Res.) Sept. 1997	US FDA (Centre for Drug Eval.& Res./Off. Testing & Res.) Sept. 1997

Model Applicability

Missing features are fingerprint features in the query molecule not found in the training set.

1. All descriptors are in range.

Feature Contribution

Top features for Carcinogen

Fingerprint	Bit	Structure/Smiles	Score	Carcinogen in training set
SCFP_6	55523958	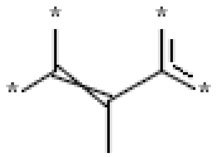	0.511	2 out of 3

SCFP_6	-392286499	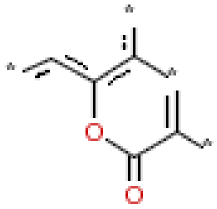	0.457	1 out of 1
SCFP_6	1157879834	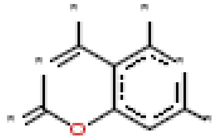	0.266	1 out of 2
**Top Features for Non-Carcinogen**				
**Fingerprint**	**Bit**	**Structure/Smiles**	**Score**	**Carcinogen in training set**
SCFP_6	-1211866396	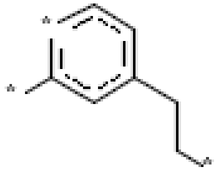	-1.233	1 out of 22
SCFP_6	-1642341584	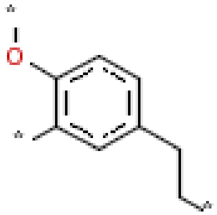	-0.847	0 out of 5
SCFP_6	-1850586302	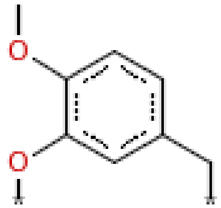	-0.587	0 out of 3

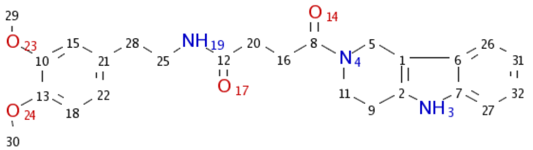

$C_{25}H_{29}N_3O_4$
Molecular Weight: 435.51546
ALogP: 2.831
Rotatable Bonds: 8
Acceptors: 4
Donors: 2

Model Prediction

Prediction: Non-Carcinogen
Probability: 0.173
Enrichment: 0.586
Bayesian Score: -8.458

Prediction: Positive if the Bayesian score is above the estimated best cutoff value.
Probability: The esimated probability that the sample is in the category.
Enrichment: An estimate of enrichment, that is, the increased likelihood (versus random) of this sample being in the category.
Bayesian Score: The standard Laplacian-modified Bayesian score.

Structural Similar Compounds

Name	Reserpine	Pergolide	Etodolac
Structure	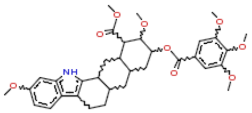	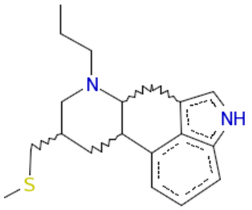	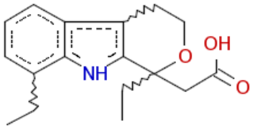
Actual Endpoint	Carcinogen	Non-Carcinogen	Non-Carcinogen
Predicted Endpoint	Carcinogen	Non-Carcinogen	Non-Carcinogen
Similarity	0.352	0.303	0.297
Reference	US FDA (Centre for Drug Eval.& Res./Off. Testing & Res.) Sept. 1997	US FDA (Centre for Drug Eval.& Res./Off. Testing & Res.) Sept. 1997	US FDA (Centre for Drug Eval.& Res./Off. Testing & Res.) Sept. 1997

Model Applicability

Missing features are fingerprint features in the query molecule not found in the training set.

1. All descriptors are in range.

Feature Contribution

Top features for Carcinogen

Fingerprint	Bit	Structure/Smiles	Score	Carcinogen in training set
SCFP_6	1651620003	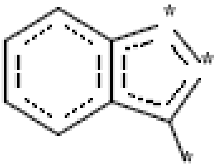	0.468	4 out of 8

SCFP_6	-1272768868	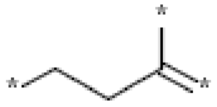	0.402	20 out of 49
SCFP_6	-704249936	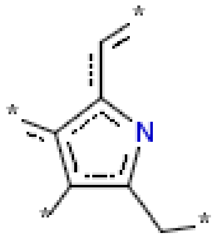	0.373	2 out of 4
**Top Features for Non-Carcinogen**				
**Fingerprint**	**Bit**	**Structure/Smiles**	**Score**	**Carcinogen in training set**
SCFP_6	-1211866396	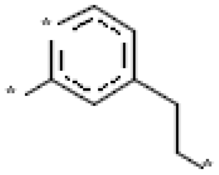	-1.233	1 out of 22
SCFP_6	-1343150366	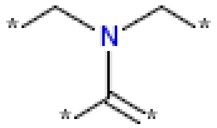	-1.017	1 out of 17
SCFP_6	-1642341584	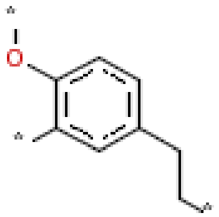	-0.847	0 out of 5

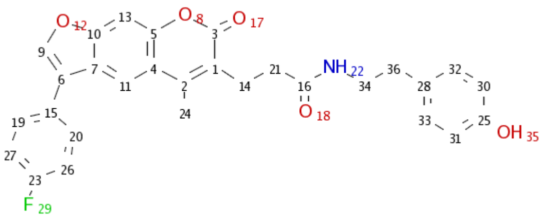

$C_{29}H_{24}FNO_5$
Molecular Weight: 485.50296
ALogP: 5.695
Rotatable Bonds: 7
Acceptors: 4
Donors: 2

Model Prediction

Prediction: Non-Carcinogen
Probability: 0.208
Enrichment: 0.706
Bayesian Score: -3.871

Prediction: Positive if the Bayesian score is above the estimated best cutoff value.
Probability: The esimated probability that the sample is in the category.
Enrichment: An estimate of enrichment, that is, the increased likelihood (versus random) of this sample being in the category.
Bayesian Score: The standard Laplacian-modified Bayesian score.

Structural Similar Compounds

Name	Glyburide	Fluvastatin	Glimepride
Structure	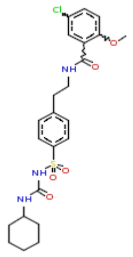	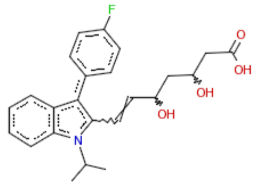	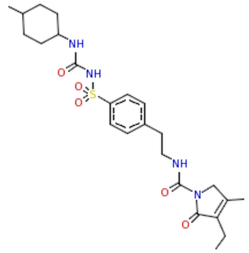
Actual Endpoint	Non-Carcinogen	Non-Carcinogen	Carcinogen
Predicted Endpoint	Non-Carcinogen	Non-Carcinogen	Carcinogen
Similarity	0.357	0.324	0.319
Reference	US FDA (Centre for Drug Eval.& Res./Off. Testing & Res.) Sept. 1997	US FDA (Centre for Drug Eval.& Res./Off. Testing & Res.) Sept. 1997	US FDA (Centre for Drug Eval.& Res./Off. Testing & Res.) Sept. 1997

Model Applicability

Missing features are fingerprint features in the query molecule not found in the training set.

1. All descriptors are in range.

Feature Contribution

Top features for Carcinogen				
**Fingerprint**	**Bit**	**Structure/Smiles**	**Score**	**Carcinogen in training set**
SCFP_6	1958008606	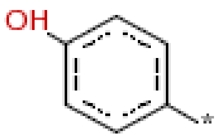	0.661	3 out of 4

SCFP_6	794417578	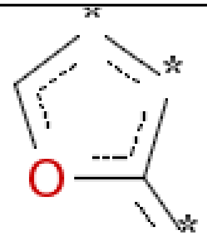	0.569	5 out of 9
SCFP_6	55523958	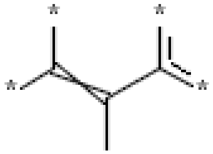	0.511	2 out of 3
**Top Features for Non-Carcinogen**				
**Fingerprint**	**Bit**	**Structure/Smiles**	**Score**	**Carcinogen in training set**
SCFP_6	-1211866396	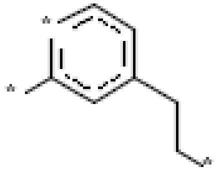	-1.233	1 out of 22
SCFP_6	-1642341584	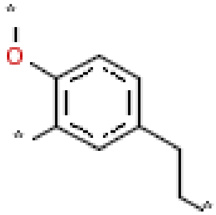	-0.847	0 out of 5
SCFP_6	1689050571	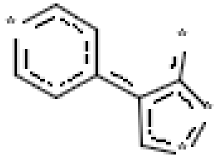	-0.725	0 out of 4

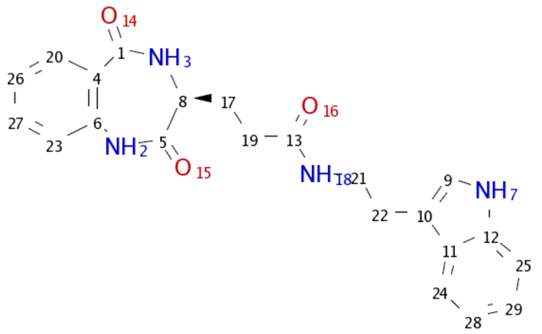

$C_{22}H_{22}N_4O_3$
Molecular Weight: 390.43508
ALogP: 1.807
Rotatable Bonds: 6
Acceptors: 3
Donors: 4

Model Prediction

Prediction: Non-Carcinogen
Probability: 0.208
Enrichment: 0.707
Bayesian Score: -3.852

Prediction: Positive if the Bayesian score is above the estimated best cutoff value.
Probability: The esimated probability that the sample is in the category.
Enrichment: An estimate of enrichment, that is, the increased likelihood (versus random) of this sample being in the category.
Bayesian Score: The standard Laplacian-modified Bayesian score.

Structural Similar Compounds

Name	Tryptophan	Prilocaine	Sumatriptan
Structure	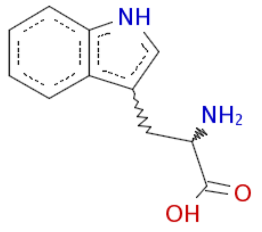	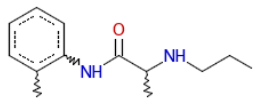	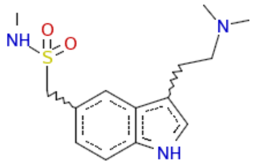
Actual Endpoint	Non-Carcinogen	Carcinogen	Non-Carcinogen
Predicted Endpoint	Non-Carcinogen	Carcinogen	Non-Carcinogen
Similarity	0.440	0.400	0.375
Reference	US FDA (Centre for Drug Eval.& Res./Off. Testing & Res.) Sept. 1997	US FDA (Centre for Drug Eval.& Res./Off. Testing & Res.) Sept. 1997	US FDA (Centre for Drug Eval.& Res./Off. Testing & Res.) Sept. 1997

Model Applicability

Missing features are fingerprint features in the query molecule not found in the training set.

1. All descriptors are in range.

Feature Contribution

Top features for Carcinogen

Fingerprint	Bit	Structure/Smiles	Score	Carcinogen in training set
SCFP_6	2097618059	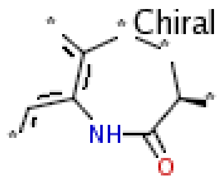	0.711	7 out of 11

SCFP_6	1631845520	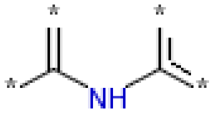	0.501	9 out of 19
SCFP_6	1651620003	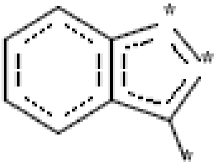	0.468	4 out of 8
**Top Features for Non-Carcinogen**				
**Fingerprint**	**Bit**	**Structure/Smiles**	**Score**	**Carcinogen in training set**
SCFP_6	-673674794	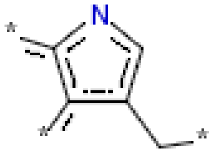	-0.847	0 out of 5
SCFP_6	1188101983	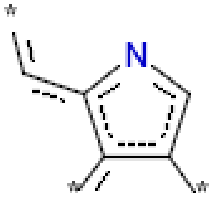	-0.847	0 out of 5
SCFP_6	-1946889102	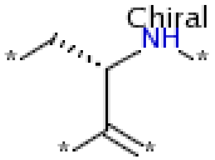	-0.741	1 out of 12

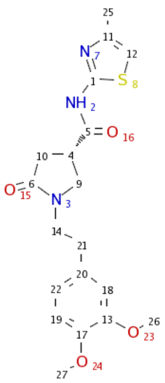

$C_{19}H_{23}N_{3}O_{4}S$
Molecular Weight: 389.46862
ALogP: 1.461
Rotatable Bonds: 7
Acceptors: 5
Donors: 1

Model Prediction

Prediction: Non-Carcinogen
Probability: 0.214
Enrichment: 0.726
Bayesian Score: -3.407

Prediction: Positive if the Bayesian score is above the estimated best cutoff value.
Probability: The esimated probability that the sample is in the category.
Enrichment: An estimate of enrichment, that is, the increased likelihood (versus random) of this sample being in the category.
Bayesian Score: The standard Laplacian-modified Bayesian score.

Structural Similar Compounds

Name	Sulfamethazine	Furothiazole	Glipizide
Structure	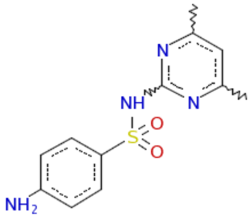	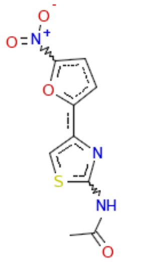	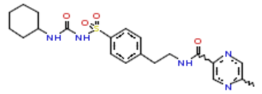
Actual Endpoint	Carcinogen	Carcinogen	Non-Carcinogen
Predicted Endpoint	Carcinogen	Carcinogen	Non-Carcinogen
Similarity	0.314	0.302	0.295
Reference	US FDA (Centre for Drug Eval.& Res./Off. Testing & Res.) Sept. 1997	US FDA (Centre for Drug Eval.& Res./Off. Testing & Res.) Sept. 1997	US FDA (Centre for Drug Eval.& Res./Off. Testing & Res.) Sept. 1997

Model Applicability

Missing features are fingerprint features in the query molecule not found in the training set.

1. All descriptors are in range.

Feature Contribution

Top features for Carcinogen				
Fingerprint	Bit	Structure/Smiles	Score	Carcinogen in training set
SCFP_6	-100019659	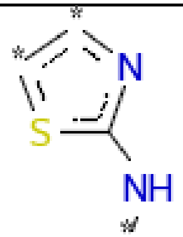	0.672	2 out of 2

SCFP_6	-1043310069	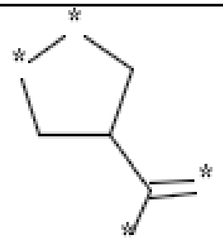	0.504	13 out of 28
SCFP_6	1631845520	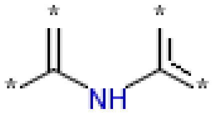	0.501	9 out of 19

Top Features for Non-Carcinogen

**Fingerprint**	**Bit**	**Structure/Smiles**	**Score**	**Carcinogen in training set**
SCFP_6	-1211866396	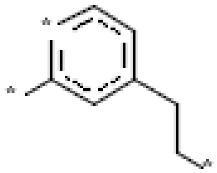	-1.233	1 out of 22
SCFP_6	-1343150366	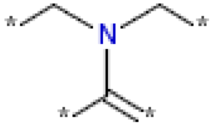	-1.017	1 out of 17
SCFP_6	-1642341584	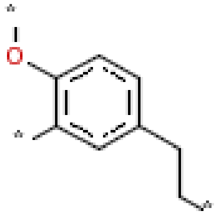	-0.847	0 out of 5

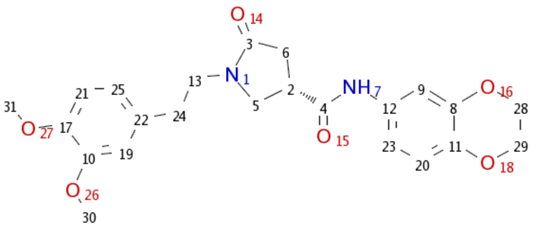

$C_{23}H_{26}N_2O_6$
Molecular Weight: 426.46234
ALogP: 1.967
Rotatable Bonds: 7
Acceptors: 6
Donors: 1

Model Prediction

Prediction: Non-Carcinogen
Probability: 0.202
Enrichment: 0.685
Bayesian Score: -4.416

Prediction: Positive if the Bayesian score is above the estimated best cutoff value.
Probability: The esimated probability that the sample is in the category.
Enrichment: An estimate of enrichment, that is, the increased likelihood (versus random) of this sample being in the category.
Bayesian Score: The standard Laplacian-modified Bayesian score.

Structural Similar Compounds

Name	Phenacetin	Nitroacetophenetide	Carteolol
Structure	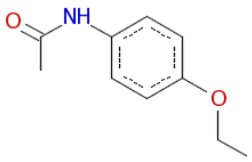	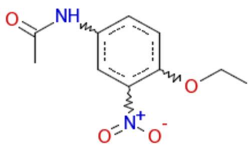	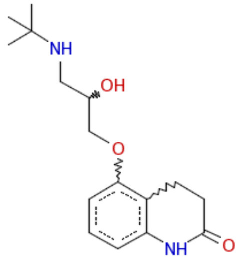
Actual Endpoint	Carcinogen	Carcinogen	Non-Carcinogen
Predicted Endpoint	Carcinogen	Carcinogen	Non-Carcinogen
Similarity	0.425	0.409	0.358
Reference	US FDA (Centre for Drug Eval.& Res./Off. Testing & Res.) Sept. 1997	US FDA (Centre for Drug Eval.& Res./Off. Testing & Res.) Sept. 1997	US FDA (Centre for Drug Eval.& Res./Off. Testing & Res.) Sept. 1997

Model Applicability

Missing features are fingerprint features in the query molecule not found in the training set.

1. All descriptors are in range.

Feature Contribution

Top features for Carcinogen

Fingerprint	Bit	Structure/Smiles	Score	Carcinogen in training set
SCFP_6	2097618059	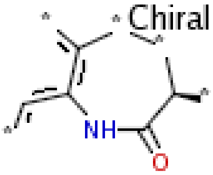	0.711	7 out of 11

SCFP_6	-531283893	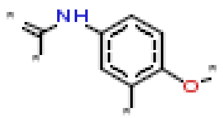	0.661	3 out of 4
SCFP_6	-347048986	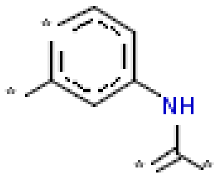	0.655	4 out of 6

Top Features for Non-Carcinogen

Fingerprint	Bit	Structure/Smiles	Score	Carcinogen in training set
SCFP_6	-1211866396	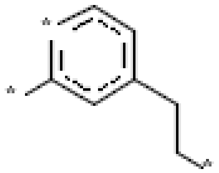	-1.233	1 out of 22
SCFP_6	-1343150366	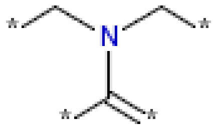	-1.017	1 out of 17
SCFP_6	-1642341584	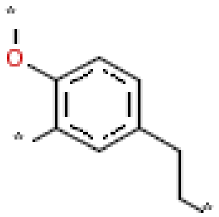	-0.847	0 out of 5

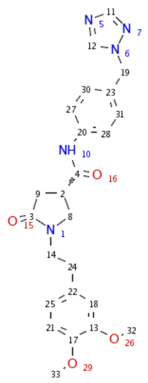

$C_{24}H_{27}N_5O_4$
Molecular Weight: 449.50228
ALogP: 1.731
Rotatable Bonds: 9
Acceptors: 6
Donors: 1

Model Prediction

Prediction: Non-Carcinogen

Probability: 0.182

Enrichment: 0.618

Bayesian Score: -6.696

Prediction: Positive if the Bayesian score is above the estimated best cutoff value.
Probability: The esimated probability that the sample is in the category.
Enrichment: An estimate of enrichment, that is, the increased likelihood (versus random) of this sample being in the category.
Bayesian Score: The standard Laplacian-modified Bayesian score.

Structural Similar Compounds

Name	Phenacetin	Nitroacetophenetide	Itraconazole
Structure	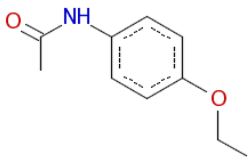	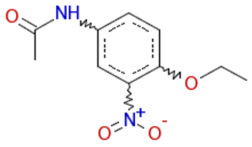	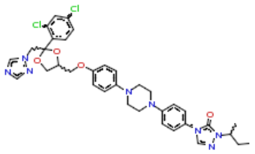
Actual Endpoint	Carcinogen	Carcinogen	Non-Carcinogen
Predicted Endpoint	Carcinogen	Carcinogen	Non-Carcinogen
Similarity	0.314	0.309	0.291
Reference	US FDA (Centre for Drug Eval.& Res./Off. Testing & Res.) Sept. 1997	US FDA (Centre for Drug Eval.& Res./Off. Testing & Res.) Sept. 1997	US FDA (Centre for Drug Eval.& Res./Off. Testing & Res.) Sept. 1997

Model Applicability

Missing features are fingerprint features in the query molecule not found in the training set.

1. All descriptors are in range.

Feature Contribution

Top features for Carcinogen

Fingerprint	Bit	Structure/Smiles	Score	Carcinogen in training set
SCFP_6	2097618059	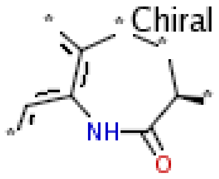	0.711	7 out of 11

SCFP_6	814408713	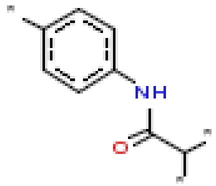	0.672	2 out of 2
SCFP_6	-347048986	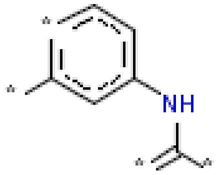	0.655	4 out of 6

Top Features for Non-Carcinogen

Fingerprint	Bit	Structure/Smiles	Score	Carcinogen in training set
SCFP_6	-1211866396	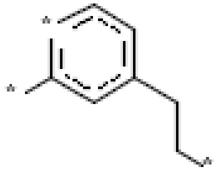	-1.233	1 out of 22
SCFP_6	-1343150366	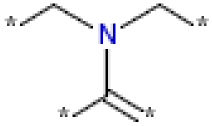	-1.017	1 out of 17
SCFP_6	-1642341584	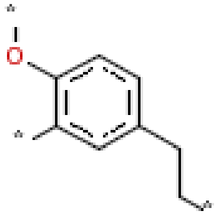	-0.847	0 out of 5

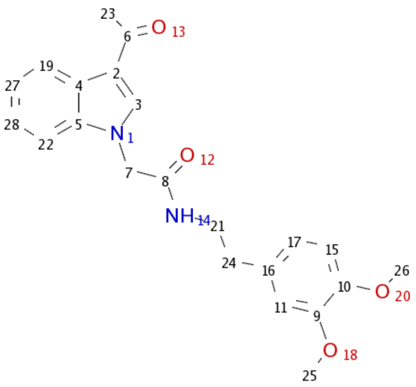

$C_{22}H_{24}N_2O_4$
Molecular Weight: 380.43696
ALogP: 3.001
Rotatable Bonds: 8
Acceptors: 4
Donors: 1

Structural Similar Compounds

Name	Propafenone	Glyburide	Ondansetron
Structure	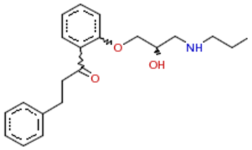	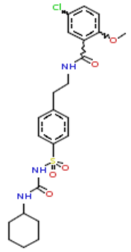	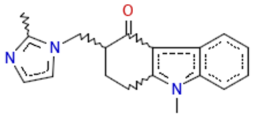
Actual Endpoint	Non-Carcinogen	Non-Carcinogen	Non-Carcinogen
Predicted Endpoint	Non-Carcinogen	Non-Carcinogen	Non-Carcinogen
Similarity	0.345	0.344	0.333
Reference	US FDA (Centre for Drug Eval.& Res./Off. Testing & Res.) Sept. 1997	US FDA (Centre for Drug Eval.& Res./Off. Testing & Res.) Sept. 1997	US FDA (Centre for Drug Eval.& Res./Off. Testing & Res.) Sept. 1997

Model Prediction

Prediction: Non-Carcinogen
Probability: 0.175
Enrichment: 0.596
Bayesian Score: -7.808

Prediction: Positive if the Bayesian score is above the estimated best cutoff value.
Probability: The esimated probability that the sample is in the category.
Enrichment: An estimate of enrichment, that is, the increased likelihood (versus random) of this sample being in the category.
Bayesian Score: The standard Laplacian-modified Bayesian score.

Model Applicability

Missing features are fingerprint features in the query molecule not found in the training set.

1. Missing Feature: SCFP_6 1964617636 [*]C(=[*])Cn(:[*]):[*]

Feature Contribution

Top features for Carcinogen				
**Fingerprint**	**Bit**	**Structure/Smiles**	**Score**	**Carcinogen in training set**
SCFP_6	1651620003	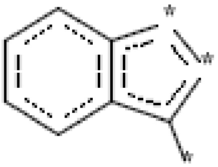	0.468	4 out of 8

SCFP_6	-1379673609	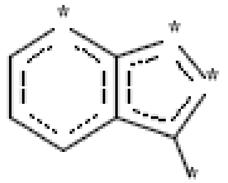	0.337	6 out of 15
SCFP_6	125999298	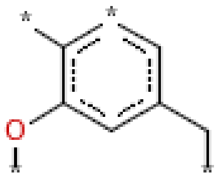	0.234	7 out of 20

Top Features for Non-Carcinogen

Fingerprint	Bit	Structure/Smiles	Score	Carcinogen in training set
SCFP_6	-1211866396	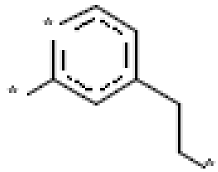	-1.233	1 out of 22
SCFP_6	-1642341584	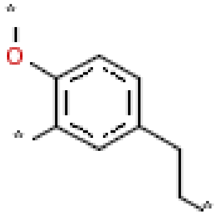	-0.847	0 out of 5
SCFP_6	-1850586302	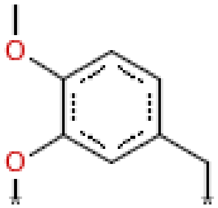	-0.587	0 out of 3

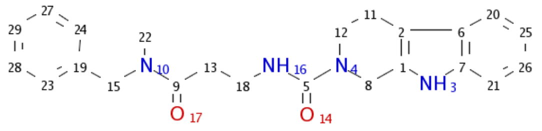

$C_{23}H_{26}N_4O_2$
Molecular Weight: 390.47814
ALogP: 2.702
Rotatable Bonds: 5
Acceptors: 2
Donors: 2

Model Prediction

Prediction: Carcinogen
Probability: 0.243
Enrichment: 0.825
Bayesian Score: -1.515

Prediction: Positive if the Bayesian score is above the estimated best cutoff value.
Probability: The esimated probability that the sample is in the category.
Enrichment: An estimate of enrichment, that is, the increased likelihood (versus random) of this sample being in the category.
Bayesian Score: The standard Laplacian-modified Bayesian score.

Structural Similar Compounds

Name	Etodolac	Tryptophan	Glimepride
Structure	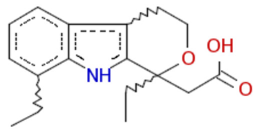	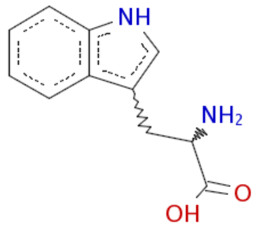	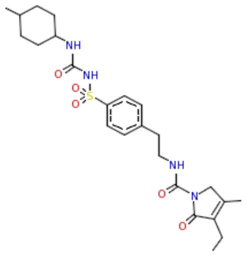
Actual Endpoint	Non-Carcinogen	Non-Carcinogen	Carcinogen
Predicted Endpoint	Non-Carcinogen	Non-Carcinogen	Carcinogen
Similarity	0.368	0.340	0.297
Reference	US FDA (Centre for Drug Eval.& Res./Off. Testing & Res.) Sept. 1997	US FDA (Centre for Drug Eval.& Res./Off. Testing & Res.) Sept. 1997	US FDA (Centre for Drug Eval.& Res./Off. Testing & Res.) Sept. 1997

Model Applicability

Missing features are fingerprint features in the query molecule not found in the training set.

1. All descriptors are in range.

Feature Contribution

Top features for Carcinogen				
Fingerprint	Bit	Structure/Smiles	Score	Carcinogen in training set
SCFP_6	-1629661413	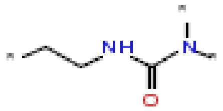	0.661	3 out of 4

SCFP_6	1651620003	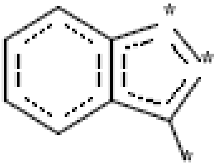	0.468	4 out of 8
SCFP_6	1962031760	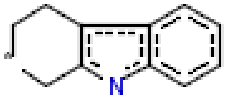	0.457	1 out of 1
**Top Features for Non-Carcinogen**				
**Fingerprint**	**Bit**	**Structure/Smiles**	**Score**	**Carcinogen in training set**
SCFP_6	-1343150366	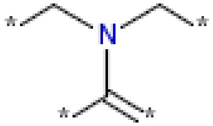	-1.017	1 out of 17
SCFP_6	403834996	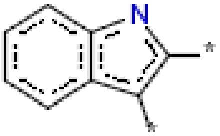	-0.725	0 out of 4
SCFP_6	2095592978	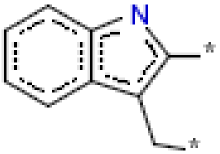	-0.587	0 out of 3

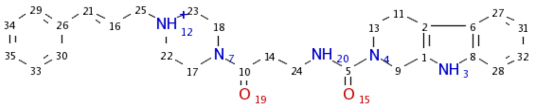

$C_{28}H_{34}N_5O_2$
Molecular Weight: 472.60186
ALogP: 1.567
Rotatable Bonds: 6
Acceptors: 2
Donors: 3

Model Prediction

Prediction: Carcinogen
Probability: 0.257
Enrichment: 0.874
Bayesian Score: -0.742

Prediction: Positive if the Bayesian score is above the estimated best cutoff value.
Probability: The esimated probability that the sample is in the category.
Enrichment: An estimate of enrichment, that is, the increased likelihood (versus random) of this sample being in the category.
Bayesian Score: The standard Laplacian-modified Bayesian score.

Structural Similar Compounds

Name	Etodolac	Tryptophan	Bromocriptine
Structure	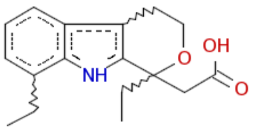	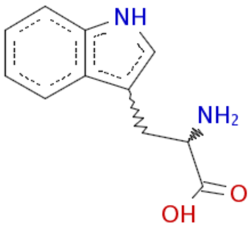	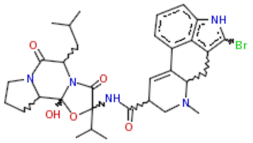
Actual Endpoint	Non-Carcinogen	Non-Carcinogen	Non-Carcinogen
Predicted Endpoint	Non-Carcinogen	Non-Carcinogen	Non-Carcinogen
Similarity	0.333	0.286	0.272
Reference	US FDA (Centre for Drug Eval.& Res./Off. Testing & Res.) Sept. 1997	US FDA (Centre for Drug Eval.& Res./Off. Testing & Res.) Sept. 1997	US FDA (Centre for Drug Eval.& Res./Off. Testing & Res.) Sept. 1997

Model Applicability

Missing features are fingerprint features in the query molecule not found in the training set.

1. Missing Feature: SCFP_6 1930718785 [*]C[NH+](C[*])C[*]
2. Missing Feature: SCFP_6 -1396915742 [*]CC[NH+]([*])[*]
3. Missing Feature: SCFP_6 -1396885951 [*][NH+]([*])CC=[*]

Feature Contribution

Top features for Carcinogen

Fingerprint	Bit	Structure/Smiles	Score	Carcinogen in training set
SCFP_6	-1629661413	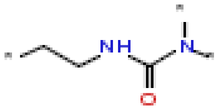	0.661	3 out of 4

SCFP_6	1651620003	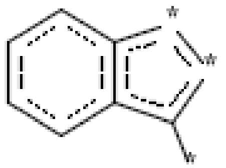	0.468	4 out of 8
SCFP_6	1962031760	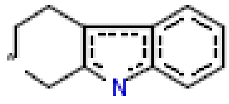	0.457	1 out of 1
**Top Features for Non-Carcinogen**				
**Fingerprint**	**Bit**	**Structure/Smiles**	**Score**	**Carcinogen in training set**
SCFP_6	-1343150366	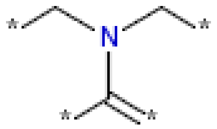	-1.017	1 out of 17
SCFP_6	403834996	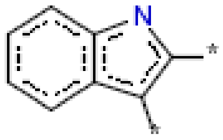	-0.725	0 out of 4
SCFP_6	-2103400817	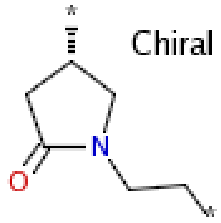	-0.587	0 out of 3

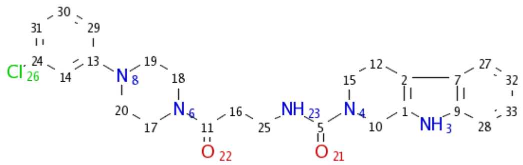

$C_{25}H_{28}ClN_5O_2$
Molecular Weight: 465.97512
ALogP: 3.413
Rotatable Bonds: 4
Acceptors: 3
Donors: 2

Model Prediction

Prediction: Non-Carcinogen
Probability: 0.181
Enrichment: 0.616
Bayesian Score: -6.767

Prediction: Positive if the Bayesian score is above the estimated best cutoff value.
Probability: The esimated probability that the sample is in the category.
Enrichment: An estimate of enrichment, that is, the increased likelihood (versus random) of this sample being in the category.
Bayesian Score: The standard Laplacian-modified Bayesian score.

Structural Similar Compounds

Name	Temazepam	Diazepam	Ketoconazole
Structure	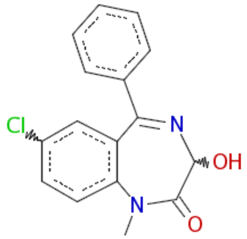	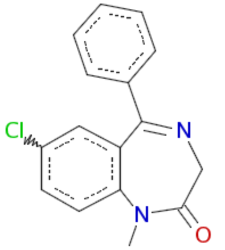	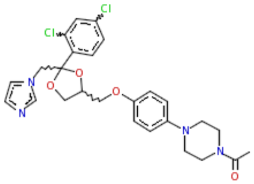
Actual Endpoint	Non-Carcinogen	Carcinogen	Non-Carcinogen
Predicted Endpoint	Carcinogen	Carcinogen	Non-Carcinogen
Similarity	0.339	0.328	0.312
Reference	US FDA (Centre for Drug Eval.& Res./Off. Testing & Res.) Sept. 1997	US FDA (Centre for Drug Eval.& Res./Off. Testing & Res.) Sept. 1997	US FDA (Centre for Drug Eval.& Res./Off. Testing & Res.) Sept. 1997

Model Applicability

Missing features are fingerprint features in the query molecule not found in the training set.

1. All descriptors are in range.

Feature Contribution

Top features for Carcinogen

Fingerprint	Bit	Structure/Smiles	Score	Carcinogen in training set
SCFP_6	-1629661413	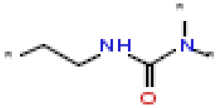	0.661	3 out of 4

SCFP_6	1651620003	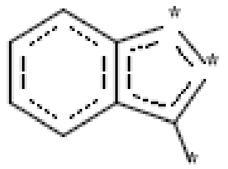	0.468	4 out of 8
SCFP_6	1962031760	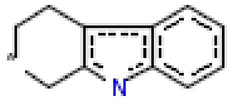	0.457	1 out of 1
**Top Features for Non-Carcinogen**				
**Fingerprint**	**Bit**	**Structure/Smiles**	**Score**	**Carcinogen in training set**
SCFP_6	-1343150366	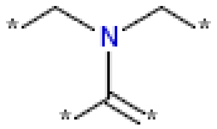	-1.017	1 out of 17
SCFP_6	403834996	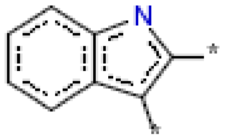	-0.725	0 out of 4
SCFP_6	-2103400817	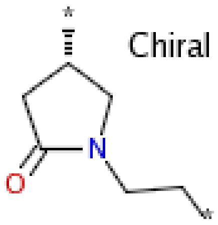	-0.587	0 out of 3

MOST ACTIVE.mol

TOPKAT_Mouse_Male_FDA_None_vs_Carcinogen

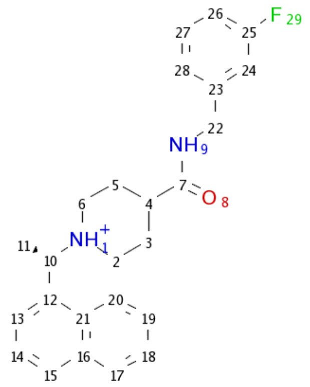

$C_{25}H_{28}FN_2O$
Molecular Weight: 391.50102
ALogP: 3.098
Rotatable Bonds: 5
Acceptors: 1
Donors: 2

Model Prediction

Prediction: Carcinogen
Probability: 0.326
Enrichment: 1.108
Bayesian Score: 2.069

Prediction: Positive if the Bayesian score is above the estimated best cutoff value.
Probability: The esimated probability that the sample is in the category.
Enrichment: An estimate of enrichment, that is, the increased likelihood (versus random) of this sample being in the category.
Bayesian Score: The standard Laplacian-modified Bayesian score.

Structural Similar Compounds

Name	Bupropion	Chlorpropamide	Phenobarbital
Structure	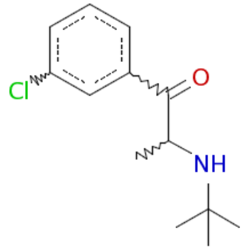	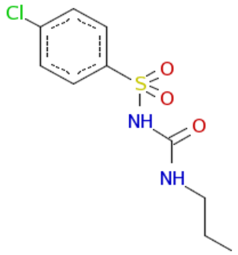	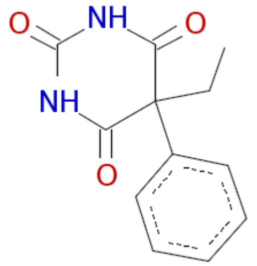
Actual Endpoint	Non-Carcinogen	Non-Carcinogen	Carcinogen
Predicted Endpoint	Non-Carcinogen	Non-Carcinogen	Carcinogen
Similarity	0.362	0.327	0.311
Reference	US FDA (Centre for Drug Eval.& Res./Off. Testing & Res.) Sept. 1997	US FDA (Centre for Drug Eval.& Res./Off. Testing & Res.) Sept. 1997	US FDA (Centre for Drug Eval.& Res./Off. Testing & Res.) Sept. 1997

Model Applicability

Missing features are fingerprint features in the query molecule not found in the training set.

1. Missing Feature: SCFP_6 1930718785 [*]C[NH+](C[*])C[*]
2. Missing Feature: SCFP_6 -1396915742 [*]CC[NH+]([*])[*]
3. Missing Feature: SCFP_6 -1688664082 [*][NH+]([*])[C@H](C)[c](:[*]):[*]

Feature Contribution

Top features for Carcinogen

Fingerprint	Bit	Structure/Smiles	Score	Carcinogen in training set
SCFP_6	2098257782	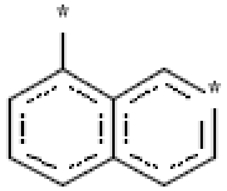	0.511	2 out of 3

SCFP_6	-1043310069	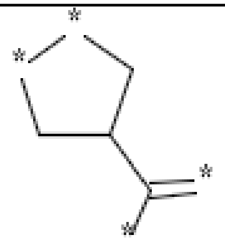	0.504	13 out of 28
SCFP_6	1651620003	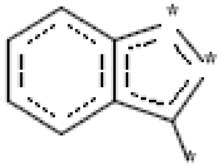	0.468	4 out of 8
**Top Features for Non-Carcinogen**				
**Fingerprint**	**Bit**	**Structure/Smiles**	**Score**	**Carcinogen in training set**
SCFP_6	-1794884847	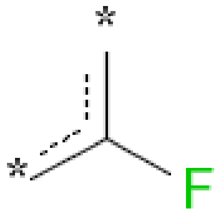	-0.562	2 out of 16
SCFP_6	-730654023	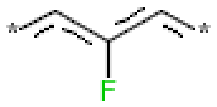	-0.562	2 out of 16
SCFP_6	-1381307546	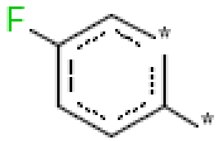	-0.455	2 out of 14

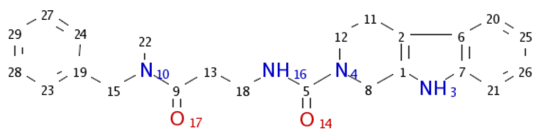

$C_{23}H_{26}N_4O_2$
Molecular Weight: 390.47814
ALogP: 2.702
Rotatable Bonds: 5
Acceptors: 2
Donors: 2

Model Prediction

Prediction: Single-Carcinogen
Probability: 0.220
Enrichment: 0.729
Bayesian Score: -2.238

Prediction: Positive if the Bayesian score is above the estimated best cutoff value.
Probability: The esimated probability that the sample is in the category.
Enrichment: An estimate of enrichment, that is, the increased likelihood (versus random) of this sample being in the category.
Bayesian Score: The standard Laplacian-modified Bayesian score.

Structural Similar Compounds

Name	Glimepride	Methylphenidate	Reserpine
Structure	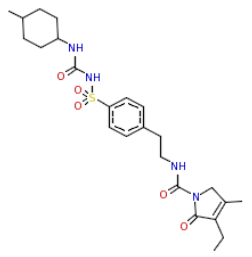	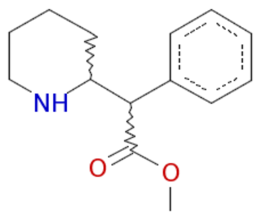	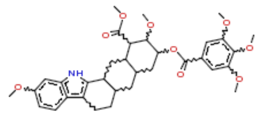
Actual Endpoint	Single-Carcinogen	Single-Carcinogen	Multiple-Carcinogen
Predicted Endpoint	Single-Carcinogen	Single-Carcinogen	Multiple-Carcinogen
Similarity	0.297	0.283	0.264
Reference	US FDA (Centre for Drug Eval.& Res./Off. Testing & Res.) Sept. 1997	US FDA (Centre for Drug Eval.& Res./Off. Testing & Res.) Sept. 1997	US FDA (Centre for Drug Eval.& Res./Off. Testing & Res.) Sept. 1997

Model Applicability

Missing features are fingerprint features in the query molecule not found in the training set.

1. All descriptors are in range.

Feature Contribution

Top features for Multiple-Carcinogen

Fingerprint	Bit	Structure/Smiles	Score	Multiple-Carcinogen in training set
FCFP_4	-1826427264	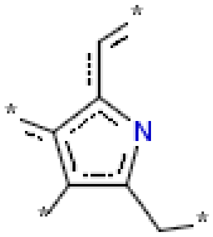	0.421	1 out of 1

FCFP_4	-1169541771	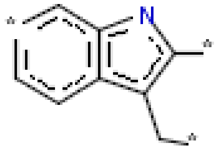	0.421	1 out of 1
FCFP_4	1673997923	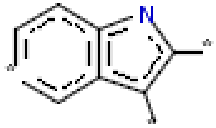	0.421	1 out of 1

Top Features for Single-Carcinogen

Fingerprint	Bit	Structure/Smiles	Score	Multiple-Carcinogen in training set
FCFP_4	907036844	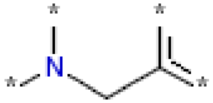	-0.662	0 out of 3
FCFP_4	991735244	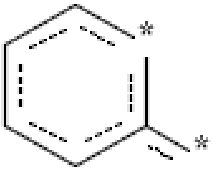	-0.486	5 out of 28
FCFP_4	159404153	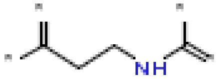	-0.486	0 out of 2

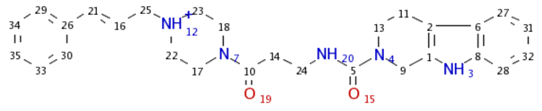

$C_{28}H_{34}N_5O_2$
Molecular Weight: 472.60186
ALogP: 1.567
Rotatable Bonds: 6
Acceptors: 2
Donors: 3

Model Prediction

Prediction: Single-Carcinogen
Probability: 0.228
Enrichment: 0.757
Bayesian Score: -1.919

Prediction: Positive if the Bayesian score is above the estimated best cutoff value.
Probability: The esimated probability that the sample is in the category.
Enrichment: An estimate of enrichment, that is, the increased likelihood (versus random) of this sample being in the category.
Bayesian Score: The standard Laplacian-modified Bayesian score.

Structural Similar Compounds

Name	Methylphenidate	Glimepride	Reserpine
Structure	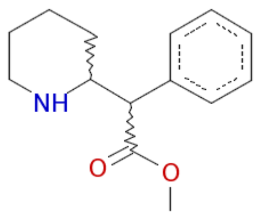	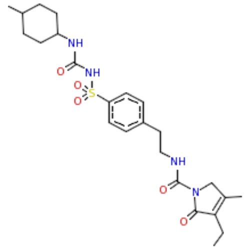	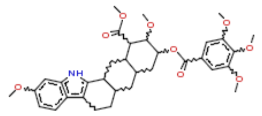
Actual Endpoint	Single-Carcinogen	Single-Carcinogen	Multiple-Carcinogen
Predicted Endpoint	Single-Carcinogen	Single-Carcinogen	Multiple-Carcinogen
Similarity	0.258	0.257	0.232
Reference	US FDA (Centre for Drug Eval.& Res./Off. Testing & Res.) Sept. 1997	US FDA (Centre for Drug Eval.& Res./Off. Testing & Res.) Sept. 1997	US FDA (Centre for Drug Eval.& Res./Off. Testing & Res.) Sept. 1997

Model Applicability

Missing features are fingerprint features in the query molecule not found in the training set.

1. Missing Feature: FCFP_4 10 [*][NH+]([*])[*]
2. Missing Feature: FCFP_4 -1853714334 [*]C[NH+](C[*])C[*]
3. Missing Feature: FCFP_4 1155241219 [*]CC[NH+]([*])[*]

Feature Contribution

Top features for Multiple-Carcinogen

Fingerprint	Bit	Structure/Smiles	Score	Multiple-Carcinogen in training set
FCFP_4	-1169541771	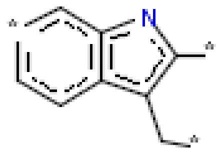	0.421	1 out of 1

FCFP_4	1673997923	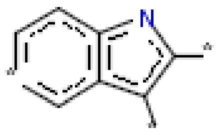	0.421	1 out of 1
FCFP_4	-1826427264	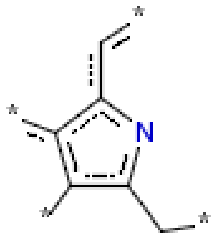	0.421	1 out of 1

Top Features for Single-Carcinogen

**Fingerprint**	**Bit**	**Structure/Smiles**	**Score**	**Multiple-Carcinogen in training set**
FCFP_4	907036844	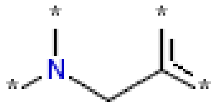	-0.662	0 out of 3
FCFP_4	991735244	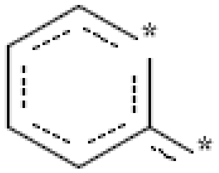	-0.486	5 out of 28
FCFP_4	159404153	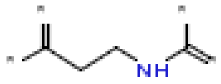	-0.486	0 out of 2

MOST ACTIVE.mol

TOPKAT_Mouse_Male_FDA_Single_vs_Multiple

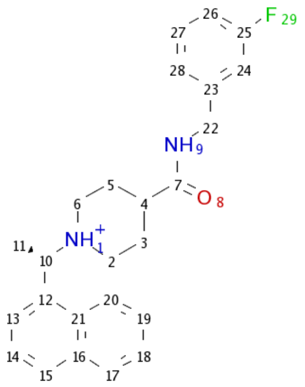

$C_{25}H_{28}FN_2O$
Molecular Weight: 391.50102
ALogP: 3.098
Rotatable Bonds: 5
Acceptors: 1
Donors: 2

Model Prediction

Prediction: Single-Carcinogen
Probability: 0.184
Enrichment: 0.610
Bayesian Score: -3.965

Prediction: Positive if the Bayesian score is above the estimated best cutoff value.
Probability: The esimated probability that the sample is in the category.
Enrichment: An estimate of enrichment, that is, the increased likelihood (versus random) of this sample being in the category.
Bayesian Score: The standard Laplacian-modified Bayesian score.

Structural Similar Compounds

Name	Phenobarbital	Sertraline	Diazepam
Structure	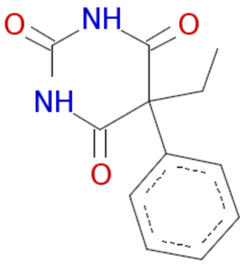	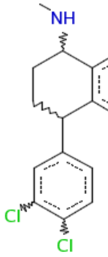	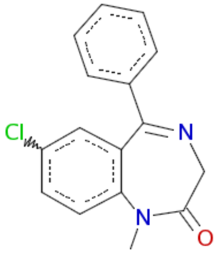
Actual Endpoint	Multiple-Carcinogen	Single-Carcinogen	Multiple-Carcinogen
Predicted Endpoint	Single-Carcinogen	Single-Carcinogen	Single-Carcinogen
Similarity	0.311	0.306	0.302
Reference	US FDA (Centre for Drug Eval.& Res./Off. Testing & Res.) Sept. 1997	US FDA (Centre for Drug Eval.& Res./Off. Testing & Res.) Sept. 1997	US FDA (Centre for Drug Eval.& Res./Off. Testing & Res.) Sept. 1997

Model Applicability

Missing features are fingerprint features in the query molecule not found in the training set.

1. Missing Feature: FCFP_4 10 [*][NH+]([*])[*]
2. Missing Feature: FCFP_4 -1853714334 [*]C[NH+](C[*])C[*]
3. Missing Feature: FCFP_4 1155241219 [*]CC[NH+]([*])[*]
4. Missing Feature: FCFP_4 -680623486 [*][NH+]([*])[C@H](C)[c](:[*]):[*]

Feature Contribution

Top features for Multiple-Carcinogen				
**Fingerprint**	**Bit**	**Structure/Smiles**	**Score**	**Multiple-Carcinogen in training set**
FCFP_4	-1043339860	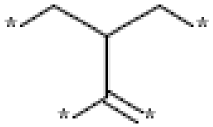	0.424	12 out of 24

FCFP_4	427906732	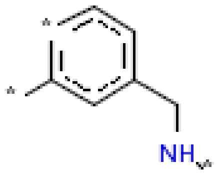	0.421	1 out of 1
FCFP_4	907096426	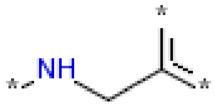	0.421	1 out of 1

Top Features for Single-Carcinogen

Fingerprint	Bit	Structure/Smiles	Score	Multiple-Carcinogen in training set
FCFP_4	71476542	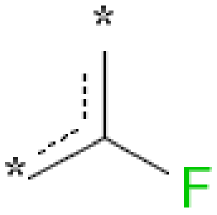	-0.725	1 out of 10
FCFP_4	367998008	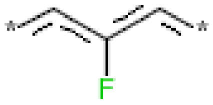	-0.725	1 out of 10
FCFP_4	551850122	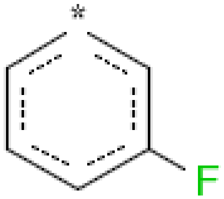	-0.560	1 out of 8

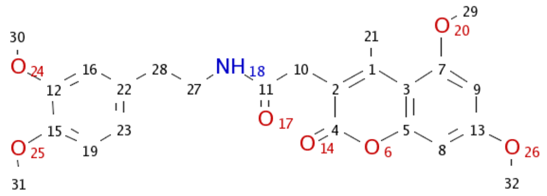

$C_{24}H_{27}NO_7$
Molecular Weight: 441.47368
ALogP: 3.388
Rotatable Bonds: 9
Acceptors: 7
Donors: 1

Model Prediction

Prediction: Moderate_Severe
Probability: 0.887
Enrichment: 1.288
Bayesian Score: 1.972

Prediction: Positive if the Bayesian score is above the estimated best cutoff value.
Probability: The esimated probability that the sample is in the category.
Enrichment: An estimate of enrichment, that is, the increased likelihood (versus random) of this sample being in the category.
Bayesian Score: The standard Laplacian-modified Bayesian score.

Structural Similar Compounds

Name	NPE 9	NPE 10	NPE 13
Structure	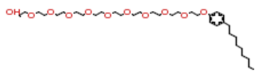	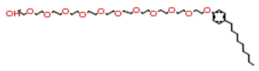	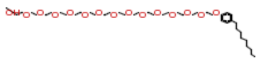
Actual Endpoint	Moderate_Severe	Moderate_Severe	Moderate_Severe
Predicted Endpoint	Moderate_Severe	Moderate_Severe	Moderate_Severe
Similarity	0.370	0.370	0.370
Reference	In: Environ. and Human Safety of Major Surfactants; Talmage; S; 1994 Lewis Publi	In: Environ. and Human Safety of Major Surfactants; Talmage; S; 1994 Lewis Publi	In: Environ. and Human Safety of Major Surfactants; Talmage; S; 1994 Lewis Publi

Model Applicability

Missing features are fingerprint features in the query molecule not found in the training set.

1. All descriptors are in range.

Feature Contribution

Top features for Moderate_Severe				
**Fingerprint**	**Bit**	**Structure/Smiles**	**Score**	**Moderate_Severe in training set**
FCFP_8	1985089045	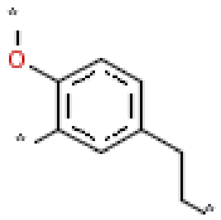	0.386	16 out of 16

FCFP_8	-497728148	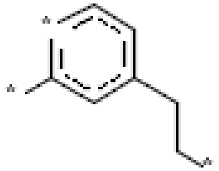	0.357	24 out of 25
FCFP_8	-547731249	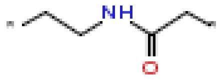	0.295	3 out of 3

Top Features for Mild

Fingerprint	Bit	Structure/Smiles	Score	Moderate_Severe in training set
FCFP_8	-1977641857	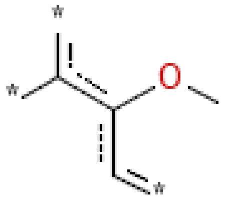	-0.779	4 out of 15
FCFP_8	-1099193755	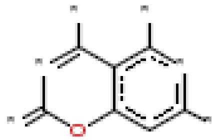	-0.359	2 out of 5
FCFP_8	136627117	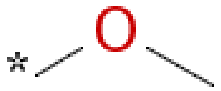	-0.314	46 out of 96

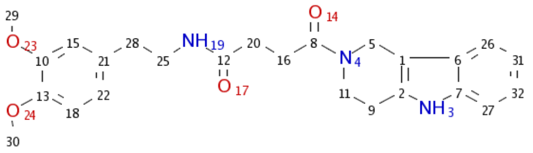

$C_{25}H_{29}N_3O_4$
Molecular Weight: 435.51546
ALogP: 2.831
Rotatable Bonds: 8
Acceptors: 4
Donors: 2

Model Prediction

Prediction: Mild

Probability: 0.827

Enrichment: 1.201

Bayesian Score: -0.779

Prediction: Positive if the Bayesian score is above the estimated best cutoff value.
Probability: The esimated probability that the sample is in the category.
Enrichment: An estimate of enrichment, that is, the increased likelihood (versus random) of this sample being in the category.
Bayesian Score: The standard Laplacian-modified Bayesian score.

Structural Similar Compounds

Name	ISOQUINOLINE; 1;2;3;4-TETRAHYDRO-2-OCTANOYL-	CARBAMIC ACID; METHYL-; 1-(5;6;7;8-TETRAHYDRO)NAPHTHYL ESTER	QUINOLINE; 1;2;3;4-TETRAHYDRO-1-(2-METHYL-1-OXOPENTYL)-
Structure	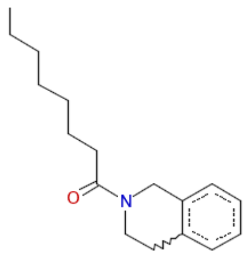	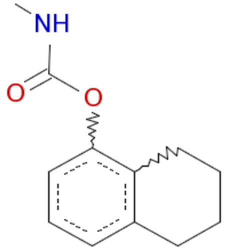	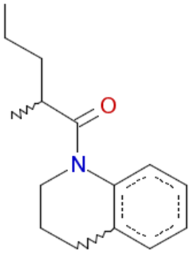
Actual Endpoint	Mild	Mild	Mild
Predicted Endpoint	Mild	Mild	Mild
Similarity	0.358	0.291	0.281
Reference	NTIS** AD-A042-527	28ZPAK-;163;72	NTIS** AD-A056-106

Model Applicability

Missing features are fingerprint features in the query molecule not found in the training set.

1. Missing Feature: FCFP_8 203707511 [*]C[c]1:n:[*]:[*]:[c]:1[*]

Feature Contribution

Top features for Moderate_Severe				
**Fingerprint**	**Bit**	**Structure/Smiles**	**Score**	**Moderate_Severe in training set**
FCFP_8	1985089045	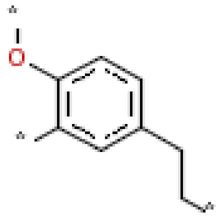	0.386	16 out of 16

FCFP_8	-497728148	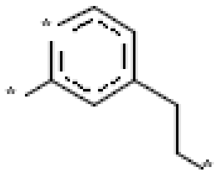	0.357	24 out of 25
FCFP_8	-547731249	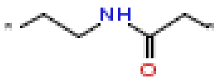	0.295	3 out of 3

Top Features for Mild

Fingerprint	Bit	Structure/Smiles	Score	Moderate_Severe in training set
FCFP_8	-1977641857	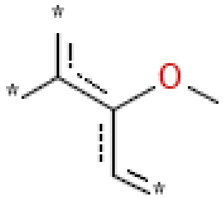	-0.779	4 out of 15
FCFP_8	907036844	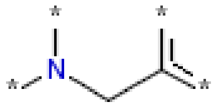	-0.598	1 out of 4
FCFP_8	-1134225568	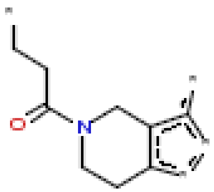	-0.506	0 out of 1

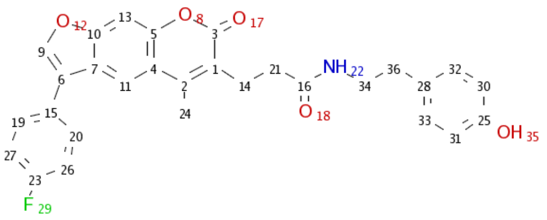

$C_{29}H_{24}FNO_5$
Molecular Weight: 485.50296
ALogP: 5.695
Rotatable Bonds: 7
Acceptors: 4
Donors: 2

Model Prediction

Prediction: Moderate_Severe
Probability: 0.895
Enrichment: 1.300
Bayesian Score: 2.204

Prediction: Positive if the Bayesian score is above the estimated best cutoff value.
Probability: The esimated probability that the sample is in the category.
Enrichment: An estimate of enrichment, that is, the increased likelihood (versus random) of this sample being in the category.
Bayesian Score: The standard Laplacian-modified Bayesian score.

Structural Similar Compounds

Name	NONYLPHENOL	ACETIC ACID; 2-CHLORO-5-NITROPHENYL ESTER	CYCLOHEXENYL-4-PHENOL
Structure	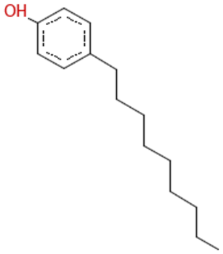	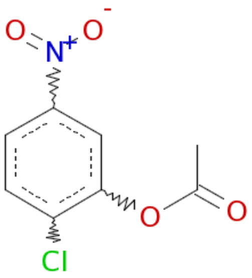	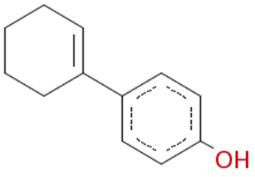
Actual Endpoint	Moderate_Severe	Mild	Moderate_Severe
Predicted Endpoint	Moderate_Severe	Mild	Moderate_Severe
Similarity	0.291	0.262	0.259
Reference	In: Environ. and Human Safety of Major Surfactants; Talmage; S; 1994 Lewis Publi	28ZPAK-;92;72	AIHAAP 30;470;69

Model Applicability

Missing features are fingerprint features in the query molecule not found in the training set.

1. All descriptors are in range.

Feature Contribution

Top features for Moderate_Severe

Fingerprint	Bit	Structure/Smiles	Score	Moderate_Severe in training set
FCFP_8	-1066794953	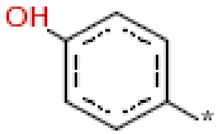	0.380	13 out of 13

FCFP_8	-497728148	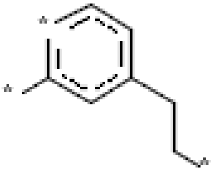	0.357	24 out of 25
FCFP_8	-158888774	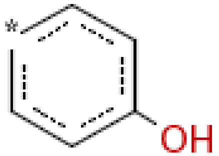 	0.357	24 out of 25

Top Features for Mild

Fingerprint	Bit	Structure/Smiles	Score	Moderate_Severe in training set
FCFP_8	-620155118	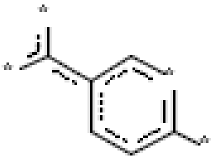	-0.596	9 out of 26
FCFP_8	-1861645784	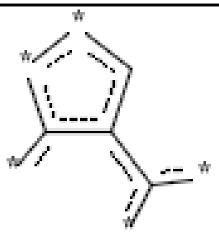	-0.596	9 out of 26
FCFP_8	565114151	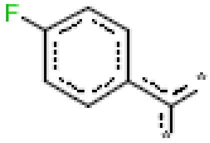 	-0.506	0 out of 1

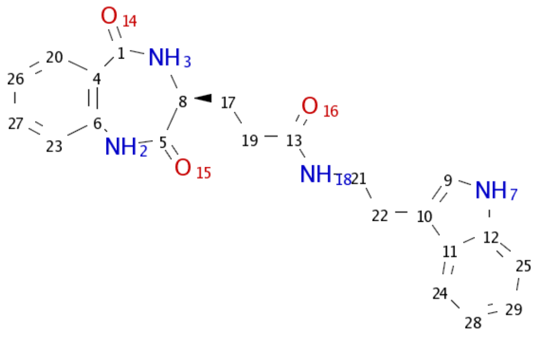

$C_{22}H_{22}N_4O_3$
Molecular Weight: 390.43508
ALogP: 1.807
Rotatable Bonds: 6
Acceptors: 3
Donors: 4

Model Prediction

Prediction: Moderate_Severe
Probability: 0.856
Enrichment: 1.243
Bayesian Score: 1.167

Prediction: Positive if the Bayesian score is above the estimated best cutoff value.
Probability: The esimated probability that the sample is in the category.
Enrichment: An estimate of enrichment, that is, the increased likelihood (versus random) of this sample being in the category.
Bayesian Score: The standard Laplacian-modified Bayesian score.

Structural Similar Compounds

Name	ACETANILIDE; 4'-(2-HYDROXYETHYLSULFONYL)-	2-NAPHTHALENE SULFONIC ACID;5-AMINO-	1-AMINO-4-BENZOYLAMINO-ANTHRAQUINONE
Structure	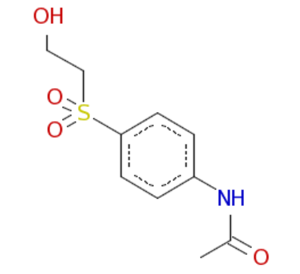	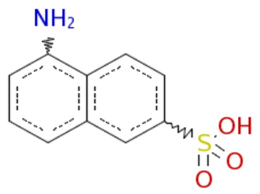	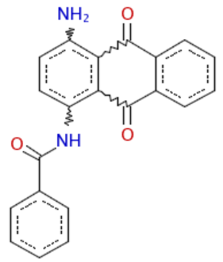
Actual Endpoint	Mild	Mild	Mild
Predicted Endpoint	Mild	Mild	Mild
Similarity	0.288	0.280	0.269
Reference	28ZPAK-;201;72	28ZPAK-;187;72	28ZPAK-;124;72

Model Applicability

Missing features are fingerprint features in the query molecule not found in the training set.

1. All descriptors are in range.

Feature Contribution

Top features for Moderate_Severe

Fingerprint	Bit	Structure/Smiles	Score	Moderate_Severe in training set
FCFP_8	-547731249	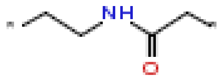	0.295	3 out of 3

FCFP_8	-1272709286	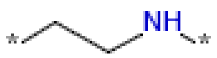	0.287	234 out of 266
FCFP_8	155061250	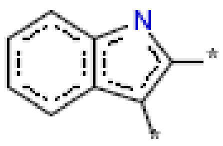	0.258	2 out of 2
**Top Features for Mild**				
**Fingerprint**	**Bit**	**Structure/Smiles**	**Score**	**Moderate_Severe in training set**
FCFP_8	-1549103449	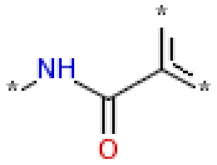	-0.502	2 out of 6
FCFP_8	1011367537	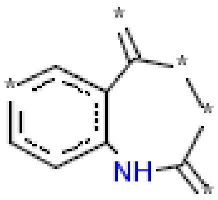	-0.327	4 out of 9
FCFP_8	-1320007763	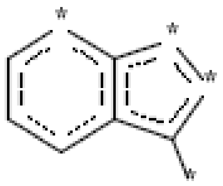	-0.314	19 out of 40

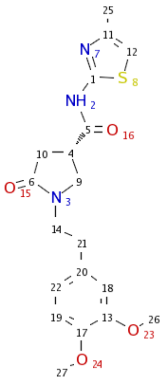

$C_{19}H_{23}N_3O_4S$
Molecular Weight: 389.46862
ALogP: 1.461
Rotatable Bonds: 7
Acceptors: 5
Donors: 1

Structural Similar Compounds

Name	ISOQUINOLINE; 1;2;3;4-TETRAHYDRO-2-OCTANOYL-	NPE 10	NPE 12
Structure	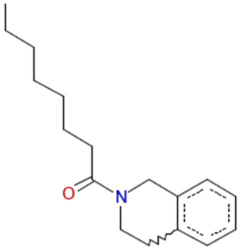	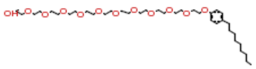	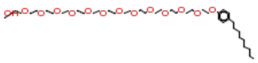
Actual Endpoint	Mild	Moderate_Severe	Moderate_Severe
Predicted Endpoint	Mild	Moderate_Severe	Moderate_Severe
Similarity	0.340	0.327	0.327
Reference	NTIS** AD-A042-527	In: Environ. and Human Safety of Major Surfactants; Talmage; S; 1994 Lewis Publi	In: Environ. and Human Safety of Major Surfactants; Talmage; S; 1994 Lewis Publi

Model Prediction

Prediction: Mild
Probability: 0.835
Enrichment: 1.211
Bayesian Score: -0.462

Prediction: Positive if the Bayesian score is above the estimated best cutoff value.
Probability: The esimated probability that the sample is in the category.
Enrichment: An estimate of enrichment, that is, the increased likelihood (versus random) of this sample being in the category.
Bayesian Score: The standard Laplacian-modified Bayesian score.

Model Applicability

Missing features are fingerprint features in the query molecule not found in the training set.

1. All descriptors are in range.

Feature Contribution

Top features for Moderate_Severe

Fingerprint	Bit	Structure/Smiles	Score	Moderate_Severe in training set
FCFP_8	1985089045	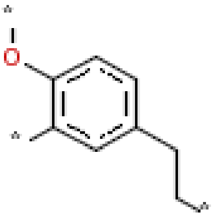	0.386	16 out of 16

FCFP_8	-497728148	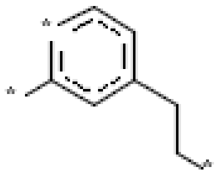	0.357	24 out of 25
FCFP_8	-124655670	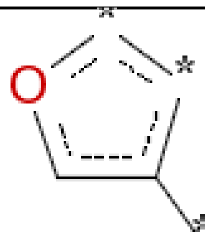	0.261	14 out of 16
**Top Features for Mild**				
**Fingerprint**	**Bit**	**Structure/Smiles**	**Score**	**Moderate_Severe in training set**
FCFP_8	-1977641857	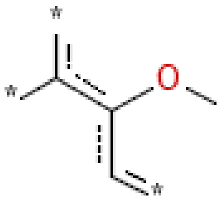	-0.779	4 out of 15
FCFP_8	-2005207466	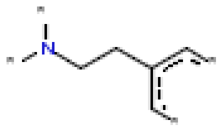	-0.506	0 out of 1
FCFP_8	206055935	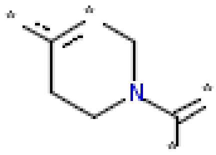	-0.506	0 out of 1

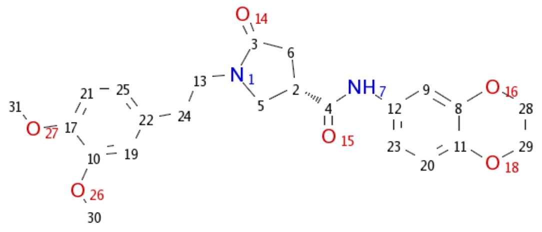

$C_{23}H_{26}N_2O_6$
Molecular Weight: 426.46234
ALogP: 1.967
Rotatable Bonds: 7
Acceptors: 6
Donors: 1

Structural Similar Compounds

Name	p-Acetophenetidide; 3'-(bis(2-hydroxyethyl)amino)-	P-ACETOPHENETIDIDE;3-NITRO-	ACETANILIDE; 3'-AMINO-4'-ETHOXY-
Structure	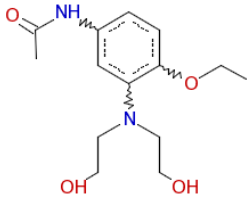	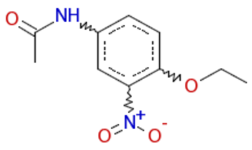	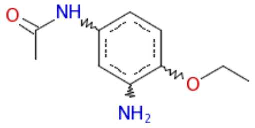
Actual Endpoint	Moderate_Severe	Moderate_Severe	Mild
Predicted Endpoint	Moderate_Severe	Moderate_Severe	Mild
Similarity	0.444	0.409	0.395
Reference	Prehled Prumyslove Toxikologie; Organicke Latky; Marhold; J. -;645;86	28ZPAK-;115;72	28ZPAK 115;72

Model Prediction

Prediction: Moderate_Severe
Probability: 0.840
Enrichment: 1.219
Bayesian Score: -0.215

Prediction: Positive if the Bayesian score is above the estimated best cutoff value.
Probability: The esimated probability that the sample is in the category.
Enrichment: An estimate of enrichment, that is, the increased likelihood (versus random) of this sample being in the category.
Bayesian Score: The standard Laplacian-modified Bayesian score.

Model Applicability

Missing features are fingerprint features in the query molecule not found in the training set.

1. All descriptors are in range.

Feature Contribution

Top features for Moderate_Severe				
**Fingerprint**	**Bit**	**Structure/Smiles**	**Score**	**Moderate_Severe in training set**
FCFP_8	365650923	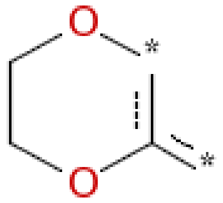	0.388	17 out of 17

FCFP_8	1985089045	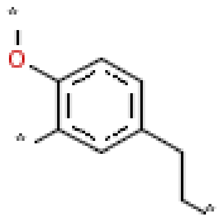	0.386	16 out of 16
FCFP_8	-497728148	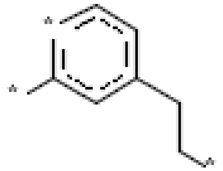	0.357	24 out of 25
**Top Features for Mild**				
**Fingerprint**	**Bit**	**Structure/Smiles**	**Score**	**Moderate_Severe in training set**
FCFP_8	-1977641857	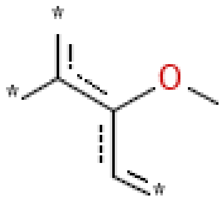	-0.779	4 out of 15
FCFP_8	206055935	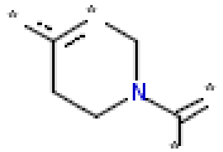	-0.506	0 out of 1
FCFP_8	-2005207466	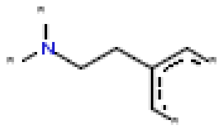	-0.506	0 out of 1

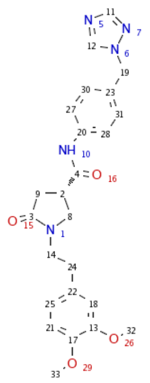

$C_{24}H_{27}N_5O_4$
Molecular Weight: 449.50228
ALogP: 1.731
Rotatable Bonds: 9
Acceptors: 6
Donors: 1

Structural Similar Compounds

Name	p-Acetophenetidide; 3'-(bis(2-hydroxyethyl)amino)-	0-ANISIDINE;ACETOACETYL	ISOQUINOLINE; 1;2;3;4-TETRAHYDRO-2-OCTANOYL-
Structure	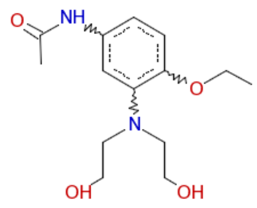	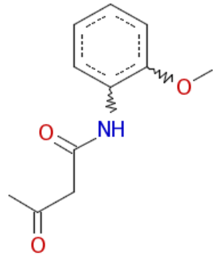	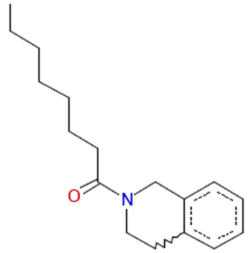
Actual Endpoint	Moderate_Severe	Mild	Mild
Predicted Endpoint	Moderate_Severe	Mild	Mild
Similarity	0.316	0.315	0.315
Reference	Prehled Prumyslove Toxikologie; Organicke Latky; Marhold; J. -;645;86	28ZPAK-;116;72	NTIS** AD-A042-527

Model Prediction

Prediction: Mild

Probability: 0.692

Enrichment: 1.004

Bayesian Score: -3.557

Prediction: Positive if the Bayesian score is above the estimated best cutoff value.
Probability: The esimated probability that the sample is in the category.
Enrichment: An estimate of enrichment, that is, the increased likelihood (versus random) of this sample being in the category.
Bayesian Score: The standard Laplacian-modified Bayesian score.

Model Applicability

Missing features are fingerprint features in the query molecule not found in the training set.

1. Missing Feature: FCFP_8 -2049666792 [*]Cn1:c:[*]:[*]:n:1
2. Missing Feature: FCFP_8 -124685461 [*]1:[*]:n:c:n:1
3. Missing Feature: FCFP_8 906560188 [*]:[c](:[*])Cn(:[*]):[*]

Feature Contribution

Top features for Moderate_Severe				
**Fingerprint**	**Bit**	**Structure/Smiles**	**Score**	**Moderate_Severe in training set**
FCFP_8	1985089045	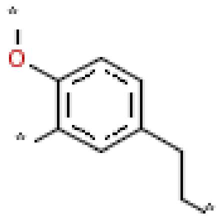	0.386	16 out of 16

FCFP_8	-497728148	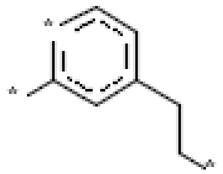	0.357	24 out of 25
FCFP_8	1028934530	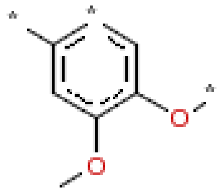	0.258	2 out of 2

Top Features for Mild

Fingerprint	Bit	Structure/Smiles	Score	Moderate_Severe in training set
FCFP_8	4427049	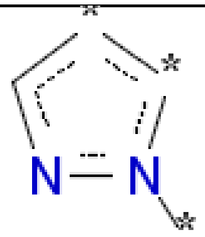	-1.291	0 out of 4
FCFP_8	-1977641857	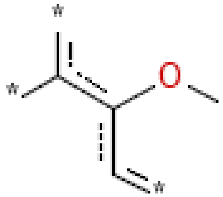	-0.779	4 out of 15
FCFP_8	-790336137	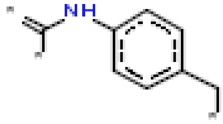	-0.506	0 out of 1

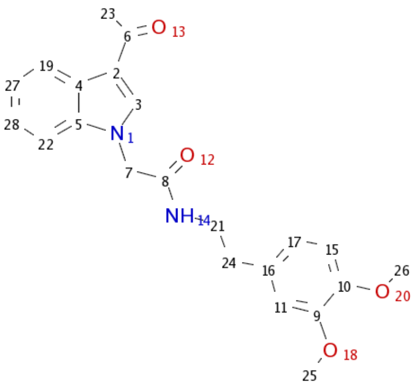

$C_{22}H_{24}N_2O_4$
Molecular Weight: 380.43696
ALogP: 3.001
Rotatable Bonds: 8
Acceptors: 4
Donors: 1

Model Prediction

Prediction: Mild
Probability: 0.835
Enrichment: 1.212
Bayesian Score: -0.436

Prediction: Positive if the Bayesian score is above the estimated best cutoff value.
Probability: The esimated probability that the sample is in the category.
Enrichment: An estimate of enrichment, that is, the increased likelihood (versus random) of this sample being in the category.
Bayesian Score: The standard Laplacian-modified Bayesian score.

Structural Similar Compounds

Name	7H-BENZIMIDAZO(2;1-a)BENZ(de)ISOQUINOLIN-7-ONE; 10-METHOXY-	CARBAMIC ACID; METHYL-; 1-(5;6;7;8-TETRAHYDRO)NAPHTHYL ESTER	ANTHRAQUINONE;1;5-DIMETHOXY
Structure	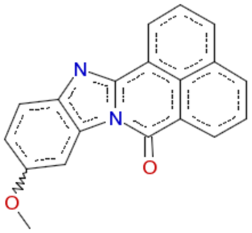	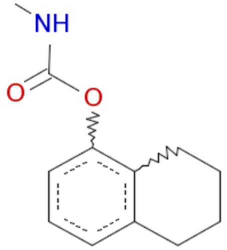	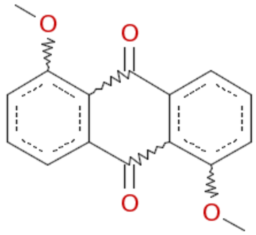
Actual Endpoint	Mild	Mild	Mild
Predicted Endpoint	Mild	Mild	Mild
Similarity	0.333	0.327	0.311
Reference	28ZPAK 147;72	28ZPAK-;163;72	28ZPAK-;113;72

Model Applicability

Missing features are fingerprint features in the query molecule not found in the training set.

1. Missing Feature: FCFP_8 -1645149908 [*]C(=[*])Cn(:[*]):[*]

Feature Contribution

Top features for Moderate_Severe				
**Fingerprint**	**Bit**	**Structure/Smiles**	**Score**	**Moderate_Severe in training set**
FCFP_8	1985089045	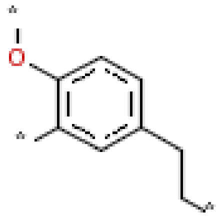	0.386	16 out of 16

FCFP_8	-497728148	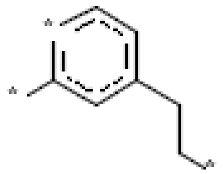	0.357	24 out of 25
FCFP_8	-547731249	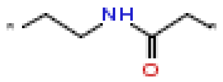 	0.295	3 out of 3

Top Features for Mild

Fingerprint	Bit	Structure/Smiles	Score	Moderate_Severe in training set
FCFP_8	-306856457	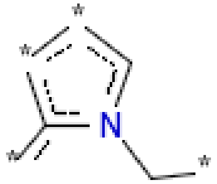 	-0.841	0 out of 2
FCFP_8	-1977641857	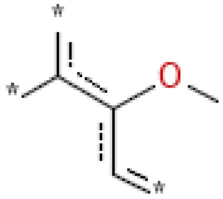 	-0.779	4 out of 15
FCFP_8	-1320007763	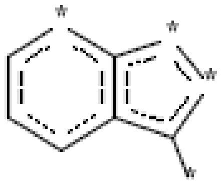	-0.314	19 out of 40

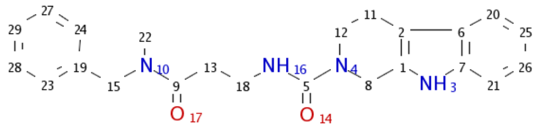

$C_{23}H_{26}N_4O_2$
Molecular Weight: 390.47814
ALogP: 2.702
Rotatable Bonds: 5
Acceptors: 2
Donors: 2

Model Prediction

Prediction: Mild
Probability: 0.717
Enrichment: 1.041
Bayesian Score: -3.210

Prediction: Positive if the Bayesian score is above the estimated best cutoff value.
Probability: The esimated probability that the sample is in the category.
Enrichment: An estimate of enrichment, that is, the increased likelihood (versus random) of this sample being in the category.
Bayesian Score: The standard Laplacian-modified Bayesian score.

Structural Similar Compounds

Name	ISOQUINOLINE; 1;2;3;4-TETRAHYDRO-2-OCTANOYL-	ETHANOL; 2-(BENZYLOXY)-	2-NAPHTHALENE SULFONIC ACID;5-AMINO-
Structure	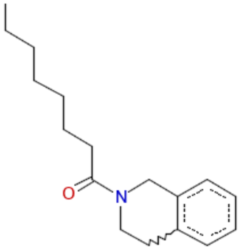	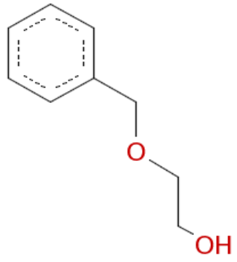	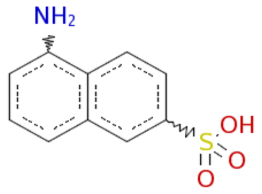
Actual Endpoint	Mild	Moderate_Severe	Mild
Predicted Endpoint	Mild	Moderate_Severe	Mild
Similarity	0.367	0.304	0.286
Reference	NTIS** AD-A042-527	AJOPAA 29;1363;46	28ZPAK-;187;72

Model Applicability

Missing features are fingerprint features in the query molecule not found in the training set.

1. Missing Feature: FCFP_8 203707511 [*]C[c]1:n:[*]:[*]:[c]:1[*]

Feature Contribution

Top features for Moderate_Severe				
**Fingerprint**	**Bit**	**Structure/Smiles**	**Score**	**Moderate_Severe in training set**
FCFP_8	159404153	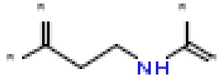	0.326	15 out of 16

FCFP_8	-1272709286	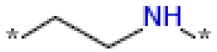	0.287	234 out of 266
FCFP_8	155061250	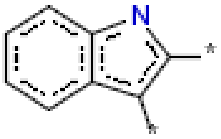	0.258	2 out of 2
**Top Features for Mild**				
**Fingerprint**	**Bit**	**Structure/Smiles**	**Score**	**Moderate_Severe in training set**
FCFP_8	907036844	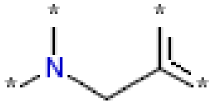	-0.598	1 out of 4
FCFP_8	599018404	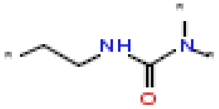	-0.506	0 out of 1
FCFP_8	206055935	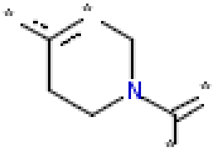	-0.506	0 out of 1

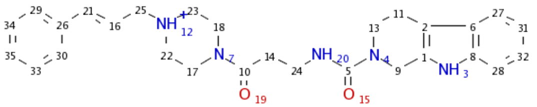

$C_{28}H_{34}N_5O_2$
Molecular Weight: 472.60186
ALogP: 1.567
Rotatable Bonds: 6
Acceptors: 2
Donors: 3

Model Prediction

Prediction: Mild
Probability: 0.754
Enrichment: 1.095
Bayesian Score: -2.624

Prediction: Positive if the Bayesian score is above the estimated best cutoff value.
Probability: The esimated probability that the sample is in the category.
Enrichment: An estimate of enrichment, that is, the increased likelihood (versus random) of this sample being in the category.
Bayesian Score: The standard Laplacian-modified Bayesian score.

Structural Similar Compounds

Name	ISOQUINOLINE; 1;2;3;4-TETRAHYDRO-2-OCTANOYL-	CARBAMIC ACID; METHYL-; 1-(5;6;7;8-TETRAHYDRO)NAPHTHYL ESTER	ETHANOL; 2-(BENZYLOXY)-
Structure	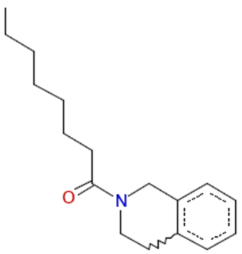	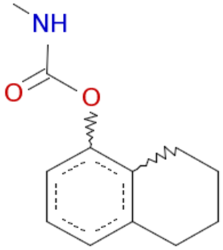	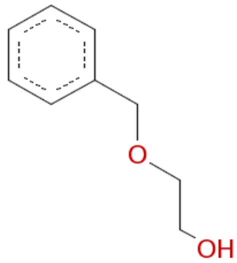
Actual Endpoint	Mild	Mild	Moderate_Severe
Predicted Endpoint	Mild	Mild	Moderate_Severe
Similarity	0.283	0.246	0.228
Reference	NTIS** AD-A042-527	28ZPAK-;163;72	AJOPAA 29;1363;46

Model Applicability

Missing features are fingerprint features in the query molecule not found in the training set.

1. Missing Feature: FCFP_8 10 [*][NH+]([*])[*]
2. Missing Feature: FCFP_8 203707511 [*]C[c]1:n:[*]:[*]:[c]:1[*]
3. Missing Feature: FCFP_8 -1853714334 [*]C[NH+](C[*])C[*]
4. Missing Feature: FCFP_8 1155241219 [*]CC[NH+]([*])[*]

Feature Contribution

Top features for Moderate_Severe				
**Fingerprint**	**Bit**	**Structure/Smiles**	**Score**	**Moderate_Severe in training set**
FCFP_8	159404153	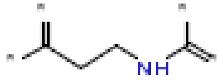	0.326	15 out of 16

FCFP_8	-1272709286	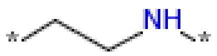	0.287	234 out of 266
FCFP_8	1673997923	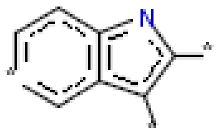	0.258	2 out of 2

Top Features for Mild

Fingerprint	Bit	Structure/Smiles	Score	Moderate_Severe in training set
FCFP_8	907036844	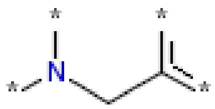	-0.598	1 out of 4
FCFP_8	-146015125	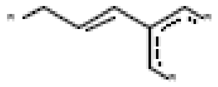	-0.506	0 out of 1
FCFP_8	599018404	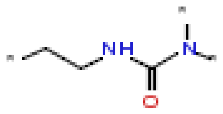	-0.506	0 out of 1

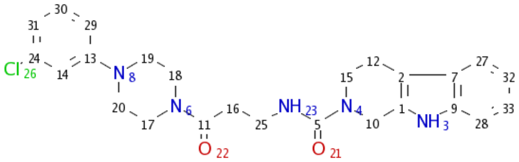

$C_{25}H_{28}ClN_5O_2$
Molecular Weight: 465.97512
ALogP: 3.413
Rotatable Bonds: 4
Acceptors: 3
Donors: 2

Model Prediction

Prediction: Mild
Probability: 0.827
Enrichment: 1.201
Bayesian Score: -0.773

Prediction: Positive if the Bayesian score is above the estimated best cutoff value.
Probability: The esimated probability that the sample is in the category.
Enrichment: An estimate of enrichment, that is, the increased likelihood (versus random) of this sample being in the category.
Bayesian Score: The standard Laplacian-modified Bayesian score.

Structural Similar Compounds

Name	ETHANOL;2-(P-CHLOROPHENOXY)-	ISOQUINOLINE; 1;2;3;4-TETRAHYDRO-2-OCTANOYL-	ETHANOL; 2;2'-(PHENYLIMINO)DI-
Structure	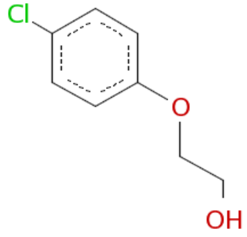	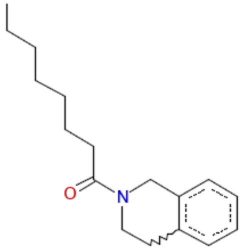	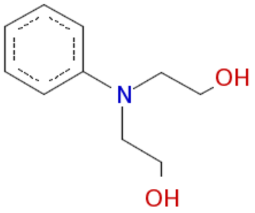
Actual Endpoint	Moderate_Severe	Mild	Moderate_Severe
Predicted Endpoint	Moderate_Severe	Mild	Moderate_Severe
Similarity	0.268	0.267	0.264
Reference	28ZPAK-;81;72	NTIS** AD-A042-527	UCDS** 6/13/60

Model Applicability

Missing features are fingerprint features in the query molecule not found in the training set.

1. Missing Feature: FCFP_8 203707511 [*]C[c]1:n:[*]:[*]:[c]:1[*]

Feature Contribution

Top features for Moderate_Severe				
**Fingerprint**	**Bit**	**Structure/Smiles**	**Score**	**Moderate_Severe in training set**
FCFP_8	159404153	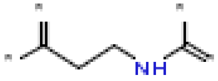	0.326	15 out of 16

FCFP_8	-745491832	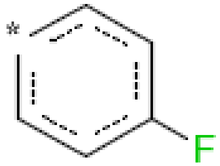	0.305	29 out of 32
FCFP_8	-1272709286	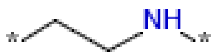	0.287	234 out of 266

Top Features for Mild

Fingerprint	Bit	Structure/Smiles	Score	Moderate_Severe in training set
FCFP_8	907036844	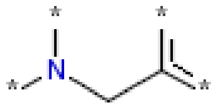	-0.598	1 out of 4
FCFP_8	599018404	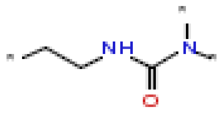	-0.506	0 out of 1
FCFP_8	-2005207466	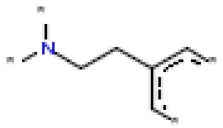	-0.506	0 out of 1

MOST ACTIVE.mol

TOPKAT_Ocular_Irritancy_Mild_vs_Moderate_Severe

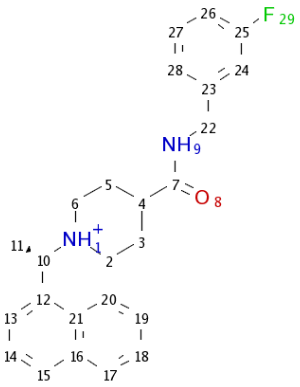

$C_{25}H_{28}FN_2O$
Molecular Weight: 391.50102
ALogP: 3.098
Rotatable Bonds: 5
Acceptors: 1
Donors: 2

Model Prediction

Prediction: Mild
Probability: 0.764
Enrichment: 1.108
Bayesian Score: -2.447

Prediction: Positive if the Bayesian score is above the estimated best cutoff value.
Probability: The esimated probability that the sample is in the category.
Enrichment: An estimate of enrichment, that is, the increased likelihood (versus random) of this sample being in the category.
Bayesian Score: The standard Laplacian-modified Bayesian score.

Structural Similar Compounds

Name	7H-Benz(de)anthracen-7-one; 3;9-dibromo-	Indole-2;3-dione; 5-bromo-	SULFOXIDE; p-CHLOROPHENYL METHYL
Structure	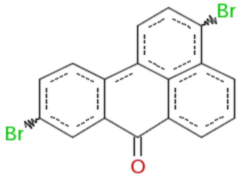	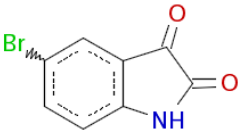	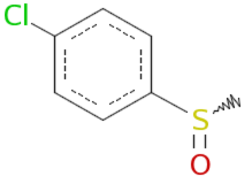
Actual Endpoint	Mild	Moderate_Severe	Moderate_Severe
Predicted Endpoint	Mild	Moderate_Severe	Moderate_Severe
Similarity	0.320	0.319	0.310
Reference	Prehled Prumyslove Toxikologie; Organicke Latky; Marhold; J. pp 567;86	Prehled Prumyslove Toxikologie; Organicke Latky; Marhold; J. -;821;86	NTIS** AD-A082-824

Model Applicability

Missing features are fingerprint features in the query molecule not found in the training set.

1. Missing Feature: FCFP_8 10 [*][NH+]([*])[*]
2. Missing Feature: FCFP_8 -1853714334 [*]C[NH+](C[*])C[*]
3. Missing Feature: FCFP_8 1155241219 [*]CC[NH+]([*])[*]
4. Missing Feature: FCFP_8 -680623486 [*][NH+]([*])[C@H](C)[c](:[*]):[*]

Feature Contribution

Top features for Moderate_Severe

Fingerprint	Bit	Structure/Smiles	Score	Moderate_Severe in training set
FCFP_8	907096426	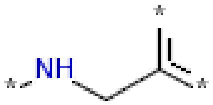	0.334	5 out of 5

FCFP_8	-745491832	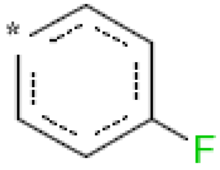	0.305	29 out of 32
FCFP_8	427906732	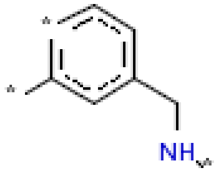	0.295	3 out of 3
**Top Features for Mild**				
**Fingerprint**	**Bit**	**Structure/Smiles**	**Score**	**Moderate_Severe in training set**
FCFP_8	-1700637232	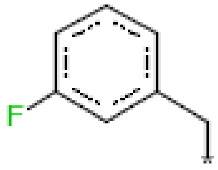	-1.334	1 out of 10
FCFP_8	-384723139	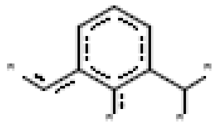	-0.418	4 out of 10
FCFP_8	-1320007763	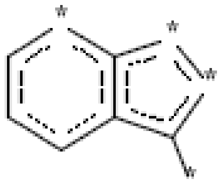	-0.314	19 out of 40

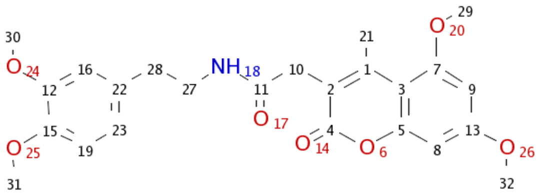

$C_{24}H_{27}NO_7$
Molecular Weight: 441.47368
ALogP: 3.388
Rotatable Bonds: 9
Acceptors: 7
Donors: 1

Model Prediction

Prediction: Moderate
Probability: 0.633
Enrichment: 1.020
Bayesian Score: -1.689

Prediction: Positive if the Bayesian score is above the estimated best cutoff value.
Probability: The esimated probability that the sample is in the category.
Enrichment: An estimate of enrichment, that is, the increased likelihood (versus random) of this sample being in the category.
Bayesian Score: The standard Laplacian-modified Bayesian score.

Structural Similar Compounds

Name	NPE 12	NPE 10	NPE 9
Structure	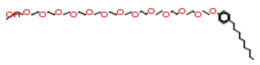	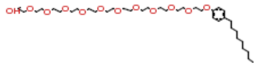	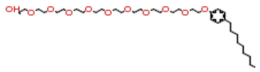
Actual Endpoint	Severe	Severe	Moderate
Predicted Endpoint	Severe	Severe	Severe
Similarity	0.370	0.370	0.370
Reference	In: Environ. and Human Safety of Major Surfactants; Talmage; S; 1994 Lewis Publi	In: Environ. and Human Safety of Major Surfactants; Talmage; S; 1994 Lewis Publi	In: Environ. and Human Safety of Major Surfactants; Talmage; S; 1994 Lewis Publi

Model Applicability

Missing features are fingerprint features in the query molecule not found in the training set.

1. All descriptors are in range.

Feature Contribution

Top features for Severe				
Fingerprint	Bit	Structure/Smiles	Score	Severe in training set
SCFP_10	125999298	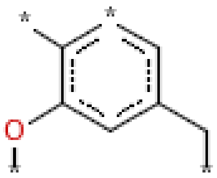	0.425	7 out of 7

SCFP_10	130348166	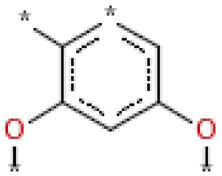	0.381	4 out of 4
SCFP_10	-1272709286	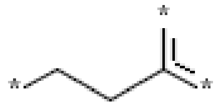	0.237	24 out of 31
**Top Features for Moderate**				
**Fingerprint**	**Bit**	**Structure/Smiles**	**Score**	**Severe in training set**
SCFP_10	2005026407	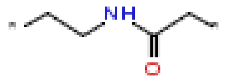	-0.792	0 out of 2
SCFP_10	-587569116	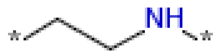	-0.613	11 out of 35
SCFP_10	1256995004	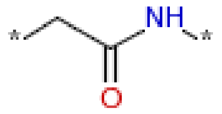	-0.477	12 out of 33

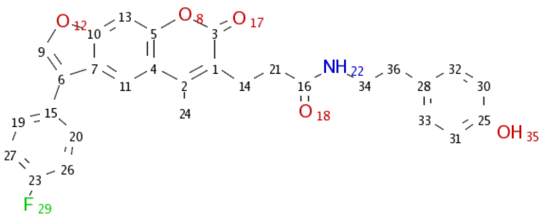

$C_{29}H_{24}FNO_5$

Molecular Weight: 485.50296

ALogP: 5.695

Rotatable Bonds: 7

Acceptors: 4

Donors: 2

Structural Similar Compounds

Name	NONYLPHENOL	CYCLOHEXENYL-4-PHENOL	RESORCINOL; MONOACETATE
Structure	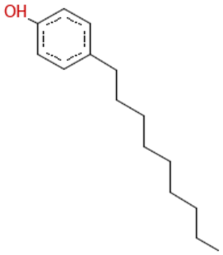	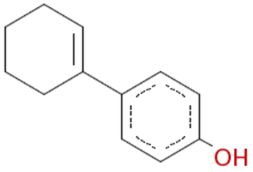	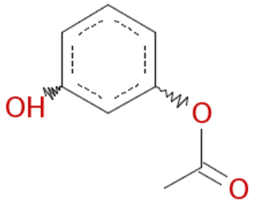
Actual Endpoint	Severe	Severe	Severe
Predicted Endpoint	Severe	Severe	Severe
Similarity	0.291	0.259	0.254
Reference	In: Environ. and Human Safety of Major Surfactants; Talmage; S; 1994 Lewis Publi	AIHAAP 30;470;69	JAPMA8 46;185;57

Model Prediction

Prediction: Moderate

Probability: 0.534

Enrichment: 0.861

Bayesian Score: -3.393

Prediction: Positive if the Bayesian score is above the estimated best cutoff value.
Probability: The esimated probability that the sample is in the category.
Enrichment: An estimate of enrichment, that is, the increased likelihood (versus random) of this sample being in the category.
Bayesian Score: The standard Laplacian-modified Bayesian score.

Model Applicability

Missing features are fingerprint features in the query molecule not found in the training set.

1. All descriptors are in range.

Feature Contribution

Top features for Severe

Fingerprint	Bit	Structure/Smiles	Score	Severe in training set
SCFP_10	1689050571	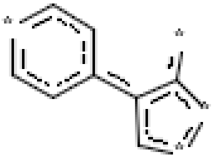	0.306	2 out of 2

SCFP_10	611156666	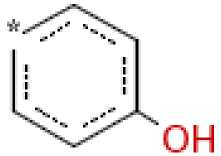	0.303	20 out of 24
SCFP_10	-1850560426	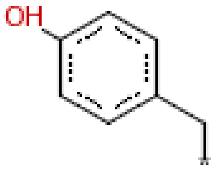	0.291	6 out of 7
**Top Features for Moderate**				
**Fingerprint**	**Bit**	**Structure/Smiles**	**Score**	**Severe in training set**
SCFP_10	2005026407	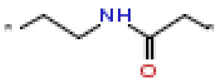	-0.792	0 out of 2
SCFP_10	-587569116	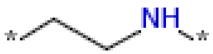	-0.613	11 out of 35
SCFP_10	-496111702	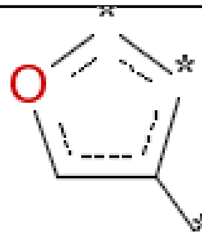	-0.536	1 out of 4

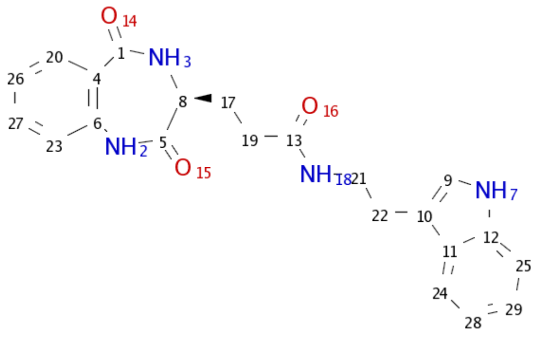

$C_{22}H_{22}N_4O_3$
Molecular Weight: 390.43508
ALogP: 1.807
Rotatable Bonds: 6
Acceptors: 3
Donors: 4

Structural Similar Compounds

Name	Anthranilic acid; N-methyl-	1;5-NAPHTHALENEDISULFONIC ACID;2-AMINO-	INDOLE
Structure	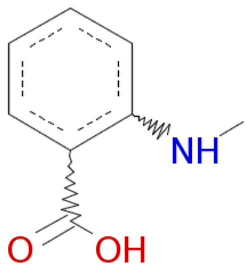	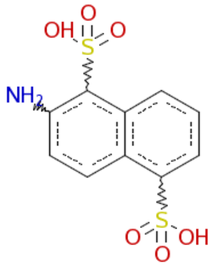	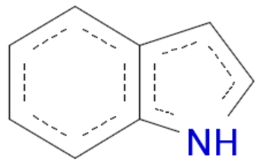
Actual Endpoint	Moderate	Moderate	Severe
Predicted Endpoint	Moderate	Moderate	Severe
Similarity	0.265	0.265	0.261
Reference	Prehled Prumyslove Toxikologie; Organicke Latky; Marhold; J. -;741;86	28ZPAK-;188;72	AIHAAP 23;95;62

Model Prediction

Prediction: Moderate
Probability: 0.438
Enrichment: 0.706
Bayesian Score: -4.739

Prediction: Positive if the Bayesian score is above the estimated best cutoff value.
Probability: The esimated probability that the sample is in the category.
Enrichment: An estimate of enrichment, that is, the increased likelihood (versus random) of this sample being in the category.
Bayesian Score: The standard Laplacian-modified Bayesian score.

Model Applicability

Missing features are fingerprint features in the query molecule not found in the training set.

1. Missing Feature: SCFP_10 -1946889102 [*]C[C@H](N[*])C(=[*])[*]

Feature Contribution

Top features for Severe				
Fingerprint	Bit	Structure/Smiles	Score	Severe in training set
SCFP_10	-496201075	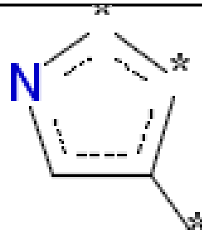	0.384	12 out of 13

SCFP_10	-673674794	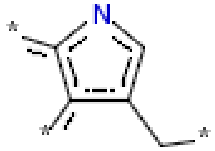	0.381	4 out of 4
SCFP_10	1188101983	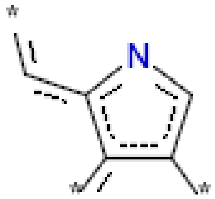	0.306	2 out of 2
**Top Features for Moderate**				
**Fingerprint**	**Bit**	**Structure/Smiles**	**Score**	**Severe in training set**
SCFP_10	-1072897324	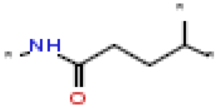	-0.795	3 out of 13
SCFP_10	2005026407	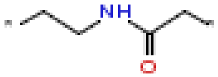	-0.792	0 out of 2
SCFP_10	1655488245	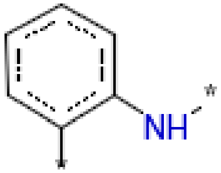	-0.792	0 out of 2

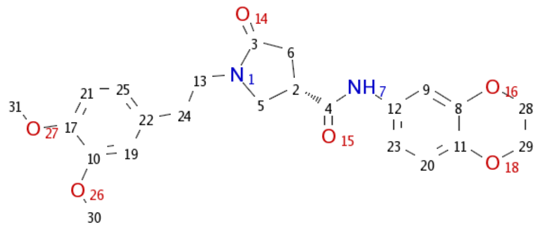

$C_{23}H_{26}N_2O_6$
Molecular Weight: 426.46234
ALogP: 1.967
Rotatable Bonds: 7
Acceptors: 6
Donors: 1

Model Prediction

Prediction: Moderate
Probability: 0.454
Enrichment: 0.733
Bayesian Score: -4.521

Prediction: Positive if the Bayesian score is above the estimated best cutoff value.
Probability: The esimated probability that the sample is in the category.
Enrichment: An estimate of enrichment, that is, the increased likelihood (versus random) of this sample being in the category.
Bayesian Score: The standard Laplacian-modified Bayesian score.

Structural Similar Compounds

Name	p-Acetophenetidide; 3'-(bis(2-hydroxyethyl)amino)-	P-ACETOPHENETIDIDE;3-NITRO-	C12 APE 11
Structure	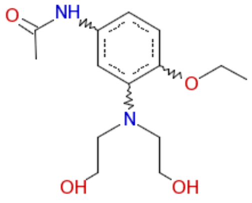	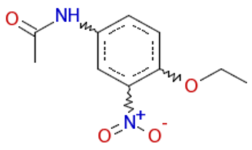	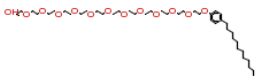
Actual Endpoint	Moderate	Moderate	Severe
Predicted Endpoint	Moderate	Moderate	Severe
Similarity	0.444	0.409	0.370
Reference	Prehled Prumyslove Toxikologie; Organicke Latky; Marhold; J. -;645;86	28ZPAK-;115;72	In: Environ. and Human Safety of Major Surfactants; Talmage; S; 1994 Lewis Publi

Model Applicability

Missing features are fingerprint features in the query molecule not found in the training set.

1. All descriptors are in range.

Feature Contribution

Top features for Severe				
Fingerprint	Bit	Structure/Smiles	Score	Severe in training set
SCFP_10	125999298	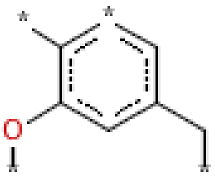	0.425	7 out of 7

SCFP_10	-1272709286	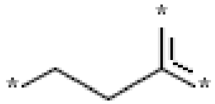	0.237	24 out of 31
SCFP_10	1242547645	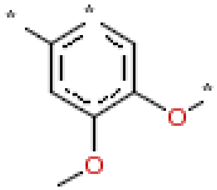	0.220	1 out of 1
**Top Features for Moderate**				
**Fingerprint**	**Bit**	**Structure/Smiles**	**Score**	**Severe in training set**
SCFP_10	929361185	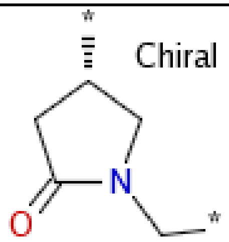	-1.012	2 out of 12
SCFP_10	-2103400817	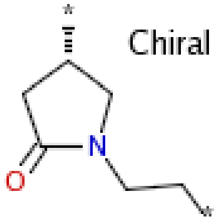	-0.936	2 out of 11
SCFP_10	-531283893	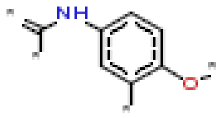	-0.792	0 out of 2

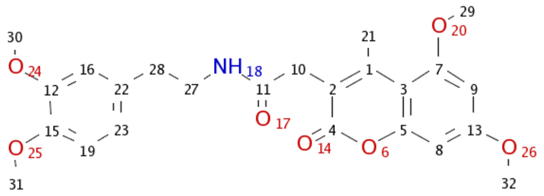

$C_{24}H_{27}NO_7$
Molecular Weight: 441.47368
ALogP: 3.388
Rotatable Bonds: 9
Acceptors: 7
Donors: 1

Structural Similar Compounds

Name	NPE 2	NPE 13	C12 APE 11
Structure	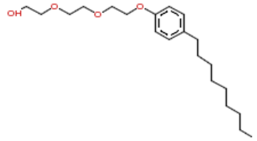	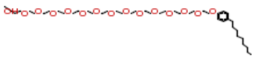	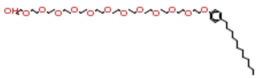
Actual Endpoint	Non-Irritant	Irritant	Irritant
Predicted Endpoint	Irritant	Irritant	Irritant
Similarity	0.370	0.370	0.370
Reference	In: Environ. and Human Safety of Major Surfactants; Talmage; S; 1994 Lewis Publi	In: Environ. and Human Safety of Major Surfactants; Talmage; S; 1994 Lewis Publi	In: Environ. and Human Safety of Major Surfactants; Talmage; S; 1994 Lewis Publi

Model Prediction

Prediction: Irritant
Probability: 0.999
Enrichment: 1.175
Bayesian Score: 0.888

Prediction: Positive if the Bayesian score is above the estimated best cutoff value.
Probability: The esimated probability that the sample is in the category.
Enrichment: An estimate of enrichment, that is, the increased likelihood (versus random) of this sample being in the category.
Bayesian Score: The standard Laplacian-modified Bayesian score.

Model Applicability

Missing features are fingerprint features in the query molecule not found in the training set.

1. Missing Feature: ECFP_10 1792159373 [*]C(=C(C)[c](:[*]):[*])[*]
2. Missing Feature: ECFP_10 -770854792 [*]CC(=C([*])[*])C(=[*])[*]

Feature Contribution

Top features for Irritant

Fingerprint	Bit	Structure/Smiles	Score	Irritant in training set
ECFP_10	2077607946	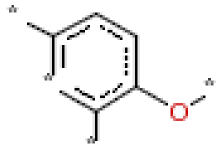	0.195	14 out of 14

ECFP_10	-560785749	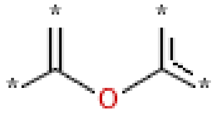	0.190	10 out of 10
ECFP_10	1680623188	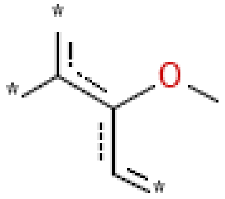	0.188	9 out of 9

Top Features for Non-Irritant

Fingerprint	Bit	Structure/Smiles	Score	Irritant in training set
ECFP_10	2055803015	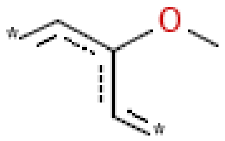	-0.323	5 out of 9
ECFP_10	-570915357	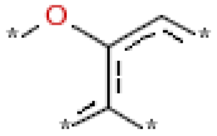	-0.270	1 out of 2
ECFP_10	1573945311	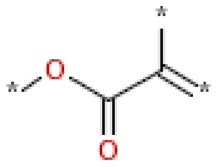	-0.270	1 out of 2

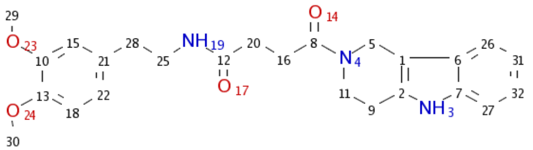

$C_{25}H_{29}N_3O_4$
Molecular Weight: 435.51546
ALogP: 2.831
Rotatable Bonds: 8
Acceptors: 4
Donors: 2

Model Prediction

Prediction: Irritant
Probability: 1.000
Enrichment: 1.175
Bayesian Score: 2.582

Prediction: Positive if the Bayesian score is above the estimated best cutoff value.
Probability: The esimated probability that the sample is in the category.
Enrichment: An estimate of enrichment, that is, the increased likelihood (versus random) of this sample being in the category.
Bayesian Score: The standard Laplacian-modified Bayesian score.

Structural Similar Compounds

Name	ISOQUINOLINE; 1;2;3;4-TETRAHYDRO-2-OCTANOYL-	CARBAMIC ACID; METHYL-; 1-(5;6;7;8-TETRAHYDRO)NAPHTHYL ESTER	QUINOLINE; 1;2;3;4-TETRAHYDRO-1-(2-METHYL-1-OXOPENTYL)-
Structure	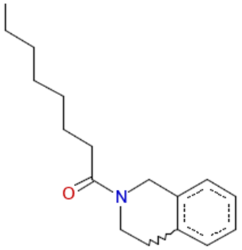	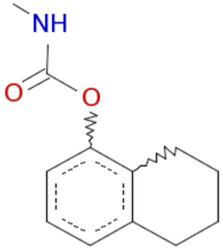	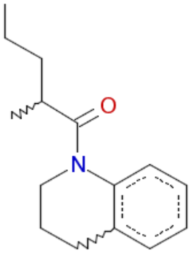
Actual Endpoint	Irritant	Irritant	Irritant
Predicted Endpoint	Irritant	Irritant	Irritant
Similarity	0.358	0.291	0.281
Reference	NTIS** AD-A042-527	28ZPAK-;163;72	NTIS** AD-A056-106

Model Applicability

Missing features are fingerprint features in the query molecule not found in the training set.

1. Missing Feature: ECFP_10 979542842 [*]C[c]1:n:[*]:[*]:[c]:1[*]

Feature Contribution

Top features for Irritant				
Fingerprint	Bit	Structure/Smiles	Score	Irritant in training set
ECFP_10	2077607946	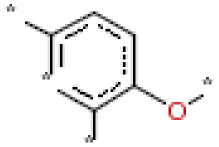	0.195	14 out of 14

ECFP_10	-1102925512	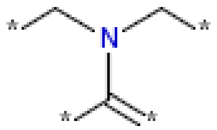	0.194	13 out of 13
ECFP_10	1680623188	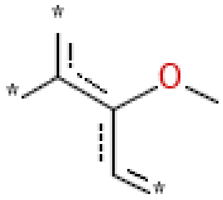	0.188	9 out of 9

Top Features for Non-Irritant

Fingerprint	Bit	Structure/Smiles	Score	Irritant in training set
ECFP_10	-1789102870	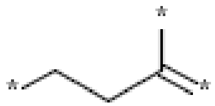	-0.422	48 out of 91
ECFP_10	497523368	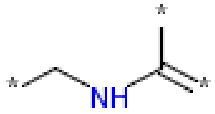	-0.134	2 out of 3
ECFP_10	-1059365320	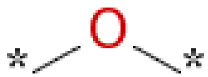	-0.074	421 out of 560

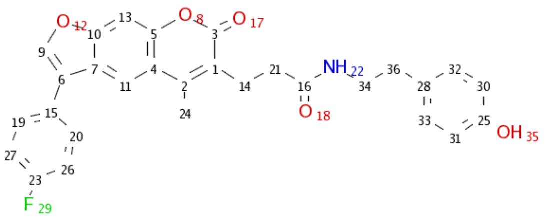

$C_{29}H_{24}FNO_5$
Molecular Weight: 485.50296
ALogP: 5.695
Rotatable Bonds: 7
Acceptors: 4
Donors: 2

Model Prediction

Prediction: Irritant

Probability: 0.999

Enrichment: 1.175

Bayesian Score: 1.400

Prediction: Positive if the Bayesian score is above the estimated best cutoff value.
Probability: The esimated probability that the sample is in the category.
Enrichment: An estimate of enrichment, that is, the increased likelihood (versus random) of this sample being in the category.
Bayesian Score: The standard Laplacian-modified Bayesian score.

Structural Similar Compounds

Name	NONYLPHENOL	ACETIC ACID; 2-CHLORO-5-NITROPHENYL ESTER	CYCLOHEXENYL-4-PHENOL
Structure	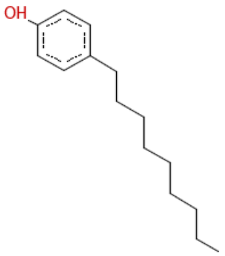	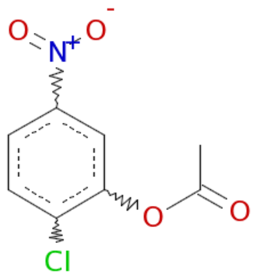	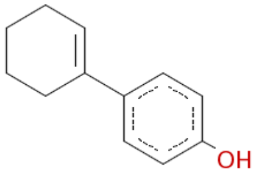
Actual Endpoint	Irritant	Irritant	Irritant
Predicted Endpoint	Irritant	Irritant	Irritant
Similarity	0.291	0.262	0.259
Reference	In: Environ. and Human Safety of Major Surfactants; Talmage; S; 1994 Lewis Publi	28ZPAK-;92;72	AIHAAP 30;470;69

Model Applicability

Missing features are fingerprint features in the query molecule not found in the training set.

1. Missing Feature: ECFP_10 -770854792 [*]CC(=C([*])[*])C(=[*])[*]
2. Missing Feature: ECFP_10 1792159373 [*]C(=C(C)[c](:[*]):[*])[*]
3. Missing Feature: ECFP_10 -785659985 [*][c]1:[*]:[*]:o:c:1

Feature Contribution

Top features for Irritant				
Fingerprint	Bit	Structure/Smiles	Score	Irritant in training set
ECFP_10	-1046436026	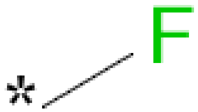	0.197	16 out of 16

ECFP_10	-560785749	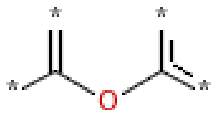	0.190	10 out of 10
ECFP_10	1731843802	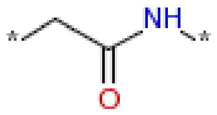	0.182	7 out of 7

Top Features for Non-Irritant

Fingerprint	Bit	Structure/Smiles	Score	Irritant in training set
ECFP_10	-1789102870	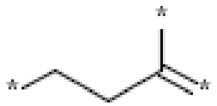	-0.422	48 out of 91
ECFP_10	1573945311	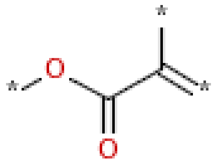	-0.270	1 out of 2
ECFP_10	-570915357	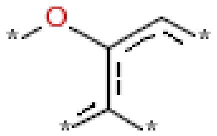	-0.270	1 out of 2

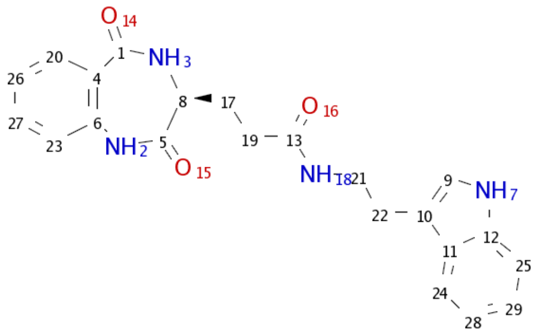

$C_{22}H_{22}N_4O_3$

Molecular Weight: 390.43508

ALogP: 1.807

Rotatable Bonds: 6

Acceptors: 3

Donors: 4

Model Prediction

Prediction: Irritant

Probability: 0.999

Enrichment: 1.174

Bayesian Score: 0.594

Prediction: Positive if the Bayesian score is above the estimated best cutoff value.
Probability: The esimated probability that the sample is in the category.
Enrichment: An estimate of enrichment, that is, the increased likelihood (versus random) of this sample being in the category.
Bayesian Score: The standard Laplacian-modified Bayesian score.

Structural Similar Compounds

Name	ACETANILIDE; 4'-(2-HYDROXYETHYLSULFONYL)-	2-NAPHTHALENE SULFONIC ACID;5-AMINO-	1-AMINO-4-BENZOYLAMINO-ANTHRAQUINONE
Structure	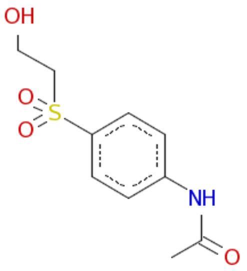	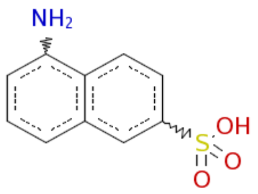	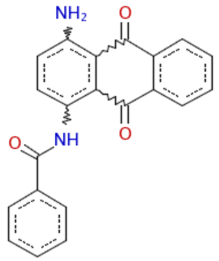
Actual Endpoint	Irritant	Irritant	Irritant
Predicted Endpoint	Irritant	Irritant	Irritant
Similarity	0.288	0.280	0.269
Reference	28ZPAK-;201;72	28ZPAK-;187;72	28ZPAK-;124;72

Model Applicability

Missing features are fingerprint features in the query molecule not found in the training set.

1. Missing Feature: ECFP_10 -1693599735 [*]C([*])NC(=[*])[*]
2. Missing Feature: ECFP_10 -867777309 [*]NC(=O)C([*])[*]
3. Missing Feature: ECFP_10 -410102202 [*]C[C@H](N[*])C(=[*])[*]
4. Missing Feature: ECFP_10 -1020449580 [*][c]1:[*]:[*]:n:c:1

Feature Contribution

Top features for Irritant				
**Fingerprint**	**Bit**	**Structure/Smiles**	**Score**	**Irritant in training set**
ECFP_10	1731843802	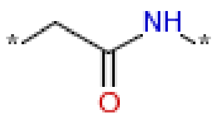	0.182	7 out of 7

ECFP_10	-2024509555	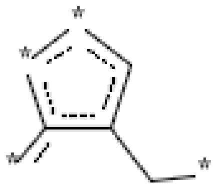	0.182	7 out of 7
ECFP_10	-280629989	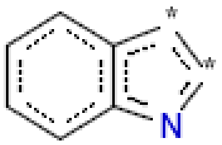	0.154	3 out of 3

Top Features for Non-Irritant

Fingerprint	Bit	Structure/Smiles	Score	Irritant in training set
ECFP_10	1335833675	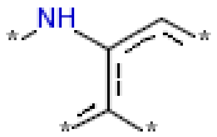	-0.539	1 out of 3
ECFP_10	-1789102870	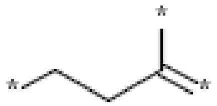	-0.422	48 out of 91
ECFP_10	-1789838820	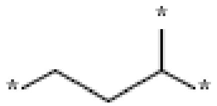	-0.288	4 out of 7

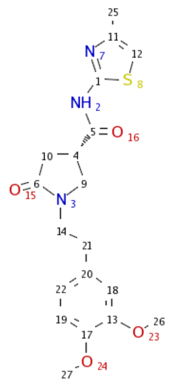

$C_{19}H_{23}N_{3}O_{4}S$
Molecular Weight: 389.46862
ALogP: 1.461
Rotatable Bonds: 7
Acceptors: 5
Donors: 1

Structural Similar Compounds

Name	ISOQUINOLINE; 1;2;3;4-TETRAHYDRO-2-OCTANOYL-	NPE 4	NPE 12
Structure	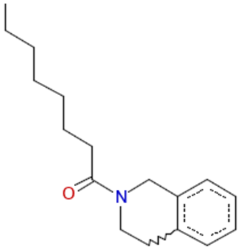	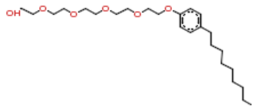	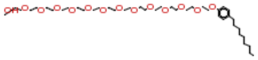
Actual Endpoint	Irritant	Irritant	Irritant
Predicted Endpoint	Irritant	Irritant	Irritant
Similarity	0.340	0.327	0.327
Reference	NTIS** AD-A042-527	In: Environ. and Human Safety of Major Surfactants; Talmage; S; 1994 Lewis Publi	In: Environ. and Human Safety of Major Surfactants; Talmage; S; 1994 Lewis Publi

Model Prediction

Prediction: Irritant
Probability: 1.000
Enrichment: 1.176
Bayesian Score: 3.189

Prediction: Positive if the Bayesian score is above the estimated best cutoff value.
Probability: The esimated probability that the sample is in the category.
Enrichment: An estimate of enrichment, that is, the increased likelihood (versus random) of this sample being in the category.
Bayesian Score: The standard Laplacian-modified Bayesian score.

Model Applicability

Missing features are fingerprint features in the query molecule not found in the training set.

1. Missing Feature: ECFP_10 -1596132236 [*]N[c]1:n:[*]:[*]:s:1
2. Missing Feature: ECFP_10 -81134287 [*]NC(=O)C([*])[*]
3. Missing Feature: ECFP_10 -1426923364 [*][c]1:[*]:[*]:c:s:1
4. Missing Feature: ECFP_10 -224638920 [*][c]1:[*]:[*]:s:c:1

Feature Contribution

Top features for Irritant				
**Fingerprint**	**Bit**	**Structure/Smiles**	**Score**	**Irritant in training set**
ECFP_10	834876373	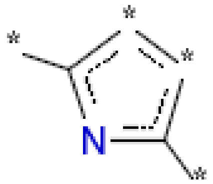	0.203	28 out of 28

ECFP_10	2077607946	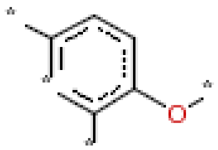	0.195	14 out of 14
ECFP_10	655739385	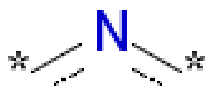	0.188	53 out of 54
**Top Features for Non-Irritant**				
**Fingerprint**	**Bit**	**Structure/Smiles**	**Score**	**Irritant in training set**
ECFP_10	-1059365320	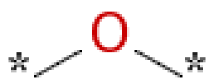	-0.074	421 out of 560
ECFP_10	2099970318	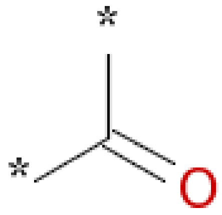	-0.072	297 out of 394
ECFP_10	-1100000244	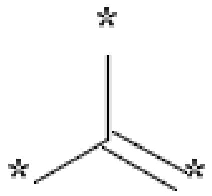	-0.061	327 out of 429

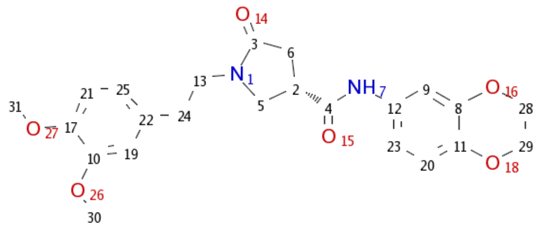

$C_{23}H_{26}N_2O_6$
Molecular Weight: 426.46234
ALogP: 1.967
Rotatable Bonds: 7
Acceptors: 6
Donors: 1

Structural Similar Compounds

Name	p-Acetophenetidide; 3'-(bis(2-hydroxyethyl)amino)-	P-ACETOPHENETIDIDE;3-NITRO-	ACETANILIDE; 3'-AMINO-4'-ETHOXY-
Structure	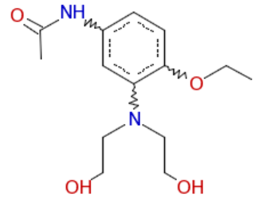	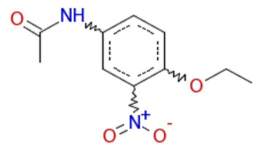	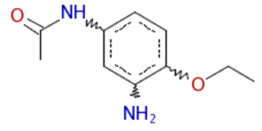
Actual Endpoint	Irritant	Irritant	Irritant
Predicted Endpoint	Irritant	Irritant	Irritant
Similarity	0.444	0.409	0.395
Reference	Prehled Prumyslove Toxikologie; Organicke Latky; Marhold; J. -;645;86	28ZPAK-;115;72	28ZPAK 115;72

Model Prediction

Prediction: Irritant
Probability: 1.000
Enrichment: 1.175
Bayesian Score: 2.220

Prediction: Positive if the Bayesian score is above the estimated best cutoff value.
Probability: The esimated probability that the sample is in the category.
Enrichment: An estimate of enrichment, that is, the increased likelihood (versus random) of this sample being in the category.
Bayesian Score: The standard Laplacian-modified Bayesian score.

Model Applicability

Missing features are fingerprint features in the query molecule not found in the training set.

1. Missing Feature: ECFP_10 -81134287 [*]NC(=O)C([*])[*]

Feature Contribution

Top features for Irritant

Fingerprint	Bit	Structure/Smiles	Score	Irritant in training set
ECFP_10	2077607946	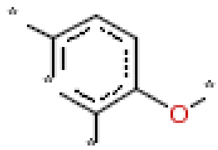	0.195	14 out of 14

ECFP_10	1680623188	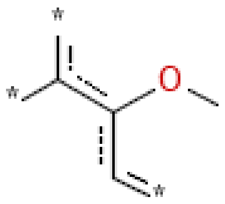	0.188	9 out of 9
ECFP_10	-857146788	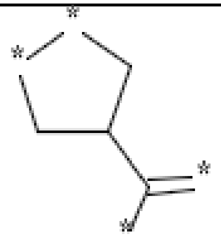	0.178	6 out of 6

Top Features for Non-Irritant

Fingerprint	Bit	Structure/Smiles	Score	Irritant in training set
ECFP_10	561585423	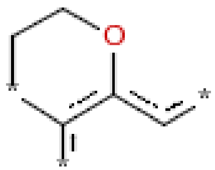	-0.593	0 out of 1
ECFP_10	-1421422591	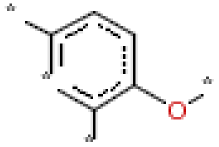	-0.270	1 out of 2
ECFP_10	-570915357	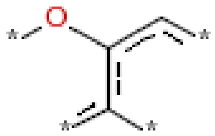	-0.270	1 out of 2

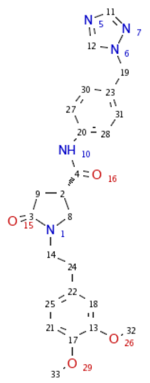

$C_{24}H_{27}N_5O_4$
Molecular Weight: 449.50228
ALogP: 1.731
Rotatable Bonds: 9
Acceptors: 6
Donors: 1

Structural Similar Compounds

Name	p-Acetophenetidide; 3'-(bis(2-hydroxyethyl)amino)-	ISOQUINOLINE; 1;2;3;4-TETRAHYDRO-2-OCTANOYL-	0-ANISIDINE;ACETOACETYL
Structure	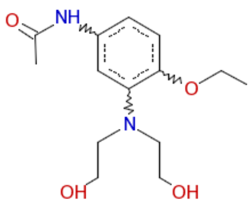	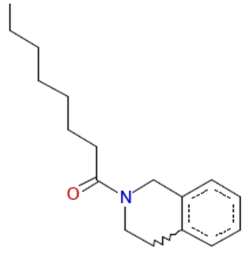	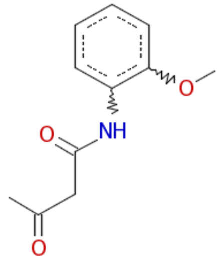
Actual Endpoint	Irritant	Irritant	Irritant
Predicted Endpoint	Irritant	Irritant	Irritant
Similarity	0.316	0.315	0.315
Reference	Prehled Prumyslove Toxikologie; Organicke Latky; Marhold; J. -;645;86	NTIS** AD-A042-527	28ZPAK-;116;72

Model Prediction

Prediction: Irritant

Probability: 1.000

Enrichment: 1.176

Bayesian Score: 3.257

Prediction: Positive if the Bayesian score is above the estimated best cutoff value.
Probability: The esimated probability that the sample is in the category.
Enrichment: An estimate of enrichment, that is, the increased likelihood (versus random) of this sample being in the category.
Bayesian Score: The standard Laplacian-modified Bayesian score.

Model Applicability

Missing features are fingerprint features in the query molecule not found in the training set.

1. Missing Feature: ECFP_10 -81134287 [*]NC(=O)C([*])[*]
2. Missing Feature: ECFP_10 -285549720 [*]Cn1:c:[*]:[*]:n:1
3. Missing Feature: ECFP_10 -676555381 [*]n1:[*]:[*]:c:n:1
4. Missing Feature: ECFP_10 -710237522 [*]1:[*]:n:c:n:1
5. Missing Feature: ECFP_10 -708878603 [*]n1:[*]:[*]:n:c:1
6. Missing Feature: ECFP_10 772089587 [*]:[c](:[*])Cn(:[*]):[*]

Feature Contribution

Top features for Irritant

Fingerprint	Bit	Structure/Smiles	Score	Irritant in training set

ECFP_10	2077607946	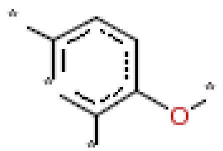	0.195	14 out of 14
ECFP_10	655739385	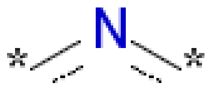	0.188	53 out of 54
ECFP_10	1680623188	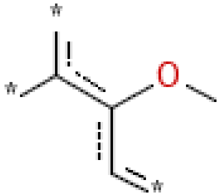	0.188	9 out of 9
**Top Features for Non-Irritant**				
**Fingerprint**	**Bit**	**Structure/Smiles**	**Score**	**Irritant in training set**
ECFP_10	-1059365320	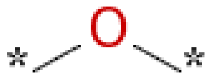	-0.074	421 out of 560
ECFP_10	2099970318	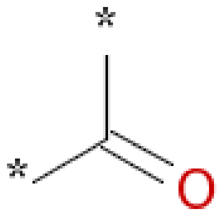	-0.072	297 out of 394

ECFP_10	-1100000244	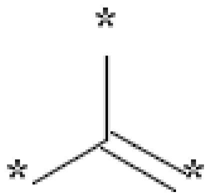	-0.061	327 out of 429

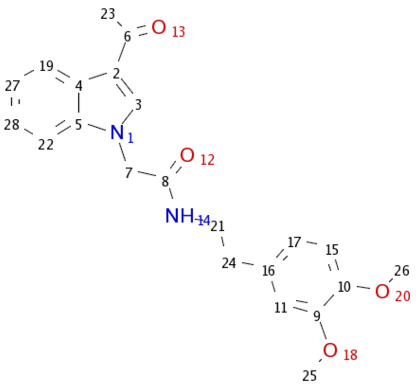

$C_{22}H_{24}N_2O_4$
Molecular Weight: 380.43696
ALogP: 3.001
Rotatable Bonds: 8
Acceptors: 4
Donors: 1

Model Prediction

Prediction: Irritant
Probability: 0.999
Enrichment: 1.175
Bayesian Score: 1.059

Prediction: Positive if the Bayesian score is above the estimated best cutoff value.
Probability: The esimated probability that the sample is in the category.
Enrichment: An estimate of enrichment, that is, the increased likelihood (versus random) of this sample being in the category.
Bayesian Score: The standard Laplacian-modified Bayesian score.

Structural Similar Compounds

Name	7H-BENZIMIDAZO(2;1-a)BENZ(de)ISOQUINOLIN-7-ONE; 10-METHOXY-	CARBAMIC ACID; METHYL-; 1-(5;6;7;8-TETRAHYDRO)NAPHTHYL ESTER	ANTHRAQUINONE;1;5-DIMETHOXY
Structure	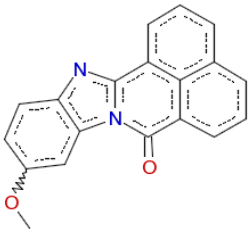	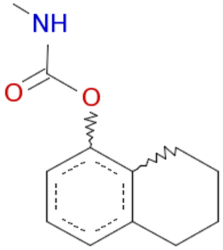	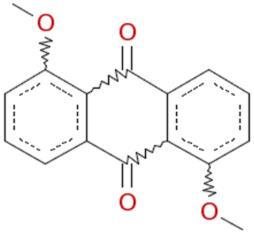
Actual Endpoint	Irritant	Irritant	Irritant
Predicted Endpoint	Irritant	Irritant	Irritant
Similarity	0.333	0.327	0.311
Reference	28ZPAK 147;72	28ZPAK-;163;72	28ZPAK-;113;72

Model Applicability

Missing features are fingerprint features in the query molecule not found in the training set.

1. Missing Feature: ECFP_10 -284945219 [*]Cn1:c:[*]:[*]:[c]:1:[*]
2. Missing Feature: ECFP_10 499043293 [*]C(=[*])Cn(:[*]):[*]

Feature Contribution

Top features for Irritant

Fingerprint	Bit	Structure/Smiles	Score	Irritant in training set
ECFP_10	2077607946	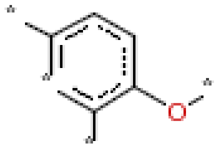	0.195	14 out of 14

ECFP_10	1680623188	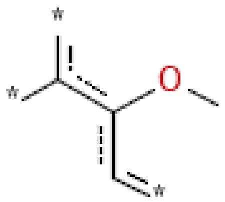	0.188	9 out of 9
ECFP_10	1731843802	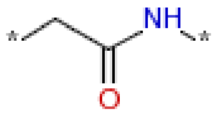	0.182	7 out of 7
**Top Features for Non-Irritant**				
**Fingerprint**	**Bit**	**Structure/Smiles**	**Score**	**Irritant in training set**
ECFP_10	129482634	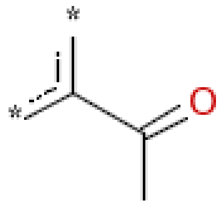	-0.346	2 out of 4
ECFP_10	1337040050	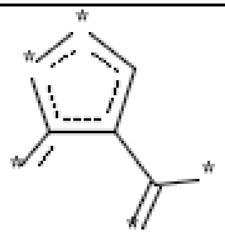	-0.340	17 out of 30
ECFP_10	497523368	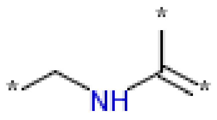	-0.134	2 out of 3

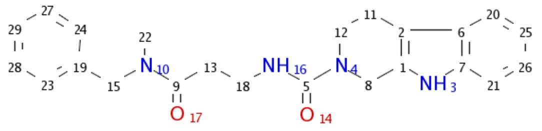

$C_{23}H_{26}N_4O_2$
Molecular Weight: 390.47814
ALogP: 2.702
Rotatable Bonds: 5
Acceptors: 2
Donors: 2

Model Prediction

Prediction: Irritant
Probability: 1.000
Enrichment: 1.175
Bayesian Score: 2.326

Prediction: Positive if the Bayesian score is above the estimated best cutoff value.
Probability: The esimated probability that the sample is in the category.
Enrichment: An estimate of enrichment, that is, the increased likelihood (versus random) of this sample being in the category.
Bayesian Score: The standard Laplacian-modified Bayesian score.

Structural Similar Compounds

Name	ISOQUINOLINE; 1;2;3;4-TETRAHYDRO-2-OCTANOYL-	ETHANOL; 2-(BENZYLOXY)-	2-NAPHTHALENE SULFONIC ACID;5-AMINO-
Structure	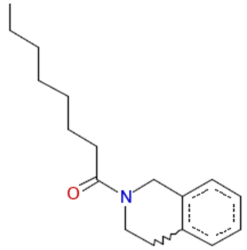	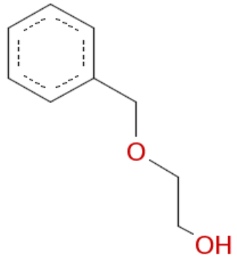	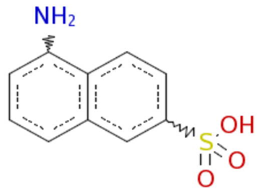
Actual Endpoint	Irritant	Irritant	Irritant
Predicted Endpoint	Irritant	Irritant	Irritant
Similarity	0.367	0.304	0.286
Reference	NTIS** AD-A042-527	AJOPAA 29;1363;46	28ZPAK-;187;72

Model Applicability

Missing features are fingerprint features in the query molecule not found in the training set.

1. Missing Feature: ECFP_10 979542842 [*]C[c]1:n:[*]:[*]:[c]:1[*]
2. Missing Feature: ECFP_10 1657836083 [*]NC(=O)N([*])[*]

Feature Contribution

Top features for Irritant				
**Fingerprint**	**Bit**	**Structure/Smiles**	**Score**	**Irritant in training set**
ECFP_10	865379614	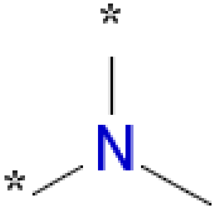	0.204	34 out of 34

ECFP_10	-1102925512	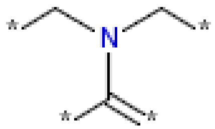	0.194	13 out of 13
ECFP_10	-757679000	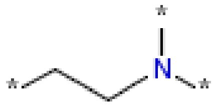	0.186	48 out of 49
**Top Features for Non-Irritant**				
**Fingerprint**	**Bit**	**Structure/Smiles**	**Score**	**Irritant in training set**
ECFP_10	-1789102870	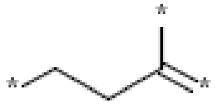	-0.422	48 out of 91
ECFP_10	497523368	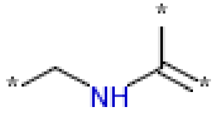	-0.134	2 out of 3
ECFP_10	2099970318	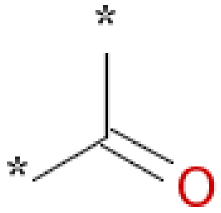	-0.072	297 out of 394

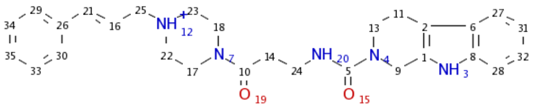

$C_{28}H_{34}N_5O_2$
Molecular Weight: 472.60186
ALogP: 1.567
Rotatable Bonds: 6
Acceptors: 2
Donors: 3

Model Prediction

Prediction: Irritant
Probability: 0.999
Enrichment: 1.175
Bayesian Score: 0.971

Prediction: Positive if the Bayesian score is above the estimated best cutoff value.
Probability: The esimated probability that the sample is in the category.
Enrichment: An estimate of enrichment, that is, the increased likelihood (versus random) of this sample being in the category.
Bayesian Score: The standard Laplacian-modified Bayesian score.

Structural Similar Compounds

Name	ISOQUINOLINE; 1;2;3;4-TETRAHYDRO-2-OCTANOYL-	CARBAMIC ACID; METHYL-; 1-(5;6;7;8-TETRAHYDRO)NAPHTHYL ESTER	ETHANOL; 2-(BENZYLOXY)-
Structure	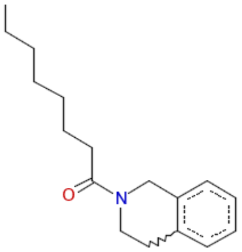	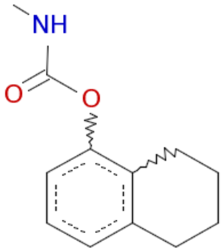	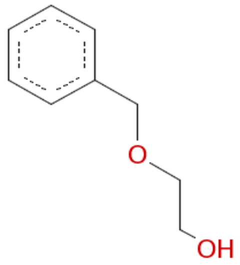
Actual Endpoint	Irritant	Irritant	Irritant
Predicted Endpoint	Irritant	Irritant	Irritant
Similarity	0.283	0.246	0.228
Reference	NTIS** AD-A042-527	28ZPAK-;163;72	AJOPAA 29;1363;46

Model Applicability

Missing features are fingerprint features in the query molecule not found in the training set.

1. Missing Feature: ECFP_10 1976330679 [*][NH+]([*])[*]
2. Missing Feature: ECFP_10 979542842 [*]C[c]1:n:[*]:[*]:[c]:1[*]
3. Missing Feature: ECFP_10 1657836083 [*]NC(=O)N([*])[*]
4. Missing Feature: ECFP_10 1133499173 [*]C[NH+](C[*])C[*]
5. Missing Feature: ECFP_10 -244159614 [*]CC[NH+]([*])[*]
6. Missing Feature: ECFP_10 474483418 [*][NH+]([*])CC=[*]

Feature Contribution

Top features for Irritant

Fingerprint	Bit	Structure/Smiles	Score	Irritant in training set

ECFP_10	-1102925512	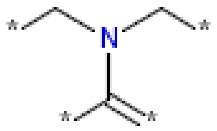	0.194	13 out of 13
ECFP_10	-757679000	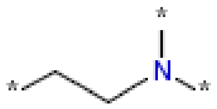	0.186	48 out of 49
ECFP_10	1341750291	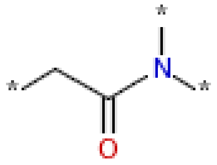	0.178	6 out of 6

Top Features for Non-Irritant

Fingerprint	Bit	Structure/Smiles	Score	Irritant in training set
ECFP_10	312779028	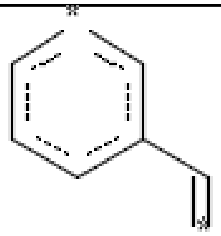	-0.539	1 out of 3
ECFP_10	-1789102870	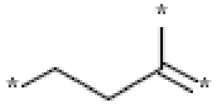	-0.422	48 out of 91

ECFP_10	-97048906	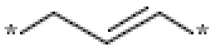	-0.348	10 out of 18

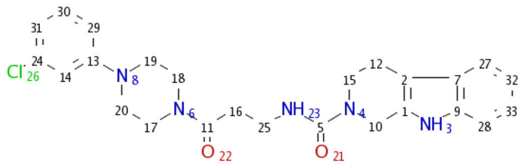

$C_{25}H_{28}ClN_5O_2$
Molecular Weight: 465.97512
ALogP: 3.413
Rotatable Bonds: 4
Acceptors: 3
Donors: 2

Model Prediction

Prediction: Irritant
Probability: 1.000
Enrichment: 1.176
Bayesian Score: 3.078

Prediction: Positive if the Bayesian score is above the estimated best cutoff value.
Probability: The esimated probability that the sample is in the category.
Enrichment: An estimate of enrichment, that is, the increased likelihood (versus random) of this sample being in the category.
Bayesian Score: The standard Laplacian-modified Bayesian score.

Structural Similar Compounds

Name	ETHANOL;2-(P-CHLOROPHENOXY)-	ISOQUINOLINE; 1;2;3;4-TETRAHYDRO-2-OCTANOYL-	ETHANOL; 2;2'-(PHENYLIMINO)DI-
Structure	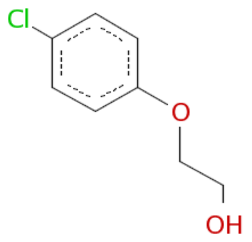	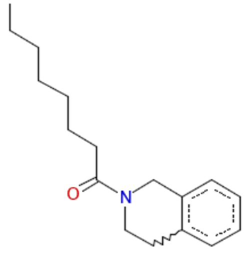	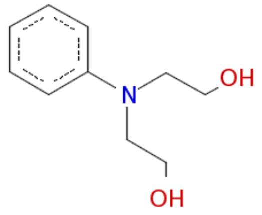
Actual Endpoint	Irritant	Irritant	Irritant
Predicted Endpoint	Irritant	Irritant	Irritant
Similarity	0.268	0.267	0.264
Reference	28ZPAK-;81;72	NTIS** AD-A042-527	UCDS** 6/13/60

Model Applicability

Missing features are fingerprint features in the query molecule not found in the training set.

1. Missing Feature: ECFP_10 979542842 [*]C[c]1:n:[*]:[*]:[c]:1[*]
2. Missing Feature: ECFP_10 1657836083 [*]NC(=O)N([*])[*]

Feature Contribution

Top features for Irritant

Fingerprint	Bit	Structure/Smiles	Score	Irritant in training set
ECFP_10	-1102925512	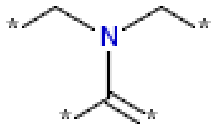	0.194	13 out of 13

ECFP_10	-175021654	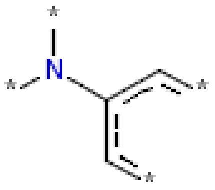	0.190	10 out of 10
ECFP_10	-757679000	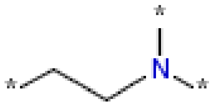	0.186	48 out of 49
**Top Features for Non-Irritant**				
**Fingerprint**	**Bit**	**Structure/Smiles**	**Score**	**Irritant in training set**
ECFP_10	-1789102870	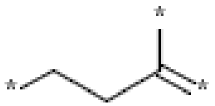	-0.422	48 out of 91
ECFP_10	497523368	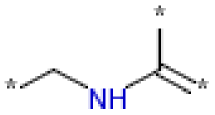	-0.134	2 out of 3
ECFP_10	2099970318	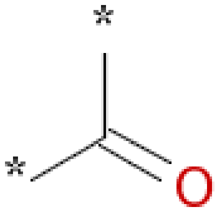	-0.072	297 out of 394

MOST ACTIVE.mol

TOPKAT_Ocular_Irritancy_None_vs_Irritant

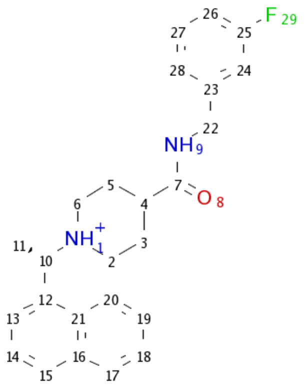

$C_{25}H_{28}FN_2O$

Molecular Weight: 391.50102

ALogP: 3.098

Rotatable Bonds: 5

Acceptors: 1

Donors: 2

Model Prediction

Prediction: Irritant

Probability: 1.000

Enrichment: 1.175

Bayesian Score: 2.233

Prediction: Positive if the Bayesian score is above the estimated best cutoff value.
Probability: The esimated probability that the sample is in the category.
Enrichment: An estimate of enrichment, that is, the increased likelihood (versus random) of this sample being in the category.
Bayesian Score: The standard Laplacian-modified Bayesian score.

Structural Similar Compounds

Name	7H-Benz(de)anthracen-7-one; 3;9-dibromo-	Indole-2;3-dione; 5-bromo-	SULFOXIDE; p-CHLOROPHENYL METHYL
Structure	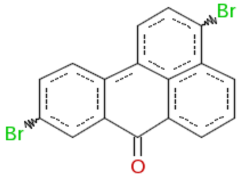	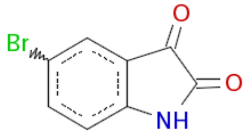	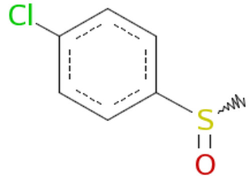
Actual Endpoint	Irritant	Irritant	Irritant
Predicted Endpoint	Irritant	Irritant	Irritant
Similarity	0.320	0.319	0.310
Reference	Prehled Prumyslove Toxikologie; Organicke Latky; Marhold; J. pp 567;86	Prehled Prumyslove Toxikologie; Organicke Latky; Marhold; J. -;821;86	NTIS** AD-A082-824

Model Applicability

Missing features are fingerprint features in the query molecule not found in the training set.

1. Missing Feature: ECFP_10 1976330679 [*][NH+]([*])[*]
2. Missing Feature: ECFP_10 2070272975 [*]C[NH+](C[*])C([*])[*]
3. Missing Feature: ECFP_10 -244159614 [*]CC[NH+]([*])[*]
4. Missing Feature: ECFP_10 -81134287 [*]NC(=O)C([*])[*]
5. Missing Feature: ECFP_10 -1670638661 [*][NH+]([*])[C@H](C)[c](:[*]):[*]
6. Missing Feature: ECFP_10 769925792 [*]NC[c](:[*]):[*]

Feature Contribution

Top features for Irritant				
Fingerprint	Bit	Structure/Smiles	Score	Irritant in training set

ECFP_10	-1046436026	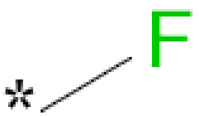	0.197	16 out of 16
ECFP_10	-857146788	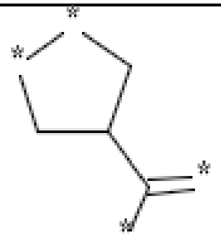	0.178	6 out of 6
ECFP_10	1335340087	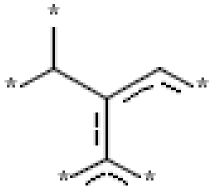	0.165	4 out of 4
**Top Features for Non-Irritant**				
**Fingerprint**	**Bit**	**Structure/Smiles**	**Score**	**Irritant in training set**
ECFP_10	497523368	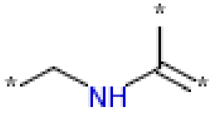	-0.134	2 out of 3
ECFP_10	2099970318	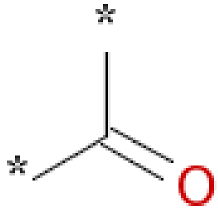	-0.072	297 out of 394

ECFP_10	-1100000244	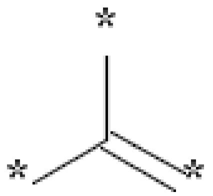	-0.061	327 out of 429

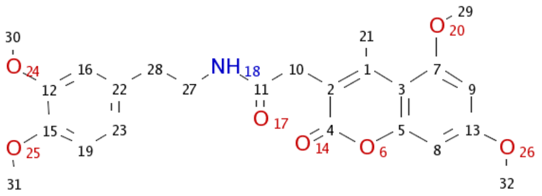

$C_{24}H_{27}NO_7$

Molecular Weight: 441.47368

ALogP: 3.388

Rotatable Bonds: 9

Acceptors: 7

Donors: 1

Model Prediction

Prediction: Non-Carcinogen

Probability: 0.228

Enrichment: 0.707

Bayesian Score: -3.146

Prediction: Positive if the Bayesian score is above the estimated best cutoff value.
Probability: The esimated probability that the sample is in the category.
Enrichment: An estimate of enrichment, that is, the increased likelihood (versus random) of this sample being in the category.
Bayesian Score: The standard Laplacian-modified Bayesian score.

Structural Similar Compounds

Name	Mesuprine	Nabumetone	Atenolol
Structure	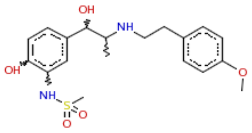	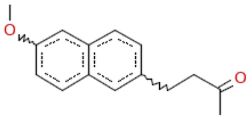	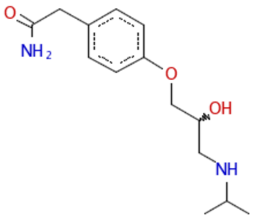
Actual Endpoint	Carcinogen	Non-Carcinogen	Carcinogen
Predicted Endpoint	Carcinogen	Non-Carcinogen	Non-Carcinogen
Similarity	0.415	0.395	0.383
Reference	US FDA (Centre for Drug Eval.& Res./Off. Testing & Res.) Sept. 1997	US FDA (Centre for Drug Eval.& Res./Off. Testing & Res.) Sept. 1997	US FDA (Centre for Drug Eval.& Res./Off. Testing & Res.) Sept. 1997

Model Applicability

Missing features are fingerprint features in the query molecule not found in the training set.

1. All descriptors are in range.

Feature Contribution

Top features for Carcinogen

Fingerprint	Bit	Structure/Smiles	Score	Carcinogen in training set
FCFP_10	-1678275541	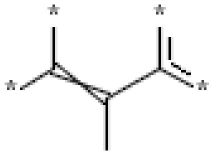	0.799	4 out of 4

FCFP_10	1985089045	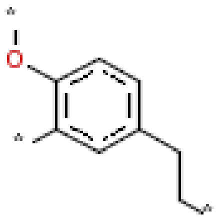	0.454	5 out of 9
FCFP_10	-922480536	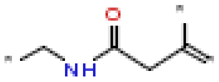	0.422	1 out of 1
**Top Features for Non-Carcinogen**				
**Fingerprint**	**Bit**	**Structure/Smiles**	**Score**	**Carcinogen in training set**
FCFP_10	-1038421835	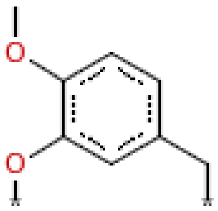	-0.810	0 out of 4
FCFP_10	-1099193755	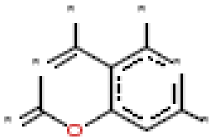	-0.503	3 out of 18
FCFP_10	-1405834164	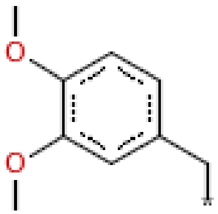	-0.485	0 out of 2

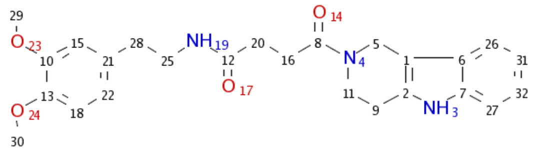

$C_{25}H_{29}N_3O_4$
Molecular Weight: 435.51546
ALogP: 2.831
Rotatable Bonds: 8
Acceptors: 4
Donors: 2

Model Prediction

Prediction: Carcinogen
Probability: 0.265
Enrichment: 0.823
Bayesian Score: -0.369

Prediction: Positive if the Bayesian score is above the estimated best cutoff value.
Probability: The esimated probability that the sample is in the category.
Enrichment: An estimate of enrichment, that is, the increased likelihood (versus random) of this sample being in the category.
Bayesian Score: The standard Laplacian-modified Bayesian score.

Structural Similar Compounds

Name	Deserpidine	Reserpine	Pergolide
Structure	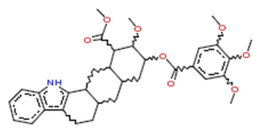	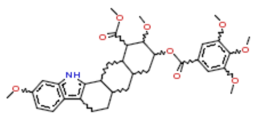	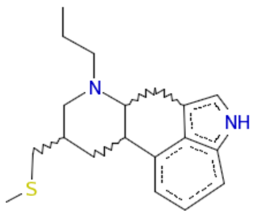
Actual Endpoint	Carcinogen	Carcinogen	Carcinogen
Predicted Endpoint	Carcinogen	Carcinogen	Carcinogen
Similarity	0.377	0.352	0.303
Reference	US FDA (Centre for Drug Eval.& Res./Off. Testing & Res.) Sept. 1997	US FDA (Centre for Drug Eval.& Res./Off. Testing & Res.) Sept. 1997	US FDA (Centre for Drug Eval.& Res./Off. Testing & Res.) Sept. 1997

Model Applicability

Missing features are fingerprint features in the query molecule not found in the training set.

1. All descriptors are in range.

Feature Contribution

Top features for Carcinogen				
**Fingerprint**	**Bit**	**Structure/Smiles**	**Score**	**Carcinogen in training set**
FCFP_10	2005402822	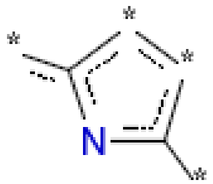	0.461	9 out of 17

FCFP_10	1985089045	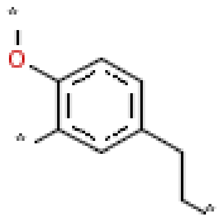	0.454	5 out of 9
FCFP_10	1673997923	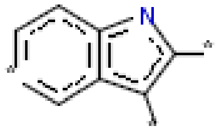	0.451	4 out of 7
**Top Features for Non-Carcinogen**				
**Fingerprint**	**Bit**	**Structure/Smiles**	**Score**	**Carcinogen in training set**
FCFP_10	-1038421835	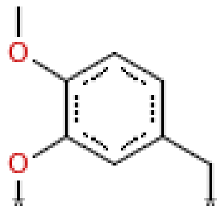	-0.810	0 out of 4
FCFP_10	907036844	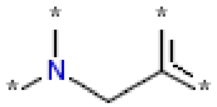	-0.503	3 out of 18
FCFP_10	206055935	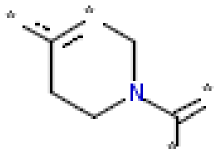	-0.485	0 out of 2

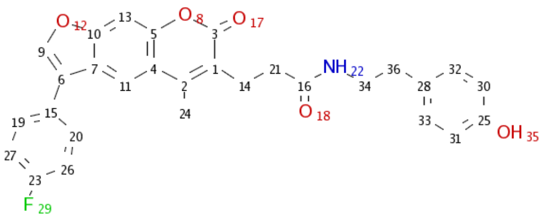

$C_{29}H_{24}FNO_5$
Molecular Weight: 485.50296
ALogP: 5.695
Rotatable Bonds: 7
Acceptors: 4
Donors: 2

Model Prediction

Prediction: Non-Carcinogen
Probability: 0.204
Enrichment: 0.635
Bayesian Score: -7.475

Prediction: Positive if the Bayesian score is above the estimated best cutoff value.
Probability: The esimated probability that the sample is in the category.
Enrichment: An estimate of enrichment, that is, the increased likelihood (versus random) of this sample being in the category.
Bayesian Score: The standard Laplacian-modified Bayesian score.

Structural Similar Compounds

Name	Glyburide	Fluvastatin	Glimepride
Structure	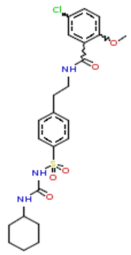	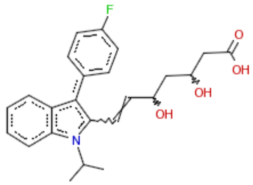	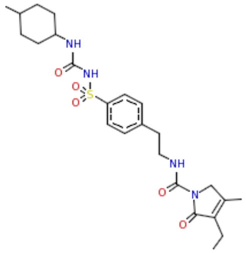
Actual Endpoint	Non-Carcinogen	Non-Carcinogen	Non-Carcinogen
Predicted Endpoint	Non-Carcinogen	Non-Carcinogen	Non-Carcinogen
Similarity	0.357	0.324	0.319
Reference	US FDA (Centre for Drug Eval.& Res./Off. Testing & Res.) Sept. 1997	US FDA (Centre for Drug Eval.& Res./Off. Testing & Res.) Sept. 1997	US FDA (Centre for Drug Eval.& Res./Off. Testing & Res.) Sept. 1997

Model Applicability

Missing features are fingerprint features in the query molecule not found in the training set.

1. All descriptors are in range.

Feature Contribution

Top features for Carcinogen

Fingerprint	Bit	Structure/Smiles	Score	Carcinogen in training set
FCFP_10	-1678275541	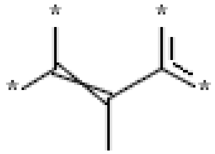	0.799	4 out of 4

FCFP_10	-1066794953	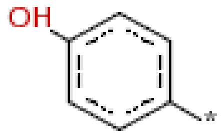	0.737	5 out of 6
FCFP_10	-922480536	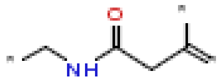	0.422	1 out of 1

Top Features for Non-Carcinogen

Fingerprint	Bit	Structure/Smiles	Score	Carcinogen in training set
FCFP_10	1985639687	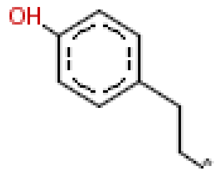	-0.810	0 out of 4
FCFP_10	-1508180856	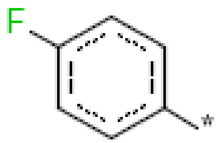	-0.791	2 out of 18
FCFP_10	551850122	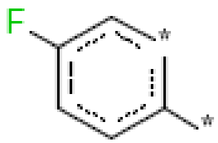	-0.679	9 out of 60

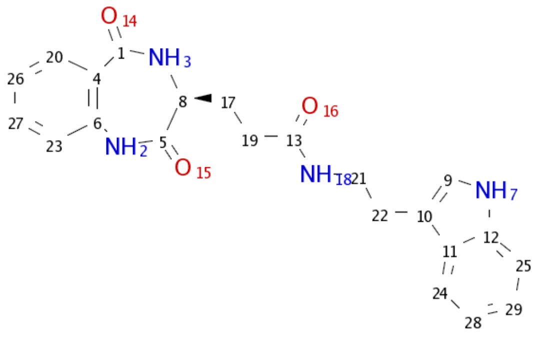

$C_{22}H_{22}N_4O_3$
Molecular Weight: 390.43508
ALogP: 1.807
Rotatable Bonds: 6
Acceptors: 3
Donors: 4

Model Prediction

Prediction: Carcinogen
Probability: 0.277
Enrichment: 0.860
Bayesian Score: 0.282

Prediction: Positive if the Bayesian score is above the estimated best cutoff value.
Probability: The esimated probability that the sample is in the category.
Enrichment: An estimate of enrichment, that is, the increased likelihood (versus random) of this sample being in the category.
Bayesian Score: The standard Laplacian-modified Bayesian score.

Structural Similar Compounds

Name	Tryptophan	Prilocaine	Sumatriptan
Structure	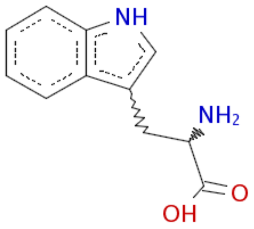	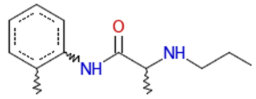	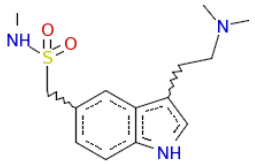
Actual Endpoint	Non-Carcinogen	Carcinogen	Non-Carcinogen
Predicted Endpoint	Non-Carcinogen	Carcinogen	Non-Carcinogen
Similarity	0.440	0.400	0.375
Reference	US FDA (Centre for Drug Eval.& Res./Off. Testing & Res.) Sept. 1997	US FDA (Centre for Drug Eval.& Res./Off. Testing & Res.) Sept. 1997	US FDA (Centre for Drug Eval.& Res./Off. Testing & Res.) Sept. 1997

Model Applicability

Missing features are fingerprint features in the query molecule not found in the training set.

1. All descriptors are in range.

Feature Contribution

Top features for Carcinogen				
**Fingerprint**	**Bit**	**Structure/Smiles**	**Score**	**Carcinogen in training set**
FCFP_10	2005402822	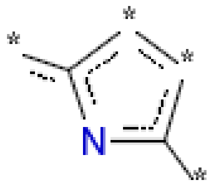	0.461	9 out of 17

FCFP_10	1673997923	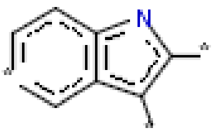	0.451	4 out of 7
FCFP_10	155061250	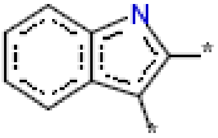	0.446	3 out of 5

Top Features for Non-Carcinogen

Fingerprint	Bit	Structure/Smiles	Score	Carcinogen in training set
FCFP_10	1011367537	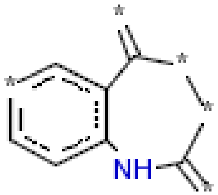	-0.644	1 out of 9
FCFP_10	244927241	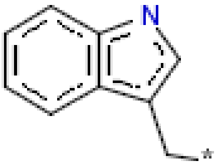	-0.485	0 out of 2
FCFP_10	-2002900105	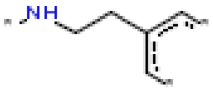	-0.465	1 out of 7

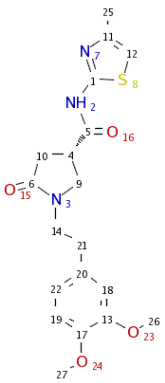

$C_{19}H_{23}N_3O_4S$
Molecular Weight: 389.46862
ALogP: 1.461
Rotatable Bonds: 7
Acceptors: 5
Donors: 1

Model Prediction

Prediction: Carcinogen
Probability: 0.251
Enrichment: 0.778
Bayesian Score: -1.273

Prediction: Positive if the Bayesian score is above the estimated best cutoff value.
Probability: The esimated probability that the sample is in the category.
Enrichment: An estimate of enrichment, that is, the increased likelihood (versus random) of this sample being in the category.
Bayesian Score: The standard Laplacian-modified Bayesian score.

Structural Similar Compounds

Name	Praziquantel	Sulfamethazine	Furothiazole
Structure	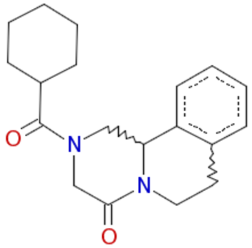	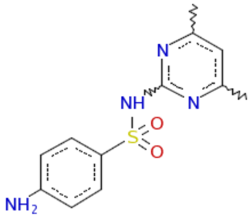	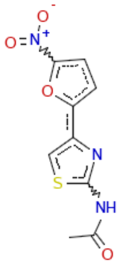
Actual Endpoint	Non-Carcinogen	Carcinogen	Carcinogen
Predicted Endpoint	Non-Carcinogen	Carcinogen	Carcinogen
Similarity	0.315	0.314	0.302
Reference	US FDA (Centre for Drug Eval.& Res./Off. Testing & Res.) Sept. 1997	US FDA (Centre for Drug Eval.& Res./Off. Testing & Res.) Sept. 1997	US FDA (Centre for Drug Eval.& Res./Off. Testing & Res.) Sept. 1997

Model Applicability

Missing features are fingerprint features in the query molecule not found in the training set.

1. All descriptors are in range.

Feature Contribution

Top features for Carcinogen

Fingerprint	Bit	Structure/Smiles	Score	Carcinogen in training set
FCFP_10	-1776076225	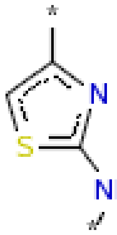	0.576	3 out of 4

FCFP_10	1985089045	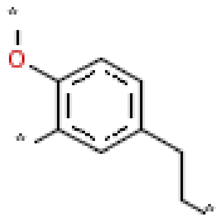	0.454	5 out of 9
FCFP_10	-1773715875	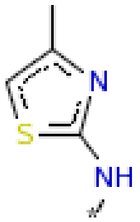	0.438	2 out of 3

Top Features for Non-Carcinogen

Fingerprint	Bit	Structure/Smiles	Score	Carcinogen in training set
FCFP_10	-2005207466	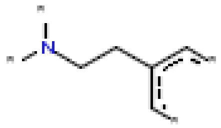	-0.810	0 out of 4
FCFP_10	-1038421835	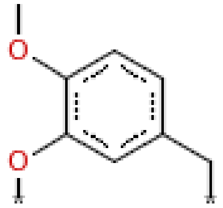	-0.810	0 out of 4
FCFP_10	1743817318	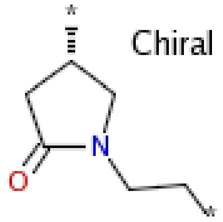	-0.810	0 out of 4

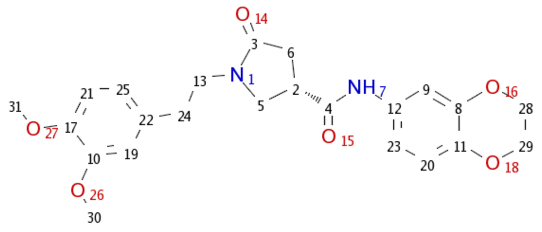

$C_{23}H_{26}N_2O_6$
Molecular Weight: 426.46234
ALogP: 1.967
Rotatable Bonds: 7
Acceptors: 6
Donors: 1

Model Prediction

Prediction: Non-Carcinogen
Probability: 0.227
Enrichment: 0.706
Bayesian Score: -3.165

Prediction: Positive if the Bayesian score is above the estimated best cutoff value.
Probability: The esimated probability that the sample is in the category.
Enrichment: An estimate of enrichment, that is, the increased likelihood (versus random) of this sample being in the category.
Bayesian Score: The standard Laplacian-modified Bayesian score.

Structural Similar Compounds

Name	Phenacetin	Nitroacetophenetide	Carteolol
Structure	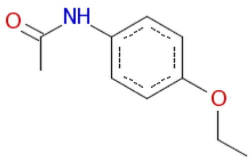	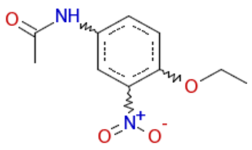	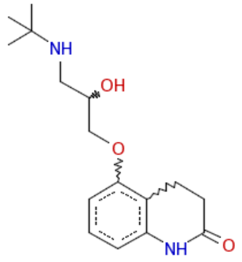
Actual Endpoint	Carcinogen	Non-Carcinogen	Non-Carcinogen
Predicted Endpoint	Carcinogen	Carcinogen	Non-Carcinogen
Similarity	0.425	0.409	0.358
Reference	US FDA (Centre for Drug Eval.& Res./Off. Testing & Res.) Sept. 1997	US FDA (Centre for Drug Eval.& Res./Off. Testing & Res.) Sept. 1997	US FDA (Centre for Drug Eval.& Res./Off. Testing & Res.) Sept. 1997

Model Applicability

Missing features are fingerprint features in the query molecule not found in the training set.

1. All descriptors are in range.

Feature Contribution

Top features for Carcinogen

Fingerprint	Bit	Structure/Smiles	Score	Carcinogen in training set
FCFP_10	-1838187238	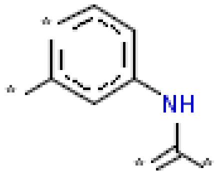	0.454	5 out of 9

FCFP_10	1985089045	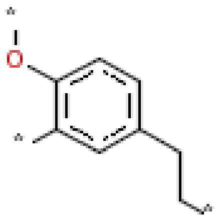	0.454	5 out of 9
FCFP_10	1908043702	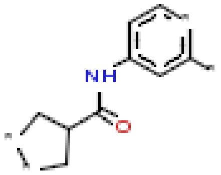	0.422	1 out of 1
**Top Features for Non-Carcinogen**				
**Fingerprint**	**Bit**	**Structure/Smiles**	**Score**	**Carcinogen in training set**
FCFP_10	-1038421835	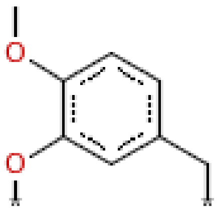	-0.810	0 out of 4
FCFP_10	1743817318	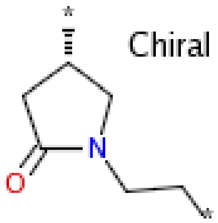	-0.810	0 out of 4
FCFP_10	-2005207466	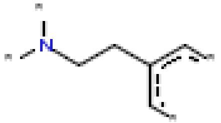	-0.810	0 out of 4

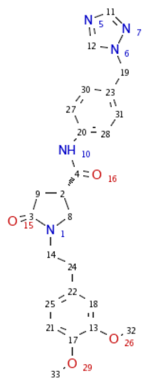

$C_{24}H_{27}N_5O_4$
Molecular Weight: 449.50228
ALogP: 1.731
Rotatable Bonds: 9
Acceptors: 6
Donors: 1

Model Prediction

Prediction: Non-Carcinogen
Probability: 0.234
Enrichment: 0.727
Bayesian Score: -2.554

Prediction: Positive if the Bayesian score is above the estimated best cutoff value.
Probability: The esimated probability that the sample is in the category.
Enrichment: An estimate of enrichment, that is, the increased likelihood (versus random) of this sample being in the category.
Bayesian Score: The standard Laplacian-modified Bayesian score.

Structural Similar Compounds

Name	Phenacetin	Nitroacetophenetide	Praziquantel
Structure	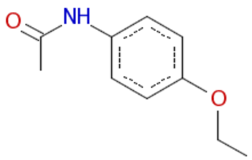	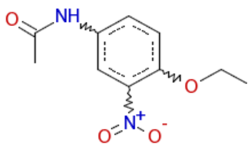	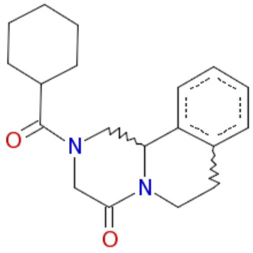
Actual Endpoint	Carcinogen	Non-Carcinogen	Non-Carcinogen
Predicted Endpoint	Carcinogen	Carcinogen	Non-Carcinogen
Similarity	0.314	0.309	0.293
Reference	US FDA (Centre for Drug Eval.& Res./Off. Testing & Res.) Sept. 1997	US FDA (Centre for Drug Eval.& Res./Off. Testing & Res.) Sept. 1997	US FDA (Centre for Drug Eval.& Res./Off. Testing & Res.) Sept. 1997

Model Applicability

Missing features are fingerprint features in the query molecule not found in the training set.

1. All descriptors are in range.

Feature Contribution

Top features for Carcinogen

Fingerprint	Bit	Structure/Smiles	Score	Carcinogen in training set
FCFP_10	-451043714	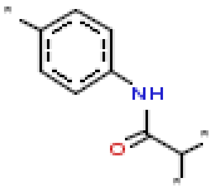	0.614	2 out of 2

FCFP_10	1985089045	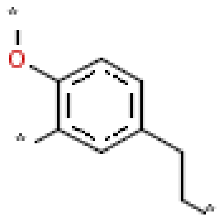	0.454	5 out of 9
FCFP_10	-1838187238	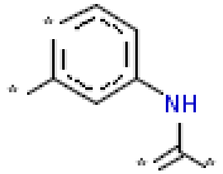	0.454	5 out of 9

Top Features for Non-Carcinogen

**Fingerprint**	**Bit**	**Structure/Smiles**	**Score**	**Carcinogen in training set**
FCFP_10	1743817318	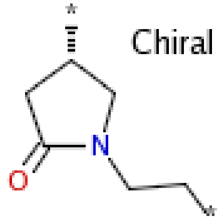	-0.810	0 out of 4
FCFP_10	-1038421835	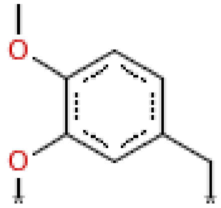	-0.810	0 out of 4
FCFP_10	-2005207466	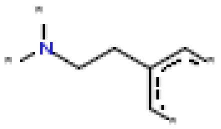	-0.810	0 out of 4

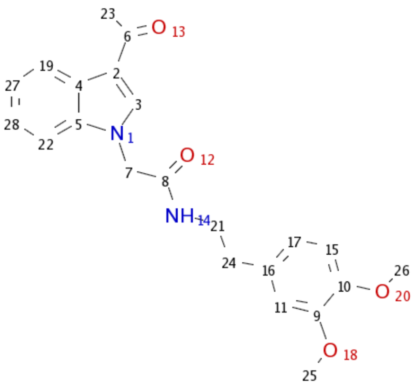

$C_{22}H_{24}N_2O_4$
Molecular Weight: 380.43696
ALogP: 3.001
Rotatable Bonds: 8
Acceptors: 4
Donors: 1

Model Prediction

Prediction: Non-Carcinogen
Probability: 0.205
Enrichment: 0.636
Bayesian Score: -7.178

Prediction: Positive if the Bayesian score is above the estimated best cutoff value.
Probability: The esimated probability that the sample is in the category.
Enrichment: An estimate of enrichment, that is, the increased likelihood (versus random) of this sample being in the category.
Bayesian Score: The standard Laplacian-modified Bayesian score.

Structural Similar Compounds

Name	Propafenone	Glyburide	Ondansetron
Structure	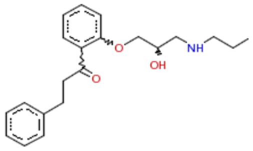	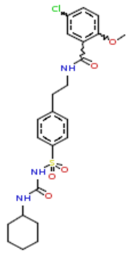	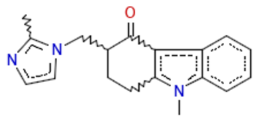
Actual Endpoint	Non-Carcinogen	Non-Carcinogen	Non-Carcinogen
Predicted Endpoint	Non-Carcinogen	Non-Carcinogen	Non-Carcinogen
Similarity	0.345	0.344	0.333
Reference	US FDA (Centre for Drug Eval.& Res./Off. Testing & Res.) Sept. 1997	US FDA (Centre for Drug Eval.& Res./Off. Testing & Res.) Sept. 1997	US FDA (Centre for Drug Eval.& Res./Off. Testing & Res.) Sept. 1997

Model Applicability

Missing features are fingerprint features in the query molecule not found in the training set.

1. All descriptors are in range.

Feature Contribution

Top features for Carcinogen

Fingerprint	Bit	Structure/Smiles	Score	Carcinogen in training set
FCFP_10	1985089045	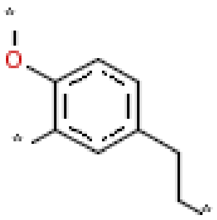	0.454	5 out of 9

FCFP_10	-387072142	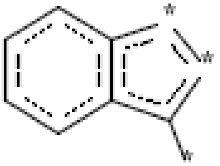	0.376	5 out of 10
FCFP_10	566058135	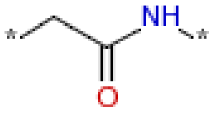	0.209	16 out of 41

Top Features for Non-Carcinogen

Fingerprint	Bit	Structure/Smiles	Score	Carcinogen in training set
FCFP_10	-1038421835	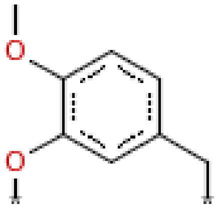	-0.810	0 out of 4
FCFP_10	956098679	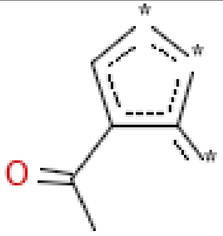	-0.810	0 out of 4
FCFP_10	-898059030	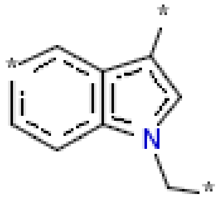	-0.661	0 out of 3

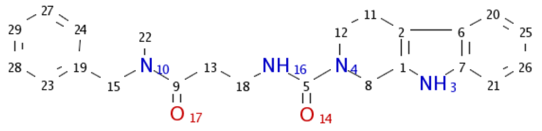

$C_{23}H_{26}N_4O_2$
Molecular Weight: 390.47814
ALogP: 2.702
Rotatable Bonds: 5
Acceptors: 2
Donors: 2

Model Prediction

Prediction: Carcinogen
Probability: 0.338
Enrichment: 1.049
Bayesian Score: 2.853

Prediction: Positive if the Bayesian score is above the estimated best cutoff value.
Probability: The esimated probability that the sample is in the category.
Enrichment: An estimate of enrichment, that is, the increased likelihood (versus random) of this sample being in the category.
Bayesian Score: The standard Laplacian-modified Bayesian score.

Structural Similar Compounds

Name	Etodolac	Tryptophan	Deserpidine
Structure	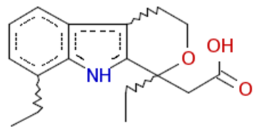	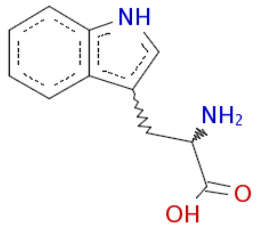	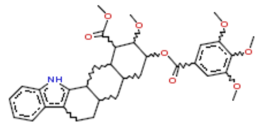
Actual Endpoint	Non-Carcinogen	Non-Carcinogen	Carcinogen
Predicted Endpoint	Non-Carcinogen	Non-Carcinogen	Carcinogen
Similarity	0.368	0.340	0.304
Reference	US FDA (Centre for Drug Eval.& Res./Off. Testing & Res.) Sept. 1997	US FDA (Centre for Drug Eval.& Res./Off. Testing & Res.) Sept. 1997	US FDA (Centre for Drug Eval.& Res./Off. Testing & Res.) Sept. 1997

Model Applicability

Missing features are fingerprint features in the query molecule not found in the training set.

1. All descriptors are in range.

Feature Contribution

Top features for Carcinogen				
**Fingerprint**	**Bit**	**Structure/Smiles**	**Score**	**Carcinogen in training set**
FCFP_10	-2051315385	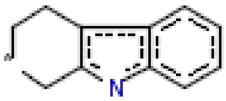	0.614	2 out of 2

FCFP_10	-1986098826	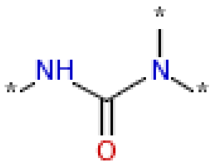	0.576	8 out of 13
FCFP_10	2005402822	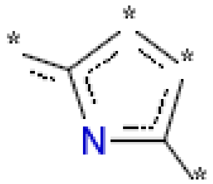	0.461	9 out of 17
**Top Features for Non-Carcinogen**				
**Fingerprint**	**Bit**	**Structure/Smiles**	**Score**	**Carcinogen in training set**
FCFP_10	-2005207466	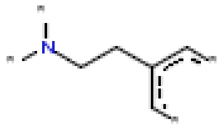	-0.810	0 out of 4
FCFP_10	-2093839777	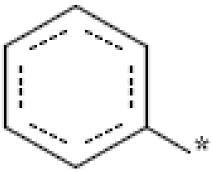	-0.558	11 out of 64
FCFP_10	907036844	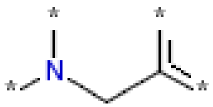	-0.503	3 out of 18

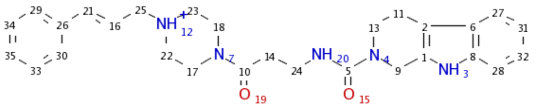

$C_{28}H_{34}N_5O_2$
Molecular Weight: 472.60186
ALogP: 1.567
Rotatable Bonds: 6
Acceptors: 2
Donors: 3

Model Prediction

Prediction: Carcinogen
Probability: 0.290
Enrichment: 0.899
Bayesian Score: 0.897

Prediction: Positive if the Bayesian score is above the estimated best cutoff value.
Probability: The esimated probability that the sample is in the category.
Enrichment: An estimate of enrichment, that is, the increased likelihood (versus random) of this sample being in the category.
Bayesian Score: The standard Laplacian-modified Bayesian score.

Structural Similar Compounds

Name	Etodolac	Tryptophan	Bromocriptine
Structure	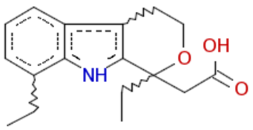	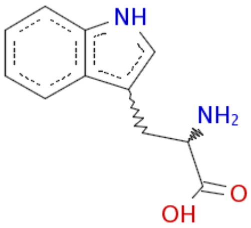	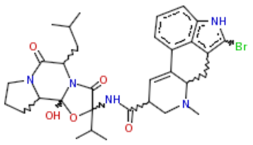
Actual Endpoint	Non-Carcinogen	Non-Carcinogen	Carcinogen
Predicted Endpoint	Non-Carcinogen	Non-Carcinogen	Carcinogen
Similarity	0.333	0.286	0.272
Reference	US FDA (Centre for Drug Eval.& Res./Off. Testing & Res.) Sept. 1997	US FDA (Centre for Drug Eval.& Res./Off. Testing & Res.) Sept. 1997	US FDA (Centre for Drug Eval.& Res./Off. Testing & Res.) Sept. 1997

Model Applicability

Missing features are fingerprint features in the query molecule not found in the training set.

1. Missing Feature: FCFP_10 10 [*][NH+]([*])[*]
2. Missing Feature: FCFP_10 -1853714334 [*]C[NH+](C[*])C[*]
3. Missing Feature: FCFP_10 1155241219 [*]CC[NH+]([*])[*]

Feature Contribution

Top features for Carcinogen

Fingerprint	Bit	Structure/Smiles	Score	Carcinogen in training set
FCFP_10	-2051315385	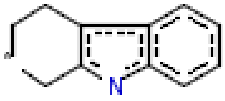	0.614	2 out of 2

FCFP_10	-1986098826	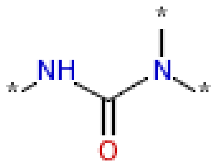	0.576	8 out of 13
FCFP_10	2005402822	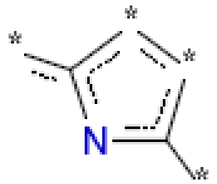	0.461	9 out of 17

Top Features for Non-Carcinogen

**Fingerprint**	**Bit**	**Structure/Smiles**	**Score**	**Carcinogen in training set**
FCFP_10	1743817318	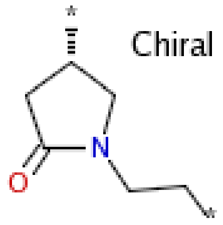	-0.810	0 out of 4
FCFP_10	-2005207466	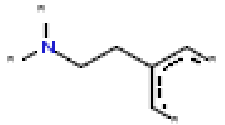	-0.810	0 out of 4
FCFP_10	-2093839777	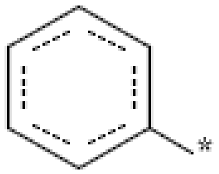	-0.558	11 out of 64

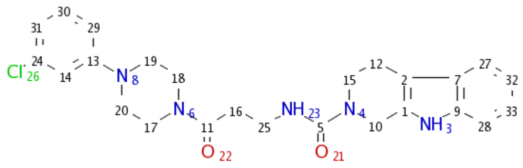

$C_{25}H_{28}ClN_5O_2$
Molecular Weight: 465.97512
ALogP: 3.413
Rotatable Bonds: 4
Acceptors: 3
Donors: 2

Model Prediction

Prediction: Non-Carcinogen
Probability: 0.238
Enrichment: 0.738
Bayesian Score: -2.246

Prediction: Positive if the Bayesian score is above the estimated best cutoff value.
Probability: The esimated probability that the sample is in the category.
Enrichment: An estimate of enrichment, that is, the increased likelihood (versus random) of this sample being in the category.
Bayesian Score: The standard Laplacian-modified Bayesian score.

Structural Similar Compounds

Name	Temazepam	Doxefazepam	Amoxapine
Structure	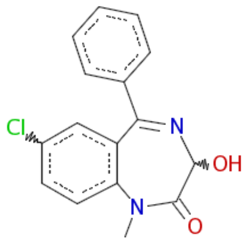	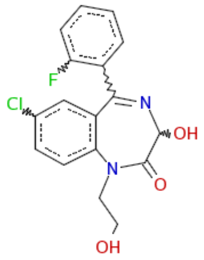	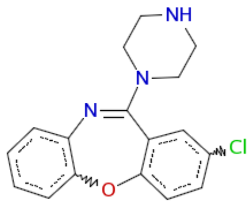
Actual Endpoint	Non-Carcinogen	Carcinogen	Carcinogen
Predicted Endpoint	Non-Carcinogen	Non-Carcinogen	Carcinogen
Similarity	0.339	0.338	0.333
Reference	US FDA (Centre for Drug Eval.& Res./Off. Testing & Res.) Sept. 1997	US FDA (Centre for Drug Eval.& Res./Off. Testing & Res.) Sept. 1997	US FDA (Centre for Drug Eval.& Res./Off. Testing & Res.) Sept. 1997

Model Applicability

Missing features are fingerprint features in the query molecule not found in the training set.

1. All descriptors are in range.

Feature Contribution

Top features for Carcinogen

Fingerprint	Bit	Structure/Smiles	Score	Carcinogen in training set
FCFP_10	-2051315385	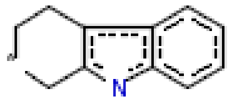	0.614	2 out of 2

FCFP_10	-1986098826	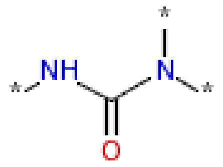	0.576	8 out of 13
FCFP_10	2005402822	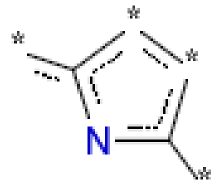	0.461	9 out of 17
**Top Features for Non-Carcinogen**				
**Fingerprint**	**Bit**	**Structure/Smiles**	**Score**	**Carcinogen in training set**
FCFP_10	1546024460	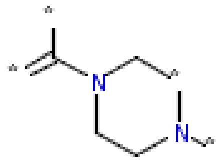	-1.055	0 out of 6
FCFP_10	-2005207466	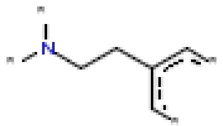	-0.810	0 out of 4
FCFP_10	1743817318	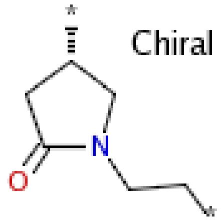	-0.810	0 out of 4

MOST ACTIVE.mol

TOPKAT_Rat_Female_FDA_None_vs_Carcinogen

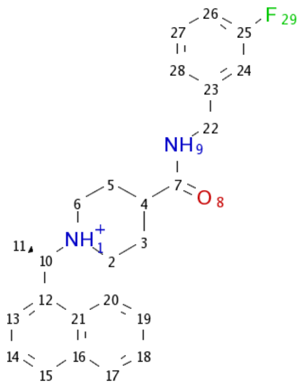

$C_{25}H_{28}FN_2O$
Molecular Weight: 391.50102
ALogP: 3.098
Rotatable Bonds: 5
Acceptors: 1
Donors: 2

Model Prediction

Prediction: Non-Carcinogen
Probability: 0.221
Enrichment: 0.686
Bayesian Score: -3.898

Prediction: Positive if the Bayesian score is above the estimated best cutoff value.
Probability: The esimated probability that the sample is in the category.
Enrichment: An estimate of enrichment, that is, the increased likelihood (versus random) of this sample being in the category.
Bayesian Score: The standard Laplacian-modified Bayesian score.

Structural Similar Compounds

Name	Bupropion	Chlorpropamide	Phenobarbital
Structure	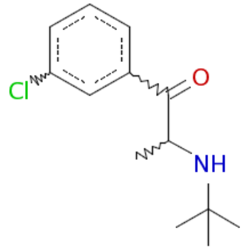	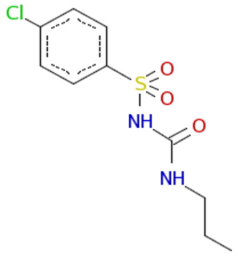	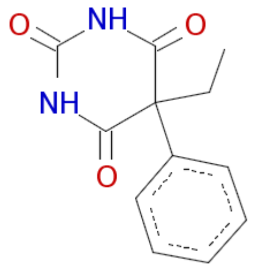
Actual Endpoint	Non-Carcinogen	Non-Carcinogen	Carcinogen
Predicted Endpoint	Non-Carcinogen	Non-Carcinogen	Carcinogen
Similarity	0.362	0.327	0.311
Reference	US FDA (Centre for Drug Eval.& Res./Off. Testing & Res.) Sept. 1997	US FDA (Centre for Drug Eval.& Res./Off. Testing & Res.) Sept. 1997	US FDA (Centre for Drug Eval.& Res./Off. Testing & Res.) Sept. 1997

Model Applicability

Missing features are fingerprint features in the query molecule not found in the training set.

1. Missing Feature: FCFP_10 10 [*][NH+]([*])[*]
2. Missing Feature: FCFP_10 -1853714334 [*]C[NH+](C[*])C[*]
3. Missing Feature: FCFP_10 1155241219 [*]CC[NH+]([*])[*]
4. Missing Feature: FCFP_10 -680623486 [*][NH+]([*])[C@H](C)[c](:[*]):[*]

Feature Contribution

Top features for Carcinogen

Fingerprint	Bit	Structure/Smiles	Score	Carcinogen in training set
FCFP_10	-387072142	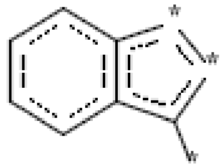	0.376	5 out of 10

FCFP_10	907096426	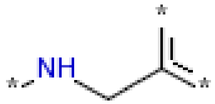	0.331	3 out of 6
FCFP_10	-1043339860	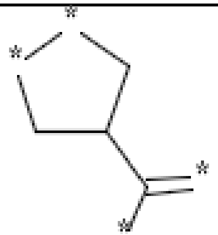	0.284	31 out of 74
**Top Features for Non-Carcinogen**				
**Fingerprint**	**Bit**	**Structure/Smiles**	**Score**	**Carcinogen in training set**
FCFP_10	551850122	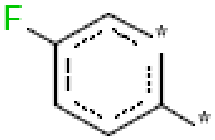	-0.679	9 out of 60
FCFP_10	-384723139	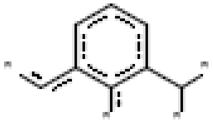	-0.661	0 out of 3
FCFP_10	-745491832	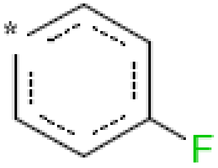	-0.652	7 out of 46

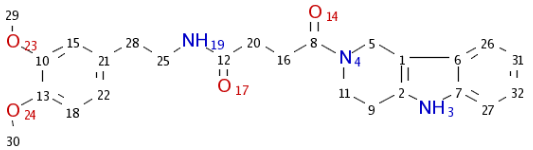

$C_{25}H_{29}N_3O_4$

Molecular Weight: 435.51546

ALogP: 2.831

Rotatable Bonds: 8

Acceptors: 4

Donors: 2

Model Prediction

Prediction: Single-Carcinogen

Probability: 0.324

Enrichment: 0.866

Bayesian Score: -5.468

Prediction: Positive if the Bayesian score is above the estimated best cutoff value.
Probability: The esimated probability that the sample is in the category.
Enrichment: An estimate of enrichment, that is, the increased likelihood (versus random) of this sample being in the category.
Bayesian Score: The standard Laplacian-modified Bayesian score.

Structural Similar Compounds

Name	Deserpidine	Reserpine	Mesuprine
Structure	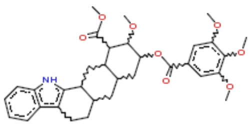	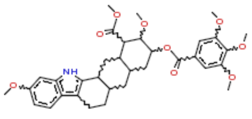	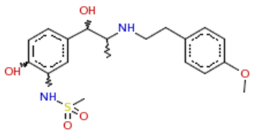
Actual Endpoint	Single-Carcinogen	Single-Carcinogen	Single-Carcinogen
Predicted Endpoint	Single-Carcinogen	Single-Carcinogen	Single-Carcinogen
Similarity	0.377	0.352	0.303
Reference	US FDA (Centre for Drug Eval.& Res./Off. Testing & Res.) Sept. 1997	US FDA (Centre for Drug Eval.& Res./Off. Testing & Res.) Sept. 1997	US FDA (Centre for Drug Eval.& Res./Off. Testing & Res.) Sept. 1997

Model Applicability

Missing features are fingerprint features in the query molecule not found in the training set.

1. Missing Feature: SCFP_4 -587479743 [*]N([*])C[c](:[*]):[*]

Feature Contribution

Top features for Multiple-Carcinogen				
Fingerprint	Bit	Structure/Smiles	Score	Multiple-Carcinogen in training set
SCFP_4	-111024397	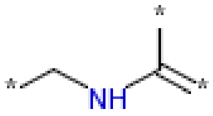	0.351	4 out of 8

SCFP_4	1256995004	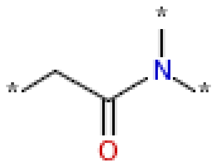	0.327	10 out of 22
SCFP_4	-1374800107	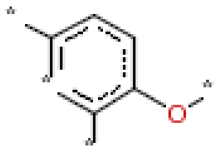	0.288	10 out of 23

Top Features for Single-Carcinogen

Fingerprint	Bit	Structure/Smiles	Score	Multiple-Carcinogen in training set
SCFP_4	-1272709286	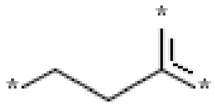	-1.157	1 out of 17
SCFP_4	-1211866396	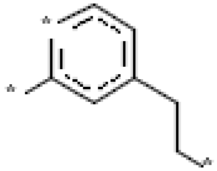	-1.062	0 out of 6
SCFP_4	-1343150366	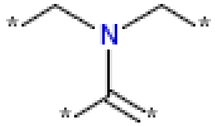	-0.946	0 out of 5

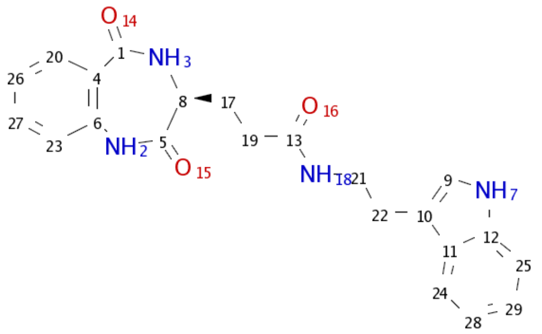

$C_{22}H_{22}N_4O_3$
Molecular Weight: 390.43508
ALogP: 1.807
Rotatable Bonds: 6
Acceptors: 3
Donors: 4

Model Prediction

Prediction: Single-Carcinogen
Probability: 0.443
Enrichment: 1.185
Bayesian Score: -1.750

Prediction: Positive if the Bayesian score is above the estimated best cutoff value.
Probability: The esimated probability that the sample is in the category.
Enrichment: An estimate of enrichment, that is, the increased likelihood (versus random) of this sample being in the category.
Bayesian Score: The standard Laplacian-modified Bayesian score.

Structural Similar Compounds

Name	Prilocaine	Pergolide	Aminoglutethimide
Structure	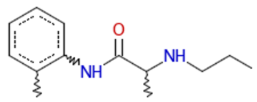	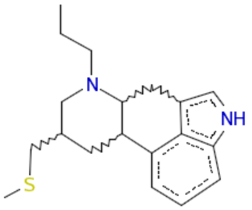	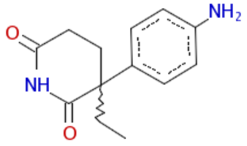
Actual Endpoint	Multiple-Carcinogen	Single-Carcinogen	Multiple-Carcinogen
Predicted Endpoint	Multiple-Carcinogen	Single-Carcinogen	Multiple-Carcinogen
Similarity	0.400	0.344	0.283
Reference	US FDA (Centre for Drug Eval.& Res./Off. Testing & Res.) Sept. 1997	US FDA (Centre for Drug Eval.& Res./Off. Testing & Res.) Sept. 1997	US FDA (Centre for Drug Eval.& Res./Off. Testing & Res.) Sept. 1997

Model Applicability

Missing features are fingerprint features in the query molecule not found in the training set.

1. All descriptors are in range.

Feature Contribution

Top features for Multiple-Carcinogen

Fingerprint	Bit	Structure/Smiles	Score	Multiple-Carcinogen in training set
SCFP_4	2097618059	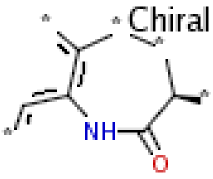	0.730	5 out of 6

SCFP_4	1631845520	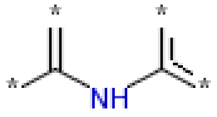	0.601	6 out of 9
SCFP_4	-1375926917	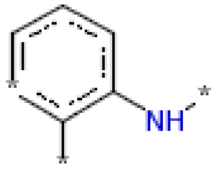	0.522	6 out of 10

Top Features for Single-Carcinogen

**Fingerprint**	**Bit**	**Structure/Smiles**	**Score**	**Multiple-Carcinogen in training set**
SCFP_4	-1272709286	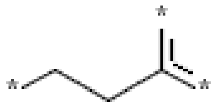	-1.157	1 out of 17
SCFP_4	-1946889102	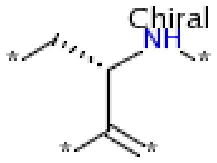	-0.816	0 out of 4
SCFP_4	622342378	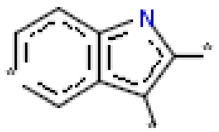	-0.816	0 out of 4

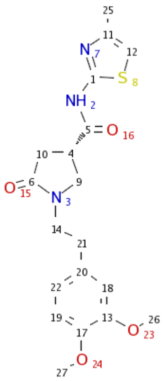

$C_{19}H_{23}N_3O_4S$
Molecular Weight: 389.46862
ALogP: 1.461
Rotatable Bonds: 7
Acceptors: 5
Donors: 1

Model Prediction

Prediction: Single-Carcinogen
Probability: 0.405
Enrichment: 1.083
Bayesian Score: -3.161

Prediction: Positive if the Bayesian score is above the estimated best cutoff value.
Probability: The esimated probability that the sample is in the category.
Enrichment: An estimate of enrichment, that is, the increased likelihood (versus random) of this sample being in the category.
Bayesian Score: The standard Laplacian-modified Bayesian score.

Structural Similar Compounds

Name	Sulfamethazine	Furothiazole	Omeprazole
Structure	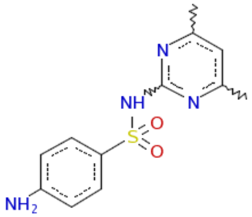	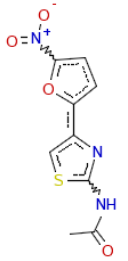	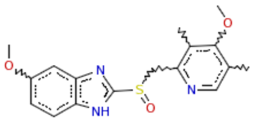
Actual Endpoint	Single-Carcinogen	Single-Carcinogen	Multiple-Carcinogen
Predicted Endpoint	Single-Carcinogen	Multiple-Carcinogen	Multiple-Carcinogen
Similarity	0.314	0.302	0.290
Reference	US FDA (Centre for Drug Eval.& Res./Off. Testing & Res.) Sept. 1997	US FDA (Centre for Drug Eval.& Res./Off. Testing & Res.) Sept. 1997	US FDA (Centre for Drug Eval.& Res./Off. Testing & Res.) Sept. 1997

Model Applicability

Missing features are fingerprint features in the query molecule not found in the training set.

1. All descriptors are in range.

Feature Contribution

Top features for Multiple-Carcinogen

Fingerprint	Bit	Structure/Smiles	Score	Multiple-Carcinogen in training set
SCFP_4	1631845520	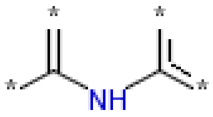	0.601	6 out of 9

SCFP_4	17	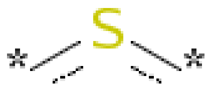	0.548	10 out of 17
SCFP_4	1310748454	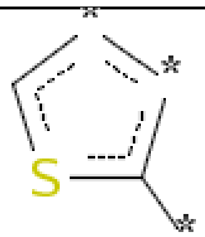	0.444	4 out of 7
**Top Features for Single-Carcinogen**				
**Fingerprint**	**Bit**	**Structure/Smiles**	**Score**	**Multiple-Carcinogen in training set**
SCFP_4	-1272709286	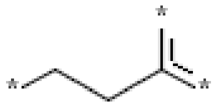	-1.157	1 out of 17
SCFP_4	-1211866396	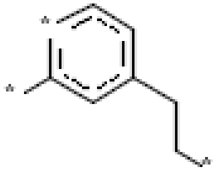	-1.062	0 out of 6
SCFP_4	-1343150366	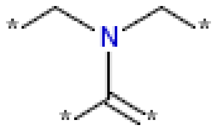	-0.946	0 out of 5

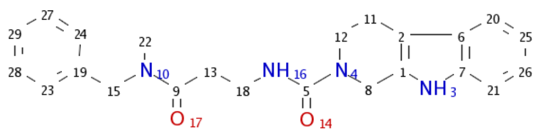

$C_{23}H_{26}N_4O_2$
Molecular Weight: 390.47814
ALogP: 2.702
Rotatable Bonds: 5
Acceptors: 2
Donors: 2

Model Prediction

Prediction: Single-Carcinogen
Probability: 0.387
Enrichment: 1.034
Bayesian Score: -3.730

Prediction: Positive if the Bayesian score is above the estimated best cutoff value.
Probability: The esimated probability that the sample is in the category.
Enrichment: An estimate of enrichment, that is, the increased likelihood (versus random) of this sample being in the category.
Bayesian Score: The standard Laplacian-modified Bayesian score.

Structural Similar Compounds

Name	Deserpidine	Pergolide	Bromocriptine
Structure	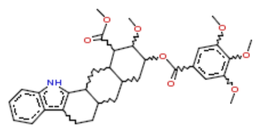	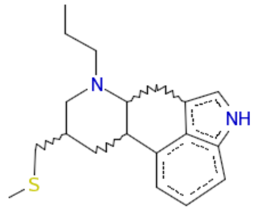	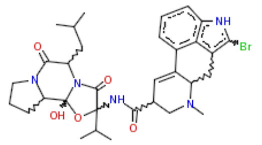
Actual Endpoint	Single-Carcinogen	Single-Carcinogen	Single-Carcinogen
Predicted Endpoint	Single-Carcinogen	Single-Carcinogen	Single-Carcinogen
Similarity	0.304	0.286	0.274
Reference	US FDA (Centre for Drug Eval.& Res./Off. Testing & Res.) Sept. 1997	US FDA (Centre for Drug Eval.& Res./Off. Testing & Res.) Sept. 1997	US FDA (Centre for Drug Eval.& Res./Off. Testing & Res.) Sept. 1997

Model Applicability

Missing features are fingerprint features in the query molecule not found in the training set.

1. Missing Feature: SCFP_4 -587479743 [*]N([*])C[c](:[*]):[*]

Feature Contribution

Top features for Multiple-Carcinogen				
**Fingerprint**	**Bit**	**Structure/Smiles**	**Score**	**Multiple-Carcinogen in training set**
SCFP_4	-1629661413	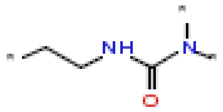	0.433	2 out of 3

SCFP_4	-111024397	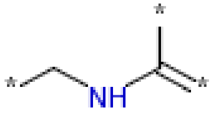	0.351	4 out of 8
SCFP_4	1256995004	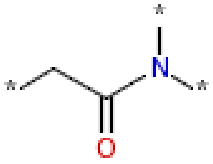	0.327	10 out of 22

Top Features for Single-Carcinogen

**Fingerprint**	**Bit**	**Structure/Smiles**	**Score**	**Multiple-Carcinogen in training set**
SCFP_4	-1272709286	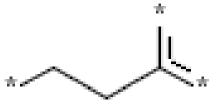	-1.157	1 out of 17
SCFP_4	-1343150366	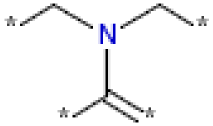	-0.946	0 out of 5
SCFP_4	622342378	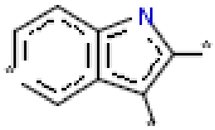	-0.816	0 out of 4

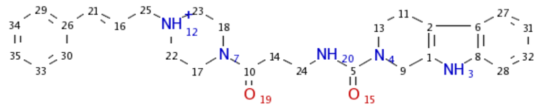

$C_{28}H_{34}N_5O_2$
Molecular Weight: 472.60186
ALogP: 1.567
Rotatable Bonds: 6
Acceptors: 2
Donors: 3

Model Prediction

Prediction: Single-Carcinogen
Probability: 0.362
Enrichment: 0.968
Bayesian Score: -4.447

Prediction: Positive if the Bayesian score is above the estimated best cutoff value.
Probability: The esimated probability that the sample is in the category.
Enrichment: An estimate of enrichment, that is, the increased likelihood (versus random) of this sample being in the category.
Bayesian Score: The standard Laplacian-modified Bayesian score.

Structural Similar Compounds

Name	Bromocriptine	Deserpidine	Pergolide
Structure	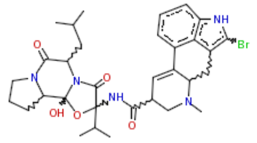	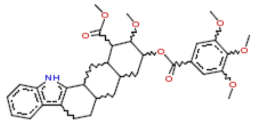	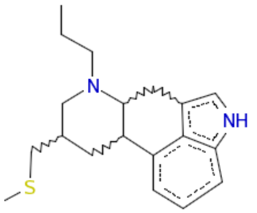
Actual Endpoint	Single-Carcinogen	Single-Carcinogen	Single-Carcinogen
Predicted Endpoint	Single-Carcinogen	Single-Carcinogen	Single-Carcinogen
Similarity	0.272	0.266	0.247
Reference	US FDA (Centre for Drug Eval.& Res./Off. Testing & Res.) Sept. 1997	US FDA (Centre for Drug Eval.& Res./Off. Testing & Res.) Sept. 1997	US FDA (Centre for Drug Eval.& Res./Off. Testing & Res.) Sept. 1997

Model Applicability

Missing features are fingerprint features in the query molecule not found in the training set.

1. Missing Feature: SCFP_4 11 [*][NH+]([*])[*]
2. Missing Feature: SCFP_4 -587479743 [*]N([*])C[c](:[*]):[*]
3. Missing Feature: SCFP_4 1930718785 [*]C[NH+](C[*])C[*]
4. Missing Feature: SCFP_4 -1396915742 [*]CC[NH+]([*])[*]
5. Missing Feature: SCFP_4 -1396885951 [*][NH+]([*])CC=[*]

Feature Contribution

Top features for Multiple-Carcinogen

Fingerprint	Bit	Structure/Smiles	Score	Multiple-Carcinogen in training set
SCFP_4	387787917	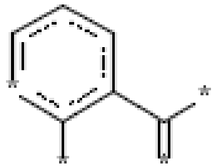	0.449	6 out of 11

SCFP_4	-1629661413	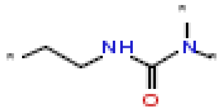	0.433	2 out of 3
SCFP_4	-111024397	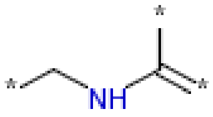	0.351	4 out of 8

Top Features for Single-Carcinogen

**Fingerprint**	**Bit**	**Structure/Smiles**	**Score**	**Multiple-Carcinogen in training set**
SCFP_4	-1272709286	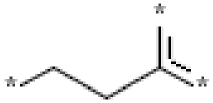	-1.157	1 out of 17
SCFP_4	-1343150366	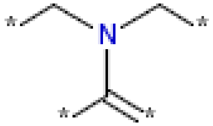	-0.946	0 out of 5
SCFP_4	622342378	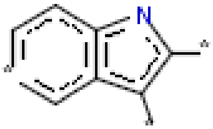	-0.816	0 out of 4

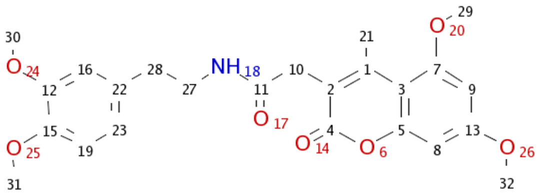

$C_{24}H_{27}NO_7$
Molecular Weight: 441.47368
ALogP: 3.388
Rotatable Bonds: 9
Acceptors: 7
Donors: 1

Model Prediction

Prediction: Non-Carcinogen
Probability: 0.231
Enrichment: 0.692
Bayesian Score: -6.420

Prediction: Positive if the Bayesian score is above the estimated best cutoff value.
Probability: The esimated probability that the sample is in the category.
Enrichment: An estimate of enrichment, that is, the increased likelihood (versus random) of this sample being in the category.
Bayesian Score: The standard Laplacian-modified Bayesian score.

Structural Similar Compounds

Name	Mesuprine	Nabumetone	Atenolol
Structure	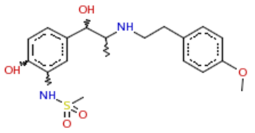	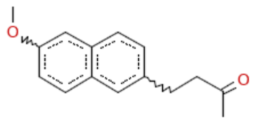	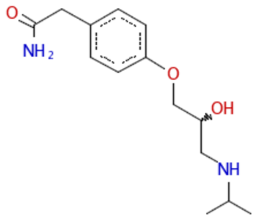
Actual Endpoint	Non-Carcinogen	Non-Carcinogen	Carcinogen
Predicted Endpoint	Non-Carcinogen	Non-Carcinogen	Non-Carcinogen
Similarity	0.415	0.395	0.383
Reference	US FDA (Centre for Drug Eval.& Res./Off. Testing & Res.) Sept. 1997	US FDA (Centre for Drug Eval.& Res./Off. Testing & Res.) Sept. 1997	US FDA (Centre for Drug Eval.& Res./Off. Testing & Res.) Sept. 1997

Model Applicability

Missing features are fingerprint features in the query molecule not found in the training set.

1. All descriptors are in range.

Feature Contribution

Top features for Carcinogen

Fingerprint	Bit	Structure/Smiles	Score	Carcinogen in training set
SCFP_12	-392286499	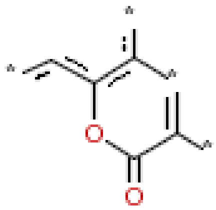	0.610	2 out of 2

SCFP_12	470041467	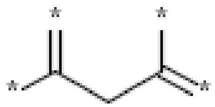	0.433	2 out of 3
SCFP_12	55523958	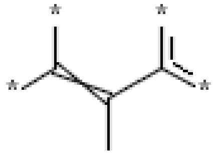	0.433	2 out of 3

Top Features for Non-Carcinogen

**Fingerprint**	**Bit**	**Structure/Smiles**	**Score**	**Carcinogen in training set**
SCFP_12	-1642341584	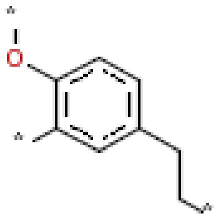	-1.258	0 out of 8
SCFP_12	-1211866396	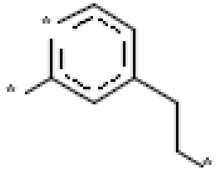	-1.084	2 out of 25
SCFP_12	-1849236245	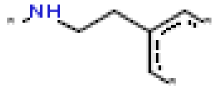	-0.815	0 out of 4

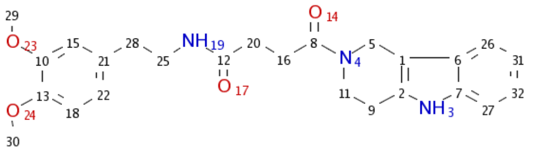

$C_{25}H_{29}N_3O_4$
Molecular Weight: 435.51546
ALogP: 2.831
Rotatable Bonds: 8
Acceptors: 4
Donors: 2

Model Prediction

Prediction: Non-Carcinogen
Probability: 0.229
Enrichment: 0.684
Bayesian Score: -7.143

Prediction: Positive if the Bayesian score is above the estimated best cutoff value.
Probability: The esimated probability that the sample is in the category.
Enrichment: An estimate of enrichment, that is, the increased likelihood (versus random) of this sample being in the category.
Bayesian Score: The standard Laplacian-modified Bayesian score.

Structural Similar Compounds

Name	Deserpidine	Reserpine	Pergolide
Structure	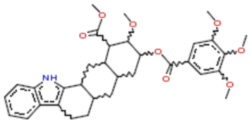	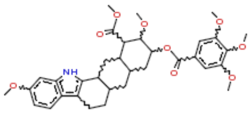	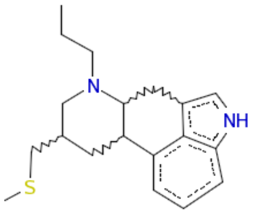
Actual Endpoint	Carcinogen	Carcinogen	Non-Carcinogen
Predicted Endpoint	Carcinogen	Carcinogen	Non-Carcinogen
Similarity	0.377	0.352	0.303
Reference	US FDA (Centre for Drug Eval.& Res./Off. Testing & Res.) Sept. 1997	US FDA (Centre for Drug Eval.& Res./Off. Testing & Res.) Sept. 1997	US FDA (Centre for Drug Eval.& Res./Off. Testing & Res.) Sept. 1997

Model Applicability

Missing features are fingerprint features in the query molecule not found in the training set.

1. All descriptors are in range.

Feature Contribution

Top features for Carcinogen

Fingerprint	Bit	Structure/Smiles	Score	Carcinogen in training set
SCFP_12	1651620003	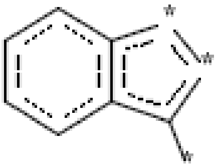	0.657	7 out of 10

SCFP_12	-1379673609	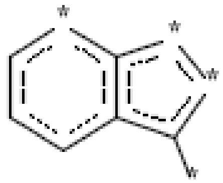	0.541	11 out of 19
SCFP_12	1655199790	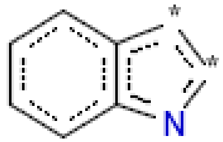	0.533	5 out of 8
**Top Features for Non-Carcinogen**				
**Fingerprint**	**Bit**	**Structure/Smiles**	**Score**	**Carcinogen in training set**
SCFP_12	-1642341584	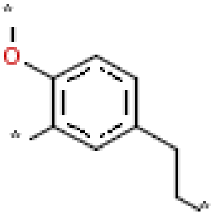	-1.258	0 out of 8
SCFP_12	-1211866396	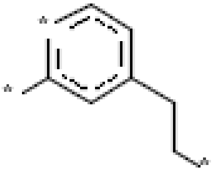	-1.084	2 out of 25
SCFP_12	-1849236245	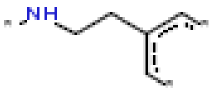	-0.815	0 out of 4

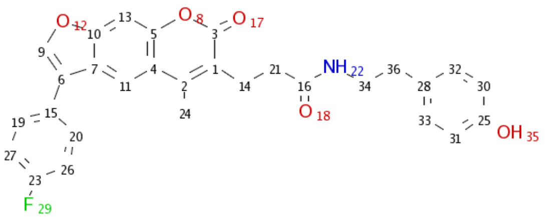

$C_{29}H_{24}FNO_5$
Molecular Weight: 485.50296
ALogP: 5.695
Rotatable Bonds: 7
Acceptors: 4
Donors: 2

Model Prediction

Prediction: Non-Carcinogen
Probability: 0.278
Enrichment: 0.832
Bayesian Score: -1.052

Prediction: Positive if the Bayesian score is above the estimated best cutoff value.
Probability: The esimated probability that the sample is in the category.
Enrichment: An estimate of enrichment, that is, the increased likelihood (versus random) of this sample being in the category.
Bayesian Score: The standard Laplacian-modified Bayesian score.

Structural Similar Compounds

Name	Glyburide	Fluvastatin	Glimepride
Structure	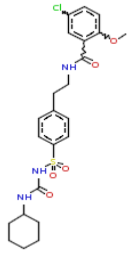	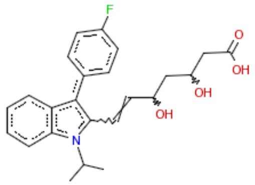	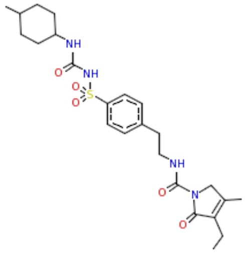
Actual Endpoint	Non-Carcinogen	Carcinogen	Non-Carcinogen
Predicted Endpoint	Non-Carcinogen	Carcinogen	Non-Carcinogen
Similarity	0.357	0.324	0.319
Reference	US FDA (Centre for Drug Eval.& Res./Off. Testing & Res.) Sept. 1997	US FDA (Centre for Drug Eval.& Res./Off. Testing & Res.) Sept. 1997	US FDA (Centre for Drug Eval.& Res./Off. Testing & Res.) Sept. 1997

Model Applicability

Missing features are fingerprint features in the query molecule not found in the training set.

1. All descriptors are in range.

Feature Contribution

Top features for Carcinogen

Fingerprint	Bit	Structure/Smiles	Score	Carcinogen in training set
SCFP_12	112644006	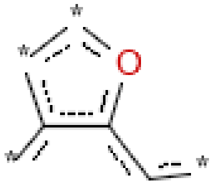	0.721	3 out of 3

SCFP_12	794417578	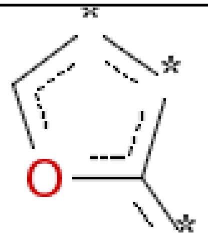	0.615	9 out of 14
SCFP_12	-392286499	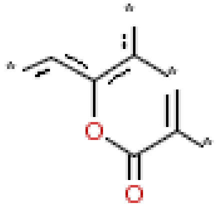	0.610	2 out of 2
**Top Features for Non-Carcinogen**				
**Fingerprint**	**Bit**	**Structure/Smiles**	**Score**	**Carcinogen in training set**
SCFP_12	-1642341584	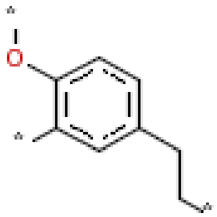	-1.258	0 out of 8
SCFP_12	-1211866396	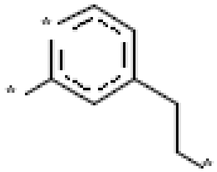	-1.084	2 out of 25
SCFP_12	-1849236245	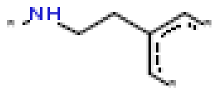	-0.815	0 out of 4

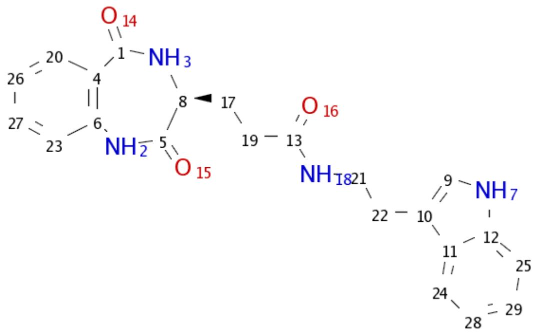

$C_{22}H_{22}N_4O_3$
Molecular Weight: 390.43508
ALogP: 1.807
Rotatable Bonds: 6
Acceptors: 3
Donors: 4

Model Prediction

Prediction: Non-Carcinogen

Probability: 0.242

Enrichment: 0.725

Bayesian Score: -4.493

Prediction: Positive if the Bayesian score is above the estimated best cutoff value.
Probability: The esimated probability that the sample is in the category.
Enrichment: An estimate of enrichment, that is, the increased likelihood (versus random) of this sample being in the category.
Bayesian Score: The standard Laplacian-modified Bayesian score.

Structural Similar Compounds

Name	Tryptophan	Prilocaine	Sumatriptan
Structure	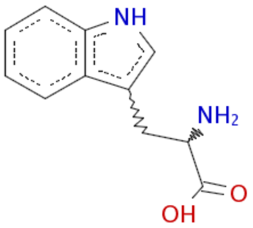	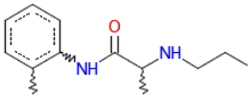	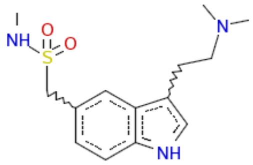
Actual Endpoint	Non-Carcinogen	Carcinogen	Non-Carcinogen
Predicted Endpoint	Non-Carcinogen	Carcinogen	Non-Carcinogen
Similarity	0.440	0.400	0.375
Reference	US FDA (Centre for Drug Eval.& Res./Off. Testing & Res.) Sept. 1997	US FDA (Centre for Drug Eval.& Res./Off. Testing & Res.) Sept. 1997	US FDA (Centre for Drug Eval.& Res./Off. Testing & Res.) Sept. 1997

Model Applicability

Missing features are fingerprint features in the query molecule not found in the training set.

1. All descriptors are in range.

Feature Contribution

Top features for Carcinogen

Fingerprint	Bit	Structure/Smiles	Score	Carcinogen in training set
SCFP_12	1651620003	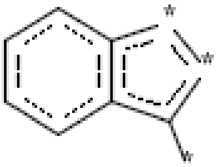	0.657	7 out of 10

SCFP_12	-1379673609	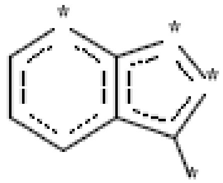	0.541	11 out of 19
SCFP_12	1655199790	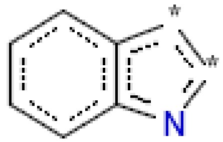	0.533	5 out of 8
**Top Features for Non-Carcinogen**				
**Fingerprint**	**Bit**	**Structure/Smiles**	**Score**	**Carcinogen in training set**
SCFP_12	-1946889102	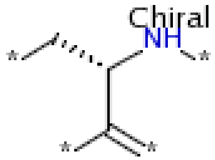	-0.871	1 out of 12
SCFP_12	-1849236245	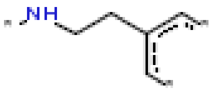	-0.815	0 out of 4
SCFP_12	-1770674960	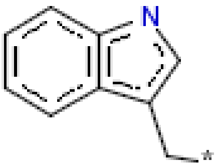	-0.665	0 out of 3

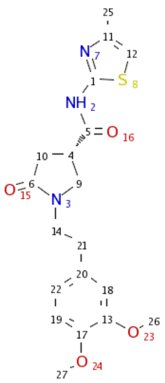

$C_{19}H_{23}N_3O_4S$
Molecular Weight: 389.46862
ALogP: 1.461
Rotatable Bonds: 7
Acceptors: 5
Donors: 1

Model Prediction

Prediction: Non-Carcinogen
Probability: 0.244
Enrichment: 0.730
Bayesian Score: -4.294

Prediction: Positive if the Bayesian score is above the estimated best cutoff value.
Probability: The esimated probability that the sample is in the category.
Enrichment: An estimate of enrichment, that is, the increased likelihood (versus random) of this sample being in the category.
Bayesian Score: The standard Laplacian-modified Bayesian score.

Structural Similar Compounds

Name	Praziquantel	Sulfamethazine	Furothiazole
Structure	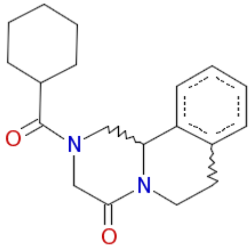	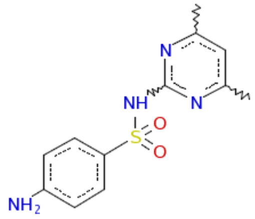	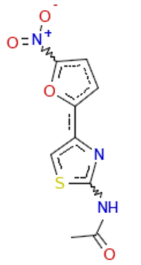
Actual Endpoint	Non-Carcinogen	Carcinogen	Carcinogen
Predicted Endpoint	Non-Carcinogen	Carcinogen	Carcinogen
Similarity	0.315	0.314	0.302
Reference	US FDA (Centre for Drug Eval.& Res./Off. Testing & Res.) Sept. 1997	US FDA (Centre for Drug Eval.& Res./Off. Testing & Res.) Sept. 1997	US FDA (Centre for Drug Eval.& Res./Off. Testing & Res.) Sept. 1997

Model Applicability

Missing features are fingerprint features in the query molecule not found in the training set.

1. All descriptors are in range.

Feature Contribution

Top features for Carcinogen

Fingerprint	Bit	Structure/Smiles	Score	Carcinogen in training set
SCFP_12	-100019659	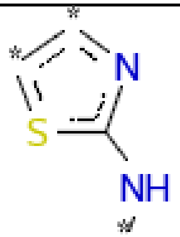	0.610	2 out of 2

SCFP_12	1310748454	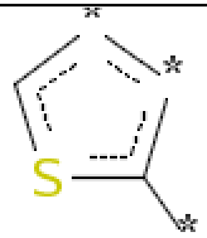	0.451	7 out of 13
SCFP_12	-614089895	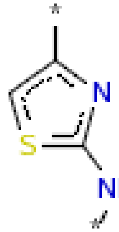	0.419	1 out of 1

Top Features for Non-Carcinogen

Fingerprint	Bit	Structure/Smiles	Score	Carcinogen in training set
SCFP_12	-1642341584	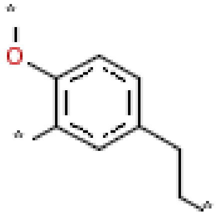	-1.258	0 out of 8
SCFP_12	-1211866396	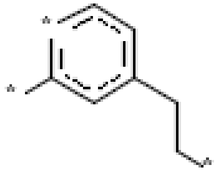	-1.084	2 out of 25
SCFP_12	-1849236245	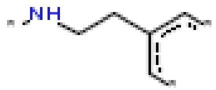	-0.815	0 out of 4

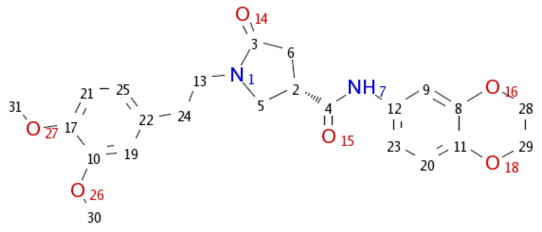

$C_{23}H_{26}N_2O_6$
Molecular Weight: 426.46234
ALogP: 1.967
Rotatable Bonds: 7
Acceptors: 6
Donors: 1

Model Prediction

Prediction: Non-Carcinogen
Probability: 0.235
Enrichment: 0.703
Bayesian Score: -5.668

Prediction: Positive if the Bayesian score is above the estimated best cutoff value.
Probability: The esimated probability that the sample is in the category.
Enrichment: An estimate of enrichment, that is, the increased likelihood (versus random) of this sample being in the category.
Bayesian Score: The standard Laplacian-modified Bayesian score.

Structural Similar Compounds

Name	Phenacetin	Nitroacetophenetide	Carteolol
Structure	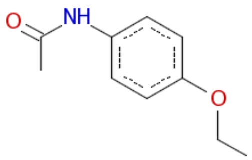	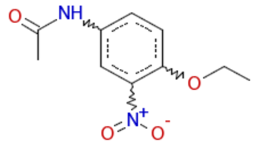	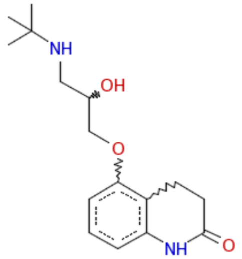
Actual Endpoint	Carcinogen	Non-Carcinogen	Non-Carcinogen
Predicted Endpoint	Carcinogen	Carcinogen	Non-Carcinogen
Similarity	0.425	0.409	0.358
Reference	US FDA (Centre for Drug Eval.& Res./Off. Testing & Res.) Sept. 1997	US FDA (Centre for Drug Eval.& Res./Off. Testing & Res.) Sept. 1997	US FDA (Centre for Drug Eval.& Res./Off. Testing & Res.) Sept. 1997

Model Applicability

Missing features are fingerprint features in the query molecule not found in the training set.

1. All descriptors are in range.

Feature Contribution

Top features for Carcinogen

Fingerprint	Bit	Structure/Smiles	Score	Carcinogen in training set
SCFP_12	-347048986	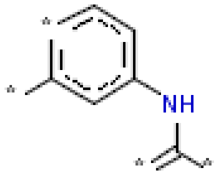	0.627	5 out of 7

SCFP_12	2097618059	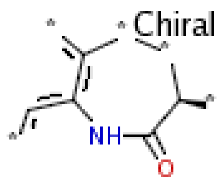	0.451	7 out of 13
SCFP_12	-1329734318	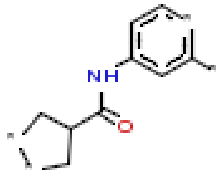	0.419	1 out of 1
**Top Features for Non-Carcinogen**				
**Fingerprint**	**Bit**	**Structure/Smiles**	**Score**	**Carcinogen in training set**
SCFP_12	-1642341584	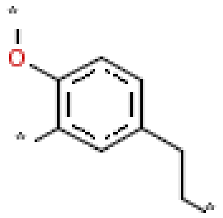	-1.258	0 out of 8
SCFP_12	-1211866396	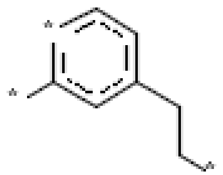	-1.084	2 out of 25
SCFP_12	-1849236245	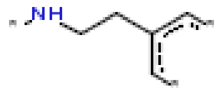	-0.815	0 out of 4

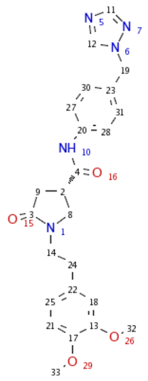

$C_{24}H_{27}N_5O_4$
Molecular Weight: 449.50228
ALogP: 1.731
Rotatable Bonds: 9
Acceptors: 6
Donors: 1

Model Prediction

Prediction: Non-Carcinogen

Probability: 0.284

Enrichment: 0.851

Bayesian Score: -0.599

Prediction: Positive if the Bayesian score is above the estimated best cutoff value.
Probability: The esimated probability that the sample is in the category.
Enrichment: An estimate of enrichment, that is, the increased likelihood (versus random) of this sample being in the category.
Bayesian Score: The standard Laplacian-modified Bayesian score.

Structural Similar Compounds

Name	Phenacetin	Nitroacetophenetide	Praziquantel
Structure	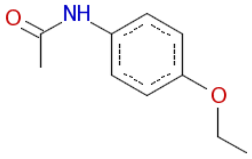	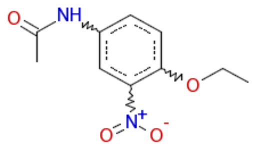	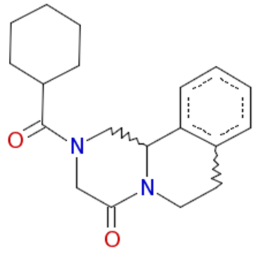
Actual Endpoint	Carcinogen	Non-Carcinogen	Non-Carcinogen
Predicted Endpoint	Carcinogen	Carcinogen	Non-Carcinogen
Similarity	0.314	0.309	0.293
Reference	US FDA (Centre for Drug Eval.& Res./Off. Testing & Res.) Sept. 1997	US FDA (Centre for Drug Eval.& Res./Off. Testing & Res.) Sept. 1997	US FDA (Centre for Drug Eval.& Res./Off. Testing & Res.) Sept. 1997

Model Applicability

Missing features are fingerprint features in the query molecule not found in the training set.

1. All descriptors are in range.

Feature Contribution

Top features for Carcinogen

Fingerprint	Bit	Structure/Smiles	Score	Carcinogen in training set
SCFP_12	599957850	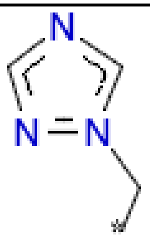	0.721	3 out of 3

SCFP_12	-443296553	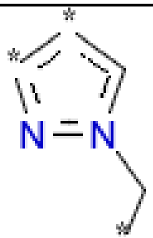	0.664	4 out of 5
SCFP_12	-347048986	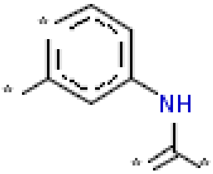	0.627	5 out of 7

Top Features for Non-Carcinogen

Fingerprint	Bit	Structure/Smiles	Score	Carcinogen in training set
SCFP_12	-1642341584	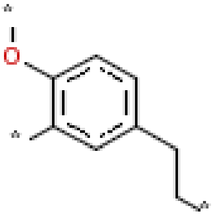	-1.258	0 out of 8
SCFP_12	-1211866396	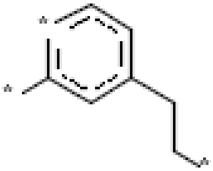	-1.084	2 out of 25
SCFP_12	-1849236245	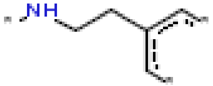	-0.815	0 out of 4

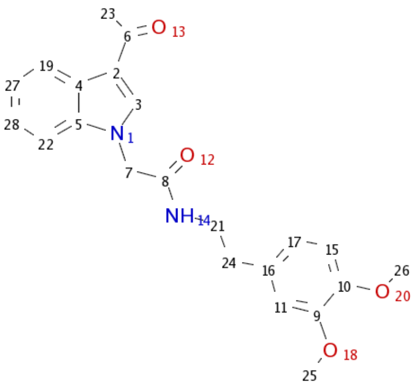

$C_{22}H_{24}N_2O_4$
Molecular Weight: 380.43696
ALogP: 3.001
Rotatable Bonds: 8
Acceptors: 4
Donors: 1

Model Prediction

Prediction: Non-Carcinogen
Probability: 0.227
Enrichment: 0.679
Bayesian Score: -7.695

Prediction: Positive if the Bayesian score is above the estimated best cutoff value.
Probability: The esimated probability that the sample is in the category.
Enrichment: An estimate of enrichment, that is, the increased likelihood (versus random) of this sample being in the category.
Bayesian Score: The standard Laplacian-modified Bayesian score.

Structural Similar Compounds

Name	Propafenone	Glyburide	Mesuprine
Structure	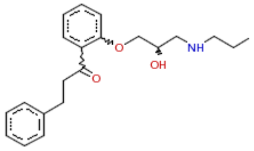	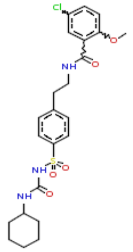	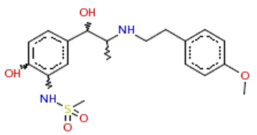
Actual Endpoint	Non-Carcinogen	Non-Carcinogen	Non-Carcinogen
Predicted Endpoint	Non-Carcinogen	Non-Carcinogen	Non-Carcinogen
Similarity	0.345	0.344	0.333
Reference	US FDA (Centre for Drug Eval.& Res./Off. Testing & Res.) Sept. 1997	US FDA (Centre for Drug Eval.& Res./Off. Testing & Res.) Sept. 1997	US FDA (Centre for Drug Eval.& Res./Off. Testing & Res.) Sept. 1997

Model Applicability

Missing features are fingerprint features in the query molecule not found in the training set.

1. Missing Feature: SCFP_12 1964617636 [*]C(=[*])Cn(:[*]):[*]

Feature Contribution

Top features for Carcinogen

Fingerprint	Bit	Structure/Smiles	Score	Carcinogen in training set
SCFP_12	1651620003	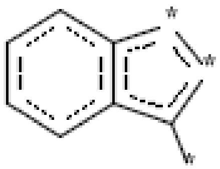	0.657	7 out of 10

SCFP_12	-1379673609	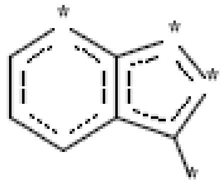	0.541	11 out of 19
SCFP_12	1655199790	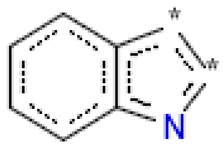	0.533	5 out of 8

Top Features for Non-Carcinogen

Fingerprint	Bit	Structure/Smiles	Score	Carcinogen in training set
SCFP_12	-1642341584	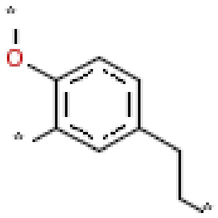	-1.258	0 out of 8
SCFP_12	-1211866396	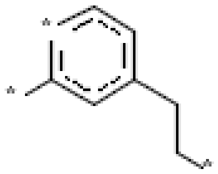	-1.084	2 out of 25
SCFP_12	-1849236245	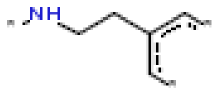	-0.815	0 out of 4

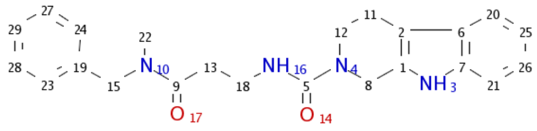

$C_{23}H_{26}N_4O_2$
Molecular Weight: 390.47814
ALogP: 2.702
Rotatable Bonds: 5
Acceptors: 2
Donors: 2

Model Prediction

Prediction: Carcinogen
Probability: 0.293
Enrichment: 0.876
Bayesian Score: -0.042

Prediction: Positive if the Bayesian score is above the estimated best cutoff value.
Probability: The esimated probability that the sample is in the category.
Enrichment: An estimate of enrichment, that is, the increased likelihood (versus random) of this sample being in the category.
Bayesian Score: The standard Laplacian-modified Bayesian score.

Structural Similar Compounds

Name	Etodolac	Tryptophan	Deserpidine
Structure	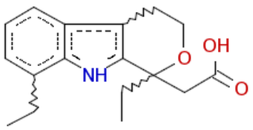	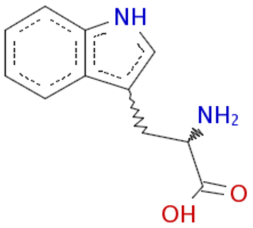	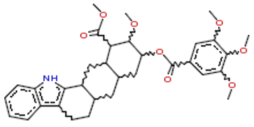
Actual Endpoint	Non-Carcinogen	Non-Carcinogen	Carcinogen
Predicted Endpoint	Non-Carcinogen	Non-Carcinogen	Carcinogen
Similarity	0.368	0.340	0.304
Reference	US FDA (Centre for Drug Eval.& Res./Off. Testing & Res.) Sept. 1997	US FDA (Centre for Drug Eval.& Res./Off. Testing & Res.) Sept. 1997	US FDA (Centre for Drug Eval.& Res./Off. Testing & Res.) Sept. 1997

Model Applicability

Missing features are fingerprint features in the query molecule not found in the training set.

1. All descriptors are in range.

Feature Contribution

Top features for Carcinogen

Fingerprint	Bit	Structure/Smiles	Score	Carcinogen in training set
SCFP_12	1651620003	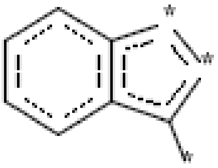	0.657	7 out of 10

SCFP_12	1962031760	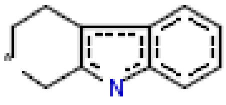	0.610	2 out of 2
SCFP_12	-1379673609	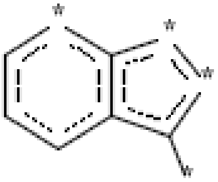	0.541	11 out of 19
**Top Features for Non-Carcinogen**				
**Fingerprint**	**Bit**	**Structure/Smiles**	**Score**	**Carcinogen in training set**
SCFP_12	-1849236245	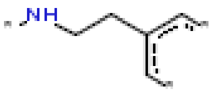	-0.815	0 out of 4
SCFP_12	-1343150366	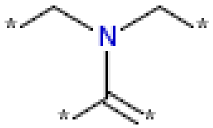	-0.644	3 out of 21
SCFP_12	1653911926	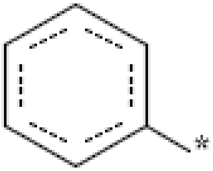	-0.487	12 out of 64

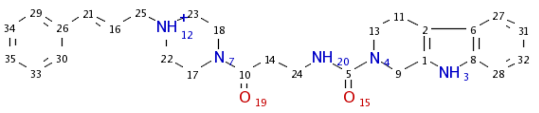

$C_{28}H_{34}N_5O_2$
Molecular Weight: 472.60186
ALogP: 1.567
Rotatable Bonds: 6
Acceptors: 2
Donors: 3

Model Prediction

Prediction: Carcinogen
Probability: 0.302
Enrichment: 0.903
Bayesian Score: 0.504

Prediction: Positive if the Bayesian score is above the estimated best cutoff value.
Probability: The esimated probability that the sample is in the category.
Enrichment: An estimate of enrichment, that is, the increased likelihood (versus random) of this sample being in the category.
Bayesian Score: The standard Laplacian-modified Bayesian score.

Structural Similar Compounds

Name	Etodolac	Tryptophan	Bromocriptine
Structure	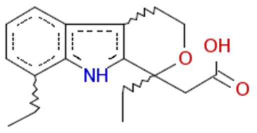	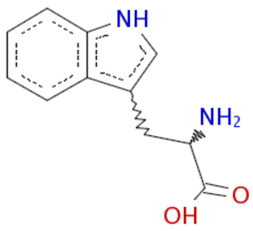	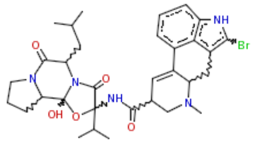
Actual Endpoint	Non-Carcinogen	Non-Carcinogen	Non-Carcinogen
Predicted Endpoint	Non-Carcinogen	Non-Carcinogen	Non-Carcinogen
Similarity	0.333	0.286	0.272
Reference	US FDA (Centre for Drug Eval.& Res./Off. Testing & Res.) Sept. 1997	US FDA (Centre for Drug Eval.& Res./Off. Testing & Res.) Sept. 1997	US FDA (Centre for Drug Eval.& Res./Off. Testing & Res.) Sept. 1997

Model Applicability

Missing features are fingerprint features in the query molecule not found in the training set.

1. Missing Feature: SCFP_12 1930718785 [*]C[NH+](C[*])C[*]
2. Missing Feature: SCFP_12 -1396915742 [*]CC[NH+]([*])[*]
3. Missing Feature: SCFP_12 -1396885951 [*][NH+]([*])CC=[*]

Feature Contribution

Top features for Carcinogen				
**Fingerprint**	**Bit**	**Structure/Smiles**	**Score**	**Carcinogen in training set**
SCFP_12	1651620003	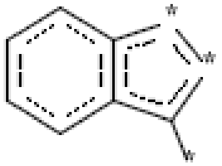	0.657	7 out of 10

SCFP_12	1962031760	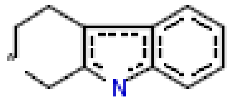	0.610	2 out of 2
SCFP_12	-1379673609	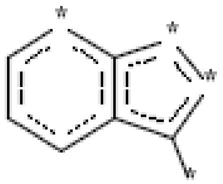	0.541	11 out of 19
**Top Features for Non-Carcinogen**				
**Fingerprint**	**Bit**	**Structure/Smiles**	**Score**	**Carcinogen in training set**
SCFP_12	-1849236245	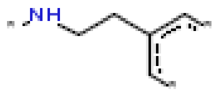	-0.815	0 out of 4
SCFP_12	-1343150366	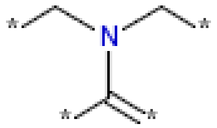	-0.644	3 out of 21
SCFP_12	1653911926	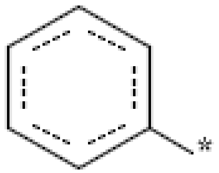	-0.487	12 out of 64

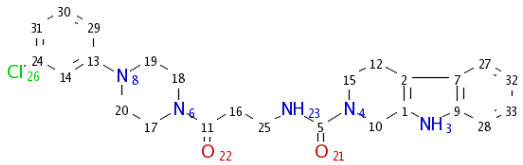

$C_{25}H_{28}ClN_5O_2$
Molecular Weight: 465.97512
ALogP: 3.413
Rotatable Bonds: 4
Acceptors: 3
Donors: 2

Model Prediction

Prediction: Non-Carcinogen
Probability: 0.252
Enrichment: 0.754
Bayesian Score: -3.348

Prediction: Positive if the Bayesian score is above the estimated best cutoff value.
Probability: The esimated probability that the sample is in the category.
Enrichment: An estimate of enrichment, that is, the increased likelihood (versus random) of this sample being in the category.
Bayesian Score: The standard Laplacian-modified Bayesian score.

Structural Similar Compounds

Name	Temazepam	Doxefazepam	Amoxapine
Structure	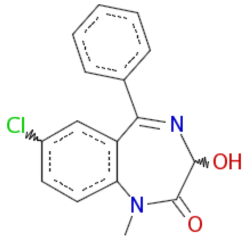	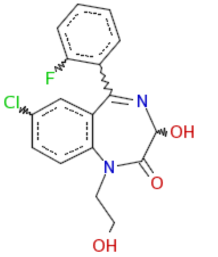	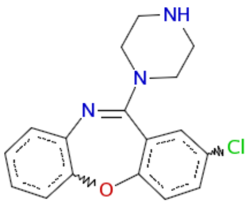
Actual Endpoint	Non-Carcinogen	Carcinogen	Carcinogen
Predicted Endpoint	Non-Carcinogen	Carcinogen	Carcinogen
Similarity	0.339	0.338	0.333
Reference	US FDA (Centre for Drug Eval.& Res./Off. Testing & Res.) Sept. 1997	US FDA (Centre for Drug Eval.& Res./Off. Testing & Res.) Sept. 1997	US FDA (Centre for Drug Eval.& Res./Off. Testing & Res.) Sept. 1997

Model Applicability

Missing features are fingerprint features in the query molecule not found in the training set.

1. All descriptors are in range.

Feature Contribution

Top features for Carcinogen

Fingerprint	Bit	Structure/Smiles	Score	Carcinogen in training set
SCFP_12	1651620003	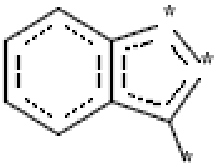	0.657	7 out of 10

SCFP_12	1962031760	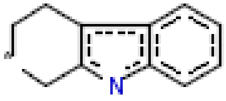	0.610	2 out of 2
SCFP_12	-1379673609	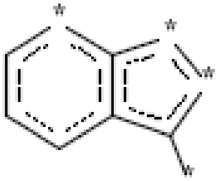	0.541	11 out of 19
**Top Features for Non-Carcinogen**				
**Fingerprint**	**Bit**	**Structure/Smiles**	**Score**	**Carcinogen in training set**
SCFP_12	-1849236245	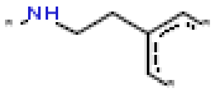	-0.815	0 out of 4
SCFP_12	52043406	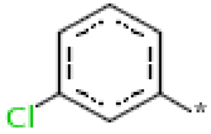	-0.665	0 out of 3
SCFP_12	-1343150366	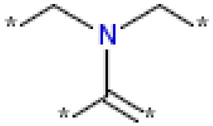	-0.644	3 out of 21

MOST ACTIVE.mol

TOPKAT_Rat_Male_FDA_None_vs_Carcinogen

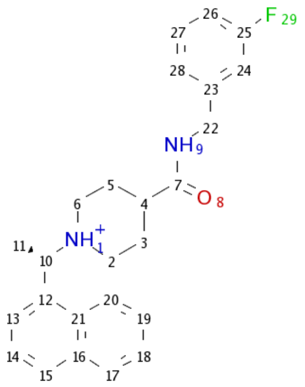

$C_{25}H_{28}FN_2O$
Molecular Weight: 391.50102
ALogP: 3.098
Rotatable Bonds: 5
Acceptors: 1
Donors: 2

Model Prediction

Prediction: Carcinogen
Probability: 0.356
Enrichment: 1.064
Bayesian Score: 3.163

Prediction: Positive if the Bayesian score is above the estimated best cutoff value.
Probability: The esimated probability that the sample is in the category.
Enrichment: An estimate of enrichment, that is, the increased likelihood (versus random) of this sample being in the category.
Bayesian Score: The standard Laplacian-modified Bayesian score.

Structural Similar Compounds

Name	Bupropion	Chlorpropamide	Phenobarbital
Structure	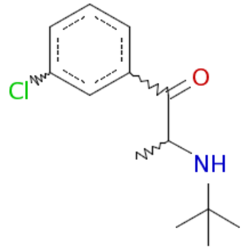	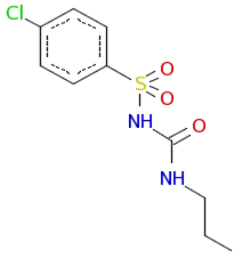	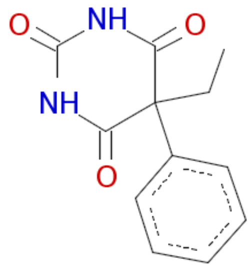
Actual Endpoint	Non-Carcinogen	Non-Carcinogen	Carcinogen
Predicted Endpoint	Non-Carcinogen	Non-Carcinogen	Carcinogen
Similarity	0.362	0.327	0.311
Reference	US FDA (Centre for Drug Eval.& Res./Off. Testing & Res.) Sept. 1997	US FDA (Centre for Drug Eval.& Res./Off. Testing & Res.) Sept. 1997	US FDA (Centre for Drug Eval.& Res./Off. Testing & Res.) Sept. 1997

Model Applicability

Missing features are fingerprint features in the query molecule not found in the training set.

1. Missing Feature: SCFP_12 1930718785 [*]C[NH+](C[*])C[*]
2. Missing Feature: SCFP_12 -1396915742 [*]CC[NH+]([*])[*]
3. Missing Feature: SCFP_12 -1688664082 [*][NH+]([*])[C@H](C)[c](:[*]):[*]

Feature Contribution

Top features for Carcinogen				
**Fingerprint**	**Bit**	**Structure/Smiles**	**Score**	**Carcinogen in training set**
SCFP_12	1651620003	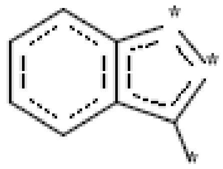	0.657	7 out of 10

SCFP_12	-1379673609	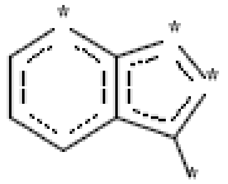	0.541	11 out of 19
SCFP_12	2098257782	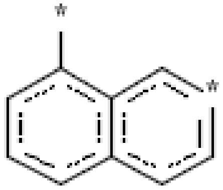	0.433	2 out of 3
**Top Features for Non-Carcinogen**				
**Fingerprint**	**Bit**	**Structure/Smiles**	**Score**	**Carcinogen in training set**
SCFP_12	11	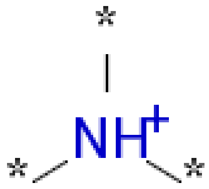	-0.274	0 out of 1
SCFP_12	-971326317	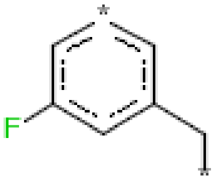	-0.274	0 out of 1
SCFP_12	-587479743	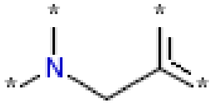	-0.274	0 out of 1

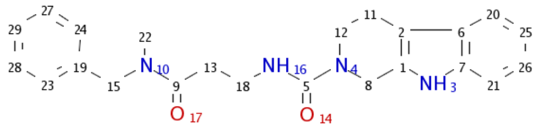

$C_{23}H_{26}N_4O_2$
Molecular Weight: 390.47814
ALogP: 2.702
Rotatable Bonds: 5
Acceptors: 2
Donors: 2

Model Prediction

Prediction: Single-Carcinogen
Probability: 0.513
Enrichment: 1.240
Bayesian Score: -2.039

Prediction: Positive if the Bayesian score is above the estimated best cutoff value.
Probability: The esimated probability that the sample is in the category.
Enrichment: An estimate of enrichment, that is, the increased likelihood (versus random) of this sample being in the category.
Bayesian Score: The standard Laplacian-modified Bayesian score.

Structural Similar Compounds

Name	Deserpidine	Reserpine	Prilocaine
Structure	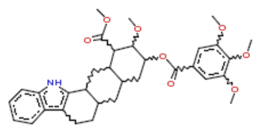	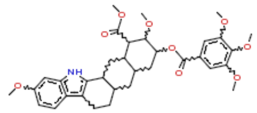	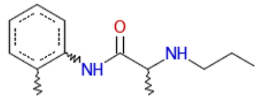
Actual Endpoint	Single-Carcinogen	Multiple-Carcinogen	Multiple-Carcinogen
Predicted Endpoint	Multiple-Carcinogen	Multiple-Carcinogen	Multiple-Carcinogen
Similarity	0.304	0.264	0.255
Reference	US FDA (Centre for Drug Eval.& Res./Off. Testing & Res.) Sept. 1997	US FDA (Centre for Drug Eval.& Res./Off. Testing & Res.) Sept. 1997	US FDA (Centre for Drug Eval.& Res./Off. Testing & Res.) Sept. 1997

Model Applicability

Missing features are fingerprint features in the query molecule not found in the training set.

1. Missing Feature: SCFP_8 -587479743 [*]N([*])C[c](:[*]):[*]

Feature Contribution

Top features for Multiple-Carcinogen				
**Fingerprint**	**Bit**	**Structure/Smiles**	**Score**	**Multiple-Carcinogen in training set**
SCFP_8	-111024397	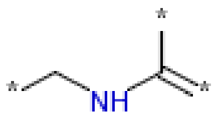	0.345	4 out of 7

SCFP_8	1256786467	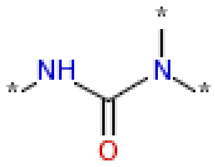	0.340	5 out of 9
SCFP_8	1256995004	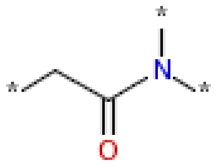	0.331	10 out of 19

Top Features for Single-Carcinogen

**Fingerprint**	**Bit**	**Structure/Smiles**	**Score**	**Multiple-Carcinogen in training set**
SCFP_8	1653911926	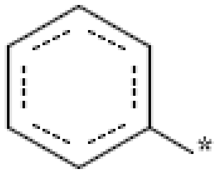	-0.985	1 out of 12
SCFP_8	-1381862798	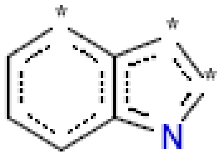	-0.572	1 out of 7
SCFP_8	1655199790	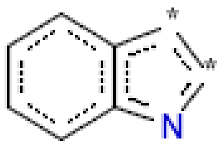	-0.342	1 out of 5

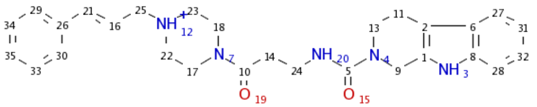

$C_{28}H_{34}N_5O_2$
Molecular Weight: 472.60186
ALogP: 1.567
Rotatable Bonds: 6
Acceptors: 2
Donors: 3

Model Prediction

Prediction: Single-Carcinogen
Probability: 0.514
Enrichment: 1.241
Bayesian Score: -1.983

Prediction: Positive if the Bayesian score is above the estimated best cutoff value.
Probability: The esimated probability that the sample is in the category.
Enrichment: An estimate of enrichment, that is, the increased likelihood (versus random) of this sample being in the category.
Bayesian Score: The standard Laplacian-modified Bayesian score.

Structural Similar Compounds

Name	Deserpidine	Reserpine	Prilocaine
Structure	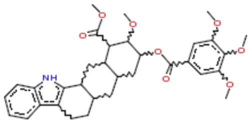	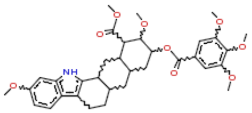	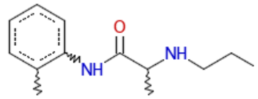
Actual Endpoint	Single-Carcinogen	Multiple-Carcinogen	Multiple-Carcinogen
Predicted Endpoint	Multiple-Carcinogen	Multiple-Carcinogen	Multiple-Carcinogen
Similarity	0.266	0.232	0.215
Reference	US FDA (Centre for Drug Eval.& Res./Off. Testing & Res.) Sept. 1997	US FDA (Centre for Drug Eval.& Res./Off. Testing & Res.) Sept. 1997	US FDA (Centre for Drug Eval.& Res./Off. Testing & Res.) Sept. 1997

Model Applicability

Missing features are fingerprint features in the query molecule not found in the training set.

1. Missing Feature: SCFP_8 11 [*][NH+]([*])[*]
2. Missing Feature: SCFP_8 -587479743 [*]N([*])C[c](:[*]):[*]
3. Missing Feature: SCFP_8 1930718785 [*]C[NH+](C[*])C[*]
4. Missing Feature: SCFP_8 -1396915742 [*]CC[NH+]([*])[*]
5. Missing Feature: SCFP_8 -1396885951 [*][NH+]([*])CC=[*]

Feature Contribution

Top features for Multiple-Carcinogen

Fingerprint	Bit	Structure/Smiles	Score	Multiple-Carcinogen in training set
SCFP_8	-1971137145	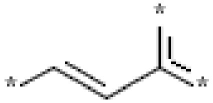	0.351	3 out of 5

SCFP_8	-111024397	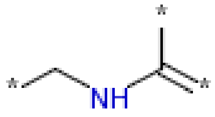	0.345	4 out of 7
SCFP_8	1256786467	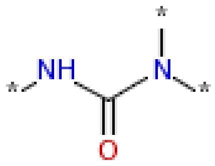	0.340	5 out of 9
**Top Features for Single-Carcinogen**				
**Fingerprint**	**Bit**	**Structure/Smiles**	**Score**	**Multiple-Carcinogen in training set**
SCFP_8	1653911926	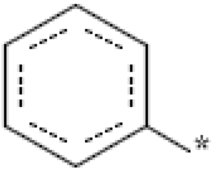	-0.985	1 out of 12
SCFP_8	-1381862798	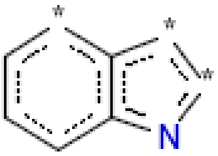	-0.572	1 out of 7
SCFP_8	1655199790	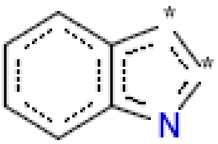	-0.342	1 out of 5

MOST ACTIVE.mol

TOPKAT_Rat_Male_FDA_Single_vs_Multiple

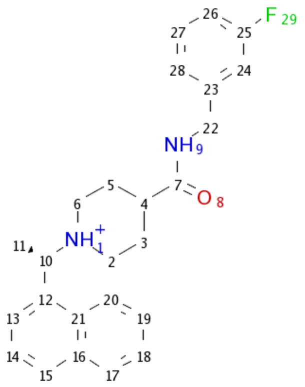

$C_{25}H_{28}FN_2O$
Molecular Weight: 391.50102
ALogP: 3.098
Rotatable Bonds: 5
Acceptors: 1
Donors: 2

Model Prediction

Prediction: Multiple-Carcinogen
Probability: 0.531
Enrichment: 1.282
Bayesian Score: 0.663

Prediction: Positive if the Bayesian score is above the estimated best cutoff value.
Probability: The esimated probability that the sample is in the category.
Enrichment: An estimate of enrichment, that is, the increased likelihood (versus random) of this sample being in the category.
Bayesian Score: The standard Laplacian-modified Bayesian score.

Structural Similar Compounds

Name	Phenobarbital	Sertraline	Oxazepam
Structure	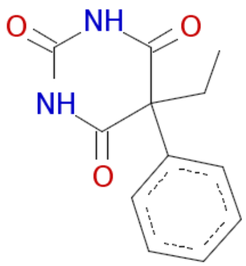	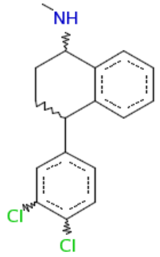	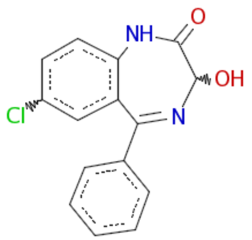
Actual Endpoint	Single-Carcinogen	Single-Carcinogen	Single-Carcinogen
Predicted Endpoint	Single-Carcinogen	Single-Carcinogen	Single-Carcinogen
Similarity	0.311	0.306	0.296
Reference	US FDA (Centre for Drug Eval.& Res./Off. Testing & Res.) Sept. 1997	US FDA (Centre for Drug Eval.& Res./Off. Testing & Res.) Sept. 1997	US FDA (Centre for Drug Eval.& Res./Off. Testing & Res.) Sept. 1997

Model Applicability

Missing features are fingerprint features in the query molecule not found in the training set.

1. Missing Feature: SCFP_8 11 [*][NH+]([*])[*]
2. Missing Feature: SCFP_8 1930718785 [*]C[NH+](C[*])C[*]
3. Missing Feature: SCFP_8 -1396915742 [*]CC[NH+]([*])[*]
4. Missing Feature: SCFP_8 -1688664082 [*][NH+]([*])[C@H](C)[c](:[*]):[*]
5. Missing Feature: SCFP_8 -587479743 [*]N([*])C[c](:[*]):[*]

Feature Contribution

Top features for Multiple-Carcinogen

Fingerprint	Bit	Structure/Smiles	Score	Multiple-Carcinogen in training set
SCFP_8	-1633424324	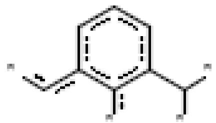	0.383	1 out of 1

SCFP_8	-168480298	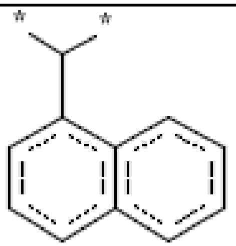	0.383	1 out of 1
SCFP_8	2094857464	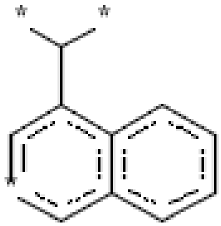	0.383	1 out of 1
**Top Features for Single-Carcinogen**				
**Fingerprint**	**Bit**	**Structure/Smiles**	**Score**	**Multiple-Carcinogen in training set**
SCFP_8	-730654023	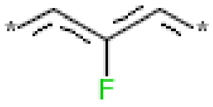	-0.463	1 out of 6
SCFP_8	-1794884847	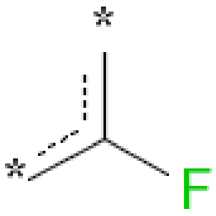	-0.463	1 out of 6
SCFP_8	-1381307546	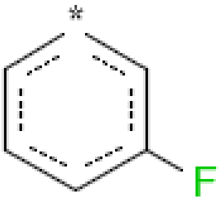	-0.463	1 out of 6

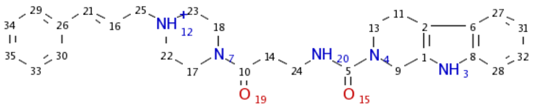

$C_{28}H_{34}N_5O_2$

Molecular Weight: 472.60186

ALogP: 1.567

Rotatable Bonds: 6

Acceptors: 2

Donors: 3

Model Prediction

Prediction: Mild

Probability: 0.041

Enrichment: 0.112

Bayesian Score: -13.395

Prediction: Positive if the Bayesian score is above the estimated best cutoff value.
Probability: The esimated probability that the sample is in the category.
Enrichment: An estimate of enrichment, that is, the increased likelihood (versus random) of this sample being in the category.
Bayesian Score: The standard Laplacian-modified Bayesian score.

Structural Similar Compounds

Name	Isoquinoline, 1,2,3,4-tetrahydro-2-octanoyl-	Cinnamic acid, phenethyl ester	Cinnamyl alcohol, propionate
Structure	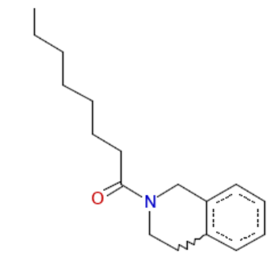	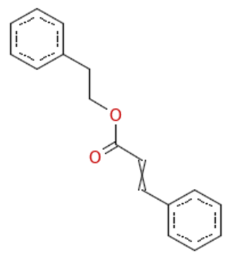	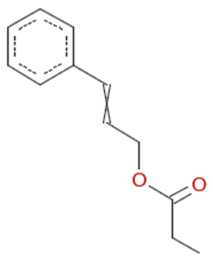
Actual Endpoint	Moderate_Severe	Mild	Mild
Predicted Endpoint	Moderate_Severe	Mild	Mild
Similarity	0.283	0.281	0.259
Reference	NTIS** National Technical Information Service. (Springfield, VA 22161) Forme rly U.S. Clearinghouse for Scientific & Technical Information. Volume(issue)/pag e/year: AD-A042-527	FCTXAV Food and Cosmetics Toxicology. (London, UK) V.1-19, 1963-81. For pu blisher information, see FCTOD7. Volume(issue)/page/year: 16,845,1978	FCTXAV Food and Cosmetics Toxicology. (London, UK) V.1-19, 1963-81. For pu blisher information, see FCTOD7. Volume(issue)/page/year: 12,859,1974

Model Applicability

Missing features are fingerprint features in the query molecule not found in the training set.

1. Missing Feature: FCFP_10 10 [*][NH+]([*])[*]
2. Missing Feature: FCFP_10 203707511 [*]C[c]1:n:[*]:[*]:[c]:1[*]
3. Missing Feature: FCFP_10 -1853714334 [*]C[NH+](C[*])C[*]
4. Missing Feature: FCFP_10 1155241219 [*]CC[NH+]([*])[*]

Feature Contribution

Top features for Moderate_Severe				
**Fingerprint**	**Bit**	**Structure/Smiles**	**Score**	**Moderate_Severe in training set**

FCFP_10	206055935	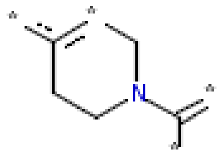	0.385	1 out of 1
FCFP_10	451847724	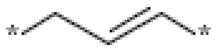	0.255	101 out of 216
FCFP_10	907007053	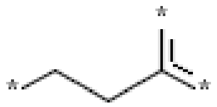	0.243	27 out of 58
**Top Features for Mild**				
**Fingerprint**	**Bit**	**Structure/Smiles**	**Score**	**Moderate_Severe in training set**
FCFP_10	-885550502	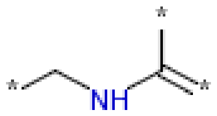	-1.052	2 out of 21
FCFP_10	307419094	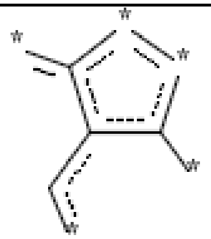	-0.917	2 out of 18

FCFP_10	-1320007763	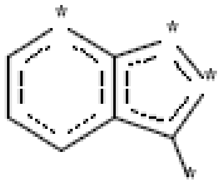	-0.761	2 out of 15

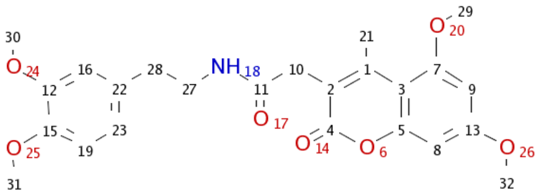

$C_{24}H_{27}NO_7$
Molecular Weight: 441.47368
ALogP: 3.388
Rotatable Bonds: 9
Acceptors: 7
Donors: 1

Structural Similar Compounds

Name	Phenol, 4-allyl-2-methoxy-, acetate	Acetic acid, phenyl-, 4-allyl-2-methoxyphenyl ester	2-Propanone, 1-(p-methoxyphenyl)-
Structure	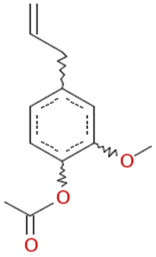	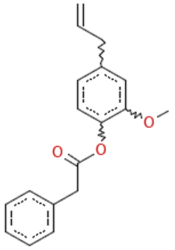	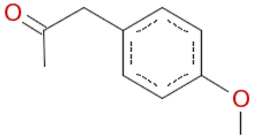
Actual Endpoint	Irritant	Irritant	Irritant
Predicted Endpoint	Irritant	Irritant	Non-Irritant
Similarity	0.463	0.419	0.410
Reference	FCTXAV Food and Cosmetics Toxicology. (London, UK) V.1-19, 1963-81. For pu blisher information, see FCTOD7. Volume(issue)/page/year: 12,877,1974	FCTXAV Food and Cosmetics Toxicology. (London, UK) V.1-19, 1963-81. For pu blisher information, see FCTOD7. Volume(issue)/page/year: 16,753,1978	FCTXAV Food and Cosmetics Toxicology. (London, UK) V.1-19, 1963-81. For pu blisher information, see FCTOD7. Volume(issue)/page/year: 17,857,1979

Model Prediction

Prediction: Non-Irritant
Probability: 0.963
Enrichment: 1.046
Bayesian Score: -1.257

Prediction: Positive if the Bayesian score is above the estimated best cutoff value.
Probability: The esimated probability that the sample is in the category.
Enrichment: An estimate of enrichment, that is, the increased likelihood (versus random) of this sample being in the category.
Bayesian Score: The standard Laplacian-modified Bayesian score.

Model Applicability

Missing features are fingerprint features in the query molecule not found in the training set.

1. All descriptors are in range.

Feature Contribution

Top features for Irritant

Fingerprint	Bit	Structure/Smiles	Score	Irritant in training set
FCFP_12	-1979033238	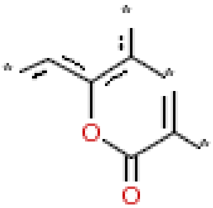	0.084	19 out of 19

FCFP_12	436886043	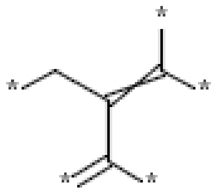	0.080	129 out of 130
FCFP_12	-1038421835	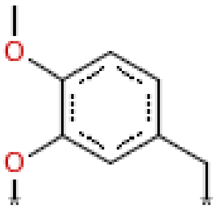	0.079	9 out of 9

Top Features for Non-Irritant

Fingerprint	Bit	Structure/Smiles	Score	Irritant in training set
FCFP_12	-2002900105	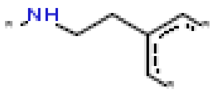	-0.650	0 out of 1
FCFP_12	566058135	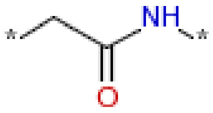	-0.367	13 out of 21
FCFP_12	-547731249	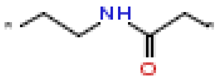	-0.222	2 out of 3

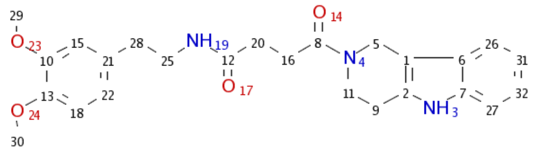

$C_{25}H_{29}N_3O_4$

Molecular Weight: 435.51546

ALogP: 2.831

Rotatable Bonds: 8

Acceptors: 4

Donors: 2

Model Prediction

Prediction: Non-Irritant

Probability: 0.952

Enrichment: 1.033

Bayesian Score: -1.727

Prediction: Positive if the Bayesian score is above the estimated best cutoff value.
Probability: The esimated probability that the sample is in the category.
Enrichment: An estimate of enrichment, that is, the increased likelihood (versus random) of this sample being in the category.
Bayesian Score: The standard Laplacian-modified Bayesian score.

Structural Similar Compounds

Name	Isoquinoline, 1,2,3,4-tetrahydro-2-octanoyl-	Acetic acid, phenyl-, 4-allyl-2-methoxyphenyl ester	Acetic acid, phenyl-, p-methoxybenzyl ester
Structure	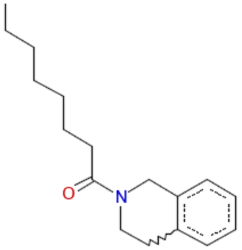	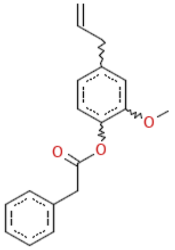	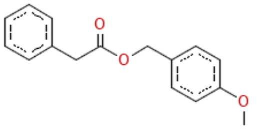
Actual Endpoint	Irritant	Irritant	Irritant
Predicted Endpoint	Irritant	Irritant	Irritant
Similarity	0.358	0.333	0.321
Reference	NTIS** National Technical Information Service. (Springfield, VA 22161) Forme rly U.S. Clearinghouse for Scientific & Technical Information. Volume(issue)/pag e/year: AD-A042-527	FCTXAV Food and Cosmetics Toxicology. (London, UK) V.1-19, 1963-81. For pu blisher information, see FCTOD7. Volume(issue)/page/year: 16,753,1978	FCTXAV Food and Cosmetics Toxicology. (London, UK) V.1-19, 1963-81. For pu blisher information, see FCTOD7. Volume(issue)/page/year: 18,651,1980

Model Applicability

Missing features are fingerprint features in the query molecule not found in the training set.

1. Missing Feature: FCFP_12 203707511 [*]C[c]1:n:[*]:[*]:[c]:1[*]

Feature Contribution

Top features for Irritant				
Fingerprint	Bit	Structure/Smiles	Score	Irritant in training set

FCFP_12	-1038421835	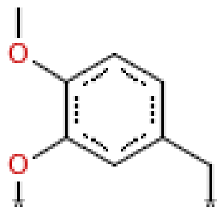	0.079	9 out of 9
FCFP_12	1985089045	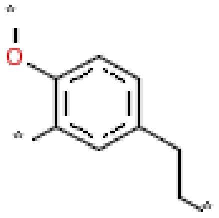	0.078	8 out of 8
FCFP_12	-917072037	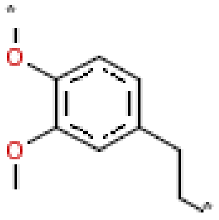	0.073	5 out of 5
**Top Features for Non-Irritant**				
**Fingerprint**	**Bit**	**Structure/Smiles**	**Score**	**Irritant in training set**
FCFP_12	-2002900105	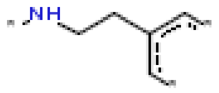	-0.650	0 out of 1
FCFP_12	566058135	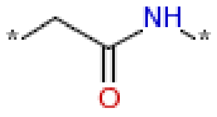	-0.367	13 out of 21

FCFP_12	-357825883	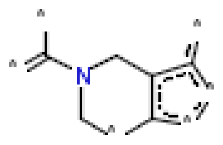	-0.347	1 out of 2

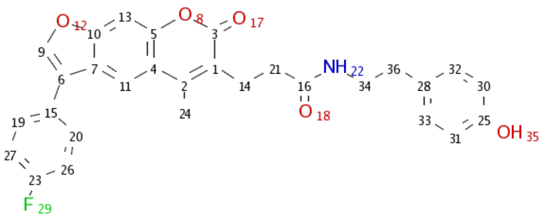

$C_{29}H_{24}FNO_5$
Molecular Weight: 485.50296
ALogP: 5.695
Rotatable Bonds: 7
Acceptors: 4
Donors: 2

Model Prediction

Prediction: Non-Irritant
Probability: 0.891
Enrichment: 0.967
Bayesian Score: -2.818

Prediction: Positive if the Bayesian score is above the estimated best cutoff value.
Probability: The esimated probability that the sample is in the category.
Enrichment: An estimate of enrichment, that is, the increased likelihood (versus random) of this sample being in the category.
Bayesian Score: The standard Laplacian-modified Bayesian score.

Structural Similar Compounds

Name	2-Butanone, 4-(4-hydroxy-3-methoxyphenyl)-	Phenol, 2-methoxy-4-propyl-	Acetic acid, 2-chloro-5-nitrophenyl ester
Structure	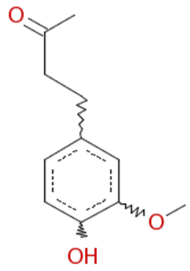	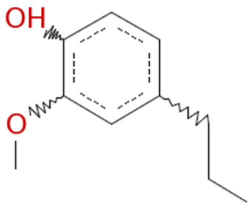	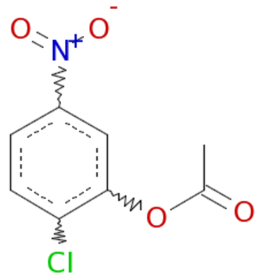
Actual Endpoint	Irritant	Irritant	Irritant
Predicted Endpoint	Irritant	Irritant	Non-Irritant
Similarity	0.274	0.267	0.262
Reference	FCTOD7 Food and Chemical Toxicology. (Pergamon Press Inc., Maxwell House, Fa irview Park, Elmsford, NY 10523) V.20- 1982- Volume(issue)/page/year: 20,851 ,1982	FCTOD7 Food and Chemical Toxicology. (Pergamon Press Inc., Maxwell House, Fa irview Park, Elmsford, NY 10523) V.20- 1982- Volume(issue)/page/year: 20,671 ,1982	28ZPAK -,92,72

Model Applicability

Missing features are fingerprint features in the query molecule not found in the training set.

1. All descriptors are in range.

Feature Contribution

Top features for Irritant

Fingerprint	Bit	Structure/Smiles	Score	Irritant in training set
FCFP_12	-2083883923	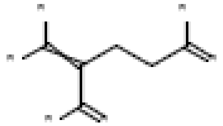	0.087	51 out of 51

FCFP_12	-1979033238	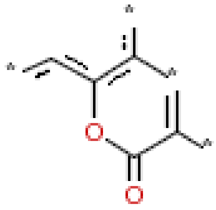	0.084	19 out of 19
FCFP_12	-124655670	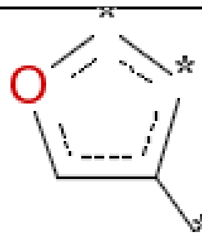	0.082	13 out of 13

Top Features for Non-Irritant

**Fingerprint**	**Bit**	**Structure/Smiles**	**Score**	**Irritant in training set**
FCFP_12	-2002900105	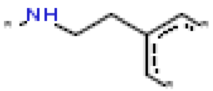	-0.650	0 out of 1
FCFP_12	566058135	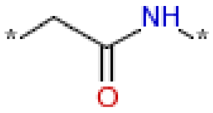	-0.367	13 out of 21
FCFP_12	-547731249	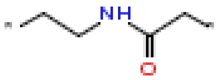	-0.222	2 out of 3

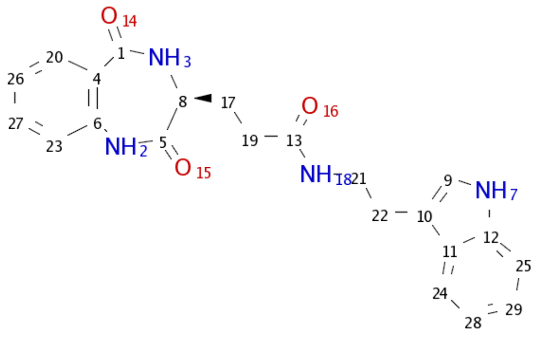

$C_{22}H_{22}N_4O_3$
Molecular Weight: 390.43508
ALogP: 1.807
Rotatable Bonds: 6
Acceptors: 3
Donors: 4

Model Prediction

Prediction: Non-Irritant
Probability: 0.457
Enrichment: 0.496
Bayesian Score: -4.734

Prediction: Positive if the Bayesian score is above the estimated best cutoff value.
Probability: The esimated probability that the sample is in the category.
Enrichment: An estimate of enrichment, that is, the increased likelihood (versus random) of this sample being in the category.
Bayesian Score: The standard Laplacian-modified Bayesian score.

Structural Similar Compounds

Name	Glutamic acid, N-(p-(methylamino)benzoyl)-, sodium salt	Acetanilide, 4'-(2-hydroxyethylsulfonyl)-	8-Methylamino-4-hydroxy-2-naphthalene sulfonic acid
Structure	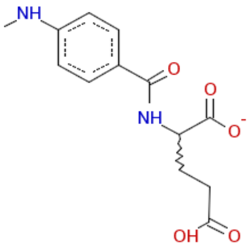	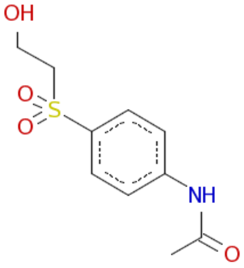	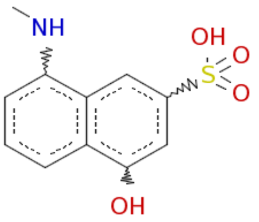
Actual Endpoint	Irritant	Non-Irritant	Non-Irritant
Predicted Endpoint	Non-Irritant	Non-Irritant	Non-Irritant
Similarity	0.316	0.288	0.273
Reference	FCTOD7 Food and Chemical Toxicology. (Pergamon Press Inc., Maxwell House, Fa irview Park, Elmsford, NY 10523) V.20- 1982- Volume(issue)/page/year: 20,563 ,1982	28ZPAK -,533,72	28ZPAK -,190,72

Model Applicability

Missing features are fingerprint features in the query molecule not found in the training set.

1. Missing Feature: FCFP_12 1618184456 [*][c]1:[*]:[*]:n:c:1

Feature Contribution

Top features for Irritant				
**Fingerprint**	**Bit**	**Structure/Smiles**	**Score**	**Irritant in training set**
FCFP_12	-1549103449	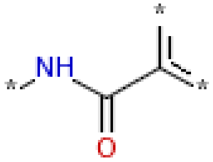	0.073	5 out of 5

FCFP_12	307448885	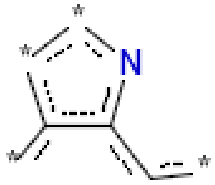	0.066	3 out of 3
FCFP_12	2005402822	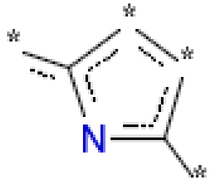	0.066	3 out of 3
**Top Features for Non-Irritant**				
**Fingerprint**	**Bit**	**Structure/Smiles**	**Score**	**Irritant in training set**
FCFP_12	1175665944	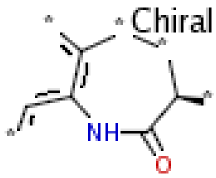	-1.020	2 out of 8
FCFP_12	-2002900105	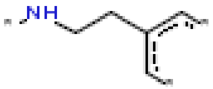	-0.650	0 out of 1
FCFP_12	1294255210	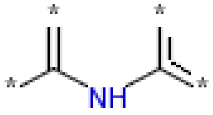	-0.486	12 out of 22

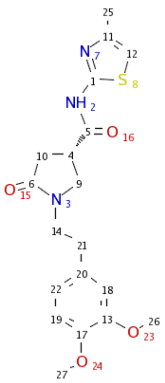

$C_{19}H_{23}N_3O_4S$
Molecular Weight: 389.46862
ALogP: 1.461
Rotatable Bonds: 7
Acceptors: 5
Donors: 1

Model Prediction

Prediction: Non-Irritant

Probability: 0.969

Enrichment: 1.052

Bayesian Score: -0.907

Prediction: Positive if the Bayesian score is above the estimated best cutoff value.
Probability: The esimated probability that the sample is in the category.
Enrichment: An estimate of enrichment, that is, the increased likelihood (versus random) of this sample being in the category.
Bayesian Score: The standard Laplacian-modified Bayesian score.

Structural Similar Compounds

Name	Phenol, 4-allyl-2-methoxy-, acetate	Isoquinoline, 1,2,3,4-tetrahydro-2-octanoyl-	Acetic acid, phenyl-, 4-allyl-2-methoxyphenyl ester
Structure	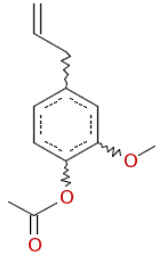	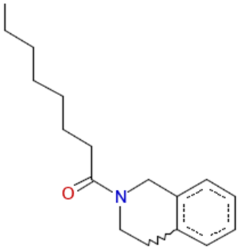	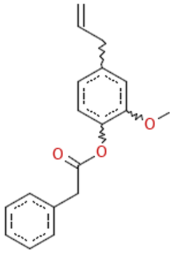
Actual Endpoint	Irritant	Irritant	Irritant
Predicted Endpoint	Irritant	Irritant	Irritant
Similarity	0.347	0.340	0.340
Reference	FCTXAV Food and Cosmetics Toxicology. (London, UK) V.1-19, 1963-81. For pu blisher information, see FCTOD7. Volume(issue)/page/year: 12,877,1974	NTIS** National Technical Information Service. (Springfield, VA 22161) Forme rly U.S. Clearinghouse for Scientific & Technical Information. Volume(issue)/pag e/year: AD-A042-527	FCTXAV Food and Cosmetics Toxicology. (London, UK) V.1-19, 1963-81. For pu blisher information, see FCTOD7. Volume(issue)/page/year: 16,753,1978

Model Applicability

Missing features are fingerprint features in the query molecule not found in the training set.

1. All descriptors are in range.

Feature Contribution

Top features for Irritant				
Fingerprint	Bit	Structure/Smiles	Score	Irritant in training set

FCFP_12	-124655670	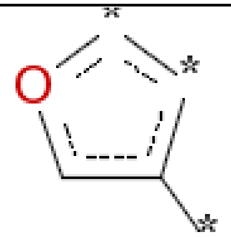	0.082	13 out of 13
FCFP_12	262046847	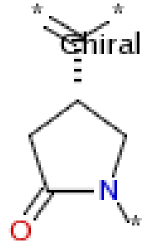	0.082	13 out of 13
FCFP_12	-1539132615	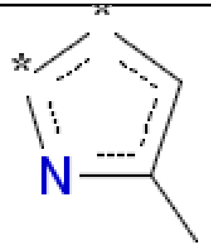	0.079	9 out of 9
**Top Features for Non-Irritant**				
**Fingerprint**	**Bit**	**Structure/Smiles**	**Score**	**Irritant in training set**
FCFP_12	1294255210	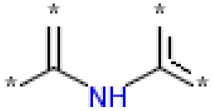	-0.486	12 out of 22
FCFP_12	566058135	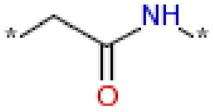	-0.367	13 out of 21

FCFP_12	-1151914249	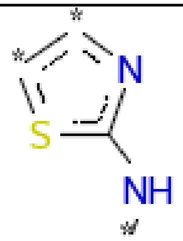	-0.109	4 out of 5

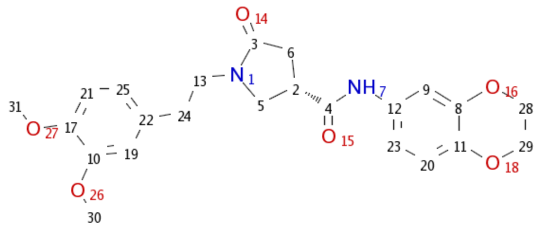

$C_{23}H_{26}N_2O_6$
Molecular Weight: 426.46234
ALogP: 1.967
Rotatable Bonds: 7
Acceptors: 6
Donors: 1

Model Prediction

Prediction: Non-Irritant
Probability: 0.001
Enrichment: 0.001
Bayesian Score: -8.229

Prediction: Positive if the Bayesian score is above the estimated best cutoff value.
Probability: The esimated probability that the sample is in the category.
Enrichment: An estimate of enrichment, that is, the increased likelihood (versus random) of this sample being in the category.
Bayesian Score: The standard Laplacian-modified Bayesian score.

Structural Similar Compounds

Name	p-Acetophenetidide, 3'-(bis(2-hydroxyethyl)amino)-	p-Acetophenetidide, 3'-nitro-	Acetanilide, 3'-amino-4'-ethoxy-
Structure	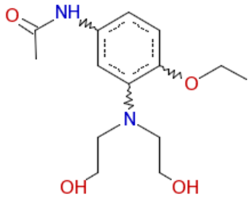	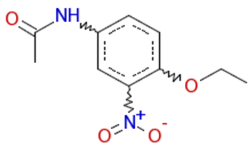	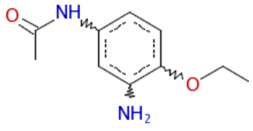
Actual Endpoint	Non-Irritant	Non-Irritant	Non-Irritant
Predicted Endpoint	Non-Irritant	Non-Irritant	Non-Irritant
Similarity	0.444	0.409	0.395
Reference	28ZPAK -,100,72	28ZPAK -,115,72	28ZPAK -,115,72

Model Applicability

Missing features are fingerprint features in the query molecule not found in the training set.

1. All descriptors are in range.

Feature Contribution

Top features for Irritant				
**Fingerprint**	**Bit**	**Structure/Smiles**	**Score**	**Irritant in training set**
FCFP_12	262046847	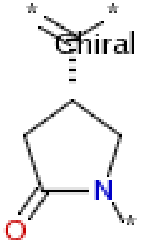	0.082	13 out of 13

FCFP_12	-1038421835	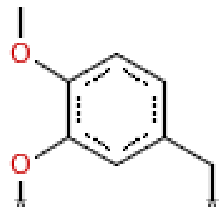	0.079	9 out of 9
FCFP_12	1985089045	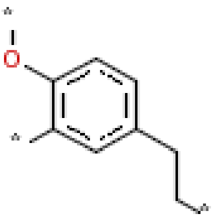	0.078	8 out of 8

Top Features for Non-Irritant

Fingerprint	Bit	Structure/Smiles	Score	Irritant in training set
FCFP_12	-789307649	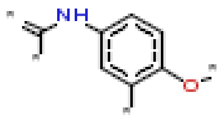	-1.539	0 out of 4
FCFP_12	1175665944	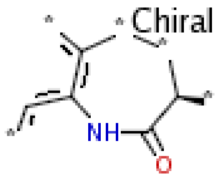	-1.020	2 out of 8
FCFP_12	-1838187238	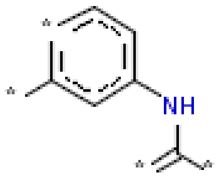	-0.692	5 out of 12

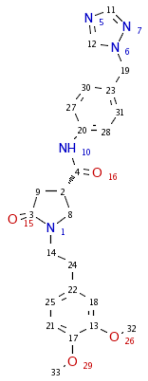

$C_{24}H_{27}N_5O_4$
Molecular Weight: 449.50228
ALogP: 1.731
Rotatable Bonds: 9
Acceptors: 6
Donors: 1

Model Prediction

Prediction: Non-Irritant
Probability: 0.648
Enrichment: 0.704
Bayesian Score: -4.154

Prediction: Positive if the Bayesian score is above the estimated best cutoff value.
Probability: The esimated probability that the sample is in the category.
Enrichment: An estimate of enrichment, that is, the increased likelihood (versus random) of this sample being in the category.
Bayesian Score: The standard Laplacian-modified Bayesian score.

Structural Similar Compounds

Name	Phenol, 4-allyl-2-methoxy-, acetate	Benzenesulfonamide, 4-amino-N-(5,6-dimethoxy-4-pyrimidinyl)-	p-Acetophenetidide, 3'-(bis(2-hydroxyethyl)amino)-
Structure	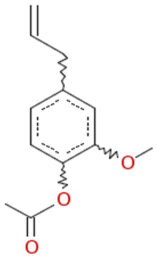	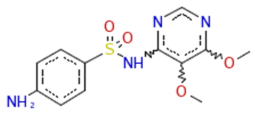	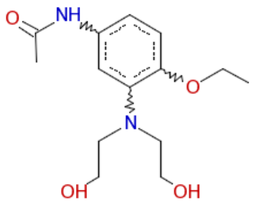
Actual Endpoint	Irritant	Irritant	Non-Irritant
Predicted Endpoint	Irritant	Non-Irritant	Non-Irritant
Similarity	0.321	0.317	0.316
Reference	FCTXAV Food and Cosmetics Toxicology. (London, UK) V.1-19, 1963-81. For pu blisher information, see FCTOD7. Volume(issue)/page/year: 12,877,1974	FCTXAV 14,307,76	28ZPAK -,100,72

Model Applicability

Missing features are fingerprint features in the query molecule not found in the training set.

1. Missing Feature: FCFP_12 906560188 [*]:[c](:[*])Cn(:[*]):[*]

Feature Contribution

Top features for Irritant

Fingerprint	Bit	Structure/Smiles	Score	Irritant in training set
FCFP_12	262046847	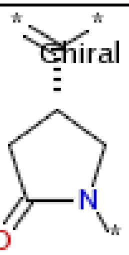	0.082	13 out of 13

FCFP_12	-1038421835	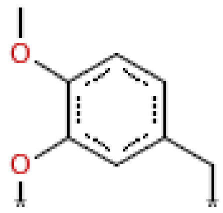	0.079	9 out of 9
FCFP_12	1985089045	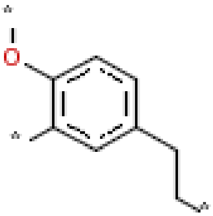	0.078	8 out of 8

Top Features for Non-Irritant

Fingerprint	Bit	Structure/Smiles	Score	Irritant in training set
FCFP_12	1175665944	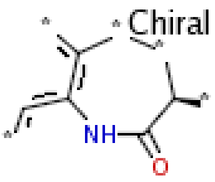	-1.020	2 out of 8
FCFP_12	-1838187238	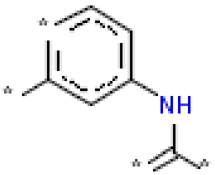	-0.692	5 out of 12
FCFP_12	-451043714	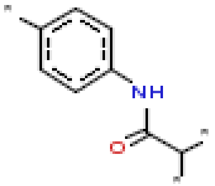	-0.650	0 out of 1

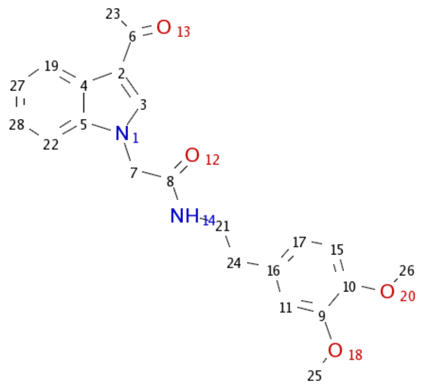

$C_{22}H_{24}N_2O_4$
Molecular Weight: 380.43696
ALogP: 3.001
Rotatable Bonds: 8
Acceptors: 4
Donors: 1

Model Prediction

Prediction: Non-Irritant
Probability: 0.954
Enrichment: 1.036
Bayesian Score: -1.641

Prediction: Positive if the Bayesian score is above the estimated best cutoff value.
Probability: The esimated probability that the sample is in the category.
Enrichment: An estimate of enrichment, that is, the increased likelihood (versus random) of this sample being in the category.
Bayesian Score: The standard Laplacian-modified Bayesian score.

Structural Similar Compounds

Name	Acetophenone, 4'-methoxy-	Phenol, 4-allyl-2-methoxy-, acetate	Acetic acid, phenyl-, 4-allyl-2-methoxyphenyl ester
Structure	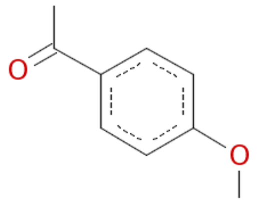	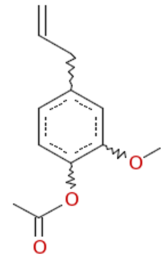	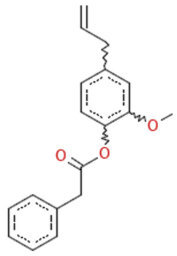
Actual Endpoint	Irritant	Irritant	Irritant
Predicted Endpoint	Irritant	Irritant	Irritant
Similarity	0.381	0.354	0.347
Reference	FCTXAV Food and Cosmetics Toxicology. (London, UK) V.1-19, 1963-81. For pu blisher information, see FCTOD7. Volume(issue)/page/year: 12,927,1974	FCTXAV Food and Cosmetics Toxicology. (London, UK) V.1-19, 1963-81. For pu blisher information, see FCTOD7. Volume(issue)/page/year: 12,877,1974	FCTXAV Food and Cosmetics Toxicology. (London, UK) V.1-19, 1963-81. For pu blisher information, see FCTOD7. Volume(issue)/page/year: 16,753,1978

Model Applicability

Missing features are fingerprint features in the query molecule not found in the training set.

1. All descriptors are in range.

Feature Contribution

Top features for Irritant

Fingerprint	Bit	Structure/Smiles	Score	Irritant in training set
FCFP_12	-124655670	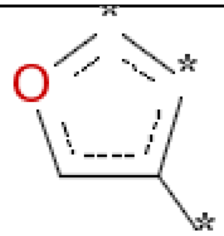	0.082	13 out of 13

FCFP_12	-1038421835	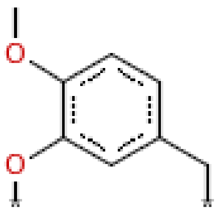	0.079	9 out of 9
FCFP_12	1985089045	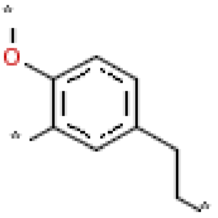	0.078	8 out of 8

Top Features for Non-Irritant

Fingerprint	Bit	Structure/Smiles	Score	Irritant in training set
FCFP_12	-2002900105	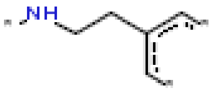	-0.650	0 out of 1
FCFP_12	566058135	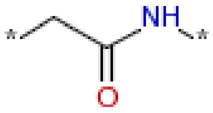	-0.367	13 out of 21
FCFP_12	-547731249	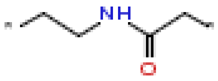	-0.222	2 out of 3

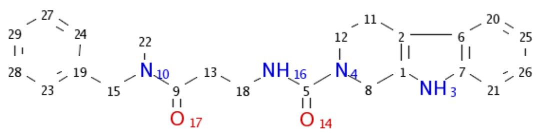

$C_{23}H_{26}N_4O_2$
Molecular Weight: 390.47814
ALogP: 2.702
Rotatable Bonds: 5
Acceptors: 2
Donors: 2

Model Prediction

Prediction: Non-Irritant
Probability: 0.957
Enrichment: 1.039
Bayesian Score: -1.535

Prediction: Positive if the Bayesian score is above the estimated best cutoff value.
Probability: The esimated probability that the sample is in the category.
Enrichment: An estimate of enrichment, that is, the increased likelihood (versus random) of this sample being in the category.
Bayesian Score: The standard Laplacian-modified Bayesian score.

Structural Similar Compounds

Name	Isoquinoline, 1,2,3,4-tetrahydro-2-octanoyl-	N-Benzyl-N-acdtyl-o-methyl cyclohexylamine	Methyl 2-hydroxy-4-phenyl butyrate
Structure	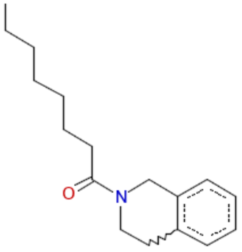	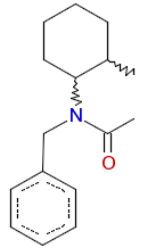	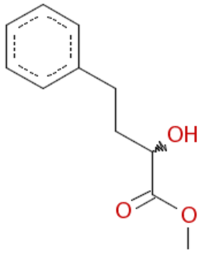
Actual Endpoint	Irritant	Non-Irritant	Non-Irritant
Predicted Endpoint	Irritant	Non-Irritant	Non-Irritant
Similarity	0.367	0.294	0.292
Reference	NTIS** National Technical Information Service. (Springfield, VA 22161) Forme rly U.S. Clearinghouse for Scientific & Technical Information. Volume(issue)/pag e/year: AD-A042-527	US ARMY	US ARMY

Model Applicability

Missing features are fingerprint features in the query molecule not found in the training set.

1. Missing Feature: FCFP_12 203707511 [*]C[c]1:n:[*]:[*]:[c]:1[*]

Feature Contribution

Top features for Irritant

Fingerprint	Bit	Structure/Smiles	Score	Irritant in training set
FCFP_12	2005402822	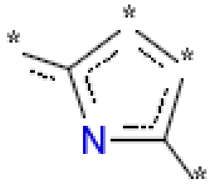	0.066	3 out of 3

FCFP_12	19	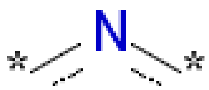	0.066	3 out of 3
FCFP_12	307448885	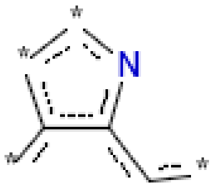	0.066	3 out of 3

Top Features for Non-Irritant

Fingerprint	Bit	Structure/Smiles	Score	Irritant in training set
FCFP_12	1910745113	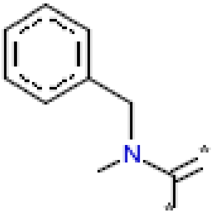	-0.650	0 out of 1
FCFP_12	-357825883	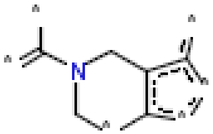	-0.347	1 out of 2
FCFP_12	-1320007763	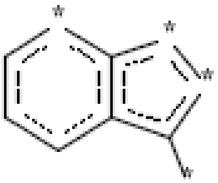	-0.089	20 out of 24

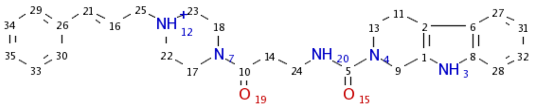

$C_{28}H_{34}N_5O_2$

Molecular Weight: 472.60186

ALogP: 1.567

Rotatable Bonds: 6

Acceptors: 2

Donors: 3

Model Prediction

Prediction: Irritant

Probability: 0.977

Enrichment: 1.061

Bayesian Score: -0.071

Prediction: Positive if the Bayesian score is above the estimated best cutoff value.
Probability: The esimated probability that the sample is in the category.
Enrichment: An estimate of enrichment, that is, the increased likelihood (versus random) of this sample being in the category.
Bayesian Score: The standard Laplacian-modified Bayesian score.

Structural Similar Compounds

Name	Isoquinoline, 1,2,3,4-tetrahydro-2-octanoyl-	Cinnamic acid, phenethyl ester	Cinnamyl alcohol, propionate
Structure	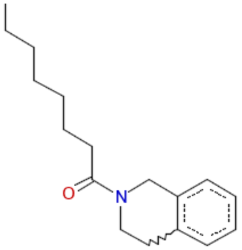	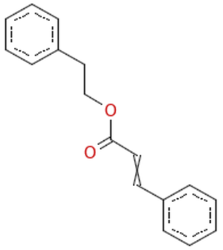	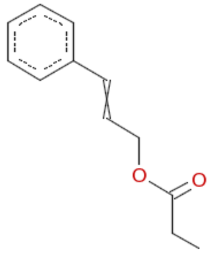
Actual Endpoint	Irritant	Irritant	Irritant
Predicted Endpoint	Irritant	Irritant	Irritant
Similarity	0.283	0.281	0.259
Reference	NTIS** National Technical Information Service. (Springfield, VA 22161) Forme rly U.S. Clearinghouse for Scientific & Technical Information. Volume(issue)/pag e/year: AD-A042-527	FCTXAV Food and Cosmetics Toxicology. (London, UK) V.1-19, 1963-81. For pu blisher information, see FCTOD7. Volume(issue)/page/year: 16,845,1978	FCTXAV Food and Cosmetics Toxicology. (London, UK) V.1-19, 1963-81. For pu blisher information, see FCTOD7. Volume(issue)/page/year: 12,859,1974

Model Applicability

Missing features are fingerprint features in the query molecule not found in the training set.

1. Missing Feature: FCFP_12 203707511 [*]C[c]1:n:[*]:[*]:[c]:1[*]
2. Missing Feature: FCFP_12 -1853714334 [*]C[NH+](C[*])C[*]
3. Missing Feature: FCFP_12 1155241219 [*]CC[NH+]([*])[*]

Feature Contribution

Top features for Irritant				
**Fingerprint**	**Bit**	**Structure/Smiles**	**Score**	**Irritant in training set**

FCFP_12	-146015125	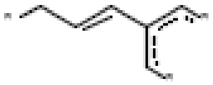	0.085	24 out of 24
FCFP_12	2011169140	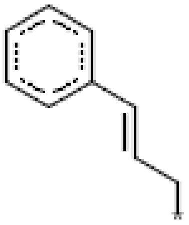	0.083	15 out of 15
FCFP_12	451847724	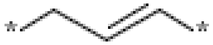	0.074	270 out of 274
**Top Features for Non-Irritant**				
**Fingerprint**	**Bit**	**Structure/Smiles**	**Score**	**Irritant in training set**
FCFP_12	-1320007763	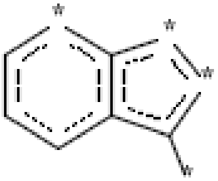	-0.089	20 out of 24
FCFP_12	1618154665	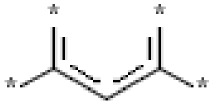	-0.084	412 out of 490

FCFP_12	16	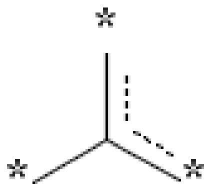	-0.084	423 out of 503

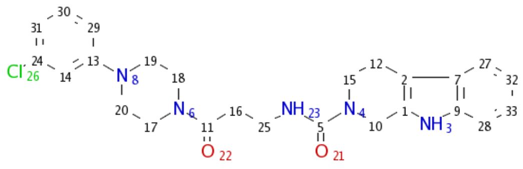

$C_{25}H_{28}ClN_5O_2$
Molecular Weight: 465.97512
ALogP: 3.413
Rotatable Bonds: 4
Acceptors: 3
Donors: 2

Model Prediction

Prediction: Non-Irritant
Probability: 0.962
Enrichment: 1.044
Bayesian Score: -1.329

Prediction: Positive if the Bayesian score is above the estimated best cutoff value.
Probability: The esimated probability that the sample is in the category.
Enrichment: An estimate of enrichment, that is, the increased likelihood (versus random) of this sample being in the category.
Bayesian Score: The standard Laplacian-modified Bayesian score.

Structural Similar Compounds

Name	Maleimide, N-(p-chlorophenyl)-	C.I. Fluorescent Brightening Agent 24	Piperazine, 1-phenyl-
Structure	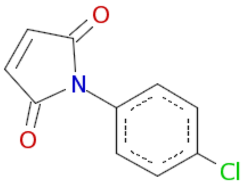	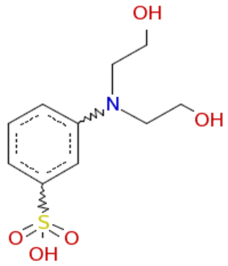	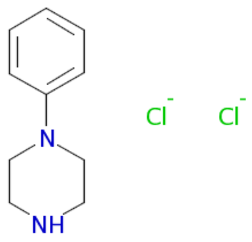
Actual Endpoint	Irritant	Irritant	Irritant
Predicted Endpoint	Non-Irritant	Non-Irritant	Irritant
Similarity	0.296	0.281	0.278
Reference	SCCUR* Shell Chemical Company. Unpublished Report. (2401 Crow Canyon Rd., S an Romon, CA 94583) Volume(issue)/page/year: -,7,1961	MVCRB3 MVC-Report. (Stockholm, Sweden) No.1-2, 1972-73. Discontinued. Volu me(issue)/page/year: 2,193,1973	85JCAE "Prehled Prumyslove Toxikologie; Organicke Latky," Marhold, J., Prague , Czechoslovakia, Avicenum, 1986 Volume(issue)/page/year: -,868,1986

Model Applicability

Missing features are fingerprint features in the query molecule not found in the training set.

1. Missing Feature: FCFP_12 203707511 [*]C[c]1:n:[*]:[*]:[c]:1[*]

Feature Contribution

Top features for Irritant

Fingerprint	Bit	Structure/Smiles	Score	Irritant in training set
FCFP_12	1604677718	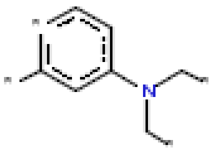	0.078	8 out of 8

FCFP_12	-1428172766	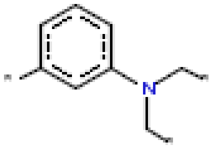	0.077	7 out of 7
FCFP_12	19	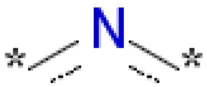	0.066	3 out of 3

Top Features for Non-Irritant

Fingerprint	Bit	Structure/Smiles	Score	Irritant in training set
FCFP_12	2134556787	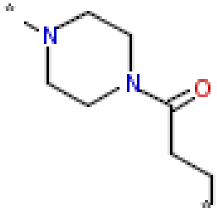	-0.347	1 out of 2
FCFP_12	367998008	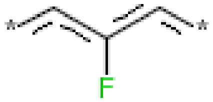	-0.129	61 out of 76
FCFP_12	71476542	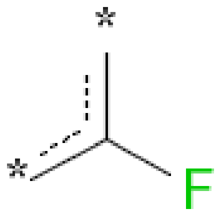	-0.120	64 out of 79

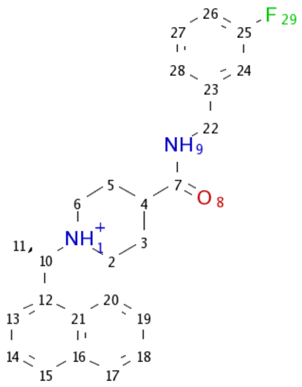

$C_{25}H_{28}FN_2O$
Molecular Weight: 391.50102
ALogP: 3.098
Rotatable Bonds: 5
Acceptors: 1
Donors: 2

Model Prediction

Prediction: Non-Irritant
Probability: 0.913
Enrichment: 0.991
Bayesian Score: -2.543

Prediction: Positive if the Bayesian score is above the estimated best cutoff value.
Probability: The esimated probability that the sample is in the category.
Enrichment: An estimate of enrichment, that is, the increased likelihood (versus random) of this sample being in the category.
Bayesian Score: The standard Laplacian-modified Bayesian score.

Structural Similar Compounds

Name	1'-Acetonaphthone	Sulfone, p-chlorophenyl methyl	Sulfoxide, p-chlorophenyl methyl
Structure	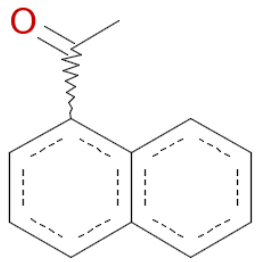	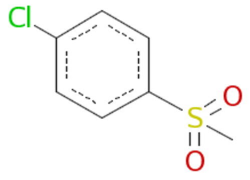	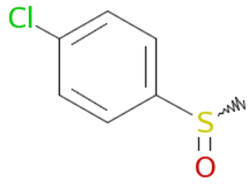
Actual Endpoint	Irritant	Irritant	Irritant
Predicted Endpoint	Non-Irritant	Non-Irritant	Non-Irritant
Similarity	0.310	0.310	0.310
Reference	FCTOD7 Food and Chemical Toxicology. (Pergamon Press Inc., Maxwell House, Fa irview Park, Elmsford, NY 10523) V.20- 1982- Volume(issue)/page/year: 20,755 ,1982	NTIS** National Technical Information Service. (Springfield, VA 22161) Forme rly U.S. Clearinghouse for Scientific & Technical Information. Volume(issue)/pag e/year: AD-A082-824	NTIS** AD-A082-824

Model Applicability

Missing features are fingerprint features in the query molecule not found in the training set.

1. Missing Feature: FCFP_12 -1853714334 [*]C[NH+](C[*])C[*]
2. Missing Feature: FCFP_12 1155241219 [*]CC[NH+]([*])[*]
3. Missing Feature: FCFP_12 -680623486 [*][NH+]([*])[C@H](C)[c](:[*]):[*]

Feature Contribution

Top features for Irritant

Fingerprint	Bit	Structure/Smiles	Score	Irritant in training set

FCFP_12	907096426	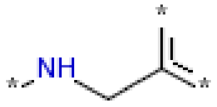	0.077	7 out of 7
FCFP_12	427906732	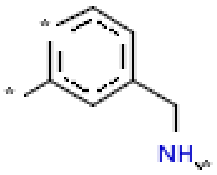	0.076	6 out of 6
FCFP_12	1390854130	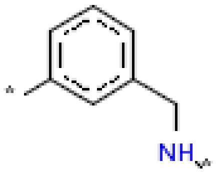	0.058	2 out of 2
**Top Features for Non-Irritant**				
**Fingerprint**	**Bit**	**Structure/Smiles**	**Score**	**Irritant in training set**
FCFP_12	-1700637232	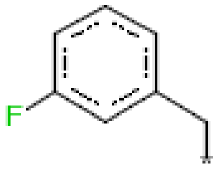	-0.846	1 out of 4
FCFP_12	566058135	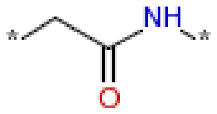	-0.367	13 out of 21

FCFP_12	460148824	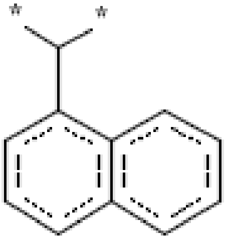	-0.222	2 out of 3

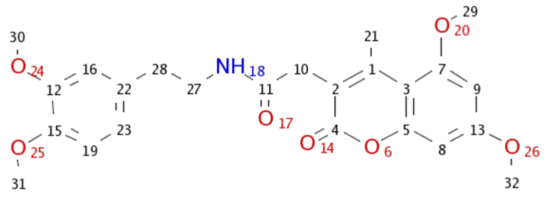

$C_{24}H_{27}NO_7$
Molecular Weight: 441.47368
ALogP: 3.388
Rotatable Bonds: 9
Acceptors: 7
Donors: 1

Model Prediction

Prediction: 120.939
Unit: mg/kg_body_weight/day

Model Applicability

Missing features are fingerprint features in the query molecule not found in the training set.

1. All properties and OPS components are within expected ranges.

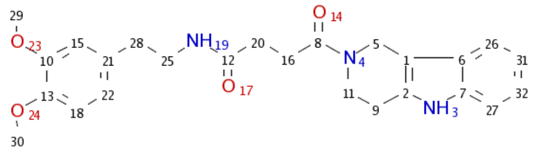

$C_{25}H_{29}N_3O_4$
Molecular Weight: 435.51546
ALogP: 2.831
Rotatable Bonds: 8
Acceptors: 4
Donors: 2

Model Prediction

Prediction: 35.731
Unit: mg/kg_body_weight/day

Model Applicability

Missing features are fingerprint features in the query molecule not found in the training set.

1. OPS PC26 out of range. Value: -3.5465. Training min, max, SD, explained variance: -3.2331, 3.4098, 1.011, 0.0124.

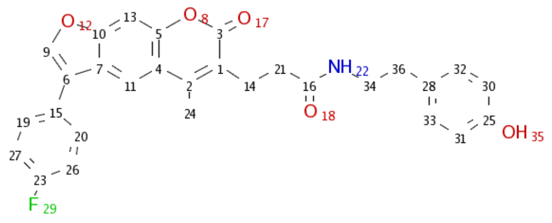

$C_{29}H_{24}FNO_5$
Molecular Weight: 485.50296
ALogP: 5.695
Rotatable Bonds: 7
Acceptors: 4
Donors: 2

Model Prediction

Prediction: 11.423
Unit: mg/kg_body_weight/day

Model Applicability

Missing features are fingerprint features in the query molecule not found in the training set.

1. All properties and OPS components are within expected ranges.

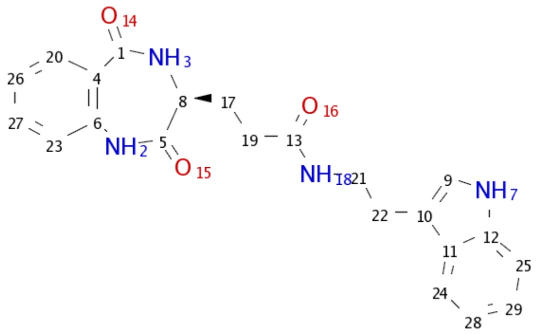

$C_{22}H_{22}N_4O_3$
Molecular Weight: 390.43508
ALogP: 1.807
Rotatable Bonds: 6
Acceptors: 3
Donors: 4

Model Prediction

Prediction: 14.541
Unit: mg/kg_body_weight/day

Model Applicability

Missing features are fingerprint features in the query molecule not found in the training set.

1. All properties and OPS components are within expected ranges.

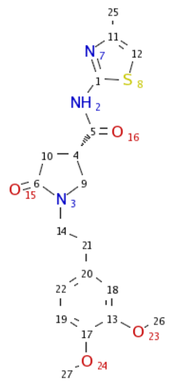

$C_{19}H_{23}N_3O_4S$
Molecular Weight: 389.46862
ALogP: 1.461
Rotatable Bonds: 7
Acceptors: 5
Donors: 1

Model Prediction

Prediction: 123.376
Unit: mg/kg_body_weight/day

Model Applicability

Missing features are fingerprint features in the query molecule not found in the training set.

1. OPS PC17 out of range. Value: 3.8215. Training min, max, SD, explained variance: -3.0124, 3.3948, 1.24, 0.0187.

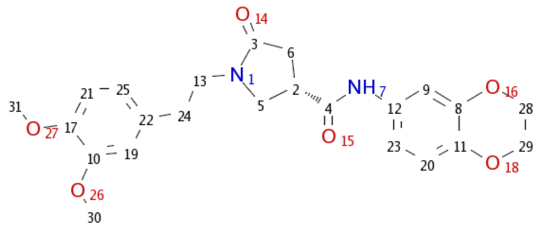

$C_{23}H_{26}N_2O_6$
Molecular Weight: 426.46234
ALogP: 1.967
Rotatable Bonds: 7
Acceptors: 6
Donors: 1

Model Prediction

Prediction: 49.533
Unit: mg/kg_body_weight/day

Model Applicability

Missing features are fingerprint features in the query molecule not found in the training set.

1. All properties and OPS components are within expected ranges.

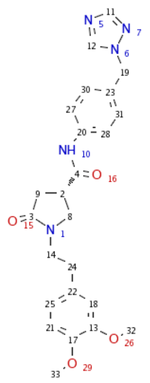

$C_{24}H_{27}N_5O_4$
Molecular Weight: 449.50228
ALogP: 1.731
Rotatable Bonds: 9
Acceptors: 6
Donors: 1

Model Prediction

Prediction: 52.595
Unit: mg/kg_body_weight/day

Model Applicability

Missing features are fingerprint features in the query molecule not found in the training set.

1. OPS PC17 out of range. Value: 3.4025. Training min, max, SD, explained variance: -3.0124, 3.3948, 1.24, 0.0187.
   Unknown FCFP_2 feature: 906560188: [*]:[c](:[*])Cn(:[*]):[*]

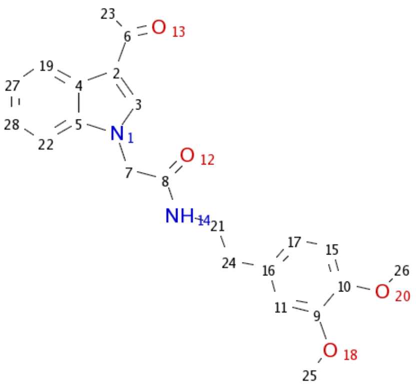

$C_{22}H_{24}N_2O_4$
Molecular Weight: 380.43696
ALogP: 3.001
Rotatable Bonds: 8
Acceptors: 4
Donors: 1

Model Prediction

Prediction: 174.770
Unit: mg/kg_body_weight/day

Model Applicability

Missing features are fingerprint features in the query molecule not found in the training set.

1. All properties and OPS components are within expected ranges.

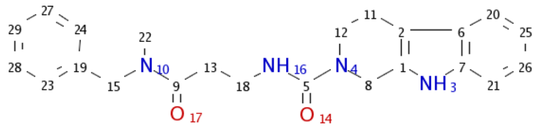

$C_{23}H_{26}N_4O_2$
Molecular Weight: 390.47814
ALogP: 2.702
Rotatable Bonds: 5
Acceptors: 2
Donors: 2

Model Prediction

Prediction: 3.760
Unit: mg/kg_body_weight/day

Model Applicability

Missing features are fingerprint features in the query molecule not found in the training set.

1. All properties and OPS components are within expected ranges.

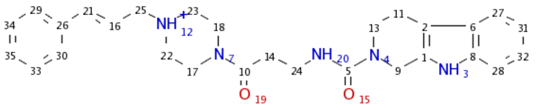

$C_{28}H_{34}N_5O_2$
Molecular Weight: 472.60186
ALogP: 1.567
Rotatable Bonds: 6
Acceptors: 2
Donors: 3

Model Prediction

Prediction: 3.296
Unit: mg/kg_body_weight/day

Model Applicability

Missing features are fingerprint features in the query molecule not found in the training set.

1. All properties and OPS components are within expected ranges.

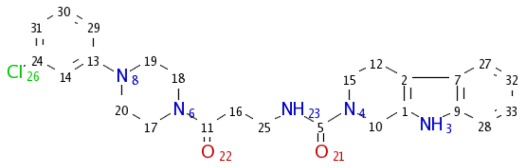

$C_{25}H_{28}ClN_5O_2$
Molecular Weight: 465.97512
ALogP: 3.413
Rotatable Bonds: 4
Acceptors: 3
Donors: 2

Model Prediction
Prediction: 6.786
Unit: mg/kg_body_weight/day

Model Applicability
Missing features are fingerprint features in the query molecule not found in the training set.

1. OPS PC23 out of range. Value: 3.0294. Training min, max, SD, explained variance: -2.8229, 2.9945, 1.071, 0.0140.
   OPS PC26 out of range. Value: -3.9141. Training min, max, SD, explained variance: -3.2331, 3.4098, 1.011, 0.0124.

MOST ACTIVE.mol

TOPKAT_Carcinogenic_Potency_TD50_Mouse

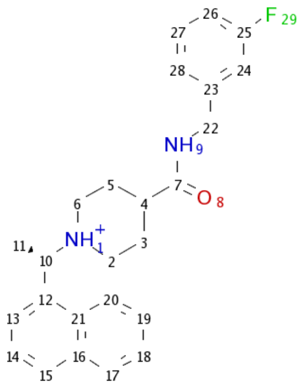

$C_{25}H_{28}FN_2O$

Molecular Weight: 391.50102

ALogP: 3.098

Rotatable Bonds: 5

Acceptors: 1

Donors: 2

Model Prediction

Prediction: 9.368

Unit: mg/kg_body_weight/day

Model Applicability

Missing features are fingerprint features in the query molecule not found in the training set.

1. All properties and OPS components are within expected ranges.
   Unknown FCFP_2 feature: -680623486: [*][NH+]([*])[C@H](C)[c](:[*]):[*]

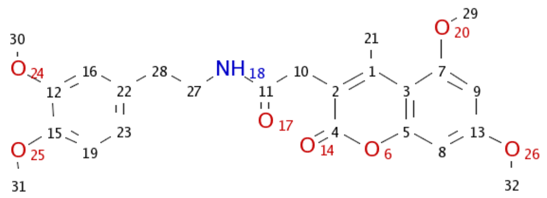

$C_{24}H_{27}NO_7$
Molecular Weight: 441.47368
ALogP: 3.388
Rotatable Bonds: 9
Acceptors: 7
Donors: 1

Model Prediction

Prediction: 5.578
Unit: mg/kg_body_weight/day

Model Applicability

Missing features are fingerprint features in the query molecule not found in the training set.

1. All properties and OPS components are within expected ranges.

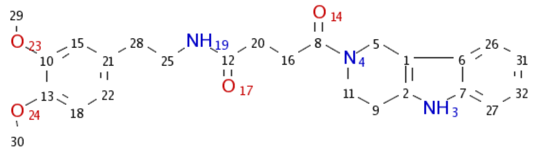

$C_{25}H_{29}N_3O_4$

Molecular Weight: 435.51546

ALogP: 2.831

Rotatable Bonds: 8

Acceptors: 4

Donors: 2

Model Prediction

Prediction: 1.199

Unit: mg/kg_body_weight/day

Model Applicability

Missing features are fingerprint features in the query molecule not found in the training set.

1. OPS PC11 out of range. Value: 6.1833. Training min, max, SD, explained variance: -6.3567, 5.7241, 1.635, 0.0252.

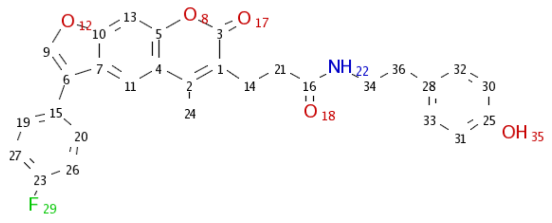

$C_{29}H_{24}FNO_5$
Molecular Weight: 485.50296
ALogP: 5.695
Rotatable Bonds: 7
Acceptors: 4
Donors: 2

Model Prediction

Prediction: 5.324
Unit: mg/kg_body_weight/day

Model Applicability

Missing features are fingerprint features in the query molecule not found in the training set.

1. All properties and OPS components are within expected ranges.

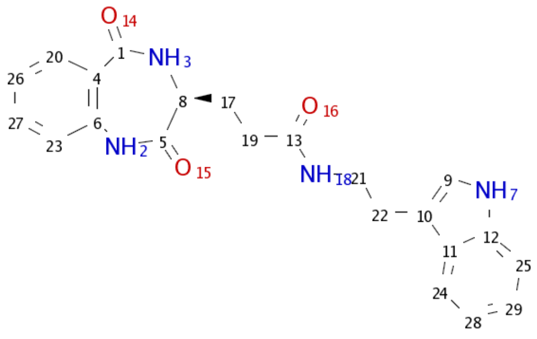

$C_{22}H_{22}N_4O_3$
Molecular Weight: 390.43508
ALogP: 1.807
Rotatable Bonds: 6
Acceptors: 3
Donors: 4

Model Prediction

Prediction: 0.827
Unit: mg/kg_body_weight/day

Model Applicability

Missing features are fingerprint features in the query molecule not found in the training set.

1. All properties and OPS components are within expected ranges.

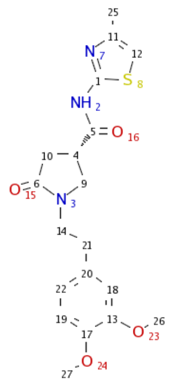

$C_{19}H_{23}N_3O_4S$
Molecular Weight: 389.46862
ALogP: 1.461
Rotatable Bonds: 7
Acceptors: 5
Donors: 1

Model Prediction

Prediction: 10.310
Unit: mg/kg_body_weight/day

Model Applicability

Missing features are fingerprint features in the query molecule not found in the training set.

1. All properties and OPS components are within expected ranges.

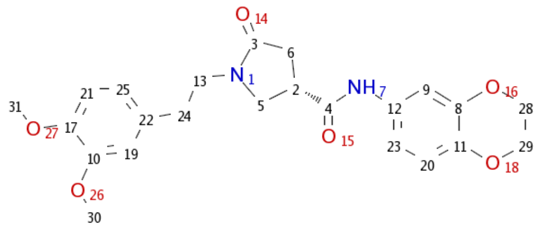

$C_{23}H_{26}N_2O_6$

Molecular Weight: 426.46234

ALogP: 1.967

Rotatable Bonds: 7

Acceptors: 6

Donors: 1

Model Prediction

Prediction: 1.163

Unit: mg/kg_body_weight/day

Model Applicability

Missing features are fingerprint features in the query molecule not found in the training set.

1. All properties and OPS components are within expected ranges.

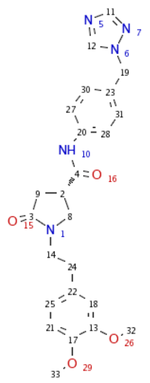

$C_{24}H_{27}N_5O_4$

Molecular Weight: 449.50228

ALogP: 1.731

Rotatable Bonds: 9

Acceptors: 6

Donors: 1

Model Prediction

Prediction: 1.861

Unit: mg/kg_body_weight/day

Model Applicability

Missing features are fingerprint features in the query molecule not found in the training set.

1. All properties and OPS components are within expected ranges.
   Unknown FCFP_2 feature: 906560188: [*]:[c](:[*])Cn(:[*]):[*]

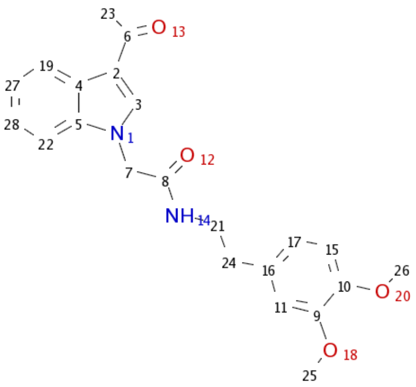

$C_{22}H_{24}N_2O_4$
Molecular Weight: 380.43696
ALogP: 3.001
Rotatable Bonds: 8
Acceptors: 4
Donors: 1

Model Prediction

Prediction: 14.544
Unit: mg/kg_body_weight/day

Model Applicability

Missing features are fingerprint features in the query molecule not found in the training set.

1. OPS PC11 out of range. Value: 5.9062. Training min, max, SD, explained variance: -6.3567, 5.7241, 1.635, 0.0252.
   OPS PC17 out of range. Value: 5.2117. Training min, max, SD, explained variance: -3.7756, 5.087, 1.368, 0.0176.

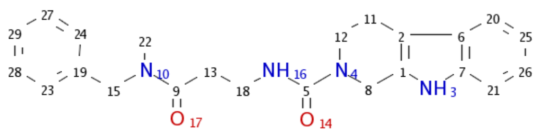

$C_{23}H_{26}N_4O_2$
Molecular Weight: 390.47814
ALogP: 2.702
Rotatable Bonds: 5
Acceptors: 2
Donors: 2

Model Prediction

Prediction: 0.325
Unit: mg/kg_body_weight/day

Model Applicability

Missing features are fingerprint features in the query molecule not found in the training set.

1. All properties and OPS components are within expected ranges.

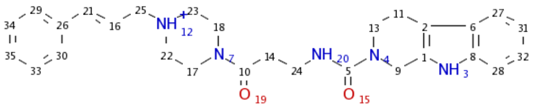

$C_{28}H_{34}N_5O_2$
Molecular Weight: 472.60186
ALogP: 1.567
Rotatable Bonds: 6
Acceptors: 2
Donors: 3

Model Prediction
Prediction: 1.726
Unit: mg/kg_body_weight/day

Model Applicability
Missing features are fingerprint features in the query molecule not found in the training set.

1. All properties and OPS components are within expected ranges.

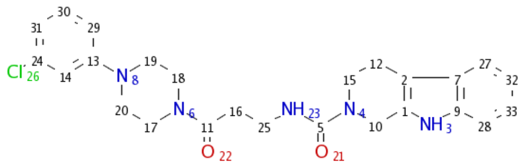

$C_{25}H_{28}ClN_5O_2$
Molecular Weight: 465.97512
ALogP: 3.413
Rotatable Bonds: 4
Acceptors: 3
Donors: 2

Model Prediction

Prediction: 0.315
Unit: mg/kg_body_weight/day

Model Applicability

Missing features are fingerprint features in the query molecule not found in the training set.

1. OPS PC11 out of range. Value: 6.7001. Training min, max, SD, explained variance: -6.3567, 5.7241, 1.635, 0.0252.
   OPS PC25 out of range. Value: 4.3169. Training min, max, SD, explained variance: -3.9292, 4.0882, 1.064, 0.0107.

MOST ACTIVE.mol

TOPKAT_Carcinogenic_Potency_TD50_Rat

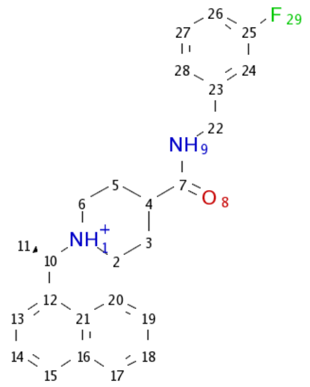

$C_{25}H_{28}FN_2O$
Molecular Weight: 391.50102
ALogP: 3.098
Rotatable Bonds: 5
Acceptors: 1
Donors: 2

Model Prediction

Prediction: 2.548
Unit: mg/kg_body_weight/day

Model Applicability

Missing features are fingerprint features in the query molecule not found in the training set.

1. All properties and OPS components are within expected ranges.
   Unknown FCFP_2 feature: -680623486: [*][NH+]([*])[C@H](C)[c](:[*]):[*]

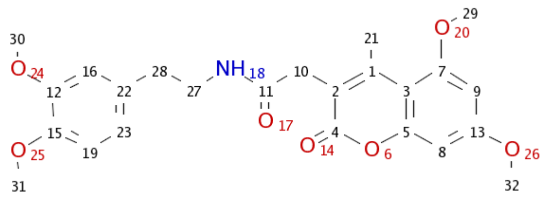

$C_{24}H_{27}NO_7$
Molecular Weight: 441.47368
ALogP: 3.388
Rotatable Bonds: 9
Acceptors: 7
Donors: 1

Model Prediction

Prediction: 0.040
Unit: g/kg_body_weight

Model Applicability

Missing features are fingerprint features in the query molecule not found in the training set.

1. All properties and OPS components are within expected ranges.

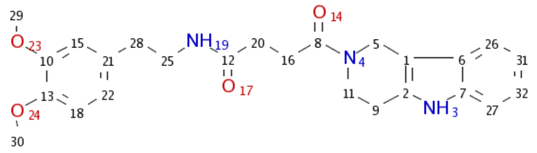

$C_{25}H_{29}N_3O_4$

Molecular Weight: 435.51546

ALogP: 2.831

Rotatable Bonds: 8

Acceptors: 4

Donors: 2

Model Prediction

Prediction: 0.540

Unit: g/kg_body_weight

Model Applicability

Missing features are fingerprint features in the query molecule not found in the training set.

1. All properties and OPS components are within expected ranges.

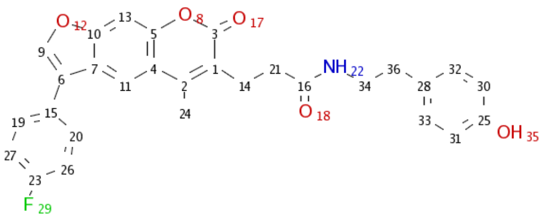

$C_{29}H_{24}FNO_5$
Molecular Weight: 485.50296
ALogP: 5.695
Rotatable Bonds: 7
Acceptors: 4
Donors: 2

Model Prediction

Prediction: 0.044
Unit: g/kg_body_weight

Model Applicability

Missing features are fingerprint features in the query molecule not found in the training set.

1. All properties and OPS components are within expected ranges.

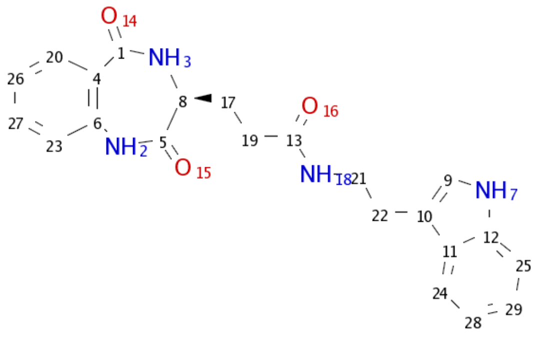

$C_{22}H_{22}N_4O_3$
Molecular Weight: 390.43508
ALogP: 1.807
Rotatable Bonds: 6
Acceptors: 3
Donors: 4

Model Prediction

Prediction: 0.233
Unit: g/kg_body_weight

Model Applicability

Missing features are fingerprint features in the query molecule not found in the training set.

1. All properties and OPS components are within expected ranges.

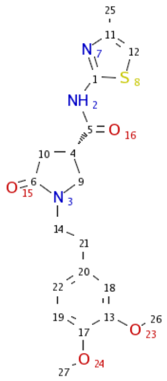

$C_{19}H_{23}N_3O_4S$

Molecular Weight: 389.46862

ALogP: 1.461

Rotatable Bonds: 7

Acceptors: 5

Donors: 1

Model Prediction

Prediction: 0.340

Unit: g/kg_body_weight

Model Applicability

Missing features are fingerprint features in the query molecule not found in the training set.

1. OPS PC33 out of range. Value: -4.7489. Training min, max, SD, explained variance: -4.34, 3.6146, 1.305, 0.0065.

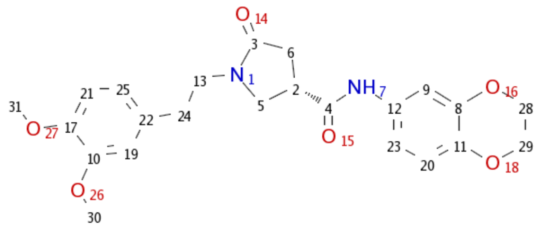

$C_{23}H_{26}N_2O_6$
Molecular Weight: 426.46234
ALogP: 1.967
Rotatable Bonds: 7
Acceptors: 6
Donors: 1

Model Prediction

Prediction: 0.446
Unit: g/kg_body_weight

Model Applicability

Missing features are fingerprint features in the query molecule not found in the training set.

1. OPS PC33 out of range. Value: -4.7216. Training min, max, SD, explained variance: -4.34, 3.6146, 1.305, 0.0065.

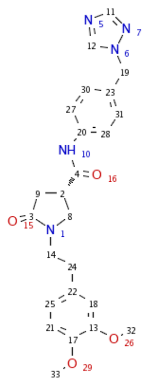

$C_{24}H_{27}N_5O_4$
Molecular Weight: 449.50228
ALogP: 1.731
Rotatable Bonds: 9
Acceptors: 6
Donors: 1

Model Prediction

Prediction: 0.151
Unit: g/kg_body_weight

Model Applicability

Missing features are fingerprint features in the query molecule not found in the training set.

1. OPS PC33 out of range. Value: -5.3637. Training min, max, SD, explained variance: -4.34, 3.6146, 1.305, 0.0065.
   OPS PC36 out of range. Value: 4.1555. Training min, max, SD, explained variance: -4.4792, 3.9684, 1.222, 0.0057.
   Unknown FCFP_2 feature: 906560188: [*]:[c](:[*])Cn(:[*]):[*]

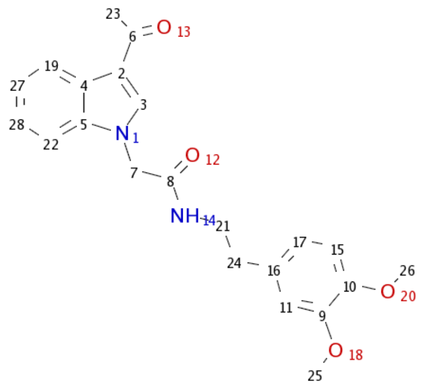

$C_{22}H_{24}N_2O_4$
Molecular Weight: 380.43696
ALogP: 3.001
Rotatable Bonds: 8
Acceptors: 4
Donors: 1

Model Prediction

Prediction: 0.139
Unit: g/kg_body_weight

Model Applicability

Missing features are fingerprint features in the query molecule not found in the training set.

1. All properties and OPS components are within expected ranges.

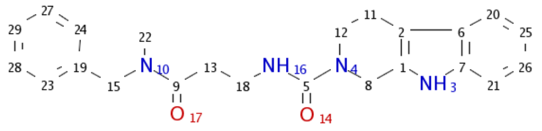

$C_{23}H_{26}N_4O_2$
Molecular Weight: 390.47814
ALogP: 2.702
Rotatable Bonds: 5
Acceptors: 2
Donors: 2

Model Prediction

Prediction: 0.015
Unit: g/kg_body_weight

Model Applicability

Missing features are fingerprint features in the query molecule not found in the training set.

1. OPS PC5 out of range. Value: -7.9239. Training min, max, SD, explained variance: -7.4165, 11.409, 3.374, 0.0435.

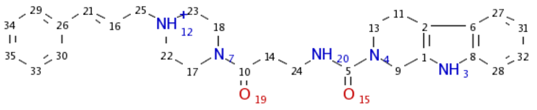

$C_{28}H_{34}N_5O_2$
Molecular Weight: 472.60186
ALogP: 1.567
Rotatable Bonds: 6
Acceptors: 2
Donors: 3

Model Prediction

Prediction: 0.002
Unit: g/kg_body_weight

Model Applicability

Missing features are fingerprint features in the query molecule not found in the training set.

1. All properties and OPS components are within expected ranges.
   Unknown FCFP_2 feature: 10: [*][NH+]([*])[*]
   Unknown FCFP_2 feature: -1853714334: [*]C[NH+](C[*])C[*]
   Unknown FCFP_2 feature: 1155241219: [*]CC[NH+]([*])[*]

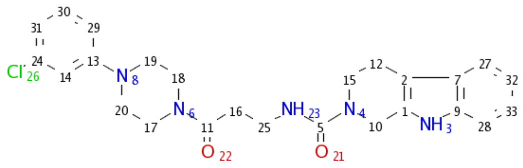

$C_{25}H_{28}ClN_5O_2$
Molecular Weight: 465.97512
ALogP: 3.413
Rotatable Bonds: 4
Acceptors: 3
Donors: 2

Model Prediction

Prediction: 0.018
Unit: g/kg_body_weight

Model Applicability

Missing features are fingerprint features in the query molecule not found in the training set.

1. All properties and OPS components are within expected ranges.

MOST ACTIVE.mol

TOPKAT_Chronic_LOAEL

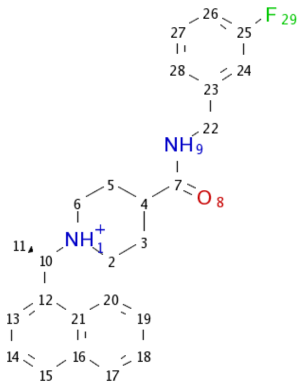

$C_{25}H_{28}FN_2O$

Molecular Weight: 391.50102

ALogP: 3.098

Rotatable Bonds: 5

Acceptors: 1

Donors: 2

Model Prediction

Prediction: 0.036

Unit: g/kg_body_weight

Model Applicability

Missing features are fingerprint features in the query molecule not found in the training set.

1. All properties and OPS components are within expected ranges.
   Unknown FCFP_2 feature: 10: [*][NH+]([*])[*]
   Unknown FCFP_2 feature: -1853714334: [*]C[NH+](C[*])C[*]
   Unknown FCFP_2 feature: 1155241219: [*]CC[NH+]([*])[*]
   Unknown FCFP_2 feature: -680623486: [*][NH+]([*])[C@H](C)[c](:[*]):[*]

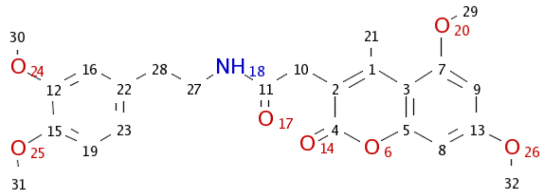

$C_{24}H_{27}NO_7$
Molecular Weight: 441.47368
ALogP: 3.388
Rotatable Bonds: 9
Acceptors: 7
Donors: 1

Model Prediction

Prediction: 0.142
Unit: g/kg_body_weight

Model Applicability

Missing features are fingerprint features in the query molecule not found in the training set.

1. OPS PC9 out of range. Value: 4.0669. Training min, max, SD, explained variance: -2.8592, 3.3844, 1.263, 0.0360.

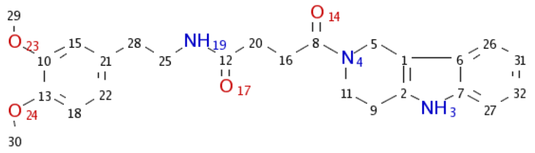

$C_{25}H_{29}N_3O_4$
Molecular Weight: 435.51546
ALogP: 2.831
Rotatable Bonds: 8
Acceptors: 4
Donors: 2

Model Prediction

Prediction: 0.144
Unit: g/kg_body_weight

Model Applicability

Missing features are fingerprint features in the query molecule not found in the training set.

1. OPS PC9 out of range. Value: 3.9159. Training min, max, SD, explained variance: -2.8592, 3.3844, 1.263, 0.0360.

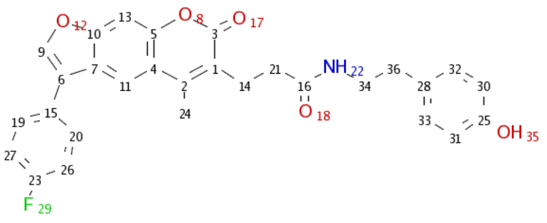

$C_{29}H_{24}FNO_5$
Molecular Weight: 485.50296
ALogP: 5.695
Rotatable Bonds: 7
Acceptors: 4
Donors: 2

Model Prediction

Prediction: 0.369
Unit: g/kg_body_weight

Model Applicability

Missing features are fingerprint features in the query molecule not found in the training set.

1. OPS PC9 out of range. Value: 4.3137. Training min, max, SD, explained variance: -2.8592, 3.3844, 1.263, 0.0360.
   OPS PC12 out of range. Value: -2.5269. Training min, max, SD, explained variance: -2.3837, 2.9195, 1.08, 0.0263.

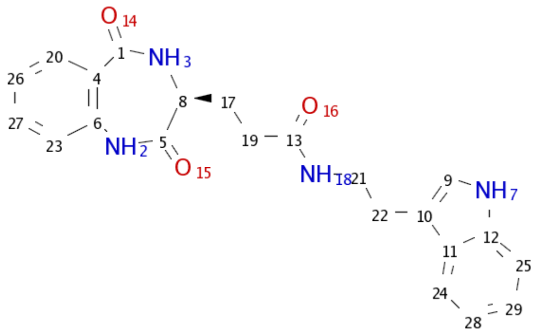

$C_{22}H_{22}N_4O_3$
Molecular Weight: 390.43508
ALogP: 1.807
Rotatable Bonds: 6
Acceptors: 3
Donors: 4

Model Prediction
Prediction: 0.328
Unit: g/kg_body_weight

Model Applicability
Missing features are fingerprint features in the query molecule not found in the training set.

1. All properties and OPS components are within expected ranges.

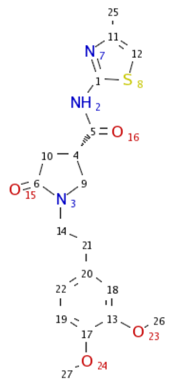

$C_{19}H_{23}N_3O_4S$

Molecular Weight: 389.46862

ALogP: 1.461

Rotatable Bonds: 7

Acceptors: 5

Donors: 1

Model Prediction

Prediction: 0.034

Unit: g/kg_body_weight

Model Applicability

Missing features are fingerprint features in the query molecule not found in the training set.

1. OPS PC9 out of range. Value: 4.7781. Training min, max, SD, explained variance: -2.8592, 3.3844, 1.263, 0.0360.
   OPS PC14 out of range. Value: 4.5353. Training min, max, SD, explained variance: -2.0624, 3.3586, 1.011, 0.0231.

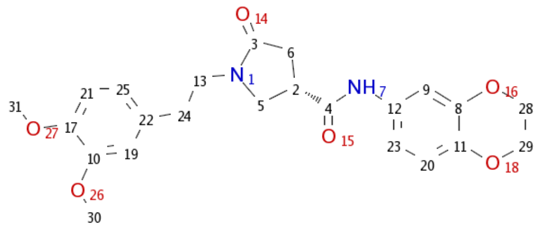

$C_{23}H_{26}N_2O_6$
Molecular Weight: 426.46234
ALogP: 1.967
Rotatable Bonds: 7
Acceptors: 6
Donors: 1

Model Prediction

Prediction: 0.123
Unit: g/kg_body_weight

Model Applicability

Missing features are fingerprint features in the query molecule not found in the training set.

1. OPS PC9 out of range. Value: 5.0363. Training min, max, SD, explained variance: -2.8592, 3.3844, 1.263, 0.0360.

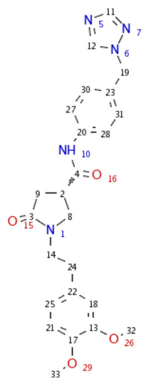

$C_{24}H_{27}N_5O_4$
Molecular Weight: 449.50228
ALogP: 1.731
Rotatable Bonds: 9
Acceptors: 6
Donors: 1

Model Prediction

Prediction: 0.101
Unit: g/kg_body_weight

Model Applicability

Missing features are fingerprint features in the query molecule not found in the training set.

1. OPS PC9 out of range. Value: 6.0539. Training min, max, SD, explained variance: -2.8592, 3.3844, 1.263, 0.0360.
   Unknown FCFP_2 feature: -2049666792: [*]Cn1:c:[*]:[*]:n:1
   Unknown FCFP_2 feature: -124685461: [*]1:[*]:n:c:n:1
   Unknown FCFP_2 feature: 906560188: [*]:[c](:[*])Cn(:[*]):[*]

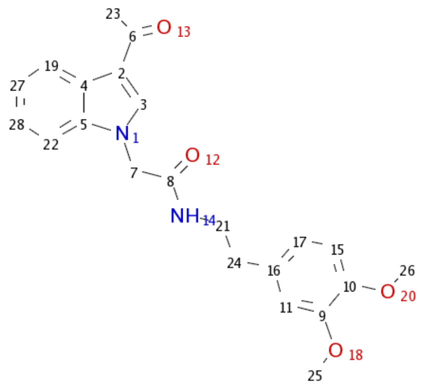

$C_{22}H_{24}N_2O_4$

Molecular Weight: 380.43696

ALogP: 3.001

Rotatable Bonds: 8

Acceptors: 4

Donors: 1

Model Prediction

Prediction: 0.046

Unit: g/kg_body_weight

Model Applicability

Missing features are fingerprint features in the query molecule not found in the training set.

1. OPS PC9 out of range. Value: 3.853. Training min, max, SD, explained variance: -2.8592, 3.3844, 1.263, 0.0360.
   Unknown FCFP_2 feature: -306856457: [*]Cn1:c:[*]:[*]:[c]:1:[*]
   Unknown FCFP_2 feature: -1645149908: [*]C(=[*])Cn(:[*]):[*]

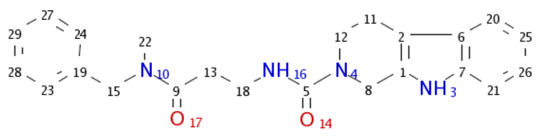

$C_{23}H_{26}N_4O_2$
Molecular Weight: 390.47814
ALogP: 2.702
Rotatable Bonds: 5
Acceptors: 2
Donors: 2

Model Prediction

Prediction: 0.069
Unit: g/kg_body_weight

Model Applicability

Missing features are fingerprint features in the query molecule not found in the training set.

1. All properties and OPS components are within expected ranges.

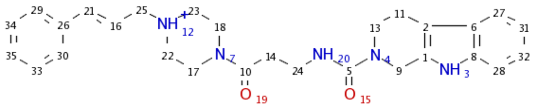

$C_{28}H_{34}N_5O_2$

Molecular Weight: 472.60186

ALogP: 1.567

Rotatable Bonds: 6

Acceptors: 2

Donors: 3

Model Prediction

Prediction: 0.071

Unit: g/kg_body_weight

Model Applicability

Missing features are fingerprint features in the query molecule not found in the training set.

1. All properties and OPS components are within expected ranges.
   Unknown FCFP_2 feature: 10: [*][NH+]([*])[*]
   Unknown FCFP_2 feature: -1853714334: [*]C[NH+](C[*])C[*]
   Unknown FCFP_2 feature: 1155241219: [*]CC[NH+]([*])[*]

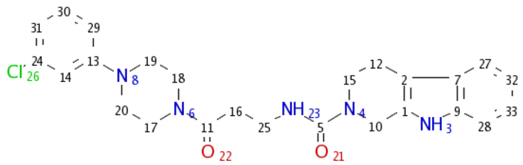

$C_{25}H_{28}ClN_5O_2$
Molecular Weight: 465.97512
ALogP: 3.413
Rotatable Bonds: 4
Acceptors: 3
Donors: 2

Model Prediction

Prediction: 0.117
Unit: g/kg_body_weight

Model Applicability

Missing features are fingerprint features in the query molecule not found in the training set.

1. OPS PC11 out of range. Value: 4.3554. Training min, max, SD, explained variance: -3.8339, 3.875, 1.233, 0.0343.

MOST ACTIVE.mol

TOPKAT_Rat_Maximum_Tolerated_Dose_Feed

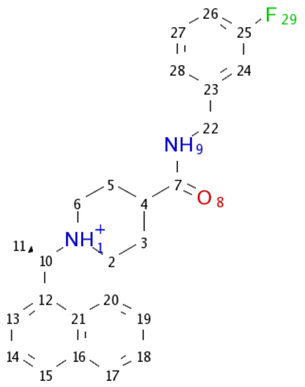

$C_{25}H_{28}FN_2O$

Molecular Weight: 391.50102

ALogP: 3.098

Rotatable Bonds: 5

Acceptors: 1

Donors: 2

Model Prediction

Prediction: 0.124

Unit: g/kg_body_weight

Model Applicability

Missing features are fingerprint features in the query molecule not found in the training set.

1. All properties and OPS components are within expected ranges.
   Unknown FCFP_2 feature: 10: [*][NH+]([*])[*]
   Unknown FCFP_2 feature: -1853714334: [*]C[NH+](C[*])C[*]
   Unknown FCFP_2 feature: 1155241219: [*]CC[NH+]([*])[*]
   Unknown FCFP_2 feature: -680623486: [*][NH+]([*])[C@H](C)[c](:[*]):[*]

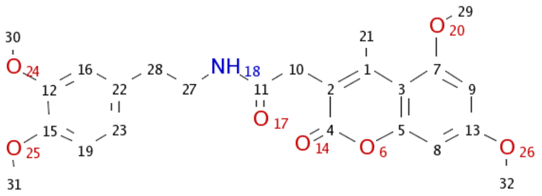

$C_{24}H_{27}NO_7$
Molecular Weight: 441.47368
ALogP: 3.388
Rotatable Bonds: 9
Acceptors: 7
Donors: 1

Model Prediction

Prediction: 0.004
Unit: g/kg_body_weight

Model Applicability

Missing features are fingerprint features in the query molecule not found in the training set.

1. Molecular_Weight out of range. Value: 441.47. Training min, max, mean, SD: 68.074, 434.63, 171.13, 85.06.
   Num_H_Acceptors out of range. Value: 7. Training min, max, mean, SD: 0, 6, 1.6146, 1.644.
   OPS PC6 out of range. Value: -3.1803. Training min, max, SD, explained variance: -2.4321, 2.9885, 1.256, 0.0488.
   Unknown FCFP_2 feature: -1678275541: [*]C(=C(C)[c](:[*]):[*])[*]

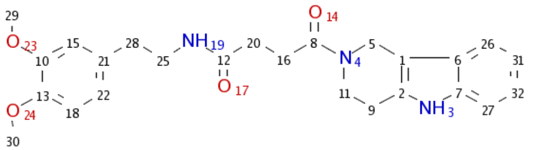

$C_{25}H_{29}N_3O_4$

Molecular Weight: 435.51546

ALogP: 2.831

Rotatable Bonds: 8

Acceptors: 4

Donors: 2

Model Prediction

Prediction: 0.019

Unit: g/kg_body_weight

Model Applicability

Missing features are fingerprint features in the query molecule not found in the training set.

1. Molecular_Weight out of range. Value: 435.52. Training min, max, mean, SD: 68.074, 434.63, 171.13, 85.06.
   Num_AromaticRings out of range. Value: 3. Training min, max, mean, SD: 0, 2, 0.5625, 0.693.
   OPS PC6 out of range. Value: -3.0009. Training min, max, SD, explained variance: -2.4321, 2.9885, 1.256, 0.0488.
   Unknown FCFP_2 feature: 19: [*]:n:[*]
   Unknown FCFP_2 feature: 203707511: [*]C[c]1:n:[*]:[*]:[c]:1[*]
   Unknown FCFP_2 feature: 2005402822: [*][c]1:[*]:[*]:[c](:[*]):n:1
   Unknown FCFP_2 feature: 907036844: [*]N([*])C[c](:[*]):[*]
   Unknown FCFP_2 feature: 307448885: [*]:c:[c]1:n:[*]:[*]:[c]:1:[*]

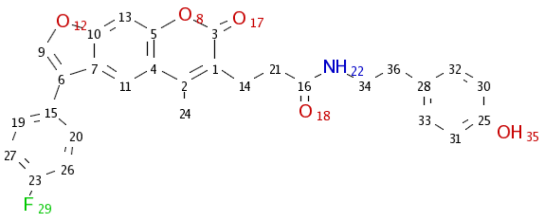

$C_{29}H_{24}FNO_5$
Molecular Weight: 485.50296
ALogP: 5.695
Rotatable Bonds: 7
Acceptors: 4
Donors: 2

Model Prediction

Prediction: 0.000
Unit: g/kg_body_weight

Model Applicability

Missing features are fingerprint features in the query molecule not found in the training set.

1. Molecular_Weight out of range. Value: 485.5. Training min, max, mean, SD: 68.074, 434.63, 171.13, 85.06.
   Num_AromaticRings out of range. Value: 4. Training min, max, mean, SD: 0, 2, 0.5625, 0.693.
   OPS PC1 out of range. Value: 8.058. Training min, max, SD, explained variance: -4.0008, 7.9165, 2.861, 0.2531.
   OPS PC10 out of range. Value: 2.8506. Training min, max, SD, explained variance: -3.9696, 2.3971, 0.982, 0.0298.
   Unknown FCFP_2 feature: -1678275541: [*]C(=C(C)[c](:[*]):[*])[*]
   Unknown FCFP_2 feature: -1861645784: [*]:[c](:[*])[c]1:c:[*]:[*]:[c]:1:[*]

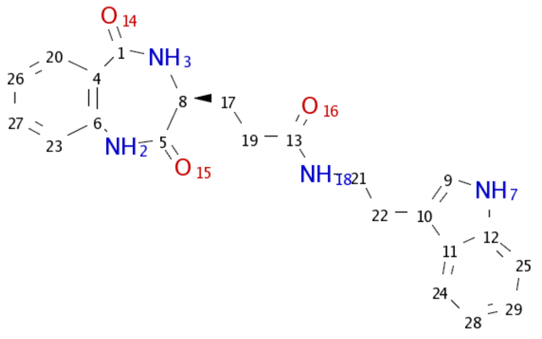

$C_{22}H_{22}N_4O_3$

Molecular Weight: 390.43508

ALogP: 1.807

Rotatable Bonds: 6

Acceptors: 3

Donors: 4

Model Prediction

Prediction: 0.908

Unit: g/kg_body_weight

Model Applicability

Missing features are fingerprint features in the query molecule not found in the training set.

1. Num_H_Donors out of range. Value: 4. Training min, max, mean, SD: 0, 3, 0.4375, 0.8311.
   Num_AromaticRings out of range. Value: 3. Training min, max, mean, SD: 0, 2, 0.5625, 0.693.
   Unknown FCFP_2 feature: 19: [*]:n:[*]
   Unknown FCFP_2 feature: 2005402822: [*][c]1:[*]:[*]:[c](:[*]):n:1
   Unknown FCFP_2 feature: 1618184456: [*][c]1:[*]:[*]:n:c:1
   Unknown FCFP_2 feature: 307448885: [*]:c:[c]1:n:[*]:[*]:[c]:1:[*]

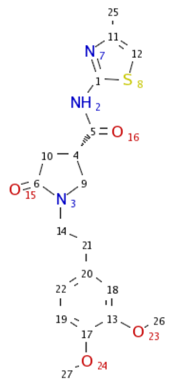

$C_{19}H_{23}N_3O_4S$
Molecular Weight: 389.46862
ALogP: 1.461
Rotatable Bonds: 7
Acceptors: 5
Donors: 1

Model Prediction

Prediction: 0.063
Unit: g/kg_body_weight

Model Applicability

Missing features are fingerprint features in the query molecule not found in the training set.

1. OPS PC6 out of range. Value: -2.5908. Training min, max, SD, explained variance: -2.4321, 2.9885, 1.256, 0.0488.
   Unknown FCFP_2 feature: -1151914249: [*]N[c]1:n:[*]:[*]:s:1

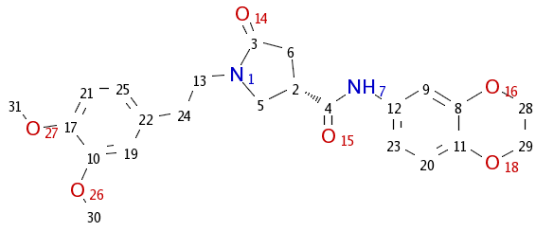

$C_{23}H_{26}N_2O_6$

Molecular Weight: 426.46234

ALogP: 1.967

Rotatable Bonds: 7

Acceptors: 6

Donors: 1

Model Prediction

Prediction: 0.006

Unit: g/kg_body_weight

Model Applicability

Missing features are fingerprint features in the query molecule not found in the training set.

1. OPS PC6 out of range. Value: -2.7305. Training min, max, SD, explained variance: -2.4321, 2.9885, 1.256, 0.0488.

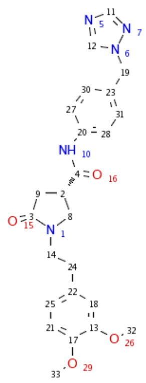

$C_{24}H_{27}N_5O_4$
Molecular Weight: 449.50228
ALogP: 1.731
Rotatable Bonds: 9
Acceptors: 6
Donors: 1

Model Prediction

Prediction: 0.002
Unit: g/kg_body_weight

Model Applicability

Missing features are fingerprint features in the query molecule not found in the training set.

1. Molecular_Weight out of range. Value: 449.5. Training min, max, mean, SD: 68.074, 434.63, 171.13, 85.06.
   Num_AromaticRings out of range. Value: 3. Training min, max, mean, SD: 0, 2, 0.5625, 0.693.
   OPS PC6 out of range. Value: -3.0296. Training min, max, SD, explained variance: -2.4321, 2.9885, 1.256, 0.0488.
   Unknown FCFP_2 feature: -2049666792: [*]Cn1:c:[*]:[*]:n:1
   Unknown FCFP_2 feature: -124685461: [*]1:[*]:n:c:n:1
   Unknown FCFP_2 feature: 906560188: [*]:[c](:[*])Cn(:[*]):[*]

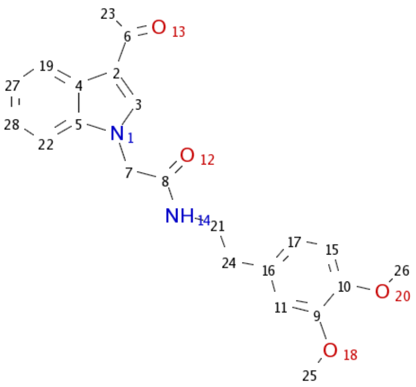

$C_{22}H_{24}N_2O_4$

Molecular Weight: 380.43696

ALogP: 3.001

Rotatable Bonds: 8

Acceptors: 4

Donors: 1

Model Prediction

Prediction: 0.001

Unit: g/kg_body_weight

Model Applicability

Missing features are fingerprint features in the query molecule not found in the training set.

1. Num_AromaticRings out of range. Value: 3. Training min, max, mean, SD: 0, 2, 0.5625, 0.693.
   OPS PC6 out of range. Value: -3.3568. Training min, max, SD, explained variance: -2.4321, 2.9885, 1.256, 0.0488.
   Unknown FCFP_2 feature: -306856457: [*]Cn1:c:[*]:[*]:[c]:1:[*]
   Unknown FCFP_2 feature: -1549192822: [*]:[c](:[*])C(=O)C
   Unknown FCFP_2 feature: -1645149908: [*]C(=[*])Cn(:[*]):[*]

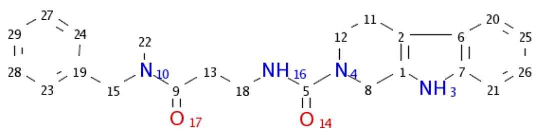

$C_{23}H_{26}N_4O_2$

Molecular Weight: 390.47814

ALogP: 2.702

Rotatable Bonds: 5

Acceptors: 2

Donors: 2

Model Prediction

Prediction: 8.074

Unit: g/kg_body_weight

Model Applicability

Missing features are fingerprint features in the query molecule not found in the training set.

1. Num_AromaticRings out of range. Value: 3. Training min, max, mean, SD: 0, 2, 0.5625, 0.693.
   Unknown FCFP_2 feature: 19: [*]:n:[*]
   Unknown FCFP_2 feature: 203707511: [*]C[c]1:n:[*]:[*]:[c]:1[*]
   Unknown FCFP_2 feature: 2005402822: [*][c]1:[*]:[*]:[c](:[*]):n:1
   Unknown FCFP_2 feature: 307448885: [*]:c:[c]1:n:[*]:[*]:[c]:1:[*]
   Unknown FCFP_2 feature: 907036844: [*]N([*])C[c](:[*]):[*]

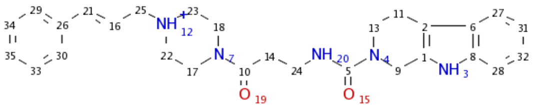

$C_{28}H_{34}N_5O_2$
Molecular Weight: 472.60186
ALogP: 1.567
Rotatable Bonds: 6
Acceptors: 2
Donors: 3

Model Prediction

Prediction: 26.912
Unit: g/kg_body_weight

Model Applicability

Missing features are fingerprint features in the query molecule not found in the training set.

1. Molecular_Weight out of range. Value: 472.6. Training min, max, mean, SD: 68.074, 434.63, 171.13, 85.06.
   Num_AromaticRings out of range. Value: 3. Training min, max, mean, SD: 0, 2, 0.5625, 0.693.
   Unknown FCFP_2 feature: 19: [*]:n:[*]
   Unknown FCFP_2 feature: 10: [*][NH+]([*])[*]
   Unknown FCFP_2 feature: 203707511: [*]C[c]1:n:[*]:[*]:[c]:1[*]
   Unknown FCFP_2 feature: 2005402822: [*][c]1:[*]:[*]:[c](:[*]):n:1
   Unknown FCFP_2 feature: 307448885: [*]:c:[c]1:n:[*]:[*]:[c]:1:[*]
   Unknown FCFP_2 feature: 907036844: [*]N([*])C[c](:[*]):[*]
   Unknown FCFP_2 feature: -1853714334: [*]C[NH+](C[*])C[*]
   Unknown FCFP_2 feature: 1155241219: [*]CC[NH+]([*])[*]

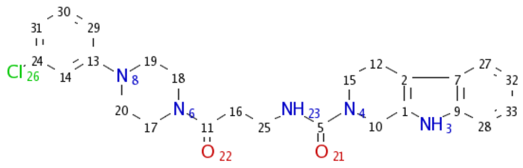

$C_{25}H_{28}ClN_5O_2$
Molecular Weight: 465.97512
ALogP: 3.413
Rotatable Bonds: 4
Acceptors: 3
Donors: 2

Model Prediction

Prediction: 0.002
Unit: g/kg_body_weight

Model Applicability

Missing features are fingerprint features in the query molecule not found in the training set.

1. Molecular_Weight out of range. Value: 465.98. Training min, max, mean, SD: 68.074, 434.63, 171.13, 85.06.
   Num_AromaticRings out of range. Value: 3. Training min, max, mean, SD: 0, 2, 0.5625, 0.693.
   OPS PC10 out of range. Value: 2.7252. Training min, max, SD, explained variance: -3.9696, 2.3971, 0.982, 0.0298.
   Unknown FCFP_2 feature: 19: [*]:n:[*]
   Unknown FCFP_2 feature: 203707511: [*]C[c]1:n:[*]:[*]:[c]:1[*]
   Unknown FCFP_2 feature: 2005402822: [*][c]1:[*]:[*]:[c](:[*]):n:1
   Unknown FCFP_2 feature: 307448885: [*]:c:[c]1:n:[*]:[*]:[c]:1:[*]
   Unknown FCFP_2 feature: 907036844: [*]N([*])C[c](:[*]):[*]

MOST ACTIVE.mol **TOPKAT_Rat_Maximum_Tolerated_Dose_Gavage**

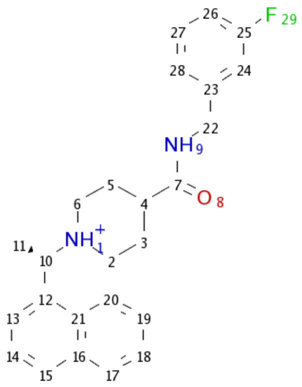

$C_{25}H_{28}FN_2O$

Molecular Weight: 391.50102

ALogP: 3.098

Rotatable Bonds: 5

Acceptors: 1

Donors: 2

Model Prediction

Prediction: 0.016

Unit: g/kg_body_weight

Model Applicability

Missing features are fingerprint features in the query molecule not found in the training set.

1. Num_AromaticRings out of range. Value: 3. Training min, max, mean, SD: 0, 2, 0.5625, 0.693.
   OPS PC7 out of range. Value: -3.4465. Training min, max, SD, explained variance: -2.8003, 2.9332, 1.16, 0.0416.
   Unknown FCFP_2 feature: 10: [*][NH+]([*])[*]
   Unknown FCFP_2 feature: -1853714334: [*]C[NH+](C[*])C[*]
   Unknown FCFP_2 feature: 1155241219: [*]CC[NH+]([*])[*]
   Unknown FCFP_2 feature: -680623486: [*][NH+]([*])[C@H](C)[c](:[*]):[*]

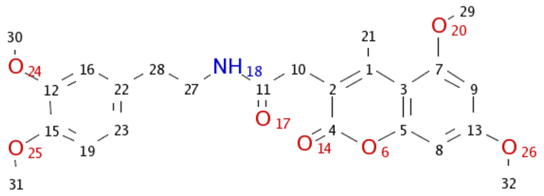

$C_{24}H_{27}NO_7$
Molecular Weight: 441.47368
ALogP: 3.388
Rotatable Bonds: 9
Acceptors: 7
Donors: 1

Model Prediction

Prediction: 4.097
Unit: g/kg_body_weight

Model Applicability

Missing features are fingerprint features in the query molecule not found in the training set.

1. OPS PC34 out of range. Value: 9.6064. Training min, max, SD, explained variance: -6.4714, 7.7587, 1.869, 0.0070.

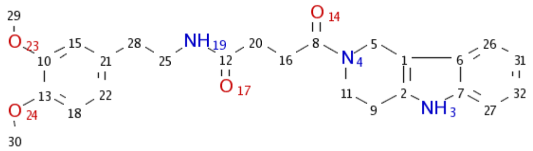

$C_{25}H_{29}N_3O_4$
Molecular Weight: 435.51546
ALogP: 2.831
Rotatable Bonds: 8
Acceptors: 4
Donors: 2

Model Prediction

Prediction: 27.191
Unit: g/kg_body_weight

Model Applicability

Missing features are fingerprint features in the query molecule not found in the training set.

1. OPS PC26 out of range. Value: 12.838. Training min, max, SD, explained variance: -7.9646, 10.26, 2.09, 0.0088.

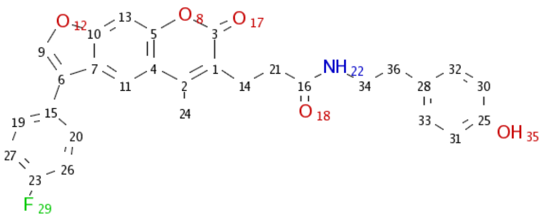

$C_{29}H_{24}FNO_5$
Molecular Weight: 485.50296
ALogP: 5.695
Rotatable Bonds: 7
Acceptors: 4
Donors: 2

Model Prediction

Prediction: 0.252
Unit: g/kg_body_weight

Model Applicability

Missing features are fingerprint features in the query molecule not found in the training set.

1. OPS PC39 out of range. Value: -8.5916. Training min, max, SD, explained variance: -6.2305, 6.7137, 1.769, 0.0063.

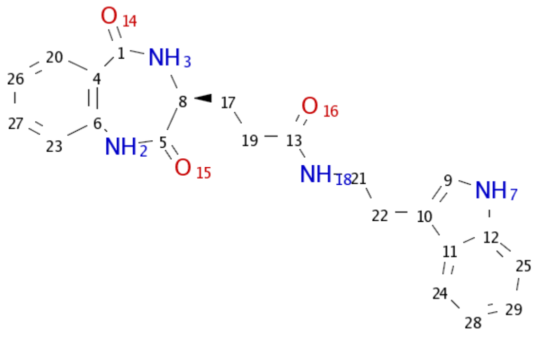

$C_{22}H_{22}N_4O_3$
Molecular Weight: 390.43508
ALogP: 1.807
Rotatable Bonds: 6
Acceptors: 3
Donors: 4

Model Prediction

Prediction: 32.394
Unit: g/kg_body_weight

Model Applicability

Missing features are fingerprint features in the query molecule not found in the training set.

1. All properties and OPS components are within expected ranges.

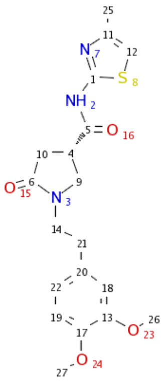

$C_{19}H_{23}N_3O_4S$

Molecular Weight: 389.46862

ALogP: 1.461

Rotatable Bonds: 7

Acceptors: 5

Donors: 1

Model Prediction

Prediction: 14.820

Unit: g/kg_body_weight

Model Applicability

Missing features are fingerprint features in the query molecule not found in the training set.

1. OPS PC19 out of range. Value: -5.9332. Training min, max, SD, explained variance: -5.2853, 15.143, 2.34, 0.0110.

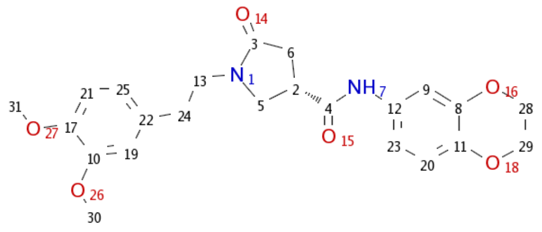

$C_{23}H_{26}N_2O_6$
Molecular Weight: 426.46234
ALogP: 1.967
Rotatable Bonds: 7
Acceptors: 6
Donors: 1

Model Prediction

Prediction: 26.757
Unit: g/kg_body_weight

Model Applicability

Missing features are fingerprint features in the query molecule not found in the training set.

1. OPS PC33 out of range. Value: 7.8436. Training min, max, SD, explained variance: -7.1323, 7.6992, 1.899, 0.0072.
   OPS PC35 out of range. Value: -7.4244. Training min, max, SD, explained variance: -7.3188, 9.6186, 1.858, 0.0069.

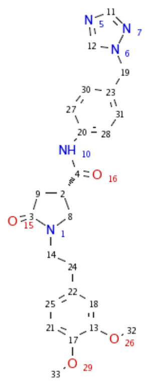

$C_{24}H_{27}N_5O_4$

Molecular Weight: 449.50228

ALogP: 1.731

Rotatable Bonds: 9

Acceptors: 6

Donors: 1

Model Prediction

Prediction: 26.742

Unit: g/kg_body_weight

Model Applicability

Missing features are fingerprint features in the query molecule not found in the training set.

1. OPS PC35 out of range. Value: -7.6474. Training min, max, SD, explained variance: -7.3188, 9.6186, 1.858, 0.0069.

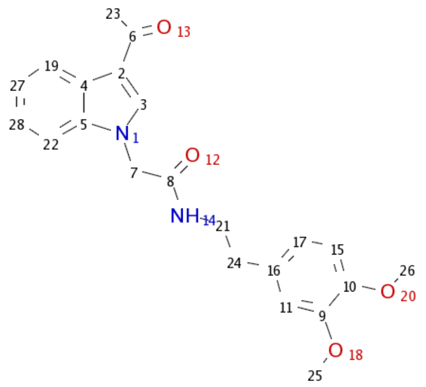

$C_{22}H_{24}N_2O_4$

Molecular Weight: 380.43696

ALogP: 3.001

Rotatable Bonds: 8

Acceptors: 4

Donors: 1

Model Prediction

Prediction: 8.151

Unit: g/kg_body_weight

Model Applicability

Missing features are fingerprint features in the query molecule not found in the training set.

1. OPS PC26 out of range. Value: 12.881. Training min, max, SD, explained variance: -7.9646, 10.26, 2.09, 0.0088.

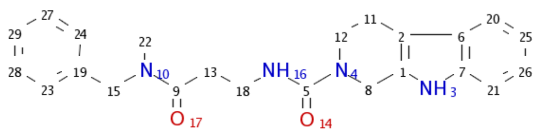

$C_{23}H_{26}N_4O_2$

Molecular Weight: 390.47814

ALogP: 2.702

Rotatable Bonds: 5

Acceptors: 2

Donors: 2

Model Prediction

Prediction: 5.277

Unit: g/kg_body_weight

Model Applicability

Missing features are fingerprint features in the query molecule not found in the training set.

1. All properties and OPS components are within expected ranges.

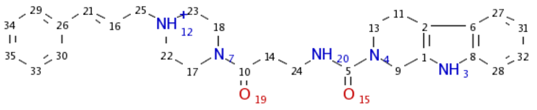

$C_{28}H_{34}N_5O_2$

Molecular Weight: 472.60186

ALogP: 1.567

Rotatable Bonds: 6

Acceptors: 2

Donors: 3

Model Prediction

Prediction: 5.145

Unit: g/kg_body_weight

Model Applicability

Missing features are fingerprint features in the query molecule not found in the training set.

1. All properties and OPS components are within expected ranges.
   Unknown FCFP_2 feature: 10: [*][NH+]([*])[*]
   Unknown FCFP_2 feature: -1853714334: [*]C[NH+](C[*])C[*]
   Unknown FCFP_2 feature: 1155241219: [*]CC[NH+]([*])[*]

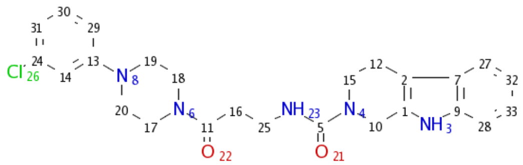

$C_{25}H_{28}ClN_5O_2$
Molecular Weight: 465.97512
ALogP: 3.413
Rotatable Bonds: 4
Acceptors: 3
Donors: 2

Model Prediction

Prediction: 2.972
Unit: g/kg_body_weight

Model Applicability

Missing features are fingerprint features in the query molecule not found in the training set.

1. All properties and OPS components are within expected ranges.

MOST ACTIVE.mol

TOPKAT_Rat_Oral_LD50

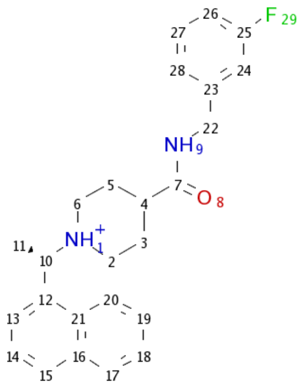

$C_{25}H_{28}FN_2O$

Molecular Weight: 391.50102

ALogP: 3.098

Rotatable Bonds: 5

Acceptors: 1

Donors: 2

Model Prediction

Prediction: 1.230

Unit: g/kg_body_weight

Model Applicability

Missing features are fingerprint features in the query molecule not found in the training set.

1. All properties and OPS components are within expected ranges.
   Unknown FCFP_2 feature: 10: [*][NH+]([*])[*]
   Unknown FCFP_2 feature: -1853714334: [*]C[NH+](C[*])C[*]
   Unknown FCFP_2 feature: 1155241219: [*]CC[NH+]([*])[*]
   Unknown FCFP_2 feature: -680623486: [*][NH+]([*])[C@H](C)[c](:[*]):[*]
